# Chalcone/1,3,4-Oxadiazole/Benzimidazole hybrids as novel anti-proliferative agents inducing apoptosis and inhibiting EGFR & BRAFV^600E^

**DOI:** 10.1186/s13065-023-01003-3

**Published:** 2023-09-16

**Authors:** Fatma Fouad Hagar, Samar H. Abbas, Hesham A. M. Gomaa, Bahaa G. M. Youssif, Ahmed M. Sayed, Dalia Abdelhamid, Mohamed Abdel-Aziz

**Affiliations:** 1 Medicinal Chemistry Department, Faculty of Pharmacy, Minia University, Minia, 61519 Egypt; 2 Pharmacology Department, College of Pharmacy, Jouf University, Sakaka, 72314 Saudi Arabia; 3 Pharmaceutical Organic Chemistry Department, Faculty of Pharmacy, Assiut University, Assiut, 71526 Egypt; 4 Pharmacognosy Department, Faculty of Pharmacy, Nahda University, Beni-Suef, 62513 Egypt

**Keywords:** Benzimidazole, Oxadiazole, Chalcone, Apoptosis, Anticancer Agents, EGFR, BRAF^V600E^

## Abstract

**Introduction:**

One of the most robust global challenges and difficulties in the 21st century is cancer. Treating cancer is a goal which continues to motivate researchers to innovate in design and development of new treatments to help battle the disease.

**Objectives:**

Our objective was developing new antiapoptotic hybrids based on biologically active heterocyclic motifs "benzimidazole?oxadiazole-chalcone hybrids'' that had shown promising ability to inhibit EGFR and induce apoptosis. We expected these scaffolds to display anticancer activity via inhibition of BRAF, EGFR, and Bcl-2 and induction of apoptosis through activation of caspases.

**Methods:**

The new hybrids **7a-x** were evaluated for their anti-proliferative, EGFR & BRAF^V600E^ inhibitory, and apoptosis induction activities were detected. Docking study & dynamic stimulation into EGFR and BRAF^V600E^ were studied.

**Results:**

All hybrids exhibited remarkable cell growth inhibition on the four tested cell lines with IC_50_ ranging from 0.95 μM to 12.50 μM. which was comparable to Doxorubicin. Compounds **7k-m** had the most potent EGFR inhibitory activity. While, compounds **7e, 7g, 7k** and **7l** showed good inhibitory activities against BRAF^V600E^. Furthermore, Compounds **7k, 7l,** and **7m** increased Caspases 3,8 & 9, Cytochrome C and Bax levels and decreased Bcl-2 protein levels. Compounds **7k-m** received the best binding scores and showed binding modes that were almost identical to each other and comparable with that of the co-crystalized Erlotinib in EGFR and BRAF active sites.

**Conclusion:**

Compounds **7k-m** could be used as potential apoptotic anti-proliferative agents upon further optimization.

**Supplementary Information:**

The online version contains supplementary material available at 10.1186/s13065-023-01003-3.

## Introduction

Treating cancer is a goal which continues to motivate researchers to innovate in design and development of new treatments to help battle the disease. Despite these efforts to combat cancer it is spreading quickly to all age groups and it is the second leading cause of death [1]. The global burden of cancer is estimated to climb to 28.4 million in 2040, representing a 47% increase over 2020 [2]. Lung, liver, stomach, breast, and colon cancer were the top five causes of cancer-related mortality worldwide [3]. Lung and breast cancers alone represent nearly one-fourth of all newly diagnosed cancer patients in 2020 [4 with lung cancer attributing to 18% of cancer mortality and breast cancer as the main cause of cancer mortality among women [5].

Apoptosis is an intriguing target for development of innovative cancer therapeutics. Normally, the apoptotic pathway becomes activated by DNA damage or uncontrolled proliferation [6]. There are two pathways that activate apoptosis: the intrinsic and extrinsic pathways which correlate with intracellular and extracellular signals [7]. The intrinsic pathway is activated in response to DNA damage and cytokine deprivation, whereas the extrinsic pathway is triggered by the immune system. The two pathways converge at the executioner caspases [3, 6, 7] which are a class of cysteine proteases that cleave target proteins required for normal cell function [8]. Activation of caspases results in plasma membrane changes and shrinking of apoptotic cells that eventually lead to cell death [9]. The intrinsic pathway is regulated by B-cell lymphoma-2 (Bcl-2) protein family which include proapoptotic effector proteins, proapoptotic BH3-only proteins, and antiapoptotic Bcl-2 proteins. Bcl-2 proteins inhibit apoptosis through inhibition of the proapoptotic Bcl-2 proteins, Bcl-2-associated X protein (BAX) and Bcl-2 homologous antagonist killer (BAK) [10]. BH3-only proteins inhibit the antiapoptotic Bcl-2 proteins [11].

Apoptosis evasion is essential for survival of cancer cells and leads to increasing invasiveness, stimulating angiogenesis, deregulation of cell proliferation, and interference with differentiation. Usually, the predominant methods of apoptosis evasion include inhibition of intrinsic pathway and caspase function, overexpression of antiapoptotic Bcl-2 proteins, and loss of BAX and/or BAK [12]. This results in resistance to any intrinsic apoptotic stimuli which includes some anticancer drugs [13].

Epidermal growth factor receptor (EGFR) and B-RAF play a role in regulating the mitogen-activated protein (MAP) kinase pathway which affects cell division, differentiation, and could lead to abnormal cell proliferation [14]. Signaling pathways of EGFR control angiogenesis, activation, and regulation of cellular proliferation. Overexpression of the EGFR gene and mutations of the EGFR tyrosine kinase domain were reported in the development and progression of different carcinomas including lung, colorectal, breast, brain, and pancreas [15]. Therefore these genetic alterations showed high probability to respond to EGFR small molecule tyrosine kinase inhibitors (TKI). Additionally, a recent study has shown that suppression of EGFR signaling was correlated to induction of intrinsic apoptosis in sensitive non-small cell lung cancer (NSCLC) EGFR-mutant cell lines [16]. However, EGFR inhibitors have exhibited limited efficacies and have been challenged by innate and acquired resistance in the clinic [17].

Erlotinib, is one of the most popular EGFR inhibitors which is currently marketed to treat many types of cancer with EGFR gene mutations (including non-small lung and pancreatic cancer) [18]. Its anticancer activity drives from suppressing intracellular phosphorylation of TK at the ATP binding site of the receptor, inhibiting JAK2V617F; a mutant version of JAK2 and inducing apoptotic cell death pathways [19]. Despite its high potency, selectivity, and acceptable safety profile, patients rapidly develop resistance within 8–12 months from the start of treatment *via* mutation in the ATP binding pocket of the EGFR kinase domain [20].

EGFR signaling is a part of a complex network that has been the target of effective cancer therapies. However, emergence of resistance is a major hurdle to developing an effective anticancer regimen [21]. The combination of an EGFR inhibitor and a BRAF inhibitor synergizes and improves anticancer activity through induction of apoptosis, as demonstrated by the combination of dabrafenib with cetuximab in colorectal cells [22]. Therefore, combination therapy that combines an anti-EGFR, anti-BRAF, and apoptotic agent may exhibit a multi-pronged approach that can be developed into a highly attractive and specific molecular oriented remedy.

In our continued research efforts to develop antiapoptotic hybrids based on biologically active heterocyclic motifs we have discovered that benzimidazole–oxadiazole-chalcone hybrids have shown promising ability to inhibit EGFR and induce apoptosis. [23–25]. Individually each motif has been reported as an effective anticancer scaffold with mechanisms of action as either EGFR inhibitor, BRAF inhibitor, or inducer of apoptosis [24, 26, 27]. In this study we explored new chemical architectures attaching the benzimidazole directly to oxadiazole and chalcone. In these compounds variably substituted aromatic rings were attached to the 2-position of benzimidazole. We envisioned this structural modification would afford improved compounds capable of interacting with EGFR and stimulating caspase-3 to induce apoptosis.

Based on the previous silico molecular docking simulations using MOE software for the most active compounds and Erlotinib into ATP binding sites it was found that Erlotinib forms 2 H-bond interactions with Met769 and Gln767 and one cation…π interaction with Leu 820. None of the earlier benzimidazole- chalcone hybrids showed identical binding interactions as Erlotinib with the same amino acid residues. Moreover, the most potent EGFR inhibitors had one H‐bonding interaction with Met769 as Erlotinib which meant this interaction was essential for activity. The new design moved the aromatic ring to a distal substitution on benzimidazole to introduce more flexibility in the structure. This modification allows the benzimidazole ring to be in closer proximity to more amino acids which are important for interaction with Erlotinib.

Herein, we report the design, synthesis and various biological evaluations of hybrid molecules formed from benzimidazole, oxadiazole, and chalcone derivatives aimed to improve anticancer activity by synergetic effect, decrease side effects, and minimize emergence of drug resistance. We expected these scaffolds to display anticancer activity *via* inhibition of BRAF, EGFR, and Bcl-2 and induction of apoptosis through activation of caspases. We present the synthetic strategy for preparing twenty-four new compounds, the results of detailed anticancer screening, and molecular docking. Lastly, mechanistic investigations of the most active compounds **7k**, **7L**, and **7m** regarding EGFR and BRAF inhibition and induction of caspases are described.

## Results and discussion

### Chemistry

**Fig. 1:**
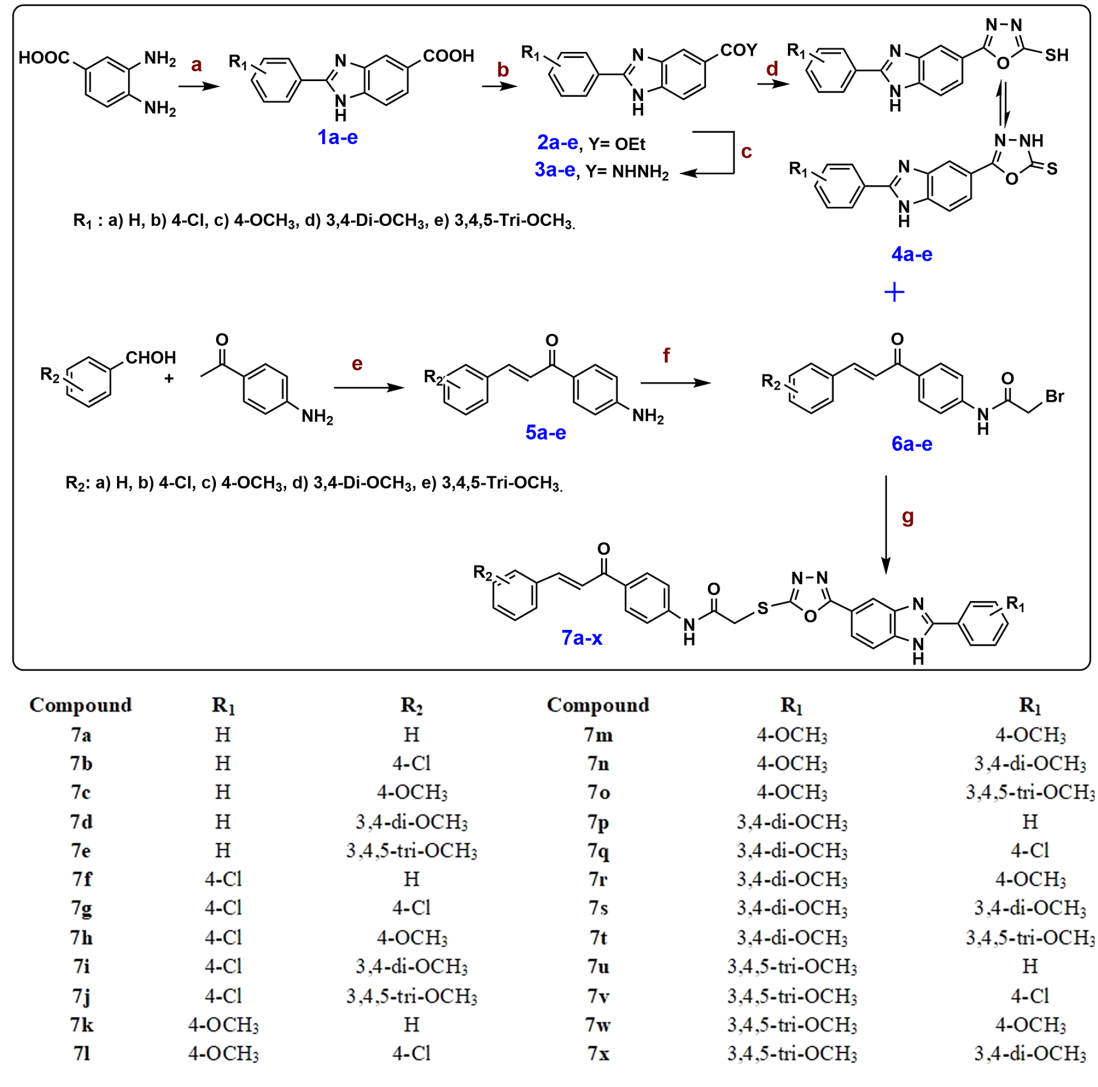
Scheme for synthesis of benzimidazole/chalcone hybrids 7a-x. **Reagent and reaction conditions:** **(a)** The appropriate sodium hydroxy(phenyl)methanesulfonate adduct, DMF, reflux 3-6 h; **(b)** EtOH, Conc. H_2_SO_4_, reflux 20 h; **(c)** NH_2_NH_2_, EtOH, reflux 3-7 h; **(d)** 1- CS_2_, KOH, EtOH, reflux 12 h.; 2- Conc HCl, (60.0-87.0%).; **(e)** KOH (60%), EtOH, stirring at 0 C for 3 h, then stirring at rt overnight; **(f)** BrCH_2_COBr, CH_2_Cl_2_, K_2_CO_3_, H_2_O, stirring overnight; **(g)** TEA, Acetonitrile, rt, 24-48 h

The series of chemical reactions that were employed to prepare targeted benzimidazole-chalcone hybrids **7a-x** is illustrated in Fig. 1. First, substituted 2-phenyl-1* H*-benzo[*d*]imidazole-5-carboxylic acids **1a-e** were synthesized through condensation of 3,4-diaminobenzoic acid with the appropriate aldehyde adduct in the presence of DMF for 3–6 h [28–29]. Fisher esterification of the resulting benzimidazole carboxylic acids **1a-e** with absolute ethanol using catalytic amount of concentrated H_2_SO_4_ as a dehydrating agent with heating for 20 h at reflux afforded the corresponding ethyl ester compounds **2a-e** [30]. Carbohydrazide compounds **3a-e** were prepared by heating the esters **2a-e** with hydrazine mono hydrate for 6–7 h at reflux [30]. Consequently, the carbohydrazide compounds **3a-e** were converted to 1,3,4 oxadiazole-benzimidazole derivatives **4a-e** by heating with carbon disulfide and KOH in absolute ethanol overnight [23, 29].

Chalcone intermediates **5a-e** were prepared by Claisen Schmidt condensation in the presence of KOH. A series of 2-bromo-*N*-(4-cinnamoylphenyl)acetamide derivatives [30].**6a-e** were synthesized by reaction of the appropriate chalcone with bromoacetyl bromide at 0^o^ C in a biphasic medium (DCM/water) using K_2_CO_3_ as a base [30]. Finally, the alkylation reaction that affords target compounds **7a**-**x** was accomplished by coupling the oxadiazole and acetylated chalcone in the presence triethylamine and acetonitrile as solvent in good yields ranging from 73–89% [30]. The structure of the new compounds was confirmed *vi*a NMR and mass spectral analyses. ^1^ H NMR spectra of compounds showed singlet signal or two singlets due to rotamers at δ: 4.14–4.46 ppm related to (S-CH_2_-CO). On other hand, in all compounds, the two protons of chalcone appear in the aromatic region as two doublet signals at δ: 7.56–7.74 ppm and δ: 7.80–8.16 ppm with coupling constant range *J* = 15.2–15.6. Additionally, the amide proton of the linker NH appears as singlet signal at δ: 10.83–11.16 ppm and benzimidazole proton NH appears as singlet signal at δ: 13.10-13.48 ppm. ^13^ C NMR spectra of compounds showed two carbonyl groups related to C = O of chalcone and N-C = O appeared at δ: 186.96-189.12 ppm and 160.22–169.50 ppm, furthermore, SCH_2_ appears at δ: 43.63–47.58 ppm.

### Biological evaluation

#### Anti-proliferative assays

##### In vitro one-dose anti-proliferative screening in NCI

The twenty-four synthesized compounds were submitted to the National Cancer Institute (NCI), USA. Eighteen hybrids **7a-h**, **7k-r**, **7u**, and **7v** were selected by NCI for one-dose assessment of their anti-proliferative activities at 10 µM dose by Sulforhodamine B colorimetric assay. The assessment was performed in 60 cell lines derived from nine tumor subpanels, comprising lung, leukemia, melanoma, colon, renal, CNS, prostate, breast, and ovarian cancer cell lines.

**Fig. 2:**
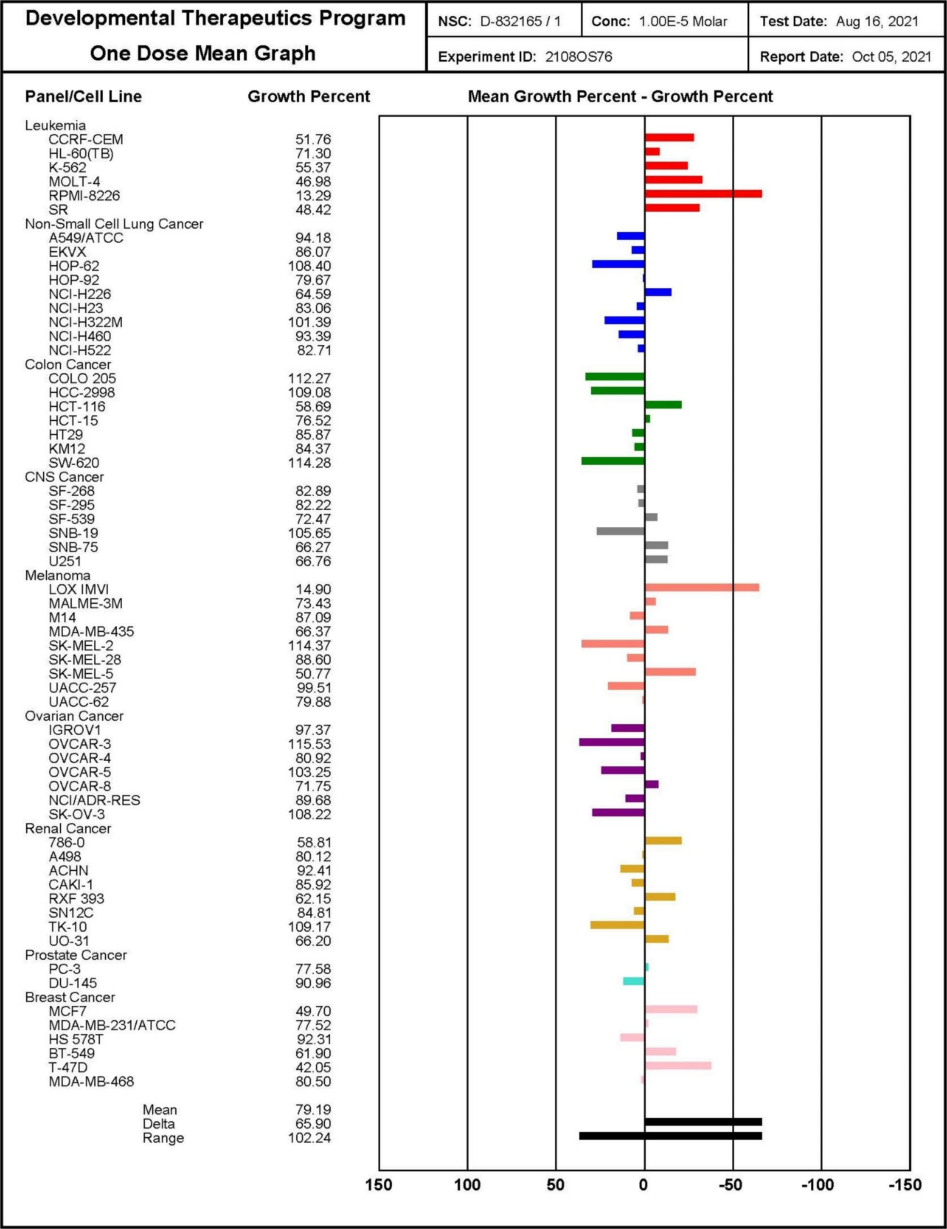
One dose mean graph for compound 7k on nine different cancer cell line panels at Conc 10 µM

The one-dose NCI results revealed that the tested hybrids displayed remarkable anti-proliferative activities especially hybrids **7e**, **7k**, and **7m-o** (Figs. 70, 71, 72, 73, 74, 75, 76, 77, 78, 79, 80, 81, 82, 83, 84, 85, 86, 87 and 88 in supporting information). The most potent compound was hybrid **7k** which displayed moderate to high anti-proliferative activities against eighteen cell lines including lung, leukemia, melanoma, colon, renal, breast, and CNS cancer cell lines with growth percentages ranging from 13 to 67% (Fig. 2). Moreover, eight compounds **7g**, **7h**, **7k-m**, **7p**, **7q**, and **7v** exhibited strong anti-proliferative activities in LOX IMVI with growth percentages ranging from − 32 to 18%. While **7e** and **7n** showed moderate activities against LOX IMVI with growth percentages 56% and 44%, respectively. In addition, **7e** and **7k** demonstrated moderate anti-proliferative activity against the CCRF-CEM cell line, with growth percent ranging from 38 to 52%. Compounds **7e**, **7g**, **7k**, and **7n** also displayed moderate anti-proliferative activities against the SR cell line with growth percentages ranging from 26 to 50%. Moreover, compounds **7c, 7e**, **7g**, **7k**, **7m**, **7p**, and **7v** exhibited notable anti-proliferative activity against the RPMI-8226 cell line with growth percentage range 4.95–56%. Furthermore, compound **7e**, **7k**, and **7n** showed moderate anti-proliferative activities in K-562 cell line with growth percent range 58%, 55% and 47% respectively. Also, compounds **7e** and **7k**, displayed moderate anti-proliferative activities against MOLT-4 cell line (growth percent 30% and 47%, respectively). Concerning HCT-116 cell line, four compounds; **7e**, **7k**, **7n**, and **7o** exhibited moderate activity against it with growth percentages equal to 40.76%, 58.69%, 57.96%, and 50.04%, respectively. On the other hand, compounds **7e**, **7g**, **7k**, **7n**, **7o**, and **7u-v** displayed moderate activities against MCF-7 cell line with growth percentages ranging from 42.88 to 59.08%. Additionally, compound **7e** displayed higher potency against proliferation of RXF 393 cell line with growth percent equal to -6.89%. Moreover, compounds **7c** and **7k** showed significant anti-proliferative activities against T-47D cell line with growth percentages equal to 52.35% and 42.05%, respectively.

##### In vitro anti-proliferative assay in LOX-IMVI

NCI in vitro anti-proliferative screening of compounds **7a-x** showed preferable activity against melanoma LOX-IMVI cell line over other cell lines. Specifically, five compounds; **7g**, **7l**, **7p**, **7q**, and **7v** showed remarkable anti-proliferative activities against it with growth percentages equal to -24.15%, -29.94%, -11.79%, 3.17%, and − 31.58%, respectively. Consequently, the half-maximal inhibitory concentration (IC_50_) of these compounds was determined in LOX-IMVI cell line using propidium iodide (PI)^31, 32^ with Staurosporine (broad spectrum protein kinase inhibitor) as a reference. Interestingly, the five compounds displayed an IC_50_ lower than that of Staurosporine (as shown in Table 1). It is noteworthy that four of these compounds contain the *para*-chloro chalcone motif (except **7p)**.

**Table 1:** IC_50_ of 7g, 7L, 7p, 7q, 7v, and Staurosporine in melanoma LOX-IMVI cell line upon 48 h incubation

**Compound**		**7g**	**7l**	**7p**	**7q**	**7v**	**Staurosporine**
**IC**_**50**_ **± SEM (µM)**		1.05 ± 0.10	0.80 ± 0.10	1.20 ± 0.10	2.50 ± 0.20	0.90 ± 0.10	7.10 ± 0.05

##### In vitro anti-proliferative assays in Panc-1, A-549, MCF-7, and HT-29 cells

The propidium iodide (PI) tests^31, 32^ were further used for assessment of the IC_50_ of all synthesized compounds and compared to Doxorubicin as a positive control against four cancer cell lines; pancreatic cancer cell line (Panc-1), epithelial line cancer cell (A-549), breast cancer cell line (MCF-7), and colon cancer cell line (HT-29).

**Fig. 3:**
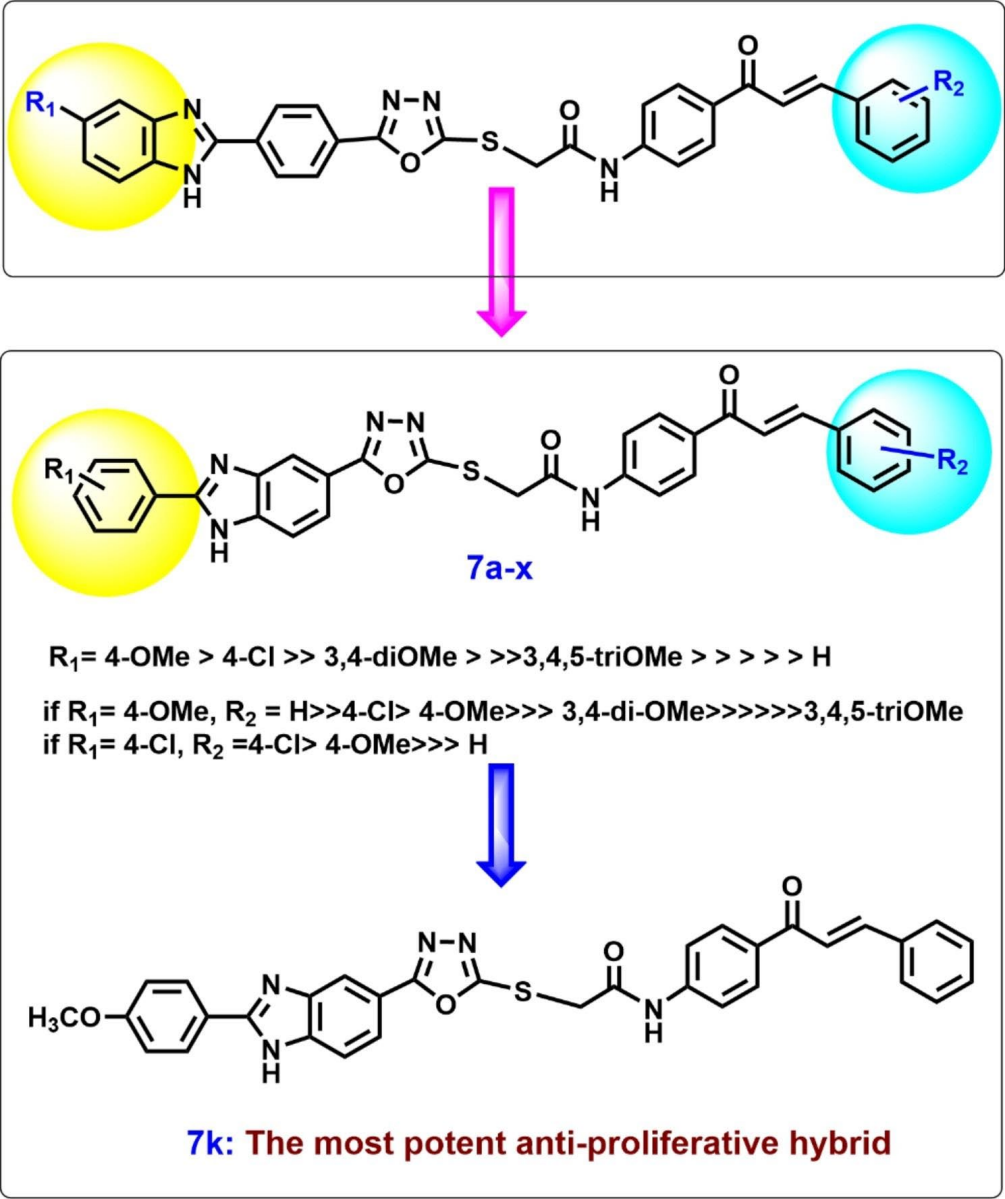
The effect of compounds derivatization on anti-proliferative activity

As presented in Table 2, all tested hybrids exhibited remarkable cell growth inhibition on the four tested cell lines with IC_50_ ranging from 0.95 µM to 12.50 µM which was comparable to Doxorubicin (IC_50_ ranging from 0.90 µM to 1.41 µM). The most effective compound was **7k** with IC_50_ ranging from 0.95 µM to 1.40 µM and the second most active one was **7L** with IC_50_ ranging from 1.10 µM to 1.65 µM. It was observed that the highest activity was displayed by compounds; **7k**, **7L**, and **7m** in which R_1_ = 4-OCH_3_. Additionally, these hybrids showed similar effects on the Panc-1, A-549, and HT-29 cell lines with slight variation in IC_50_ values. Our structure-activity relationship (SAR) analysis revealed that the anticancer activity is reliant on pattern of substitution in both phenyl rings (Fig. 3). Anticancer activity was correlated to substitution on phenyl ring attached to position two of benzimidazole ring with an effective substitution order of *p*-methoxy group > *p*-chlorine atom >>> 3,4-*di*methoxy groups. Further, it was noted that for optimal activity with p-chlorine on phenyl ring the substitution order on the chalcone phenyl ring was *p-* chlorine > *p*-methoxy >>> H.

**Table 2:** Cell viability % of MCF-10 A cells upon treatment with 50 µM of compounds 7a-x for 96 h and IC50 of compounds 7a-x & Doxorubicin in A-549, MCF-7, Panc-1 and HT-29 after 48 h incubation

Compound	Cell viability % in MCF-10 A (50 µM)	IC_50_ ± SEM (µM)			
		**A-549**	**MCF-7**	**Panc-1**	**HT-29**
7a	91	9.90 ± 1.10	9.20 ± 1.10	10.40 ± 1.30	11.15 ± 1.40
7b	88	8.20 ± 0.90	7.90 ± 0.80	8.40 ± 1.00	9.10 ± 1.10
7c	87	4.70 ± 0.50	4.15 ± 0.50	5.20 ± 0.40	5.50 ± 0.50
7d	89	9.70 ± 1.10	8.90 ± 1.10	10.30 ± 1.30	11.10 ± 1.40
7e	87	1.60 ± 0.30	1.25 ± 0.08	1.70 ± 0.30	1.95 ± 0.30
7f	87	2.15 ± 0.50	2.05 ± 0.50	2.40 ± 0.60	2.70 ± 0.50
7g	91	1.80 ± 0.60	1.45 ± 0.30	1.90 ± 0.50	2.10 ± 0.60
h	87	1.95 ± 0.60	1.75 ± 0.40	2.05 ± 0.50	2.25 ± 0.60
7i	84	9.70 ± 1.10	9.20 ± 1.00	10.15 ± 1.20	10.20 ± 1.30
7j	89	9.95 ± 1.10	9.40 ± 1.20	10.30 ± 1.20	10.70 ± 1.10
7k	91	1.20 ± 0.20	0.95 ± 0.08	1.30 ± 0.20	1.40 ± 0.20
7l	89	1.45 ± 0.30	1.10 ± 0.10	1.50 ± 0.30	1.65 ± 0.30
7m	94	1.40 ± 0.50	1.20 ± 0.08	1.50 ± 0.20	1.85 ± 0.40
7n	92	1.90 ± 0.60	1.65 ± 0.30	2.05 ± 0.50	2.20 ± 0.60
7o	89	7.60 ± 0.30	7.10 ± 0.60	8.20 ± 1.20	8.60 ± 0.40
7p	87	2.50 ± 0.50	2.10 ± 0.20	3.10 ± 0.40	3.30 ± 0.40
7q	94	3.40 ± 0.40	3.02 ± 0.30	3.80 ± 0.50	3.70 ± 0.50
7r	91	3.60 ± 0.50	3.10 ± 0.50	3.90 ± 0.50	4.20 ± 0.50
7s	87	3.80 ± 0.60	3.15 ± 0.50	4.30 ± 0.60	4.55 ± 0.60
7t	88	10.60 ± 1.10	10.10 ± 1.00	10.90 ± 1.20	10.80 ± 1.20
7u	90	2.25 ± 0.50	2.10 ± 0.08	2.40 ± 0.20	2.70 ± 0.50
7v	87	2.45 ± 0.50	2.20 ± 0.08	2.60 ± 0.20	2.90 ± 0.50
7w	91	10.90 ± 1.10	10.20 ± 1.20	11.15 ± 1.20	11.20 ± 1.30
7x	85	11.50 ± 1.20	10.800 ± 1.10	11.80 ± 1.20	12.50 ± 1.50
Doxorubicin	-	1.21 ± 0.10	0.90 ± 0.10	1.41 ± 0.10	1.01 ± 0.10

##### In vitro toxicity assay in normal cells (cell viability assay)

To check the safety of prepared compounds and evaluate their selectivity towards tumor cells over normal ones, the toxicity of compounds **7a-x** were evaluated by 3-(4,5-Dimethylthiazole-2-yl)-2,5-diphenyltetrazol (MTT) assay on the normal human mammary epithelial cell line (MCF-10 A) [33, 34]. The human mammary epithelial cell lines were incubated for four days with a concentration of 50 µM of compounds **7a-x**. All compounds showed cell viability of more than 80% (Table 2.). The cytotoxic activity of **7k** against MCF-7 was 96-fold higher than its toxicity against MCF-10 A. Similarly compounds **7L** and **7v** were 111 and 97-fold more selective towards LOX-IMVI cell line respectively.

#### EGFR inhibitory activity assay

To explore the molecular mechanism of the synthesized hybrids, an assessment of the EGFR inhibitory ability of the most potent ten compounds was performed using the EGFR-TK assay [35, 36]. The most active compounds; **7e**, **7g**, **7h**, **7k-n**, **7p**, **7q**, and **7v** were selected to determine their enzymatic inhibitory activity and compared to Erlotinib and the findings are included in Table 3. The results showed that the tested compounds inhibited EGFR with IC_50_ values ranging from 0.55 µM to 3.90 µM. The most effective compound **7k** (R_1_ = 4-OCH_3_, R_2_ = H) demonstrated the greatest inhibitory efficacy against EGFR with an IC_50_ of 0.55 µM, being approximately 7-fold less potent than Erlotinib (IC_50_ = 0.08 µM). Compounds **7L** and **7m** rank second and third in activity, with IC_50_ values of 0.80 µM and 0.90 µM, respectively. The remaining compounds displayed weak inhibitory activity against EGFR, with IC_50_ values ranging from 1.15 µM to 3.90 µM. Consequently, compounds **7k**, **7L**, and **7m** were considered promising agents which could be used as potential anti-proliferative agents targeting EGFR-TK after optimization.

#### BRAF^V600E^ inhibitory activity

An in vitro study was carried out to assess the anti-BRAF^V600E^ activity of ten compounds namely, **7e**, **7g**, **7h**, **7k-n**, **7p**, **7q**, and **7v** and compared to Erlotinib [24, 37]. The enzyme assay revealed that all ten compounds inhibited BRAF^V600E^ with IC_50_ values ranging from 0.90 µM to 4.70 µM (as shown in Table 3). Compounds **7e**, **7g**, **7k** and **7l** showed good inhibitory activities against BRAF^V600E^ (IC_50_ = 0.90 µM, 1.00, µM, 1.70 µM, and 1.90 µM, respectively) and they were revealed to be potent inhibitors of cancer cell proliferation as well as promising EGFR inhibitors (IC_50_ = 1.15 µM, 1.70 µM, 0.55 µM and 0.80 µM respectively). These encouraging results suggest that the favourable anti-proliferative activity of these compounds is mediated through inhibition of both EGFR and BRAF^V600E^.

**Table 3:** Inhibitory activities of selected compounds against EGFR and BRAFV600E

Compound	EGFR Inhibition IC_50_ ± SEM (µM)	BRAF ^V600E^ Inhibition IC_50_ ± SEM (µM)
**7e**	1.15 ± 0.20	0.90 ± 0.10
**7g**	1.70 ± 0.20	1.00 ± 0.20
**7h**	2.30 ± 0.20	2.70 ± 0.30
**7k**	0.55 ± 0.10	1.70 ± 0.20
**7L**	0.80 ± 0.10	1.90 ± 0.20
**7m**	0.90 ± 0.10	2.20 ± 0.20
**7n**	1.40 ± 0.20	3.70 ± 0.20
**7p**	3.50 ± 0.30	4.20 ± 0.30
**7q**	3.90 ± 0.30	4.70 ± 0.30
**7v**	2.80 ± 0.30	3.10 ± 0.20
**Erlotinib**	0.08 ± 0.01	0.06 ± 0.01

#### Apoptosis induction activity

##### Caspases assays

The effects of the most active compounds; **7k**, **7l**, and **7m** on caspases (3, 8, and 9) were evaluated and compared to Doxorubicin treated and untreated cells [38]. The results showed that the tested compounds increased the level of active caspase-3 by 7–8 folds when compared to control cells; and that compounds **7k**, **7l**, and **7m** induced remarkable overexpression of caspase-3 protein level (501.60 ± 4.00, 492.50 ± 4.00 and 473.60 ± 3.50 pg/mL, respectively) and were comparable to Doxorubicin (503.50 ± 4.50 pg/mL). Compared to the untreated cells, the most active anti-proliferative derivative **7k** showed an 8-fold increase in caspase-3 level. The effect of compounds **7k**, **7l**, and **7m** on caspases 8 and 9 were also investigated to underline the role of intrinsic and extrinsic apoptotic pathways in these compounds’ anti-proliferative actions. As shown in Table 4, compound **7k** increased caspase 8 and 9 levels in comparison to untreated cells by 10 and 17 folds, respectively. Compound **7l** increased caspase 8 and 9 levels by 9 and 16 folds, respectively. These results indicated that the new hybrids were capable of activating both the intrinsic and extrinsic pathways, with a stronger impact on the intrinsic pathway because caspase 9 levels were higher.

**Table 4:** Effects of compound 7k, 7l, and 7m on caspases activity and amount of Cytochrome C in MCF-7 breast cancer cells

Compound	Caspase-3		Caspase-8		Caspase-9		Cytochrome C	
	Conc (pg/mL)	Fold change	Conc (ng/mL)	Fold change	Conc (ng/mL)	Fold change	Conc (ng/mL)	Fold change
**7k**	501.600 ± 4.00	7.60	1.680	9.90	15.900	17.00	0.660	14.00
**7l**	492.500 ± 4.00	7.50	1.570	9.30	15.100	16.00	0.595	13.00
**7m**	473.600 ± 3.50	7.20	1.350	7.90	13.900	15.00	0.515	11.00
**Doxorubicin**	503.200 ± 4.20	7.65	1.750	10.00	16.200	17.40	0.604	13.10
**Control**	65.600	1.00	0.170	1.00	0.930	1.00	0.046	1.00

##### Cytochrome C assay

The amount of cytochrome C in the cell is critical for caspase activation and the beginning of the intrinsic apoptosis process [31]. The results of testing compounds **7k**, **7l**, and **7m** as Cytochrome C activators were shown in Table 4. Compounds **7k**, **7l**, and **7m** increased Cytochrome C levels by 14, 13, and 11 times, respectively, when compared to untreated control cells. These results support the assumption that Cytochrome C overexpression and the activation of intrinsic apoptotic pathway were the causes of apoptosis.

##### Expression levels of BAX and Bcl-2 proteins

The effects of compounds **7k**, **7l**, and **7m** on BAX and Bcl-2 protein levels in the MCF-7 breast cancer cell lines were studied further using Doxorubicin as a reference for comparison [39]. The results in Table 5 showed that as compared to Doxorubicin, the compounds **7k**, **7l**, and **7m** caused a significant increase in BAX levels. Compound **7k** induction of BAX level (279.50 pg/mL) was similar to Doxorubicin (276 pg/mL) with a 33-fold increase over control untreated cancer cell, followed by compound **7l** (271 pg/mL and 32-fold rise). Finally, compound **7k** reduced antiapoptotic Bcl-2 protein levels in MCF-7 cells to 0.94 ng/mL, followed by compound **7l** (0.97 ng/mL) in comparison to Doxorubicin (0.98 ng/mL).

**Table 5:** Effects of compound 7k-m on the protein expression levels of BAX and Bcl-2

Compound	BAX		Bcl-2	
	Conc (pg/mL)	Fold change	Conc (ng/mL)	Fold reduction
**7k**	279.500	33.0	0.940	5.4
**7l**	271.600	32.0	0.975	5.2
**7m**	268.200	31.5	1.050	4.9
**Doxorubicin**	276.500	32.5	0.985	5.1
**Control**	8.500	1.0	5.080	1.0

#### Cell cycle analysis and apoptosis detection

##### Cell cycle analysis

**Fig. 4:**
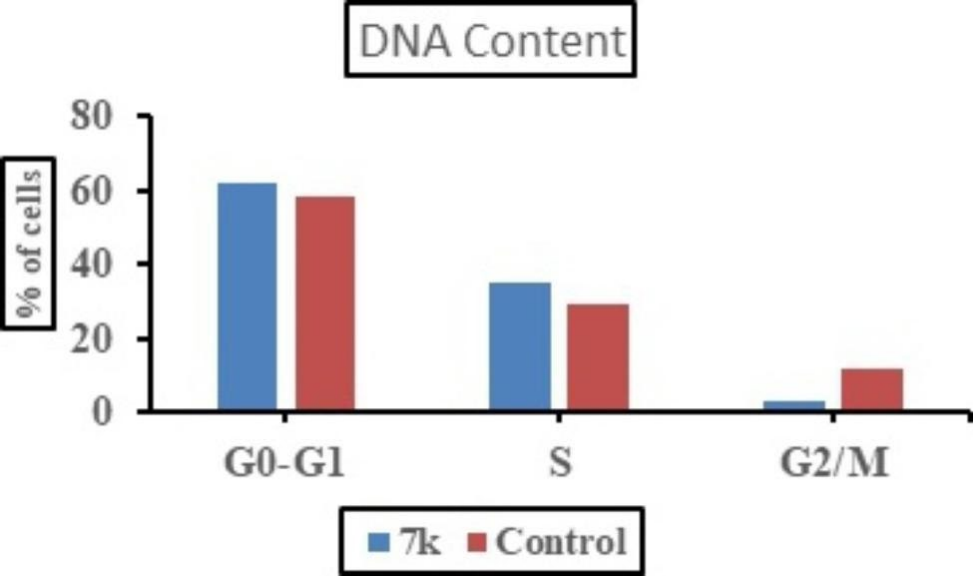
Results of cell cycle analysis in untreated MCF-7 cells (Control) and MCF-7 cells treated with IC_50_ (0.95 µM) of hybrid 7k for 24 h

Compound **7k** was applied to MCF-7 cancer cell line with IC_50_ concentration (0.95 µM) to assess its effect on cell cycle progression and apoptosis induction. Data obtained from cell cycle analysis (Fig. 4) revealed that **7k** significantly increased the percentage of accumulation of MCF-7 cells in G0-G1 and **S** phase 61.95% and 34.86% respectively as compared to 58.27% and 29.51% in untreated cells. On other hand, incubation with **7k** decreased percentage accumulation in the G2/M phase to 3.19% versus 12.22% in control. From the previous results, it was concluded that compound **7k** might arrest cells at G1/S and prevent it from entering G2/M phase as indicated by decreased percentage of accumulation of cells in G2/M phase compared to control.

##### Apoptosis detection

**Fig. 5:**
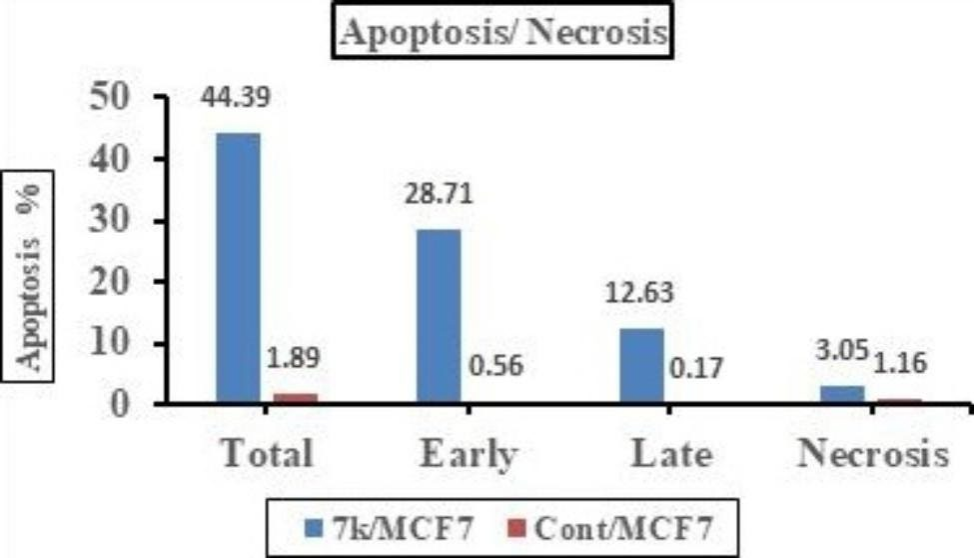
Apoptosis and necrosis percentage in MCF-7 cells after incubation with DMSO (control) and with IC_50_ of compound 7k (0.95 µM) for 24 h

**Fig. 6:**
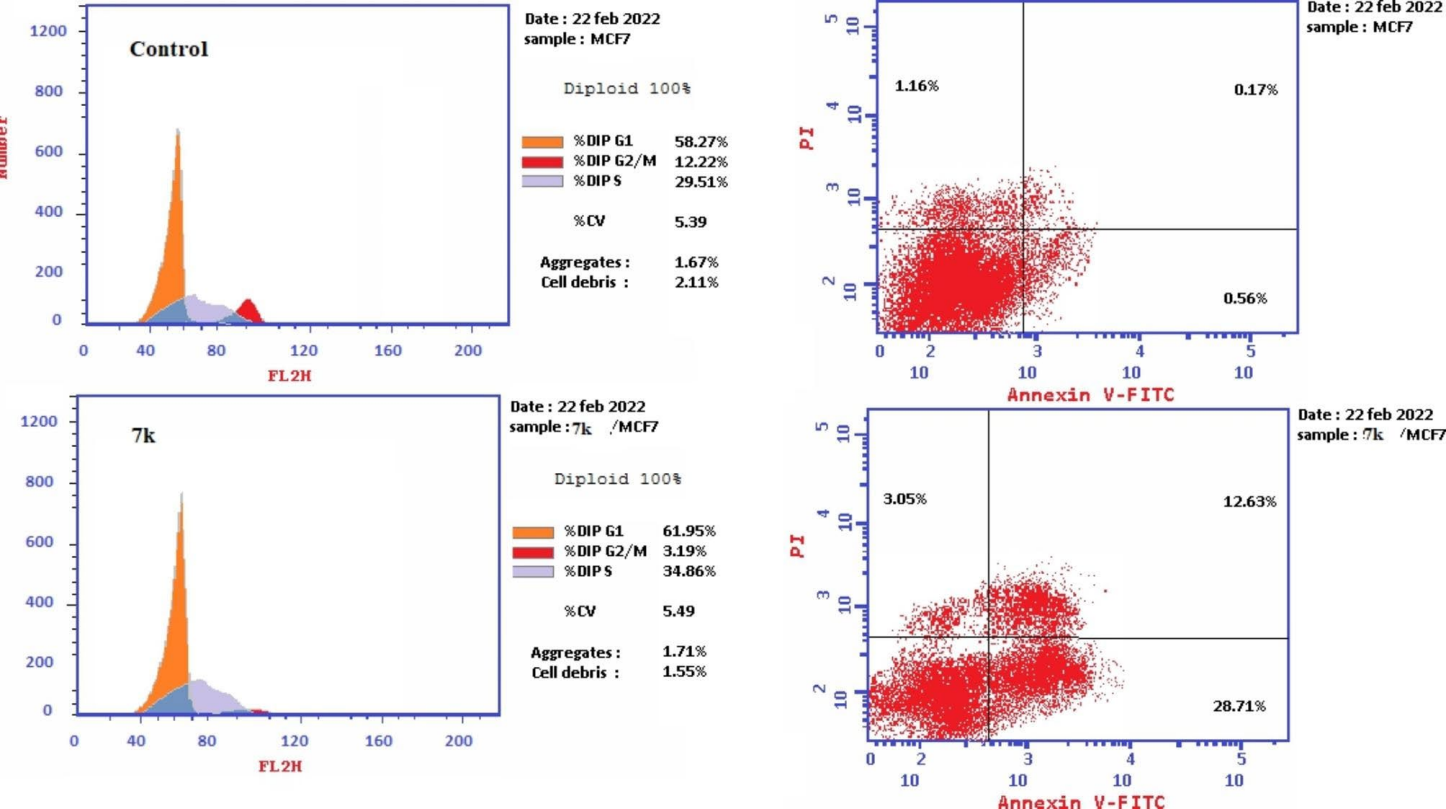
Cell cycle and apoptosis induction analysis of MCF-7 cells after incubation with IC_50_ of compound 7k (0.95 µM) for 24 h using Annexin V/PI compared to control untreated MCF-7

To further study the root for cytotoxic activity of compound **7k**, Annexin V-FITC/PI assay method^40, 41^ was used to investigate its capability to induce apoptosis. In this assay MCF-7 cell line was treated with IC_50_ concentration (0.95 µM) of **7k** and stained with Annexin V/PI then incubated for 24 h. Results showed that compound **7k** increased the level of both early and late apoptosis to 28.71% and 12.63% respectively whereas necrosis level was only 3.05% (Figs. 5 and 6). These cumulative results further indicate that compound **7k** showed potent anti-proliferative activity by induction of apoptosis.

### Docking study into EGFR and BRAF^V600E^

**Fig. 7:**
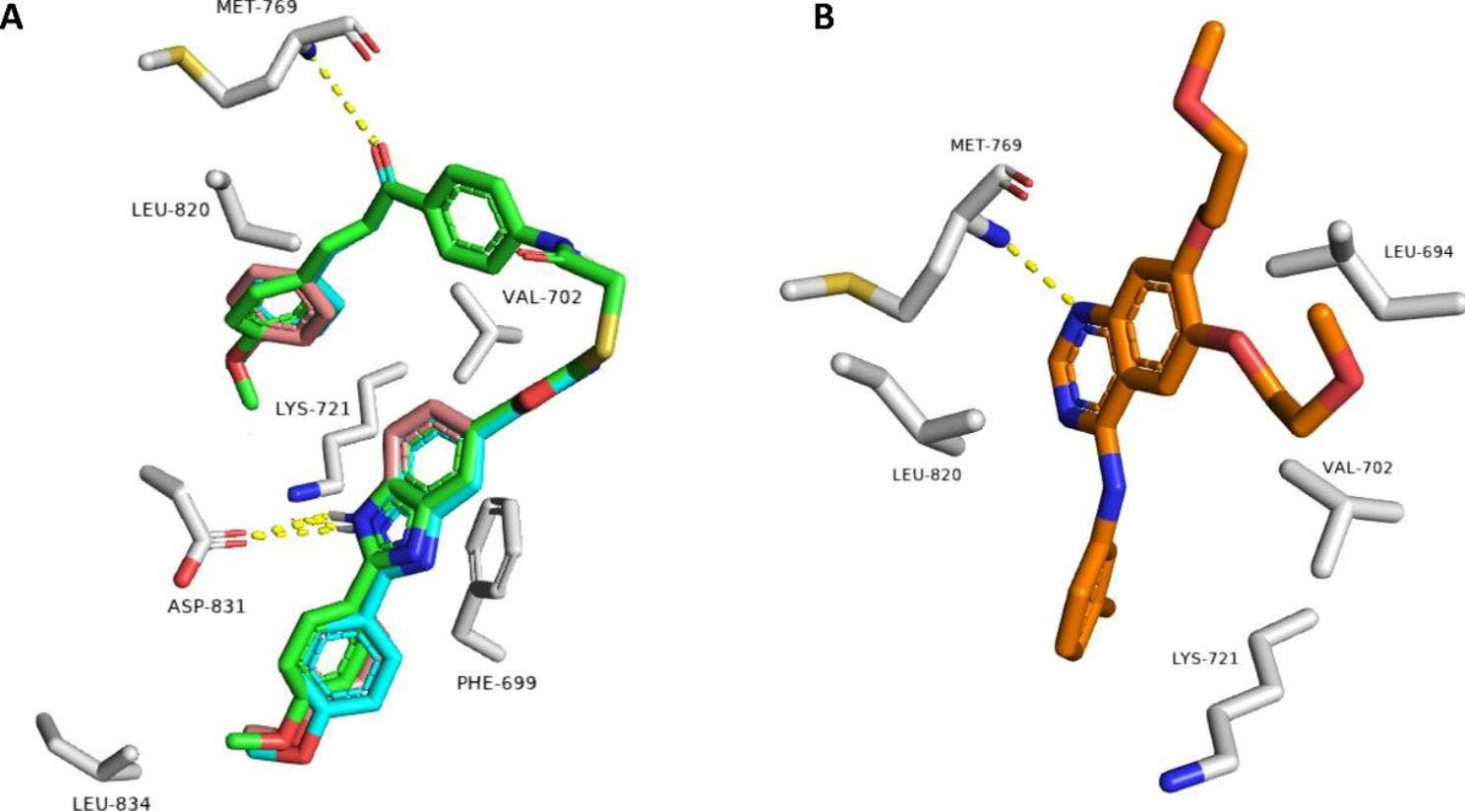
A: Binding modes of compounds 7k, 7l, 7m (green, cyan, and brick red structures, respectively) inside the binding site of kinase domain of human EGFR (PDB ID: 1M17) B: Kinase domain of EGFR with the co-crystalized Erlotinib (orange-colored structure)

**Fig. 8:**
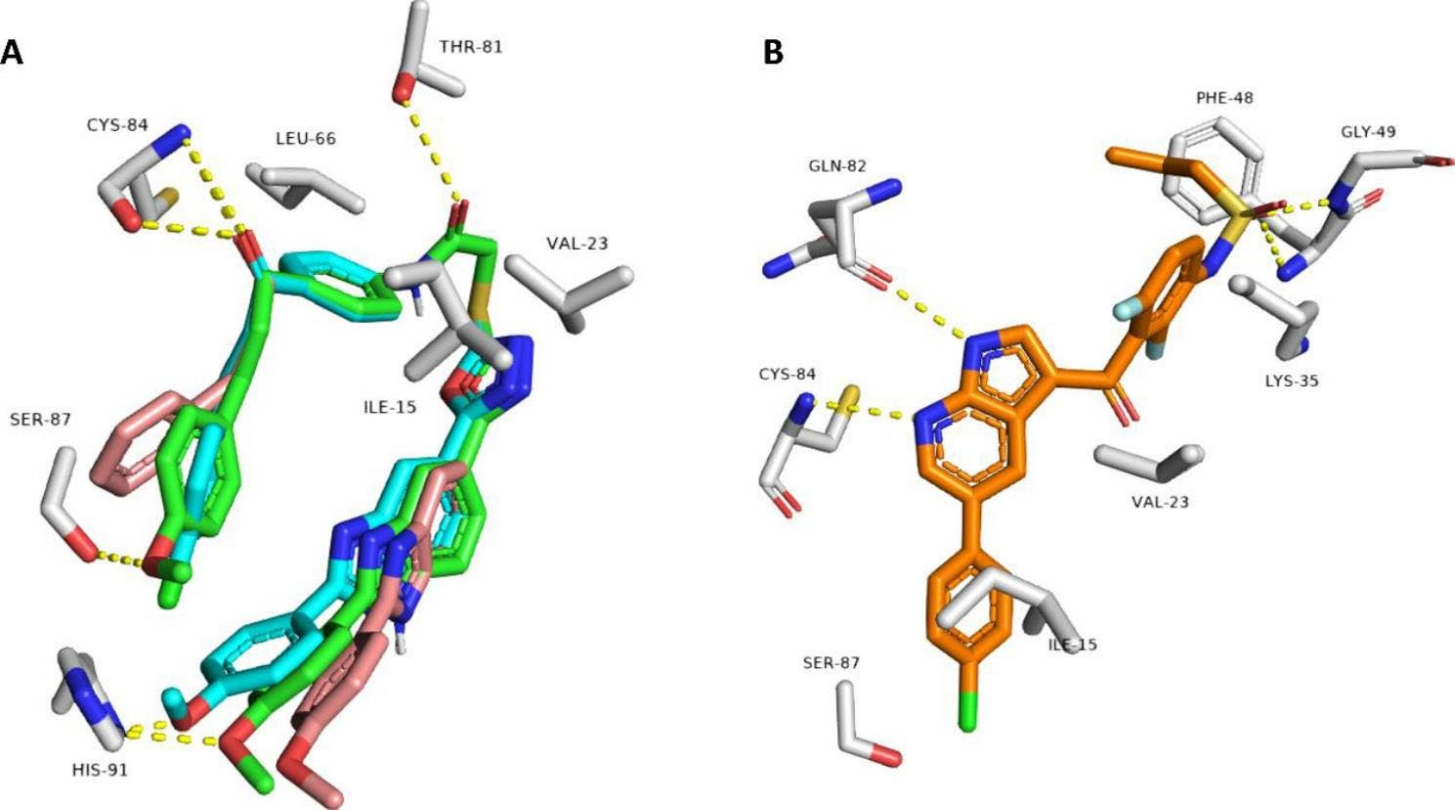
A: Binding modes of compounds 7k, 7l, 7m (green, cyan, and brick red structures, respectively) inside the binding site of kinase domain of human BRAF (PDB ID: 3OG7) B: Kinase domain of EGFR with the co-crystalized Erlotinib/Vemurafenib (orange-colored structure)

The structures of the most bioactive derivatives (i.e., **7e**, **7g**, **7h**, **7k-n**, **7p**, **7q**, and **7v**) were docked inside the active sites of EGFR and BRAF to investigate their binding interactions with these proteins (Figs. 7 and 8). The resulting docking scores of these derivatives were convergent (i.e., docking scores ranged from − 8.4 to -10.7 kcal/mol, Table 6) and comparable with that of the co-crystalized ligands Erlotinib and vemurafenib, respectively.

**Table 6:** Results of the docking compounds 7e, 7g, 7h, 7k-n, 7p, 7q, and 7v into the active sites of EGFR (PDB ID: 1M17) and BRAFV600E (PDB ID: 3OG7) in comparison with their co-crystallized ligands

Compound	Docking score EGFR	Docking score BRAF^V600E^	Interactions			
			H-bonding		Hydrophobic	
			EGFR	BRAF	EGFR	BRAF
**7e**	-8.4	-8.5	-	SER-87, HIS-91	PHE-699, VAL-702	VAL-23, LEU-66
**7g**	-9.2	-9.4	-	SER-87, HIS-91	PHE-699, LEU-834, LEU-820,	ILE-15, VAL-23
**7h**	-9.2	-9.9	-	CYS-84, SER-87, HIS-91	VAL-702, LEU-834	ILE-15, VAL-23, LEU-66
**7k**	-10.6	-9.8	MET-769, ASP-831	THR-81, CYS-84, SER-87, HIS-91	PHE-699, VAL-702, LEU-820, LEU-834	ILE-15, VAL-23, LEU-66
**7l**	-10.7	-9.9	MET-769, ASP-831	THR-81, CYS-84, HIS-91	PHE-699, VAL-702, LEU-820, LEU-834	ILE-15, VAL-23, LEU-66
**7m**	-10.4	-9.9	MET-769, ASP-831	THR-81, CYS-84,	PHE-699, VAL-702, LEU-820, LEU-834	ILE-15, VAL-23, LEU-66
**7n**	-10.1	-9.5	MET-769	THR-81, CYS-84	PHE-699, LEU-820, LEU-834	ILE-15, VAL-23, LEU-66
**7p**	-9.1	-9.0	-	THR-81	PHE-699, VAL-702, LEU-820	ILE-15, VAL-23
**7q**	-8.8	-9.1	MET-769	CYS-84	PHE-699, VAL-702, LEU-820	VAL-23, LEU-66
**7v**	-9.3	-9.1	ASP-831	THR-81, CYS-84	PHE-699, LEU-820, LEU-834	VAL-23, LEU-66
**Erlotinib**	-9.7	-9.5	MET-769	-	LEU-694, VAL-702 LEU-820	-
**Vemurafenib**	-	-9.6	-	GLN-82, CYS-84	-	ILE-15, VAL-23, LYS-35, PHE-48

With regards to EGFR, compounds **7k**, **7l**, and **7m** received the best binding scores (-10.6, -10.7, and − 10.4, respectively). Additionally, they showed binding modes that were almost identical to each other (RMSD = 0.79 Å) and comparable with that of the co-crystalized Erlotinib **(**docking score = -9.7 kcal/mol).

As shown in Fig. 7, each structure was able to form 2 H-bonds with MET-769 and ASP-831 amino acid residues, while the co-crystalized Erlotinib formed a single H-bond with MET-769. Additionally, the three structures (i.e., **7k**, **7l**, & **7m**) showed the same hydrophobic interactions with PHE-699, VAL-702, LYS-721, LEU-820, and LEU-834 which also interact with Erlotinib.

Similarly, **7k, 7l,** and **7m** were also the best-scoring compounds upon docking inside BRAF’s active site (docking scores = -9.8, -9.9, and − 9.9 kcal/mol, respectively). Moreover, they showed identical binding modes (RMSD = 0.87 Å) comparable with that of the co-crystalized inhibitors Vemurafenib and Erlotinib (docking scores = -9.6 and − 9.5 kcal/mol, respectively) (Fig. 8). The structures of **7k, 7l,** and **7m** were able to establish the same hydrophilic interactions (i.e. H-bonds) with THR-81, CYS-84, HIS-91, except for compound **7m** which formed additional H-bond through its additional methoxy group with SER-87 (Fig. 8A). Furthermore, the three structures also shared hydrophobic interactions with ILE-15, VAL-23, and LEU-66. Vemurafenib (the co-crystalized inhibitor) established a slightly different binding mode, where it formed 2 H-bonds with GLN-82 and CYS-84, and four hydrophobic interactions with ILE-15, VAL-23 (similarly to compounds 7k-m), LYS-35, and PHE-48 (Fig. 8B).

**Fig. 9:**
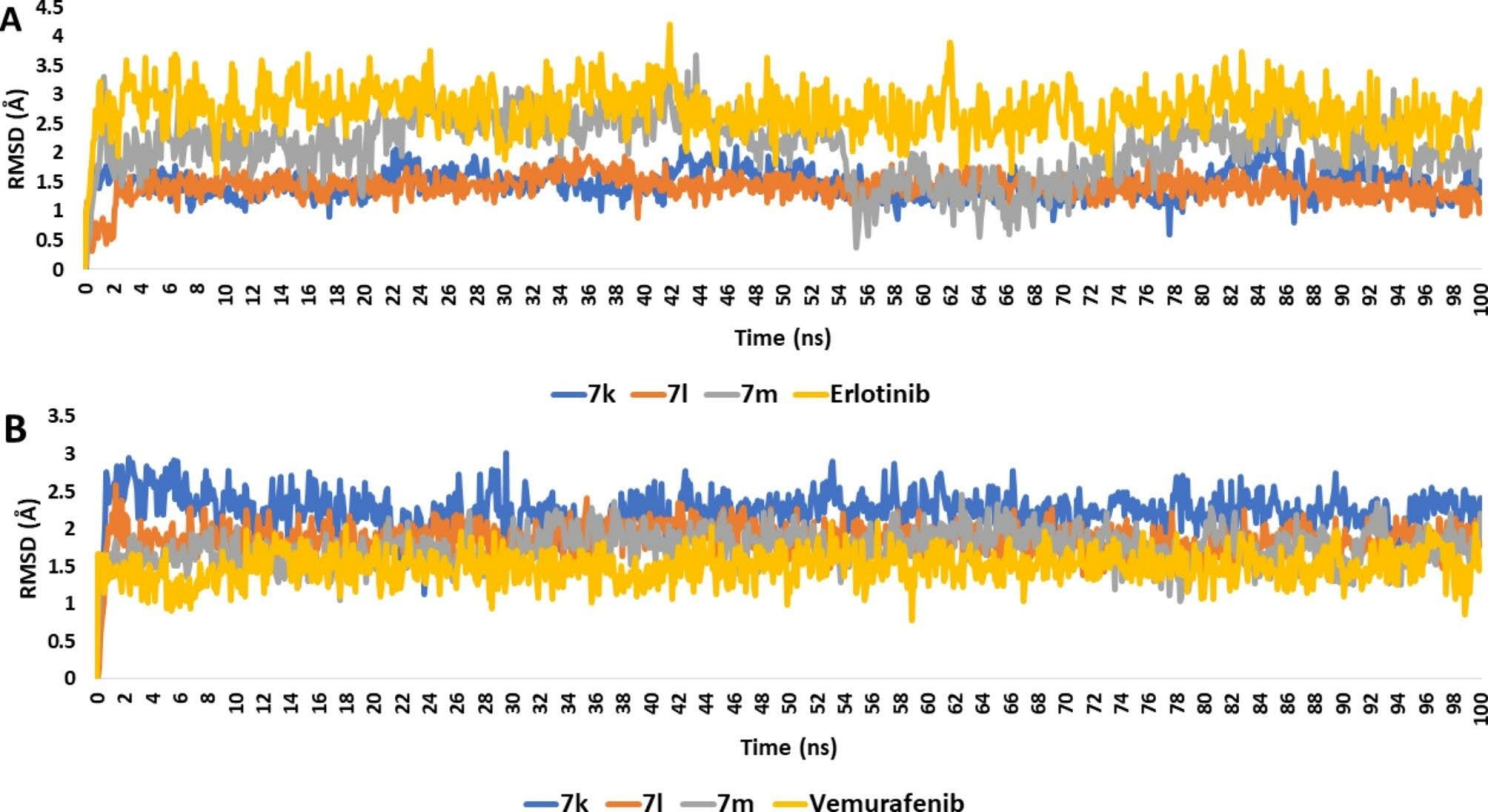
RMSDs of 7k, 7l, and 7m inside the kinase domains of both EGFR and BRAF (A and B, respectively) in comparison with the previously reported co-crystalized inhibitors (i.e., Erlotinib and Vemurafenib, respectively) over the course of 100 ns-long MD simulations

To further validate the docking results, the best docking poses for **7k**, **7l**, and **7m** inside the kinase domains of both EGFR and BRAF were subjected to 100 ns-long MD simulations. As shown in Fig. 9, the structures of the three compounds (i.e., 7k, 7L, and 7m) exhibited significant stability inside the active sites of both enzymes with low deviations from their initial state (i.e., docked poses) (Average RMSDs ranged from 1.9 Å to 2.2 Å). Accordingly, the calculated absolute binding free energy (Δ*G*_Bind_) for each compound using the MM-PBSA method were convergent and comparable with the co-crystalized inhibitors (Erlotinib and Vemurafenib, respectively) (Table 7) [42]. Judging from the previous in silico structural analysis, it can be concluded that the derivatives **7k**, **7L**, and **7m** are considered promising structure motifs acting as EGFR and BRAF dual inhibitors.

**Table 7:** The calculated Δ*G*_Bind_ (in kcal/mol) of 7k, 7l, and 7m inside the kinase domains of both EGFR and BRAF (A and B, respectively) in comparison with the previously reported co-crystalized inhibitors (i.e., Erlotinib and Vemurafenib, respectively)

Energy Component	7k		7l		7m		Erlotinib	Vemurafenib
	EGFR	BRAF	EGFR	BRAF	EGFR	BRAF	EGFR	BRAF
Δ*G* gas	-49.3409	-53.6574	-54.9876	-53.6723	-41.8203	-49.5564	-48.1047	-48.4837
Δ*G* solv	14.8213	16.4637	16.7483	17.3443	11.2943	14.4463	13.6452	16.3827
Δ*G* TOTAL	-34.5196	-38.2393	-38.2393	-36.328	-30.526	-35.1101	-34.4595	-32.101

### In silico prediction of physicochemical and pharmacokinetic properties

**Fig. 10:**
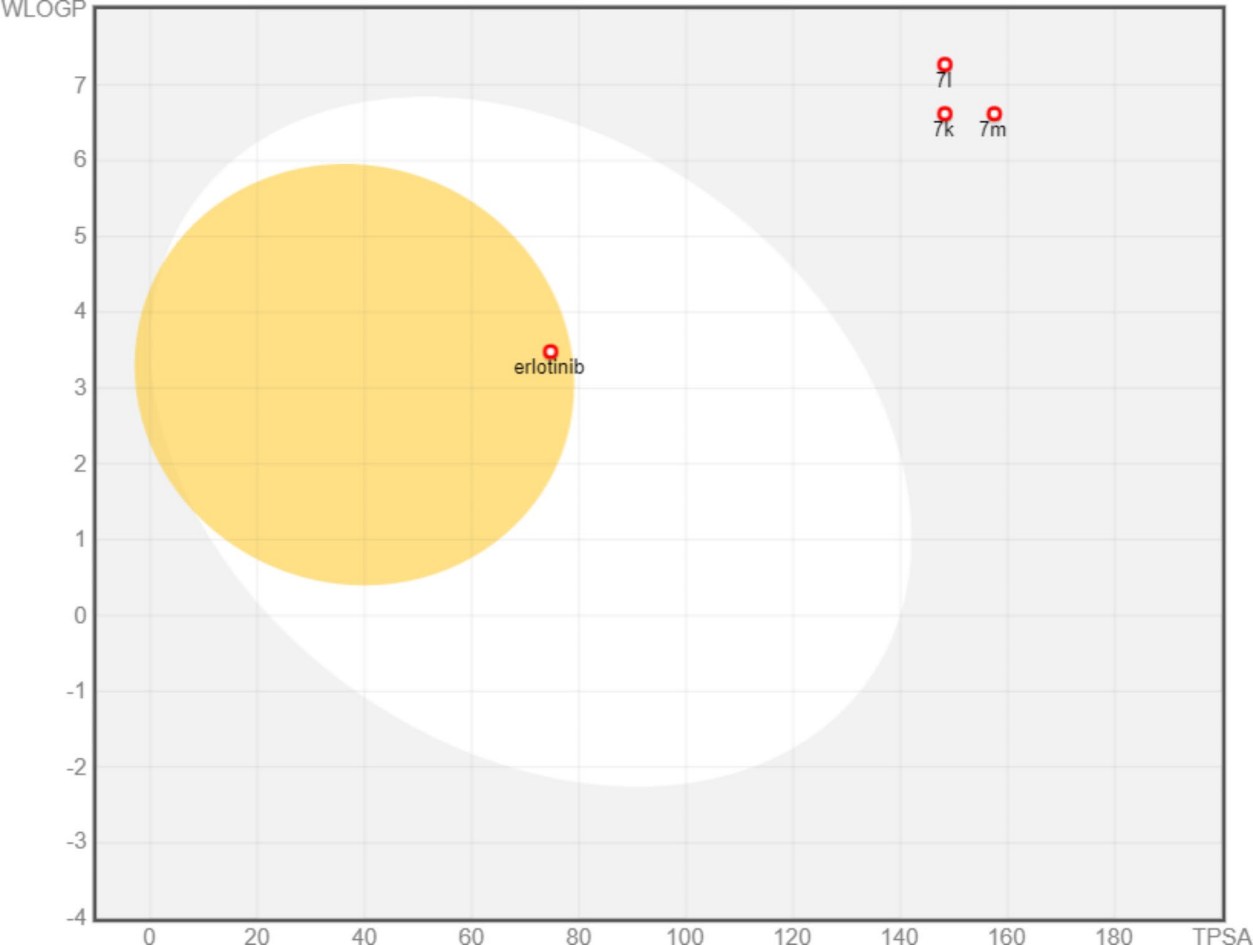
Forecast Boiled-Egg plotting from Swiss ADME online website for scaffolds **7k**, **7l**, **7m**, and **Erlotinib**

**Fig. 11:**
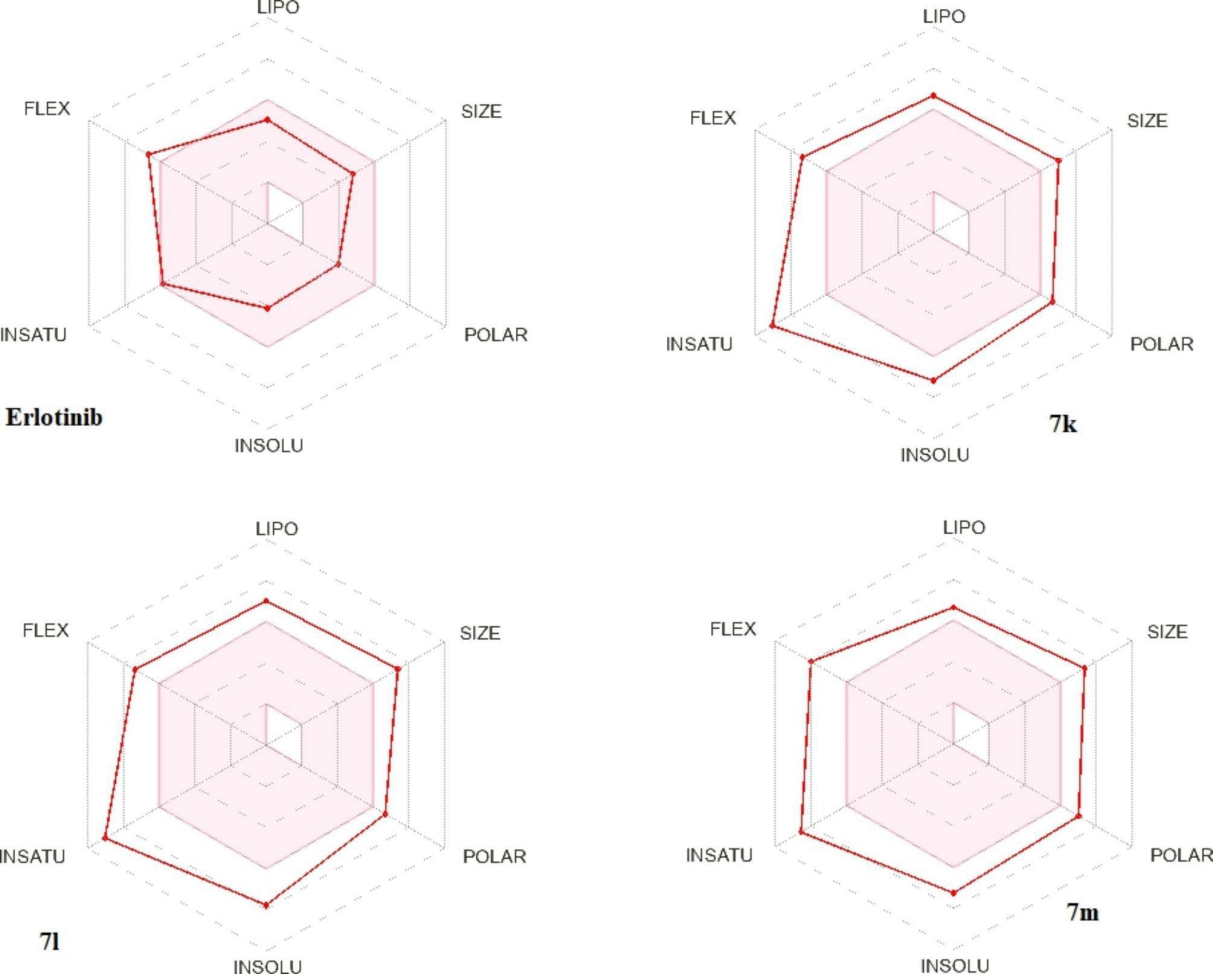
Radar bioavailability diagram from Swiss ADME website for scaffolds **7k**, **7l**, **7m**, and **Erlotinib**. The pink area characterizes the optimum property values range for the oral bioavailability, and the red lines represent the forecasted characters

In this study, we used two web servers Swiss ADME (http://www.swissadme.ch/index.php) and PKCSM (http://biosig.unimelb.edu.au/pkcsm/) to investigate the physicochemical and pharmacokinetic features of compounds **7k**, **7l** and **7m**. Swiss ADME affords information about SILICOS-IT, MLOGP, iLOGP, XLOGP3, and WLOGP, the distinct models that predict lipophilicity. Also, Log of Consensus Po/w is calculated by taking their arithmetic mean [43]. BOILED Egg is a map of polarity expressed in TPSA, another model for predicting lipophilicity. The yolk reflects the potential for BBB permeability in the BOILED Egg plot (Fig. 10), whereas the white represents the possibility for GI absorption. Finally, a bioavailability radar map is a plot of six different physicochemical parameters: size, polarity, flexibility, solubility, saturation, and lipophilicity. The pink hexagon in the center of the figure represents the optimal range for excellent oral bioavailability (Fig. 11) [44, 45].

**PKCSM** affords important information about pharmacokinetic parameters of the drug such as: Caco-2 permeability, volume of distribution at steady state (VDss), Pgp I and II inhibitors, total clearance, central nervous system (CNS) permeability, AMES toxicity, renal organic cation transporter 2 (OCT2) substrate, maximum recommended tolerated dose (MRTD) human, oral rat acute toxicity (LD_50_) and chronic toxicity-lowest observed adverse effect (LOAEL), skin sensitization, hepatotoxicity, Tetrahymena pyriformis toxicity, and LC_50_ by fathead minnow toxicity [46].

SwissADME results of compounds **7k**, **7l**, **7m**, and Erlotinib are presented in Table 8. These results indicated that compounds **7k** and **7m** obeyed Lipinski rule with one violation (M.wt > 500) while **7l** did not; with two violations: M,wt > 500 and MLOGP of 4.17 (> 4.15). Compound **7k** calculated octanol/water partition coefficient (log P) is 3.28 which is equal to Erlotinib while the log P values for **7l** and **7m** were 5.87 and 5.41, respectively. The three compounds showed low molecular flexibility (rotatable bond (RB) > 10) which may indicate poor absorption of the compounds.

All three compounds showed promising cell permeability through diverse biological membrane as topological polar surface area (TPSA) results ranged from 148.30 to 157.53 while Erlotinib was 74.73.

**Table 8:** ADMET Results of the test with SwissADME for the target compounds **7k, 7l, 7m,** and Erlotinib

Compound	M.Wt	Fraction Csp3	RB	HBA	HBD	MR	TPSA	Log P	GI absorption	BBB perm.	Lip. V.	Bio. Sc.
**7k**	587.65	0.06	11	7	2	166.67	148.30	3.28	Low	No	Yes	0.55
**7l**	622.09	0.06	11	7	2	171.68	148.30	5.87	Low	No	No	0.17
**7m**	617.67	0.09	12	8	2	173.17	157.53	5.41	Low	No	Yes	0.55
**Erlotinib**	393.44	0.27	10	6	1	111.40	74.73	3.28	High	Yes	Yes	0.55

PKCSM results were slightly different from SwissADME results. The difference in prediction outcomes between web servers are most likely due to changes in the modules and algorithms utilized on each web server. As a result, it is critical to receive ADMET characteristics from many web servers rather than just one [47].

The results shown in Table 9, revealed that the compounds **7k**, **7l**, and **7m** displayed good values for oral absorption. Erlotinib was predicted to have a cellular permeability of 97.99% while compounds **7k**, **7l**, and **7m** values ranged from 93.26 to 98.11%. The three compounds showed higher water solubility equal to -2.89 log mol/L than Erlotinib − 5.11 log mol/L. and displayed good skin permeability but low permeability in Caco2 (human colon adenocarcinoma). It was also predicted that **7k**, **7l**, and **7m** may be a substrate of the ATP-binding cassette transporter P-glycoprotein, which was found to have an inhibitory effect in epithelial cells.

Concerning distribution, the compounds showed low volume of distribution at a steady state at a value of -0.011, -0.009 and-0.038 log L/kg which was comparable to Erlotinib value of 0.167 log L/kg. Distribution parameter revealed that the compounds were poorly distributed to blood brain barrier and displayed safety in CNS. It was predicted that the three compounds have no or little drug-drug interactions as they were shown to be CYP3A4 but not CYP2D6 substrates, and CYP2C19, CYP2C9, and CYP3A4 but not CYP2D6 inhibitors.

According to excretion parameters all compounds showed better results for total clearance from liver and kidney with values of 0.678, 0.669 and 0.703 log ml/min/kg compared to Erlotinib with 0.572 log ml/min/kg value.

Furthermore, the software predicted toxicity profiles including mutagenicity and cardiotoxicity. The three compounds and Erlotinib showed similar safety profile and acceptable level profile hERG I (human ether-a-go-go-related gene I) but displayed hepatotoxicity. Based on the in-silico ADME/Tox prediction, we may infer that compounds **7k, 7l,** and **7m** may possess an acceptable pharmacokinetic and safety profile, validating its promise as an oral therapy for cancer. Complete ADMET results of testing with PKCSM are presented in Table 9.

**Table 9:** ADMET Results of the test with PKCSM for the target compounds **7k, 7l, 7m** and **Erlotinib**

Properties	7k	7l	7m	Erlotinib
**LOGP**	6.9154	7.5688	6.924	3.4051
**Surface area**	250.582	260.885	262.060	169.532
**Water Solubility**	-2.894	-2.894	-2.894	-5.114
**Caco2 Permeability**	0.133	0.026	0.114	0.877
**Intestinal absorption**	96.941	98.107	93.255	97.995
**Skin permeability**	-2.735	-2.735	-2.735	-2.802
**P-glycoprotein (Pgp) substrate**	Yes	Yes	Yes	No
**Pgp1 inhibitor**	Yes	Yes	Yes	Yes
**Pgp2 inhibitor**	Yes	Yes	Yes	Yes
**VDss**	-0.011	-0.009	-0.038	0.167
**BBB permeability**	-1.353	-1.534	-1.563	-0.604
**CNS Permeability**	-2.881	-2.824	-3.043	-3.531
**CYP2D6 substrate**	No	No	No	No
**CYP3A4 substrate**	Yes	Yes	Yes	Yes
**CYP1A2 inhibitor**	Yes	Yes	Yes	No
**CYP2C19 inhibitor**	Yes	Yes	Yes	Yes
**CYP2C9 inhibitor**	Yes	Yes	Yes	Yes
**CYP2D6 inhibitor**	No	No	No	No
**CYP3A4 inhibitor**	Yes	Yes	Yes	Yes
**Total clearance**	0.678	0.669	0.703	0.572
**Renal OCT2 substrate**	Yes	Yes	No	No
**AMES toxicity**	Yes	Yes	Yes	No
**Max tolerated dose MRTDs log**	0.339	0.324	0.315	0.375
**hERG I inhibitor**	No	No	No	No
**hERG II inhibitor**	Yes	Yes	Yes	Yes
**Oral Rat Acute Toxicity LD**_**50**_	2.479	2.479	2.479	3.155
**LOAEL**	2.165	2.095	1.895	1.477
**Hepatotoxicity**	Yes	Yes	Yes	Yes
***T. Pyriformis*** **toxicity**	0.285	0.285	0.285	0.356
**Minnow toxicity**	-1.122	-1.579	-1.875	-2.252

## Conclusion

In this study we explored the apoptotic activity of new chemical architectures comprised of substituted benzimidazole moiety directly linked to an oxadiazole ring which is tethered to a chalcone derivative. The new hybrids’ NCI assay results revealed that they had remarkable anti-proliferative activities, principally hybrids **7e**, **7k**, and **7m-o**. Furthermore, in vitro anti-proliferative assays revealed promising inhibitory activity against a panel of four cancer cell lines, with IC_50_ values comparable to Doxorubicin. Compounds **7k**, **7l**, and **7m** displayed promising activity in activation of caspases, inhibiting EGFR and/or BRAF^V600E^, induction of BAX and inhibition of Bcl-2 proteins expression. Further docking, MD simulations, and Δ*G*_Bind_ experiments were conducted to study the binding modes and stability of **7k**, **7l**, and **7m** inside the kinase domains of both EGFR and BRAF. These results indicated that these compounds could be used as potential apoptotic anti-proliferative agents upon further optimization. ADMET properties of compounds **7k**, **7l**, and **7m** were predicted successfully by two web servers Swiss ADME and PKCSM. The results revealed that the compounds had an ADMET profile that is comparatively similar to Erlotinib.

## Experimental section

### Chemistry

General Details: See Appendix A.

#### General procedure for synthesis of 2-{5-[2-aryl-1 * H*-benzo[*d*]imidazol-5-yl]-1,3,4-oxadiazol-2-ylthio}-*N*-{4-[(*E*)-3-arylacryloyl]phenyl}acetamide 7a-x [25, 30]

TEA (0.18 mL/mol) was added as a base to an equimolar mixture of the proper 2-bromo-*N*-{4-[(*E*)-3-arylacryloyl]phenyl}acetamides **6a-e** (0.09 mmol) and the proper oxadiazoles **4a-e** (0.09 mmol) in acetonitrile. The reaction mixture was left to stir from 48 to 72 h at room temperature till the formation of the precipitate. The target hybrids were obtained in a good yield by filtration, drying, and recrystallization of the formed precipitate from acetonitrile.

##### 2-{5-[2-Phenyl-1* H*-benzo[*d*]imidazol-5-yl]-1,3,4-oxadiazol-2-ylthio}-*N*-{4-[(*E*)-3-phenylacryloyl]phenyl}acetamide (7a)

Off-white crystal (0.21 g, 80% yield); m.p: 273–275 °C; ^1^ H NMR (500 MHz, DMSO-*d*_6_) δ (ppm): 4.44 (2 H, s, SCH_2_), 7.46 (4 H, d, *J* = 5.4 Hz, Ar-H), 7.52–7.60 (3 H, m, Ar-H), 7.74 (1 H, d, *J* = 15.6 Hz, CH = CH), 7.82 (3 H, d, *J* = 8.4 Hz, Ar-H), 7.86–7.90 (2 H, m, Ar-H), 7.95 (1 H, d, *J* = 15.6 Hz, CH = CH), 8.19–8.25 (5 H, m, Ar-H), 10.86 (1 H, s, NH), 13.31 (1 H, s, NH); ^13^ C NMR (125 MHz, DMSO-*d*_6_) δ (ppm): 46.16, 112.88, 117.44, 117.66, 118.79, 120.29, 121.43, 122.44, 126.95, 127.35, 129.51, 129.73, 129.98, 130.48, 130.86, 133.25, 134.99, 143.44, 144.07, 153.85, 153.97, 154.32, 163.15, 165.57, 166.45, 188.32; HRMS (ESI) m/z [M + H]^+^ calcd for C_32_H_23_N_5_O_3_S: 558.1594, found: 558.1536.

##### 2-{5-[2-Phenyl-1* H*-benzo[*d*]imidazol-5-yl]-1,3,4-oxadiazol-2-ylthio}-N-{4-[(*E*)-3-(4-chlorophenyl)acryloyl]phenyl}acetamide (7b)

Off-white crystal (0.19 g, 80% yield); m.p: 263–264 °C; ^1^ H NMR (500 MHz, DMSO-*d*_6_) δ (ppm): 4.43 (2 H, s, SCH_2_), 7.52 (3 H, d, *J* = 8.2 Hz, Ar-H), 7.53–7.60 (3 H, m, Ar-H), 7.71 (1 H, d, *J* = 15.4 Hz, CH = CH), 7.81 (2 H, d, *J* = 8.5 Hz, Ar-*H*), 7.92 (2 H, d, *J* = 8.5 Hz, Ar-H), 7.97 (1 H, d, *J* = 15.4 Hz, CH = CH), 8.16–8.24 (6 H, m, Ar-H), 10.86 (1 H, s, NH), 13.32 (1 H, s, NH); ^13^ C NMR (125 MHz, DMSO-*d*_6_) δ (ppm): 47.58, 113.59, 117.84, 119.55, 121.26, 123.20, 127.52, 128.96, 129.47, 129.76, 130.38, 130.89, 131.48, 133.20, 134.04, 134.31, 135.14, 137.74, 138.06, 142.82, 144.02, 151.09, 153.12, 165.87, 166.46, 188.31; HRMS (ESI) m/z [M + H]^+^ calcd for C_32_H_22_ClN_5_O_3_S: 592.1205, found: 592.1160.

##### 2-{5-[2-Phenyl-1* H*-benzo[*d*]imidazol-5-yl]-1,3,4-oxadiazol-2-ylthio}-*N*-{4-[(*E*)-3-(4-methoxyphenyl)acryloyl]phenyl}acetamide (7c)

Beige crystal (0.19 g, 79% yield); m.p: 175–177 °C; ^1^ H NMR (500 MHz, DMSO-*d*_6_) δ (ppm): 3.83 (3 H, s, OCH_3_), 4.26 (2 H, s, SCH_2_), 6.92–7.14 (3 H, m, Ar-H), 7.54–7.65 (5 H, m, 4 Ar-H + CH = CH), 7.73 (1 H, s, Ar-H), 7.80–7.90 (5 H, m, Ar-H), 8.16 (1 H, d, *J* = 15.4 Hz, CH = CH), 8.23–8.39 (3 H, m, Ar-H), 10.91 (1 H, s, NH), 13.34 (1 H, s, NH); ^13^ C NMR (125 MHz, DMSO-*d*_6_) δ (ppm): 43.63, 55.84, 114.86, 114.89, 114.91, 119.87, 119.88, 119.94, 127.06, 127.12, 127.24, 129.01, 129.03, 129.49, 129.51, 129.53, 129.62, 131.16, 131.38, 138.12, 139.15, 144.01, 145.35, 153.41, 161.97, 163.32, 187.81; HRMS (ESI) m/z [M + H]^+^ calcd for C_33_H_25_N_5_O_4_S: 588.1700, found: 588.1620.

##### 2-{5-[2-Phenyl-1* H*-benzo[*d*]imidazol-5-yl]-1,3,4-oxadiazol-2-ylthio}-*N*-{4-[(*E*)-3-(3,4-dimethoxyphenyl)acryloyl]phenyl}acetamide (7d)

Dark brown (0.20 g, 83% yield); m.p: 156–158 °C; ^1^ H NMR (500 MHz, DMSO-*d*_*6*_) δ (ppm): 3.82 (3 H, s, OCH_3_), 3.87 (3 H, s, OCH_3_), 4.45 (2 H, s, SCH_2_), 7.02 (1 H, d, *J* = 8.4 Hz, Ar-H), 7.38 (1 H, d, *J* = 8.4 Hz, Ar-H), 7.54 (1 H, s, Ar-H), 7.56–7.60 (3 H, m, 2 Ar-H + CH = CH), 7.69 (1 H, d, *J* = 15.4 Hz, CH = CH), 7.76 (1 H, d, *J* = 8.4 Hz, Ar-H), 7.84 (3 H, d, *J* = 8.4 Hz, Ar-H), 7.86 (1 H, s, Ar-H), 7.89–7.96 (1 H, m, Ar-H), 8.20 (2 H, d, *J* = 8.0 Hz, Ar-H), 8.24 (2 H, d, *J* = 8.0 Hz, Ar-H), 11.01 (1 H, s, NH), ^13^ C NMR (125 MHz, DMSO-*d*_*6*_) δ (ppm): 45.89, 56.06, 56.22, 111.12, 111.95, 118.87, 119.13, 119.93, 124.28, 125.29, 127.20, 127.97, 129.29, 129.49, 129.88, 129.95, 130.22, 130.77, 133.42, 134.03, 143.20, 143.45, 144.37, 149.52, 151.70, 154.21, 163.06, 166.18, 166.55, 187.79; HRMS (ESI) m/z [M + K]^+^ calcd for C_34_H_27_N_5_O_5_S: 656.1364, found 656.1407.

##### 2-{5-[2-Phenyl-1* H*-benzo[*d*]imidazol-5-yl]-1,3,4-oxadiazol-2-ylthio}-*N*-{4-[(*E*)-3-(3,4,5-trimethoxyphenyl)acryloyl]phenyl}acetamide (7e)

Dark brown (0.21 g, 85% yield); m.p: 143–144 °C; ^1^ H NMR (500 MHz, DMSO-*d*_*6*_) δ (ppm): 3.73 (3 H, s, OCH_3_), 3.88 (6 H, s, 2OCH_3_), 4.26 (1.20 H, s, SCH_2_), 4.45 (0.80 H, s, SCH_2_), 7.24 (2 H, s, Ar-H), 7.54 (1 H, d, *J* = 7.1 Hz, Ar-H), 7.57–7.60 (4 H, m, Ar-H), 7.70–7.74 (2 H, m, 1 Ar-H + CH = CH), 7.84 (1 H, d, *J* = 8.5 Hz, Ar-H), 7.94 (1 H, d, *J* = 15.3 Hz, CH = CH), 8.18 (1 H, d, *J* = 8.5 Hz, Ar-H), 8.23 (3 H, d, *J* = 8.5 Hz, Ar-H), 8.32 (1 H, d, *J* = 8.5 Hz, Ar-H), 10.89 (1 H, s, NH), 13.30 (1 H, s, NH); ^13^ C NMR (125 MHz, DMSO-*d*_*6*_) δ (ppm): 45.99, 56.62, 60.62, 106.92, 106.95, 107.12, 118.8, 121.49, 126.56, 127.12, 127.24, 127.26, 129.01, 129.03, 129.48, 129.72, 130.17, 130.47, 130.75, 139.20, 139.63, 140.36, 144.45, 145.40, 153.56, 162.28, 168.69, 186.96; HRMS (ESI) m/z [M + H]^+^ calcd for C_35_H_29_N_5_O_6_S: 648.1911, found: 648.1963.

##### 2-{5-[2-(4-Chlorophenyl)-1* H*-benzo[*d*]imidazol-5-yl]-1,3,4-oxadiazol-2-ylthio}-*N*-{4-[(*E*)-3-phenylacryloyl]phenyl}acetamide (7f)

Beige crystal (0.21 g, 84.5% yield); m.p: 161–163 °C; ^1^ H NMR (500 MHz, DMSO-*d*_*6*_) δ (ppm): 4.45 (2 H, s, SCH_2_), 7.40–7.50 (4 H, m, Ar-H), 7.63 (2 H, d, *J* = 8.3 Hz, Ar-H), 7.73 (1 H, d, *J* = 15.6 Hz, CH = CH), 7.84 (3 H, d, *J* = 8.3 Hz, Ar-H), 7.88 (3 H, d, *J* = 8.3 Hz, Ar-H), 7.94 (1 H, d, *J* = 15.6 Hz, CH = CH), 8.19 (2 H, d, *J* = 8.6 Hz, Ar-H), 8.24 (2 H, d, *J* = 8.6 Hz, Ar-H), 11.03 (1 H, s, NH); ^13^ C NMR (125 MHz, DMSO-*d*_*6*_) δ (ppm): 45.88, 117.50, 119.03, 122.39, 128.81, 128.98, 129.10, 129.26, 129.35, 129.59, 130.36, 130.45, 130.96, 133.11, 135.20, 135.67, 136.25, 143.42, 143.65, 143.92, 149.29, 155.80, 162.98, 166.37, 166.46, 188.02; HRMS (ESI) m/z [M + H]^+^ calcd for C_32_H_22_ClN_5_O_3_S: 592.1205, found: 592.1152.

##### 2-{5-[2-(4-Chlorophenyl)-1* H*-benzo[*d*]imidazol-5-yl]-1,3,4-oxadiazol-2-ylthio}-*N*-{4-[(*E*)-3-(4-chlorophenyl)acryloyl]phenyl}acetamide (7g)

Pale brown powder (0.19 g, 79.5% yield); m.p: 215–217 °C; ^1^ H NMR (400 MHz, DMSO-*d*_*6*_) δ (ppm): 4.44 (2 H, s, SCH_2_), 7.19–7.24 (1 H, m, Ar-H), 7.50 (2 H, d, *J* = 8.1 Hz, Ar-H), 7.57–7.61 (1 H, m, Ar-H), 7.69 (1 H, d, *J* = 15.5 Hz, CH = CH), 7.79 (2 H, d, *J* = 8.3 Hz, Ar-H), 7.88–7.95 (3 H, m, 2 Ar-H + CH = CH), 7.97 (1 H, s, Ar-H), 8.11 (2 H, d, *J* = 8.0 Hz, Ar-H), 8.18 (2 H, d, *J* = 8.3 Hz, Ar-H), 8.33 (2 H, d, *J* = 8.0 Hz, Ar-H), 10.89 (1 H, s, NH), 13.35 (1 H, s, NH); ^13^ C NMR (125 MHz, DMSO-*d*_*6*_) δ (ppm): 46.10, 111.76, 113.41, 118.96, 119.08, 120.91, 123.15, 124.44, 127.47, 127.83, 128.75, 129.40, 130.54, 130.98, 131.37, 133.08, 134.20, 135.45, 142.47, 143.56, 145.13, 151.96, 164.14, 165.29, 166.11, 187.92; Anal. calcd. for C_32_H_21_Cl_2_N_5_O_3_S: C 61.35, H 3.38, N 11.18, S 5.12. Found: C 61.62, H 3.52, N 11.40, S 5.29.

##### 2-{5-[2-(4-Chlorophenyl)-1* H*-benzo[*d*]imidazol-5-yl]-1,3,4-oxadiazol-2-ylthio}-*N*-{4-[(*E*)-3-(4-methoxyphenyl)acryloyl]phenyl}acetamide (7h)

Beige crystal (0.19 g, 82% yield); m.p: 228–230 °C; ^1^ H NMR (400 MHz, DMSO-*d*_*6*_) δ (ppm): 3.81 (3 H, s, OCH_3_), 4.26 (0.60 H, s, SCH_2_), 4.46 (1.40 H, s, SCH_2_), 7.01 (2 H, d, *J* = 8.8 Hz, Ar-H), 7.22–7.24 (2 H, m, 1 Ar-H + CH = CH), 7.60–7.66 (2 H, m, Ar-H), 7.69 (1 H, d, *J* = 15.5 Hz, CH = CH), 7.78–7.84 (4 H, m, Ar-H), 8.12–8.17 (4 H, m, Ar-H), 8.37–8.41 (2 H, m, Ar-H), 11.00 (1 H, s, NH); ^13^ C NMR (125 MHz, DMSO-*d*_*6*_) δ (ppm): 45.51, 55.42, 113.69, 114.44, 118.66, 119.46, 122.53, 122.59, 123.68, 127.03, 127.12, 127.27, 127.42, 129.89, 130.76, 133.02, 133.15, 142.90, 143.54, 149.98, 161.33, 163.66, 164.84, 164.92, 165.68, 168.97, 187.53; Anal. calcd. for C_33_H_24_ClN_5_O_4_S: C 63.71, H 3.89, N 11.26, S 5.15. Found: C 63.98, H 4.05, N 11.51, S 5.23.

##### 2-{5-[2-(4-Chlorophenyl)-1* H*-benzo[*d*]imidazol-5-yl]-1,3,4-oxadiazol-2-ylthio}-*N*-{4-[(*E*)-3-(3,4-dimethoxyphenyl)acryloyl]phenyl}acetamide (7i)

Pale brown powder (0.23 g, 83% yield); m.p: 208–210 °C; ^1^ H NMR (400 MHz, DMSO-*d*_*6*_) δ (ppm): 3.81 (3 H, s, OCH_3_), 3.86 (3 H, s, OCH_3_), 4.45 (2 H, s, SCH_2_), 7.01 (1 H, d, *J* = 8.3 Hz, Ar-H), 7.25 (1 H, d, *J* = 8.3 Hz, Ar-H), 7.36 (1 H, d, *J* = 8.3 Hz, Ar-H), 7.52 (1 H, s, Ar-H), 7.72–7.78 (2 H, m, 1 Ar-H + CH = CH), 7.80–7.84 (4 H, m, 3 Ar-H + CH = CH), 8.13 (2 H, d, *J* = 8.5 Hz, Ar-H), 8.18 (2 H, d, *J* = 8.5 Hz, Ar-H), 8.36 (2 H, d, *J* = 8.3 Hz, Ar-H), 10.96 (1 H, s, NH), 13.48 (1 H, s, NH); ^13^ C NMR (125 MHz, DMSO-*d*_*6*_) δ (ppm): 46.02, 56.08, 56.25, 111.25, 112.06, 113.58, 119.08, 119.99, 124.31, 124.36, 124.43, 124.47, 127.47, 127.78, 127.85, 128.08, 130.27, 130.33, 133.08, 133.12, 133.50, 143.31, 144.42, 144.47, 149.52, 151.71, 164.14, 165.30, 166.07, 187.95; Anal. calcd. for C_34_H_26_ClN_5_O_5_S: C 62.62, H 4.02, N 10.74, S 4.92. Found: C 62.84, H 4.19, N 11.03, S 5.01.

##### 2-{5-[2-(4-Chlorophenyl)-1* H*-benzo[*d*]imidazol-5-yl]-1,3,4-oxadiazol-2-ylthio}-*N*-{4-[(*E*)-3-(3,4,5-trimethoxyphenyl)acryloyl]phenyl}acetamide (7j)

Beige powder (0.20 g, 82% yield); m.p: 223–225 °C; ^1^ H NMR (400 MHz, DMSO-*d*_*6*_) δ (ppm): 3.71 (3 H, s, OCH_3_), 3.86 (6 H, s, OCH_3_), 4.32 (0.40 H, s, SCH_2_), 4.45 (1.60 H, s, SCH_2_), 7.16–7.28 (4 H, m, Ar-H), 7.56–7.62 (1 H, m, Ar-H), 7.67 (1 H, d, *J* = 15.5 Hz, CH = CH), 7.81 (2 H, d, *J* = 8.8 Hz, Ar-H), 7.89 (1 H, d, *J* = 15.5 Hz, CH = CH), 8.14 (2 H, d, *J* = 8.4 Hz, Ar-H), 8.19 (2 H, d, *J* = 8.8 Hz, Ar-H), 8.35 (2 H, d, *J* = 8.4 Hz, Ar-H), 10.89 (1 H, s, NH), 13.39 (1 H, s, NH); ^13^ C NMR (125 MHz, DMSO-*d*_*6*_) δ (ppm): 46.18, 56.63, 60.62, 107.00, 113.44, 119.09, 121.60, 122.96, 124.46, 126.91, 127.49, 127.78, 127.85, 130.42, 130.46, 130.77, 133.11, 133.33, 140.22, 143.41, 143.47, 144.48, 153.59, 164.16, 165.31, 166.08, 166.32, 188.02; Anal. calcd. for C_35_H_28_ClN_5_O_6_S: C 61.63, H 4.14, N 10.27, S 4.70. Found: C 61.83, H 4.32, N 10.53, S 4.83.

##### 2-{5-[2-(4-Methoxyphenyl)-1* H*-benzo[*d*]imidazol-5-yl]-1,3,4-oxadiazol-2-ylthio}-*N*-{4-[(*E*)-3-phenylacryloyl]phenyl}acetamide (7k)

Orange crystal (0.11 g, 74% yield); m.p: 178–180 °C; ^1^ H NMR (500 MHz, DMSO-*d*_*6*_) δ (ppm): 3.86 (3 H, s, OCH_3_), 4.34 (2 H, s, SCH_2_), 7.14 (2 H, d, *J* = 8.3 Hz, Ar-H), 7.37–7.46 (5 H, m, Ar-H), 7.73 (1 H, d, *J* = 15.4 Hz, CH = CH), 7.78 (2 H, d, *J* = 8.3 Hz, Ar-H), 7.83–7.89 (3 H, m, Ar-H), 7.95 (1 H, d, *J* = 15.4 Hz, CH = CH), 8.15–8.21 (4 H, m, Ar-H), 10.79 (1 H, s, NH), 13.10 (1 H, s, NH); ^13^ C NMR (125 MHz, DMSO-*d*_*6*_) δ (ppm): 46.12, 55.85, 114.93, 114.95, 119.05, 119.09, 121.71, 122.42, 128.73, 128.76, 128.84, 129.27, 129.33, 129.36, 129.76, 130.44, 130.48, 130.97, 131.00, 133.08, 133.10, 135.22, 143.53, 143.94, 165.32, 166.32, 188.03; HRMS (ESI) m/z [M + H]^+^ calcd. for C_33_H_25_N_5_O_4_S: 588.1700, found: 588.1643.

##### 2-{5-[2-(4-Methoxyphenyl)-1* H*-benzo[*d*]imidazol-5-yl]-1,3,4-oxadiazol-2-ylthio}-*N*-{4-[(*E*)-3-(4-chlorophenyl)acryloyl]phenyl}acetamide (7l)

Pale brown crystal (0.23 g, 80% yield); m.p: 245–247 °C; ^1^ H NMR (500 MHz, DMSO-*d*_*6*_) δ (ppm): 3.89 (3 H, s, OCH_3_), 4.26 (1 H, s, SCH_2_), 4.35 (1 H, s, SCH_2_), 7.17–7.24 (2 H, m, Ar-H), 7.51 (2 H, d, *J* = 8.2 Hz, Ar-H), 7.70 (1 H, d, *J* = 15.6 Hz, CH = CH), 7.76–7.81 (2 H, m, 1 Ar-H + CH = CH), 7.83 (1 H, d, *J* = 8.2 Hz, Ar-H), 7.92 (2 H, d, *J* = 8.4 Hz, Ar-H), 7.98 (1 H, d, *J* = 8.4 Hz, Ar-H), 8.15–8.19 (3 H, m, Ar-H), 8.28 (2 H, m, Ar-H), 8.34 (1 H, d, *J* = 8.4 Hz, Ar-H), 10.98 (1 H, s, NH); ^13^ C NMR (125 MHz, DMSO-*d*_*6*_) δ (ppm): 45.86, 55.86, 114.37, 115.00, 115.52, 119.04, 119.26, 123.14, 125.22, 125.76, 126.34, 129.37, 129.81, 130.17, 130.44, 130.48, 130.59, 130.94, 132.98, 134.19, 135.41, 143.12, 143.64, 166.17, 166.46, 167.29, 187.81; Anal. calcd. for C_33_H_24_ClN_5_O_4_S: C 63.71, H 3.89, N 11.26, S 5.15. Found: C 63.52, H 4.03, N 11.52, S 5.23.

##### 2-{5-[2-(4-Methoxyphenyl)-1* H*-benzo[*d*]imidazol-5-yl]-1,3,4-oxadiazol-2-ylthio}-*N*-{4-[(*E*)-3-(4-methoxyphenyl)acryloyl]phenyl}acetamide (7m)

Beige Crystal (0.13 g, 81% yield); m.p: 247–249 °C; ^1^ H NMR (500 MHz, DMSO-*d*_*6*_) δ (ppm): 3.82 (3 H, s, OCH_3_), 3.85 (H, s, OCH_3_), 4.25 (1.25 H, s, SCH_2_), 4.43 (0.75 H, s, SCH_2_), 6.95–7.03 (2 H, m, Ar-H), 7.12–7.15 (3 H, m, Ar-H), 7.71 (1 H, d, *J* = 15.3 Hz, CH = CH), 7.80–7.85 (5 H, m, Ar-H + CH = CH), 8.09–8.20 (6 H, m, Ar-H), 10.90 (1 H, s, NH), 13.32 (1 H, s, NH); ^13^ C NMR (125 MHz, DMSO-*d*_*6*_) δ (ppm): 46.05, 55.84, 55.85, 114.84, 114.94, 116.83, 116.86, 119.06, 119.86, 120.78, 122.38, 127.85, 128.83, 128.90, 130.30, 131.15, 133.27, 133.43, 143.20, 144.01, 154.06, 154.36, 161.55, 162.94, 166.00, 166.51, 169.50, 187.80; HRMS (ESI) m/z [M + H]^+^ calcd. for C_34_H_27_N_5_O_5_S: 618.1806, found: 618.1737.

##### 2-{5-[2-(4-Methoxyphenyl)-1* H*-benzo[*d*]imidazol-5-yl]-1,3,4-oxadiazol-2-ylthio}-*N*-{4-[(*E*)-3-(3,4-dimethoxyphenyl)acryloyl]phenyl}acetamide (7n)

Pale brown crystal (0.19 g, 78% yield); m.p: 222–224 °C; ^1^ H NMR (500 MHz, DMSO-*d*_*6*_) δ (ppm): 3.81 (3 H, s, OCH_3_), 3.87 (6 H, s, 2OCH_3_), 4.35 (0.87 H, s, SCH_2_), 4.45 (1.13 H, s, SCH_2_), 7.00 (1 H, d, *J* = 8.6 Hz, Ar-H), 7.18 (1 H, d, *J* = 8.4 Hz, Ar-H), 7.23 (1 H, d, *J* = 8.4 Hz, Ar-H), 7.36 (1 H, d, *J* = 8.6 Hz, Ar-H), 7.53 (1 H, s, Ar-H), 7.67 (1 H, d, *J* = 15.6 Hz, CH = CH), 7.69–7.85 (4 H, m, Ar-H + CH = CH), 7.91 (1 H, d, *J* = 8.6 Hz, Ar-H), 8.00 (1 H, d, *J* = 8.6 Hz, Ar-H), 8.15–8.19 (2 H, m, Ar-H), 8.25 (1 H, s, Ar-H), 8.30 (1 H, d, *J* = 8.8 Hz, Ar-H), 11.00 (1 H, s, NH), 13.30 (1 H, s, NH); ^13^ C NMR (125 MHz, DMSO-*d*_*6*_) δ (ppm): 44.37, 56.05, 56.16, 56.21, 110.97, 112.10, 114.18, 115.29, 115.51, 119.02, 119.32, 119.86, 124.40, 125.73, 127.90, 129.35, 129.81, 130.17, 130.29, 130.32, 133.56, 142.97, 144.31, 149.37, 151.24, 151.76, 152.90, 162.28, 163.10, 167.65, 187.50; Anal. calcd. for C_35_H_29_N_5_O_6_S: C 64.90, H 4.51, N 10.81, S 4.95. Found: C 65.07, H 4.69, N 11.05, S 5.01.

##### 2-{5-[2-(4-Methoxyphenyl)-1* H*-benzo[*d*]imidazol-5-yl]-1,3,4-oxadiazol-2-ylthio}-*N*-{4-[(*E*)-3-(3,4,5-trimethoxyphenyl)acryloyl]phenyl}acetamide (7o)

Pale brown Crystal (0.21 g, 86% yield); m.p: 154–156 °C; ^1^ H NMR (500 MHz, DMSO-*d*_*6*_) δ (ppm): 3.71 (3 H, s, OCH_3_), 3.84 (3 H, s, OCH_3_), 3.86 (6 H, s, 2OCH_3_), 4.39 (0.30 H, s, SCH_2_), 4.46 (1.70 H, s, SCH_2_), 7.15 (2 H, d, *J* = 8.8 Hz, Ar-H), 7.23 (2 H, s, Ar-H), 7.67 (1 H, d, *J* = 8.0 Hz, Ar-H), 7.71 (1 H, d, *J* = 15.5 Hz, CH = CH), 7.86 (2 H, d, *J* = 8.8 Hz, Ar-H), 7.91 (1 H, d, *J* = 15.5 Hz, CH = CH), 8.20 (3 H, d, *J* = 8.5 Hz, Ar-H), 8.25 (3 H, d, *J* = 8.5 Hz, Ar-H), 11.16 (1 H, s, NH), 13.38 (1 H, s, NH); ^13^ C NMR (125 MHz, DMSO-*d*_*6*_) δ (ppm): 44.32, 55.94, 56.63, 57.81, 106.96, 114.81, 114.96, 114.99, 115.02, 116.87, 119.03, 119.06, 121.72, 129.15, 129.22, 129.25, 130.43, 130.74, 132.98, 140.00, 140.15, 143.48, 144.31, 153.52, 153.55, 161.76, 166.02, 168.18, 188.00; HRMS (ESI) m/z [M + H]^+^ calcd. for C_36_H_31_N_5_O_7_S: 678.2017, found: 678.2090.

##### 2-{5-[2-(3,4-Dimethoxyphenyl)-1* H*-benzo[*d*]imidazol-5-yl]-1,3,4-oxadiazol-2-ylthio}-*N*-{4-[(*E*)-3-phenylacryloyl]phenyl}acetamide (7p)

Orange powder (0.18 g, 89.32% yield); m.p: 185–186 °C; ^1^ H NMR (500 MHz, DMSO-*d*_*6*_) δ (ppm): 3.85 (3 H, s, OCH_3_), 3.90 (3 H, s, OCH_3_), 4.26 (0.60 H, s, SCH_2_), 4.43 (1.40 H, s, SCH_2_), 7.16 (1 H, s, Ar-H), 7.40–7.55 (3 H, m, Ar-H), 7.65 (1 H, s, Ar-H), 7.74 (1 H, d, *J* = 15.4 Hz, CH = CH), 7.77–7.83 (5 H, m, Ar-H), 7.88–7.97 (3 H, m, 2 Ar-H + CH = CH), 8.08–8.20 (2 H, m, Ar-H), 8.26–8.28 (1 H, m, Ar-H), 10.88 (1 H, s, NH), 13.31(1 H, s, NH); ^13^ C NMR (125 MHz, DMSO-*d*_*6*_) δ (ppm): 46.01, 56.10, 56.23, 110.41, 112.31, 112.32, 116.86, 117.16, 119.09, 120.16, 122.22, 122.36, 129.27, 129.32, 129.36, 129.40, 129.77, 130.48, 131.10, 131.40, 133.27, 135.13, 137.82, 143.49, 143.95, 149.41, 151.29, 163.10, 166.29, 188.03; HRMS (ESI) m/z [M + H]^+^ calcd. for C_34_H_27_N_5_O_5_S: 618.1806, found: 618.1865.

##### 2-{5-[2-(3,4-Dimethoxyphenyl)-1* H*-benzo[*d*]imidazol-5-yl]-1,3,4-oxadiazol-2-ylthio}-*N*-{4-[(*E*)-3-(4-chlorophenyl)acryloyl]phenyl}acetamide (7q)

Brown powder (0.12 g, 84.62% yield); m.p: 194–195 °C; ^1^ H NMR (500 MHz, DMSO-*d*_*6*_) δ (ppm): 3.85 (3 H, s, OCH_3_), 3.91 (3 H, s, OCH_3_), 4.14 (1.20 H, s, SCH_2_), 4.36 (0.80 H, s, SCH_2_), 7.15 (1 H, d, *J* = 6.6 Hz, Ar-H), 7.52 (2 H, d, *J* = 8.8 Hz, Ar-H), 7.71 (1 H, d, *J* = 15.2 Hz, CH = CH), 7.75 (1 H, s, Ar-H), 7.80 (2 H, d, *J* = 8.0 Hz, Ar-H), 7.84 (3 H, d, *J* = 8.8 Hz, Ar-H), 7.92 (2 H, d, *J* = 8.0 Hz, Ar-H), 7.97 (1 H, d, *J* = 15.2 Hz, CH = CH)), 8.12 (1 H, s, Ar-H), 8.15–8.22 (2 H, d, *J* = 8.8 Hz, Ar-H), 10.98 (1 H, s, NH), 13.28 (1 H, s, NH); ^13^ C NMR (125 MHz, DMSO-*d*_*6*_) δ (ppm): 45.80, 56.10, 56.15, 110.49, 112.29, 116.98, 119.01, 119.10, 120.34, 120.36, 122.49, 123.16, 129.31, 129.38, 130.45, 130.49, 130.96, 132.95, 133.01, 134.20, 134.59, 135.42, 142.42, 143.63, 143.66, 149.39, 151.24, 162.00, 166.53, 187.79; HRMS (ESI) m/z [M + H]^+^ calcd. for C_34_H_26_ClN_5_O_5_S: 652.1416, found: 652.1367.

##### 2-{5-[2-(3,4-Dimethoxyphenyl)-1* H*-benzo[*d*]imidazol-5-yl]-1,3,4-oxadiazol-2-ylthio}-*N*-{4-[(*E*)-3-(4-methoxyphenyl)acryloyl]phenyl}acetamide (7r)

Beige powder (0.15 g, 78.62% yield); m.p: 170–171 °C; ^1^ H NMR (500 MHz, DMSO-*d*_*6*_) δ (ppm): 3.83 (3 H, s, OCH_3_), 3.86 (3 H, s, OCH_3_), 3.90 (3 H, s, OCH_3_), 4.34 (1.60 H, s, SCH_2_), 4.43 (0.40 H, s, SCH_2_), 7.02 (2 H, d, *J* = 8.5 Hz, Ar-H), 7.16 (1 H, d, *J* = 8.5 Hz, Ar-H), 7.64 (1 H, s, Ar-H), 7.70 (1 H, d, *J* = 15.6 Hz, CH = CH), 7.76–7.84 (7 H, m, Ar-H + CH = CH), 7.86 (1 H, s, Ar-H), 8.13–8.20 (3 H, m, Ar-H), 10.83 (1 H, s, NH), 13.29 (1 H, s, NH); ^13^ C NMR (125 MHz, DMSO-*d*_*6*_) δ (ppm): 45.98, 55.84, 56.11, 56.12, 110.43, 110.44, 112.31, 114.86, 115.52, 116.88, 118.89, 119.02, 119.03, 119.86, 119.89, 120.18, 120.21, 122.59, 124.69, 124.72, 124.89, 125.04, 127.86, 130.27, 131.16, 143.70, 149.39, 151.02, 161.75, 168.17, 187.79; Anal. calcd. for C_35_H_29_N_5_O_6_S: C 64.90, H 4.51, N 10.81, S 4.95. Found: C 64.79, H 4.63, N 11.02, S 4.92.

##### 2-{5-[2-(3,4-Dimethoxyphenyl)-1* H*-benzo[*d*]imidazol-5-yl]-1,3,4-oxadiazol-2-ylthio}-*N*-{4-[(*E*)-3-(3,4-dimethoxyphenyl)acryloyl]phenyl}acetamide (7s)

Brown powder (0.20 g, 86.30% yield); m.p: 190–193 °C; ^1^ H NMR (500 MHz, DMSO-*d*_*6*_) δ (ppm): 3.82 (3 H, s, OCH_3_), 3.85 (3 H, s, OCH_3_), 3.87 (3 H, s, OCH_3_), 3.90 (3 H, s, OCH_3_), 4.26 (0.50 H, s, SCH_2_), 4.43 (1.50 H, s, SCH_2_), 6.98–7.07 (1 H, m, Ar-H), 7.15 (1 H, d, *J* = 8.2 Hz, Ar-H), 7.37–7.44 (2 H, m, Ar-H), 7.56 (1 H, d, *J* = 15.6 Hz, CH = CH), 7.62–7.69 (1 H, m, Ar-H), 7.71 (1 H, s, Ar-H), 7.82 (4 H, d, *J* = 8.8 Hz, Ar-H), 7.87 (1 H, d, *J* = 15.6 Hz, CH = CH), 8.13 (1 H, s, Ar-H), 8.20 (1 H, d, *J* = 8.8 Hz, Ar-H), 8.31 (1 H, d, *J* = 8.8 Hz, Ar-H), 10.88 (1 H, s, NH), 13.32 (1 H, s, NH); ^13^ C NMR (125 MHz, DMSO-*d*_*6*_) δ (ppm): 46.02, 56.05, 56.10, 56.21, 110.35, 110.41, 111.01, 112.00, 112.05, 112.30, 112.35, 116.87, 118.59, 118.95, 119.04, 119.88, 119.92, 122.39, 124.34, 128.06, 130.32, 130.35, 144.52, 149.41, 149.47, 151.13, 151.30, 151.65, 151.90, 165.74, 166.18, 166.62, 188.01; HRMS (ESI) m/z [M + H]^+^ calcd. for C_36_H_31_N_5_O_7_S: 678.2017, found 678.1952.

##### 2-{5-[2-(3,4-Dimethoxyphenyl)-1* H*-benzo[*d*]imidazol-5-yl]-1,3,4-oxadiazol-2-ylthio}-*N*-{4-[(*E*)-3-(3,4,5-trimethoxyphenyl)acryloyl]phenyl}acetamide (7t)

Brown crystal (0.165 g, 79.35% yield); m.p: 190–193 °C; ^1^ H NMR (500 MHz, DMSO-*d*_*6*_) δ (ppm): 3.72 (3 H, s, OCH_3_), 3.85 (3 H, s, OCH_3_), 3.87 (6 H, s, 2OCH_3_), 3.90 (3 H, s, OCH_3_), 4.24 (0.75 H, s, SCH_2_), 4.43 (1.25 H, s, SCH_2_), 7.14–7.17 (1 H, m, Ar-H), 7.23 (2 H, s, Ar-H), 7.26 (1 H, d, *J* = 8.6 Hz, Ar-H)), 7.65–7.70 (2 H, m, 1 Ar-H + CH = CH), 7.78 (1 H, s, Ar-H), 7.81 (3 H, d, *J* = 8.6 Hz, Ar-H), 7.84 (1 H, s, Ar-H), 7.90 (1 H, d, *J* = 15.4 Hz, CH = CH), 8.21 (1 H, d, *J* = 8.4 Hz, Ar-H), 8.31 (1 H, d, *J* = 8.4 Hz, Ar-H), 10.91 (1 H, s, NH), 13.24 (1 H, s, NH); ^13^ C NMR (125 MHz, DMSO-*d*_*6*_) δ (ppm): 46.05, 56.10, 56.59, 56.65, 60.60, 107.24, 110.15, 112.31, 116.65, 118.80, 121.20, 121.50, 127.89, 128.43, 129.77, 130.59, 131.12, 133.25, 139.46, 139.98, 140.29, 143.19, 143.49, 145.35, 145.65, 146.69, 149.08, 153.72, 159.07, 164.44, 166.54, 189.12; HRMS (ESI) m/z [M + H]^+^ calcd. for C_37_H_33_N_5_O_8_S: 708.2123, found 708.2062.

##### 2-{5-[2-(3,4,5-Trimethoxyphenyl)-1* H*-benzo[*d*]imidazol-5-yl]-1,3,4-oxadiazol-2-ylthio}-*N*-{4-[(*E*)-3-phenylacryloyl]phenyl}acetamide (7u)

Brown crystal (0.13 g, 73.35% yield); m.p: 188–190 °C; ^1^ H NMR (500 MHz, DMSO-*d*_*6*_) δ (ppm): 3.92 (6 H, s, 2OCH_3_), 3.94 (3 H, s, OCH_3_), 4.36 (1.25 H, s, SCH_2_), 4.46 (0.75 H, s, SCH_2_), 7.42–7.46 (4 H, m, Ar-H), 7.71 (2 H, d, *J* = 8.6 Hz, Ar-H), 7.75 (1 H, d, *J* = 15.6 Hz, CH = CH), 7.80–7.84 (2 H, m, 1 Ar-H + CH = CH), 7.88 (2 H, s, Ar-H), 7.93 (1 H, d, *J* = 6.6 Hz, Ar-H), 7.95–8.01 (1 H, m, Ar-H), 8.16 (3 H, d, *J* = 8.6 Hz, Ar-H), 10.90 (1 H, s, NH); ^13^ C NMR (125 MHz, DMSO-*d*_*6*_) δ (ppm): 44.46, 56.68, 57.58, 110.16, 112.69, 114.10, 119.25, 120.67, 122.08, 122.98, 124.11, 124.40, 124.62, 129.77, 130.59, 130.88, 131.18, 132.08, 135.43, 141.41, 143.71, 148.56, 151.11, 153.64, 154.23, 162.72, 165.35, 187.79; Anal. calcd. for C_35_H_29_N_5_O_6_S: C 64.90, H 4.51, N 10.81, S 4.95. Found: C 65.12, H 4.68, N 10.97, S 5.03.

##### 2-{5-[2-(3,4,5-Trimethoxyphenyl)-1* H*-benzo[*d*]imidazol-5-yl]-1,3,4-oxadiazol-2-ylthio}-*N*-{4-[(*E*)-3-(4-chlorophenyl)acryloyl]phenyl}acetamide (7v)

Brown crystal (0.16 g, 77.54% yield); m.p: 196–198 °C; ^1^ H NMR (500 MHz, DMSO-*d*_*6*_) δ (ppm): 3.75 (6 H, s, OCH_3_), 3.76 (3 H, s, 2OCH_3_), 4.25 (0.75 H, s, SCH_2_), 4.43 (1.25 H, s, SCH_2_), 7.51–7.52 (5 H, m, 4 Ar-H + CH = CH), 7.72 (2 H, d, *J* = 6.6 Hz, Ar-H), 7.77–7.82 (2 H, m, 1 Ar-H + CH = CH), 7.94 (2 H, s, Ar-H), 7.97 (2 H, d, *J* = 6.6 Hz, Ar-H), 8.15 (1 H, d, *J* = 8.5 Hz, Ar-H), 8.20 (1 H, d, *J* = 6.6 Hz, Ar-H), 10.92 (1 H, s, NH); ^13^ C NMR (125 MHz, DMSO-*d*_*6*_) δ (ppm): 46.02, 56.47, 60.78, 117.17, 119.03, 121.50, 123.13, 123.16, 123.19, 128.46, 129.38, 130.06, 130.29, 130.95, 130.97, 131.01, 134.32, 135.41, 135.43, 135.52, 136.48, 142.15, 142.45, 153.72, 153.74, 163.32, 164.66, 188.00; HRMS (ESI) m/z [M + H]^+^ calcd. for C_35_H_28_ClN_5_O_6_S: 682.1522, found: 682.1471.

##### 2-{5-[2-(3,4,5-Trimethoxyphenyl)-1* H*-benzo[*d*]imidazol-5-yl]-1,3,4-oxadiazol-2-ylthio}-*N*-{4-[(*E*)-3-(4-methoxyphenyl)acryloyl]phenyl}acetamide (7w)

Brown crystal (0.163 g, 76.65% yield); m.p: 200–204 °C; ^1^ H NMR (500 MHz, DMSO-*d*_*6*_) δ (ppm): 3.76 (3 H, s, OCH_3_), 3.82 (3 H, s, OCH_3_), 3.93 (6 H, s, 2OCH_3_), 4.26 (0.70 H, s, SCH_2_), 4.45 (1.30 H, s, SCH_2_), 7.00-7.02 (2 H, m, Ar-H), 7.69 (2 H, d, *J* = 6.6 Hz, Ar-H), 7.77 (2 H, d, *J* = 6.6 Hz, Ar-H), 7.80 (1 H, d, *J* = 15.4 Hz, CH = CH), 7.83 (2 H, d, *J* = 6.6 Hz, Ar-H), 7.84–7.87 (3 H, m, Ar-H + CH = CH), 8.15–8.18 (3 H, m, Ar-H), 11.06 (1 H, s, NH); ^13^ C NMR (125 MHz, DMSO-*d*_*6*_) δ (ppm): 44.23, 55.86, 56.99, 60.41, 105.01, 114.71, 118.65, 119.85, 122.26, 122.67, 127.23, 128.35, 129.47, 130.89, 130.94, 135.90, 142.61, 142.82, 144.03, 148.83, 149.09, 152.50, 153.11, 153.13, 161.62, 162.43, 162.73, 166.16, 187.79; HRMS (ESI) m/z [M + H]^+^ calcd. for C_36_H_31_N_5_O_7_S: 678.2017, found: 678.1996.

##### 2-{5-[2-(3,4,5-Trimethoxyphenyl)-1* H*-benzo[*d*]imidazol-5-yl]-1,3,4-oxadiazol-2-ylthio}-*N*-{4-[(*E*)-3-(3,4-dimethoxyphenyl)acryloyl]phenyl}acetamide (7x)

Brown crystal (0.21 g, 86.50% yield); m.p: 212–215°C; ^1^ H NMR (500 MHz, DMSO-*d*_*6*_) δ (ppm): 3.82 (3 H, s, OCH_3_), 3.87 (3 H, s, OCH_3_), 3.92 (6 H, s, 2OCH_3_), 3.93 (3 H, s, OCH_3_), 4.36 (1.25 H, s, SCH_2_), 4.45 (0.75 H, s, SCH_2_), 7.02 (1 H, d, *J* = 8.8 Hz, Ar-H), 7.36–7.39 (1 H, m, Ar-H), 7.66–7.72 (3 H, m, 2 Ar-H + CH = CH), 7.76–7.79 (2 H, m, 1 Ar-H + CH = CH), 7.81 (2 H, s, Ar-H), 8.83 (2 H, d, *J* = 7.5 Hz, Ar-H), 8.15–8.19 (3 H, m, Ar-H), 11.03 (1 H, s, NH); ^13^ C NMR (125 MHz, DMSO-*d*_*6*_) δ (ppm); 44.46, 54.42, 56.06, 56.22, 56.97, 104.78, 110.45, 111.88, 117.22, 119.16, 119.34, 120.02, 122.57, 124.10, 124.47, 124.63, 128.08, 130.26, 132.90, 133.40, 139.78, 139.97, 144.97, 145.30, 148.64, 149.41, 151.39, 151.59, 160.22, 164.69, 167.57, 187.78; Anal. calcd. for C_37_H_33_N_5_O_8_S: C 62.79, H 4.70, N 9.90, S 4.53. Found: C 62.95, H 4.82, N 10.16, S 4.60.

### Biology

#### Screening of anti-proliferative activity by NCI

The methodology of the NCI anti-proliferative screening has been described in details elsewhere (http://www.dtp.nci.nih.gov).

#### Cytotoxic activity using MTT assay and determination of IC_50_

##### Assay for anti-proliferative effect in melanoma LOX-IMVI cell lines

Using the propidium iodide fluorescence assay with Staurosporine as the reference drug, the antiproliferative activities of compounds **7g**, **7l**, **7p**, **7q**, and **7v**, was carried out according to a previously reported procedure^31, 32^ to detect IC_50_ against LOX-IMVI cell lines. compounds 7g, 7l, 7p, 7q, and 7v were incubated with LOX-IMVI cells for 2 days. See Appendix A.

##### Assay for anti-proliferative effect

Using the propidium iodide fluorescence assay with doxorubicin as the reference drug, IC_50_ of the 24 compounds **7a-x**, was carried out according to a previously reported procedure^31, 32^ against a panel of four human cancer cell lines; epithelial cancer cell line (A-549), breast cancer cell line (MCF-7), colon cancer cell line (HT-29) and pancreas cancer cell line (Panc-1). Compounds **7a-x** were incubated with cancer cells for 2 days at different concentrations. See Appendix A.

##### Cell viability determination

MTT assay was done to determine the effect of the synthesized compounds on the viability of mammary epithelial cells (MCF-10 A) [33, 34]. Compounds **7a-x** were incubated with MCF-10 A cells for 4 days at 50 µM concentration, and the viability of cells was determined. See Appendix A.

#### EGFR inhibitory assay

The inhibitory efficacy of of the most active compounds **7e**, **7g**, **7h**, **7k-n**, **7p**, **7q**, and **7v** against EGFR was evaluated using the EGFR-TK assay [35, 36]. See Appendix A.

#### BRAF kinase assay

Compounds **7e**, **7g**, **7h**, **7k-n**, **7p**, **7q**, and **7v** were further examined for their ability to inhibit the V600E mutant BRAF using kinase assay against BRAF^V600E^. ^24, 37^ See Appendix A.

#### Apoptosis-induction activity detection

##### Caspases assays

The effect of compounds; **7k**, **7l**, and **7m** on caspases-3,8,9 was determined and compared to doxorubicin as a control according to reported assays [38]. See Appendix A.

##### Cytochrome C assay

Compounds **7k**, **7l**, and **7m** were evaluated as Cytochrome C activators in the MCF-7 human breast cancer cell line according to previously reported assays [39]. See Appendix A.

##### BAX activation assay

The most potent caspase activators **7k**, **7l**, and **7m** were investigated for their influence on BAX level in a breast cancer cell line (MCF-7) using doxorubicin as a control according to reported assay [38]. See Appendix A.

##### Bcl-2 Inhibition assay

The most potent caspase activators **7k**, **7l**, and **7m** were examined for their influence on Bacl-2 level in a breast cancer cell line (MCF-7) using doxorubicin as a control according to reported assay [38]. See Appendix A.

#### Cell apoptosis assay

Apoptosis was determined by flow cytometry based on the Annexin-V-fluoresce in isothiocyanate (FITC) and propidium iodide (PI) staining kit (BD Pharmingen, San Diego, USA) [40, 41] See Appendix A.

## Docking studies

The most bioactive derivatives (i.e. **7e**, **7g**, **7h**, **7k-n**, **7p**, **7q**, and **7v**) were drawn and docked in the active site of EGFR (PDB ID: 1M17) [48–50] and BRAF (PDB ID: 3OG7) using AutoDock Vina software program as reported in the literature. See Appendix A.

### Electronic supplementary material

Below is the link to the electronic supplementary material.

Supplementary Material 1

Supplementary Material 2

## Data Availability

Data and materials of our Figures or Tables are available with us.
