# Supplementary material for: Chalcone/1,3,4-Oxadiazole/Benzimidazole hybrids as novel anti-proliferative agents inducing apoptosis and inhibiting EGFR & BRAFV600E
Source: BMC Chem. 2023 Sep 16;17(1):116. doi: 10.1186/s13065-023-01003-3 (PMC10504751; doi:10.1186/s13065-023-01003-3)
Supplement: Supplementary file 1 — Supplementary Material 1 [file 13065_2023_1003_MOESM1_ESM.docx]

**Supplementary data**

**Chalcone/1,3,4-Oxadiazole/Benzimidazole Hybrids as Novel Anti-proliferative Agents Inducing Apoptosis and Inhibiting EGFR & BRAFV^600E^**

Fatma Hagar^1^ | Samar Abbas^1^ | Hesham Gomaa^2^ | Bahaa Youssif^3^ | Ahmed Sayed^4^ | Dalia Abdelhamid*****^1^ | Mohamed Abdel-Aziz^1^

^1^ Medicinal Chemistry Department, Faculty of Pharmacy, Minia University, Minia, Egypt

^2^ Pharmacology Department, College of Pharmacy, Jouf University, Sakaka, Saudi Arabia

^3^ Pharmaceutical Organic Chemistry Department, Faculty of Pharmacy, Assiut University, Assiut, Egypt

^4^ Pharmacognosy Department, Faculty of Pharmacy, Nahda University, Benisuef, Egypt

*** Corresponding Author:**

Dalia Abdelhamid; e-mail: daliaa_abdelhameed@mu.edu.eg

| **Fig. No.** |  | **Page No.** |
| --- | --- | --- |
| **S1** | **^1^H NMR of compound 7a (500 MHz, DMSO-*d_6_*)** | **6** |
| **S2** | **^13^C NMR of compound 7a (125 MHz, DMSO-*d_6_*)** | **7** |
| **S3** | **HRMS (ESI) spectrum of compound** **7a.** | **7** |
| **S4** | **^1^H NMR of compound 7b (500 MHz, DMSO-*d_6_*)** | **8** |
| **S5** | **^13^C NMR of compound 7b (125 MHz, DMSO-*d_6_*)** | **9** |
| **S6** | **HRMS (ESI) spectrum of compound** **7b.** | **9** |
| **S7** | **^1^H NMR of compound 7c (500 MHz, DMSO-*d_6_*)** | **10** |
| **S8** | **^13^C NMR of compound 7c (125 MHz, DMSO-*d_6_*)** | **11** |
| **S9** | **HRMS (ESI) spectrum of compound** **7c.** | **11** |
| **S 10** | **^1^H NMR of compound 7d (500 MHz, DMSO-*d_6_*)** | **12** |
| **S 11** | **^13^C NMR of compound 7d (125 MHz, DMSO-*d_6_*)** | **13** |
| **S 12** | **HRMS (ESI) spectrum of compound** **7d.** | **13** |
| **S 13** | **^1^H NMR of compound 7e (500 MHz, DMSO-*d_6_*)** | **14** |
| **S 14** | **^13^C NMR of compound 7e (125 MHz, DMSO-*d_6_*)** | **15** |
| **S 15** | **HRMS (ESI) spectrum of compound** **7e.** | **15** |
| **S 16** | **^1^H NMR of compound 7f (500 MHz, DMSO-*d_6_*)** | **16** |
| **S 17** | **^13^C NMR of compound 7f (125 MHz, DMSO-*d_6_*)** | **17** |
| **S 18** | **HRMS (ESI) spectrum of compound** **7f.** | **17** |
| **S 19** | **^1^H NMR of compound 7g (500 MHz, DMSO-*d_6_*)** | **18** |
| **S 20** | **^13^C NMR of compound 7g (125 MHz, DMSO-*d_6_*)** | **19** |
| **S 21** | **^1^H NMR of compound 7h (500 MHz, DMSO-*d_6_*)** | **20** |
| **S 22** | **^13^C NMR of compound 7h (125 MHz, DMSO-*d_6_*)** | **21** |
| **S 23** | **^1^H NMR of compound 7i (500 MHz, DMSO-*d_6_*)** | **22** |
| **S 24** | **^13^C NMR of compound 7i (125 MHz, DMSO-*d_6_*)** | **23** |
| **S 25** | **^1^H NMR of compound 7j (500 MHz, DMSO-*d_6_*)** | **24** |
| **S 26** | **^13^C NMR of compound 7j (125 MHz, DMSO-*d_6_*)** | **25** |
| **S 27** | **^1^H NMR of compound 7k (500 MHz, DMSO-*d_6_*)** | **26** |
| **S 28** | **^13^C NMR of compound 7k (125 MHz, DMSO-*d_6_*)** | **27** |
| **S 29** | **HRMS (ESI) spectrum of compound** **7k.** | **27** |
| **S 30** | **^1^H NMR of compound 7l (500 MHz, DMSO-*d_6_*)** | **28** |
| **S 31** | **^13^C NMR of compound 7l (125 MHz, DMSO-*d_6_*)** | **29** |
| **S 32** | **^1^H NMR of compound 7m (500 MHz, DMSO-*d_6_*)** | **30** |
| **S 33** | **^13^C NMR of compound 7m (125 MHz, DMSO-*d_6_*)** | **31** |
| **S 34** | **HRMS (ESI) spectrum of compound** **7m.** | **31** |
| **S 35** | **^1^H NMR of compound 7n (500 MHz, DMSO-*d_6_*)** | **32** |
| **S 36** | **^13^C NMR of compound 7n (125 MHz, DMSO-*d_6_*)** | **33** |
| **S 37** | **^1^H NMR of compound 7o (500 MHz, DMSO-*d_6_*)** | **34** |
| **S 38** | **^13^C NMR of compound 7o (125 MHz, DMSO-*d_6_*)** | **35** |
| **S 39** | **HRMS (ESI) spectrum of compound** **7o.** | **35** |
| **S 40** | **^1^H NMR of compound 7p (500 MHz, DMSO-*d_6_*)** | **36** |
| **S 41** | **^13^C NMR of compound 7p (125 MHz, DMSO-*d_6_*)** | **37** |
| **S 42** | **HRMS (ESI) spectrum of compound** **7p.** | **37** |
| **S 43** | **^1^H NMR of compound 7q (500 MHz, DMSO-*d_6_*)** | **38** |
| **S 44** | **^13^C NMR of compound 7q (125 MHz, DMSO-*d_6_*)** | **39** |
| **S 45** | **HRMS (ESI) spectrum of compound** **7q.** | **39** |
| **S 46** | **^1^H NMR of compound 7r (500 MHz, DMSO-*d_6_*)** | **40** |
| **S 47** | **^13^C NMR of compound 7r (125 MHz, DMSO-*d_6_*)** | **41** |
| **S 48** | **^1^H NMR of compound 7s (500 MHz, DMSO-*d_6_*)** | **42** |
| **S 49** | **^13^C NMR of compound 7s (125 MHz, DMSO-*d_6_*)** | **43** |
| **S 50** | **HRMS (ESI) spectrum of compound** **7s.** | **43** |
| **S 51** | **^1^H NMR of compound 7t (500 MHz, DMSO-*d_6_*)** | **44** |
| **S 52** | **^13^C NMR of compound 7t (125 MHz, DMSO-*d_6_*)** | **45** |
| **S 53** | **HRMS (ESI) spectrum of compound** **7t.** | **45** |
| **S 54** | **^1^H NMR of compound 7u (500 MHz, DMSO-*d_6_*)** | **46** |
| **S 55** | **^13^C NMR of compound 7u (125 MHz, DMSO-*d_6_*)** | **47** |
| **S 56** | **^1^H NMR of compound 7v (500 MHz, DMSO-*d_6_*)** | **48** |
| **S 57** | **^13^C NMR of compound 7v (125 MHz, DMSO-*d_6_*)** | **49** |
| **S 58** | **HRMS (ESI) spectrum of compound** **7v.** | **49** |
| **S 59** | **^1^H NMR of compound 7w (500 MHz, DMSO-*d_6_*)** | **50** |
| **S 60** | **^13^C NMR of compound 7w (125 MHz, DMSO-*d_6_*)** | **51** |
| **S 61** | **HRMS (ESI) spectrum of compound** **7w.** | **51** |
| **S 62** | **^1^H NMR of compound 7x (500 MHz, DMSO-*d_6_*)** | **52** |
| **S 63** | **^13^C NMR of compound 7x (125 MHz, DMSO-*d_6_*)** | **53** |
| **S 64** | **One dose mean graph of nine different cancer cell line panels for compound 7a** | **54** |
| **S 65** | **One dose mean graph of nine different cancer cell line panels for compound 7b** | **55** |
| **S 66** | **One dose mean graph of nine different cancer cell line panels for compound 7c** | **56** |
| **S 67** | **One dose mean graph of nine different cancer cell line panels for compound 7d** | **57** |
| **S 68** | **One dose mean graph of nine different cancer cell line panels for compound 7e** | **58** |
| **S 69** | **One dose mean graph of nine different cancer cell line panels for compound 7f** | **59** |
| **S 70** | **One dose mean graph of nine different cancer cell line panels for compound 7g** | **60** |
| **S 71** | **One dose mean graph of nine different cancer cell line panels for compound 7h** | **61** |
| **S 72** | **One dose mean graph of nine different cancer cell line panels for compound 7k** | **62** |
| **S 73** | **One dose mean graph of nine different cancer cell line panels for compound 7l** | **63** |
| **S 74** | **One dose mean graph of nine different cancer cell line panels for compound 7m** | **64** |
| **S 75** | **One dose mean graph of nine different cancer cell line panels for compound 7n** | **65** |
| **S 76** | **One dose mean graph of nine different cancer cell line panels for compound 70** | **66** |
| **S 77** | **One dose mean graph of nine different cancer cell line panels for compound 7p** | **67** |
| **S 78** | **One dose mean graph of nine different cancer cell line panels for compound 7q** | **68** |
| **S 79** | **One dose mean graph of nine different cancer cell line panels for compound 7r** | **69** |
| **S 80** | **One dose mean graph of nine different cancer cell line panels for compound 7u** | **70** |
| **S 81** | **One dose mean graph of nine different cancer cell line panels for compound 7v** | **71** |
|  | **List of Tables** |  |

| **Table NO** | |  | **Page NO** |
| --- | --- | --- | --- |
| **1**    2 | **Table S1.** Cell growth inhibition % from the NCI's *in vitro* human tumor cell screen for compounds **7a:7e and 7k:7o.**  **Table S2.** Cell growth inhibition % from the NCI's *in vitro* human tumor cell screen for compounds **7p, 7q, 7r, 7u, 7v and 7f to 7h.** | | **72-75**  76-78 |

**
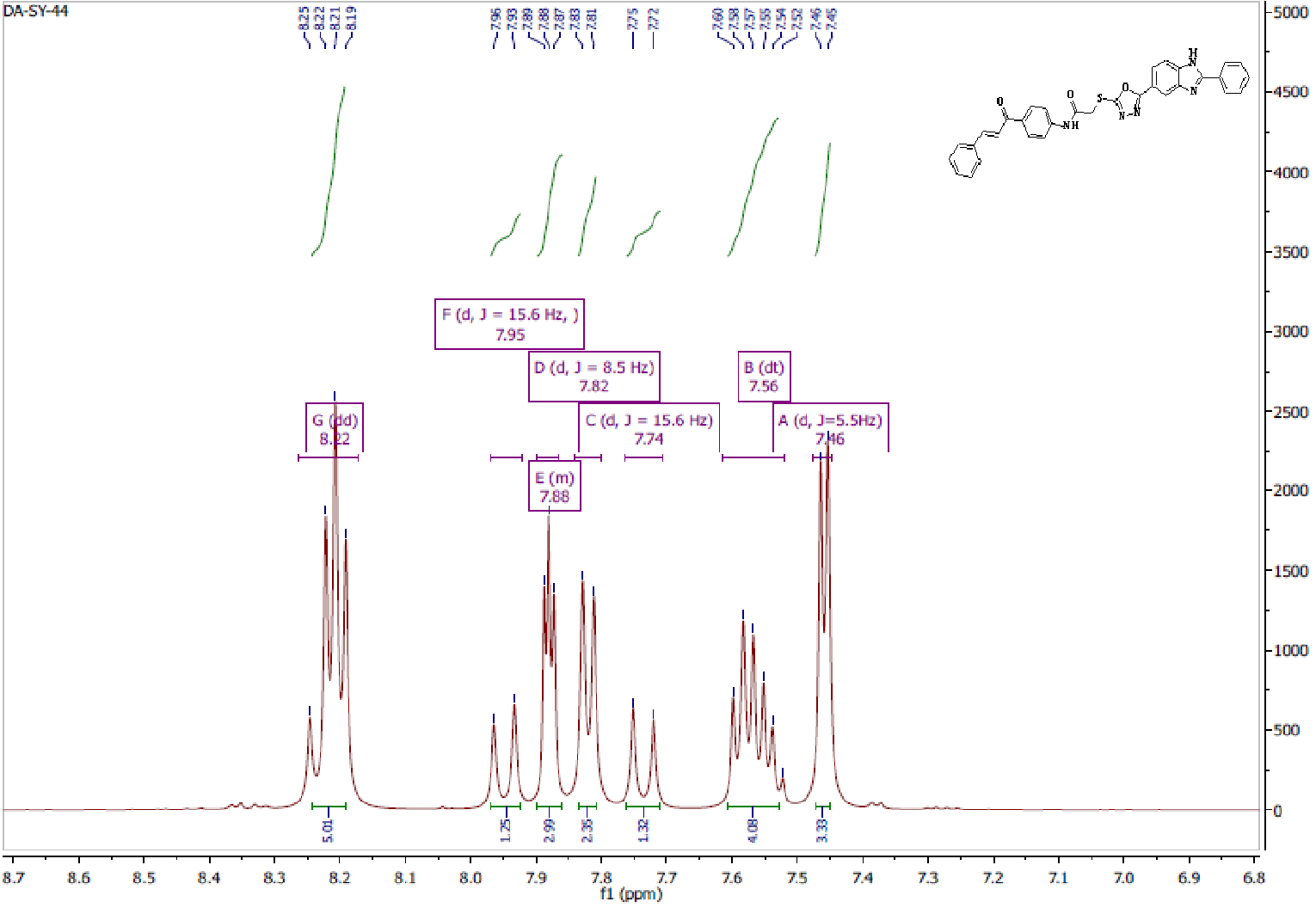
**

**
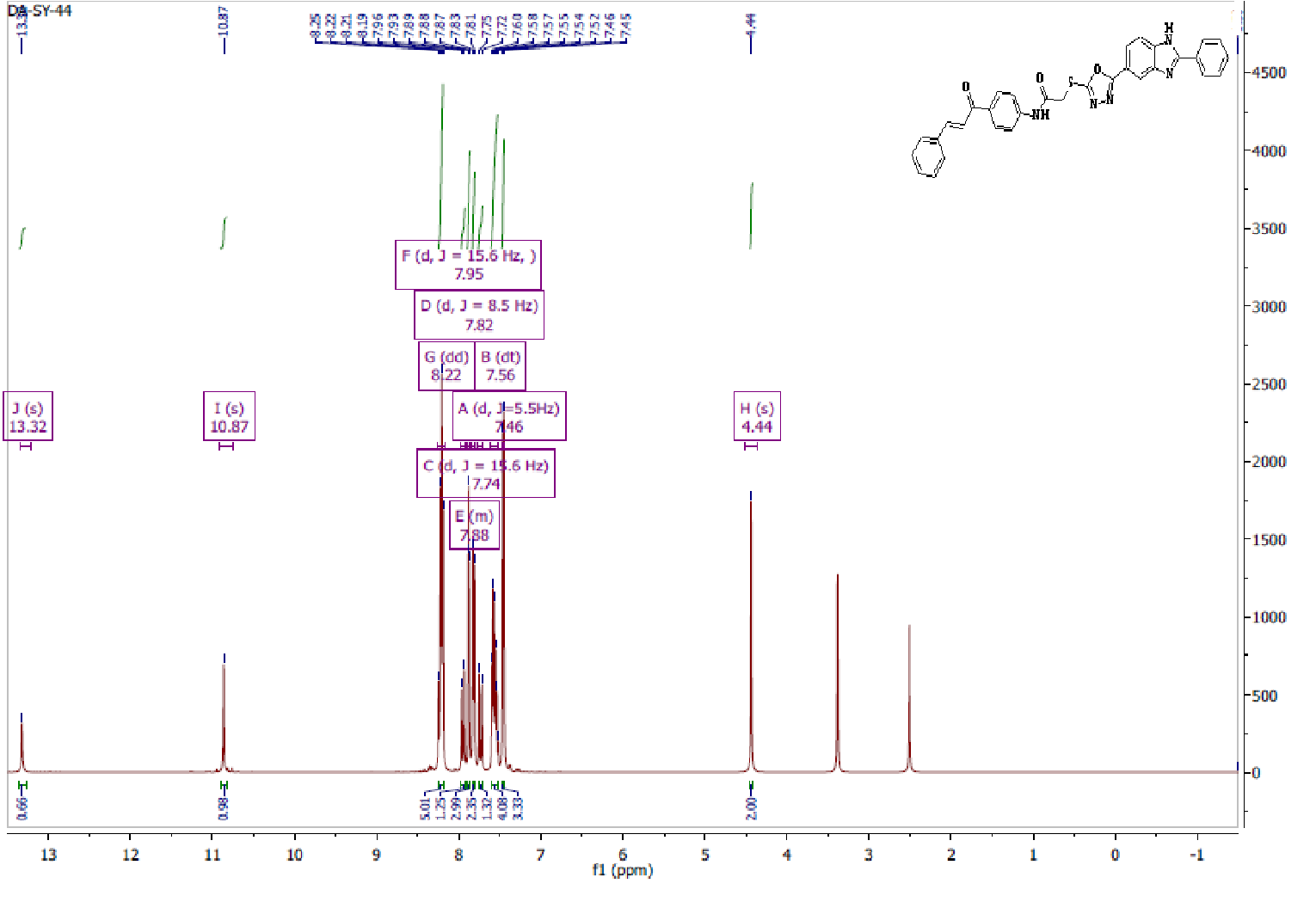
**

**Fig. S1. ^1^H NMR of compound 7a (500 MHz, DMSO-*d_6_*)**

**
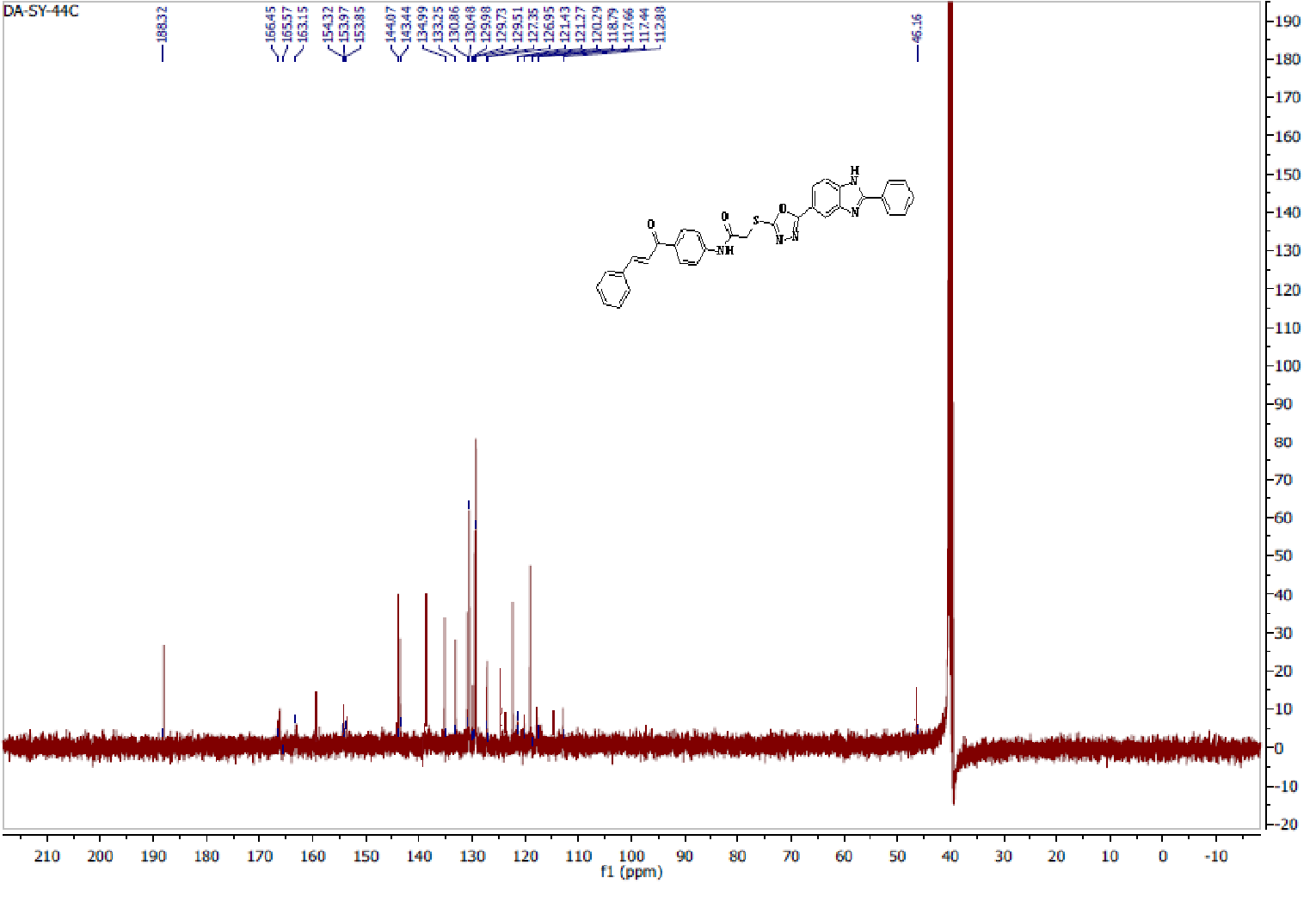
**

**Fig. S2. ^13^C NMR of compound 7a (125 MHz, DMSO-*d_6_*)**

**
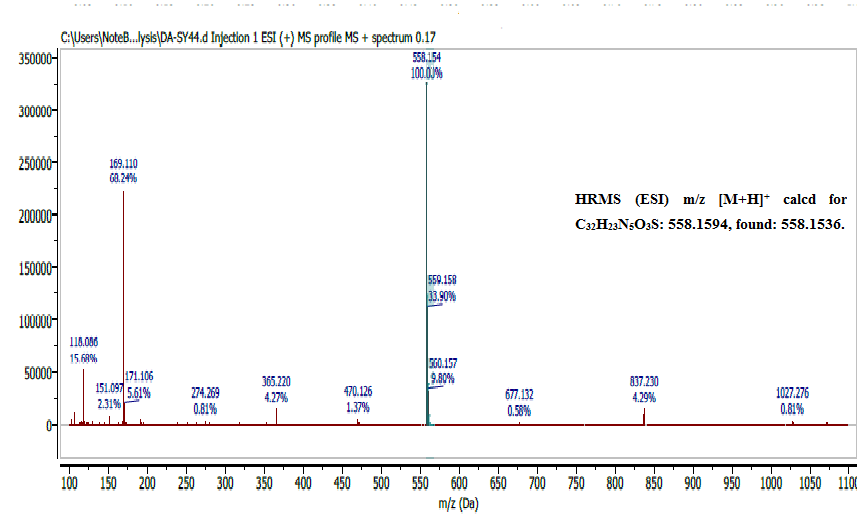
**

**Fig. S3:** **HRMS (ESI) spectrum of compound** **7a.**

**
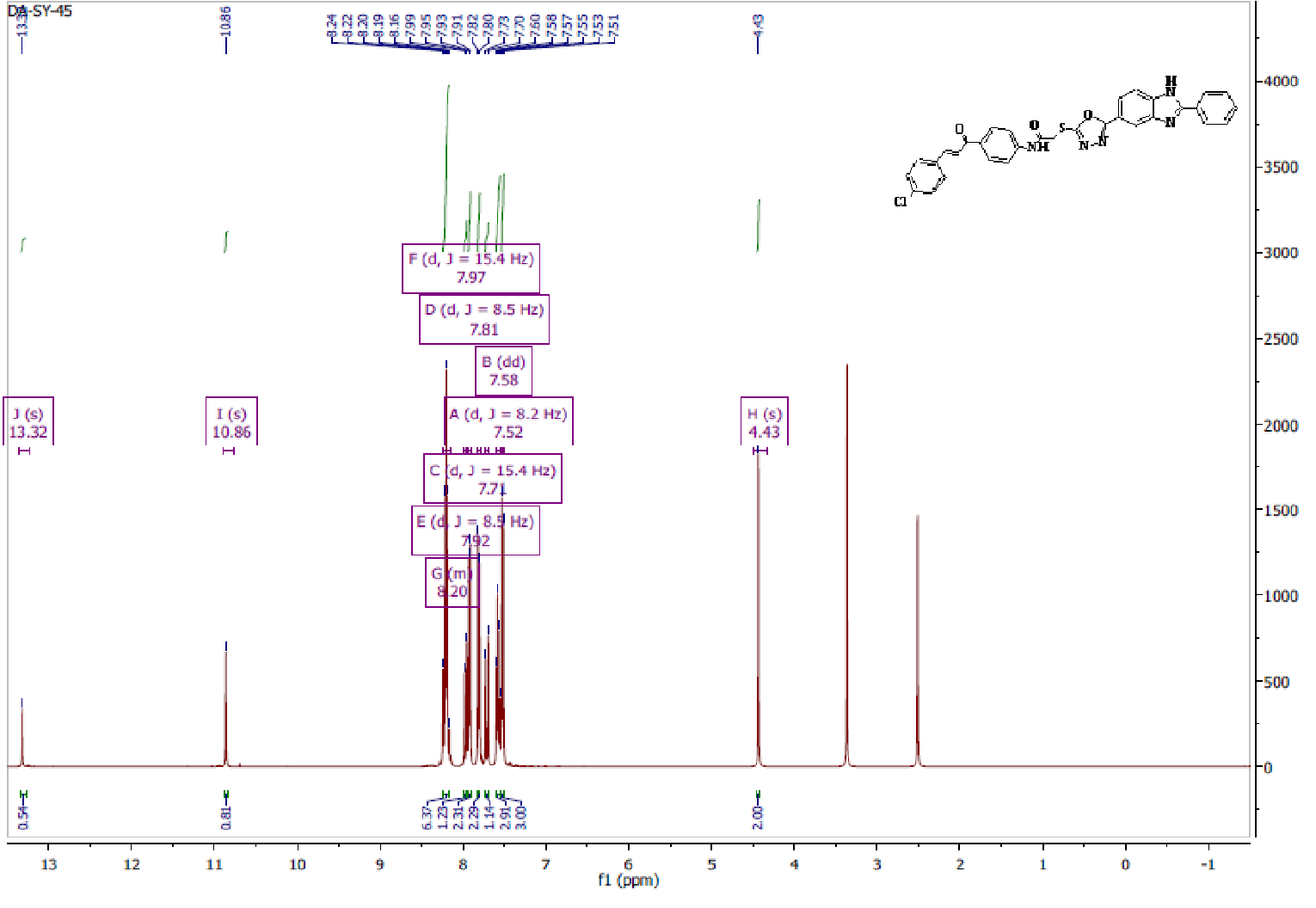
**

**
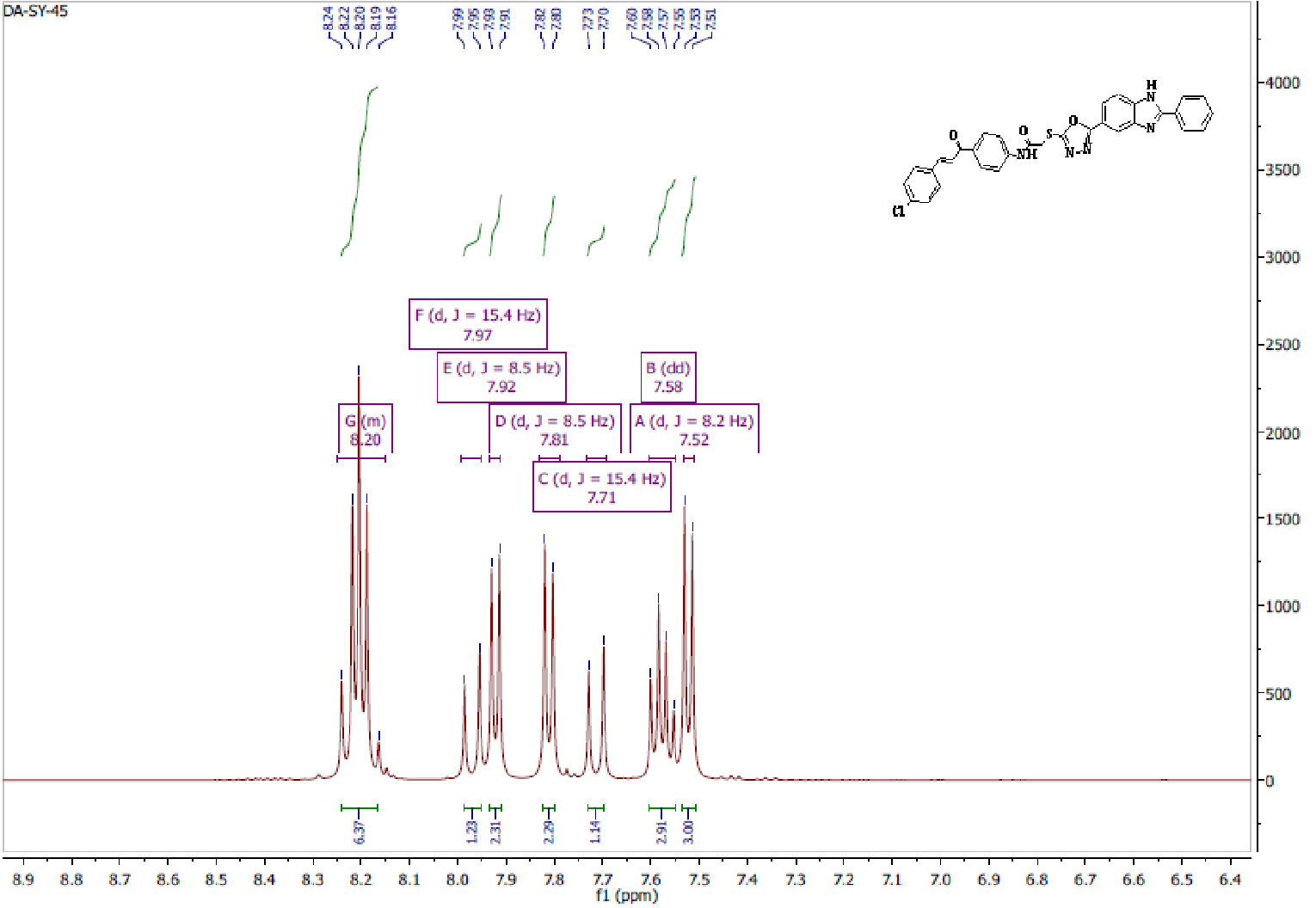
**

**Fig. S4. ^1^H NMR of compound 7b (500 MHz, DMSO-*d_6_*)**

**
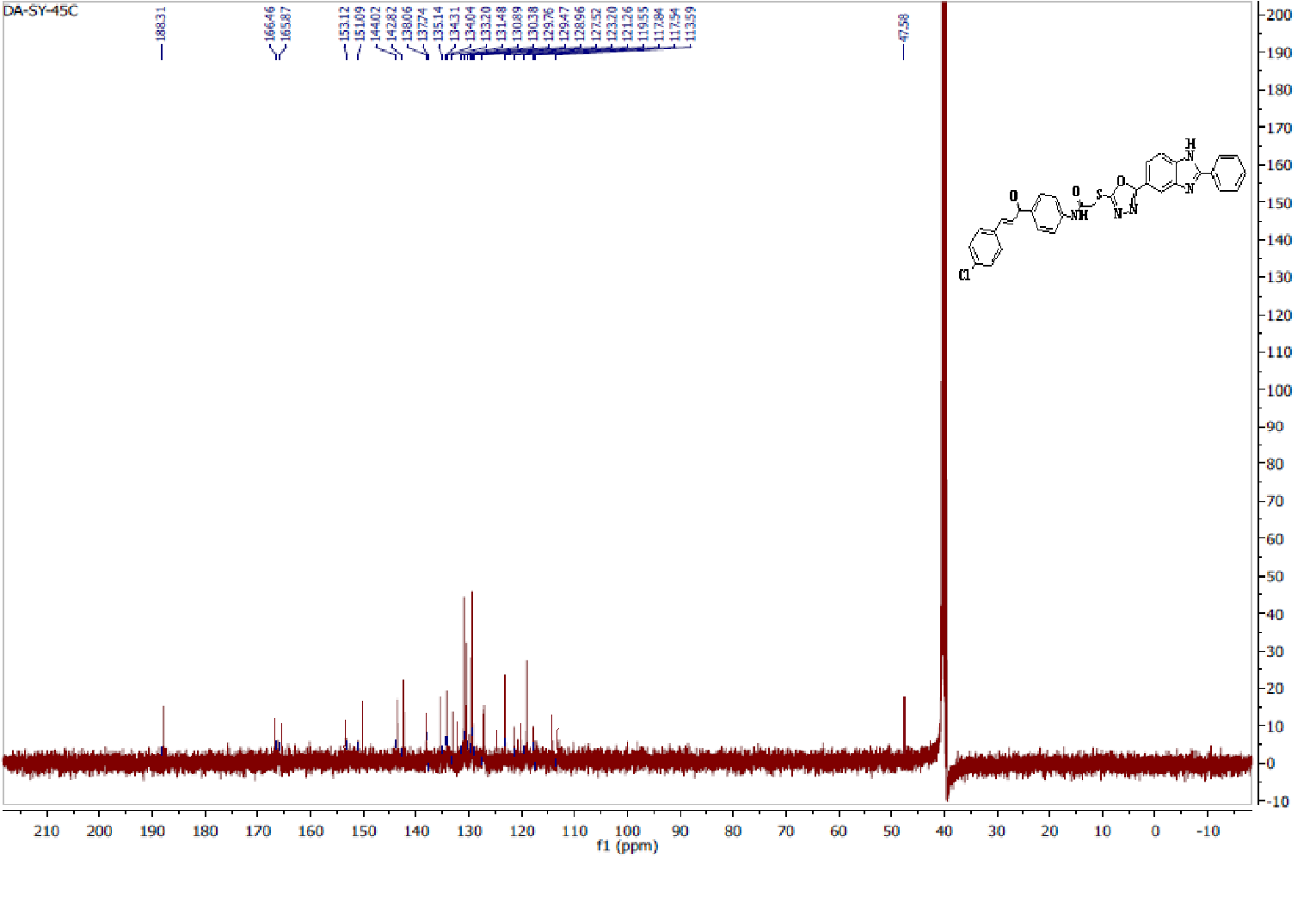
Fig. S5. ^13^C NMR of compound 7b (125 MHz, DMSO-*d_6_*)**

**
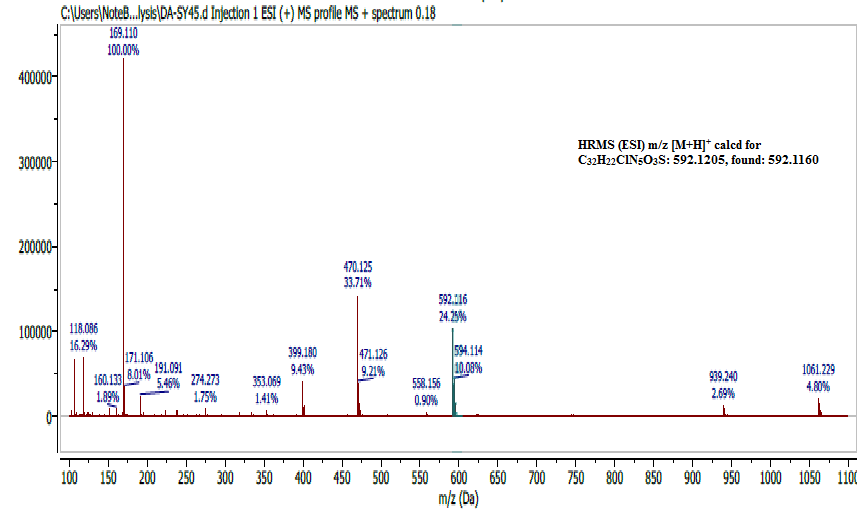
**

**Fig. S6:** **HRMS (ESI) spectrum of compound** **7b.**

**
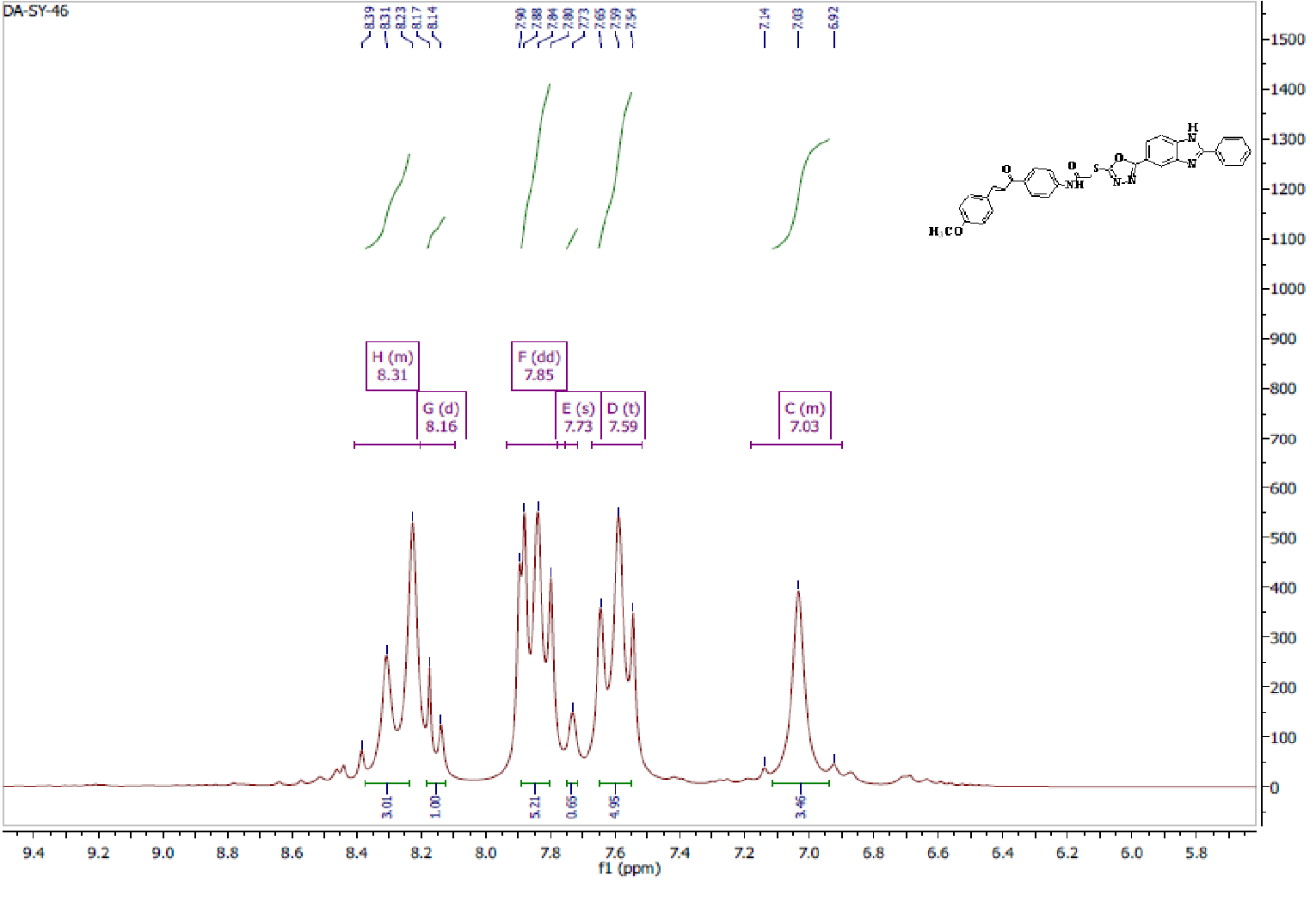
**

**
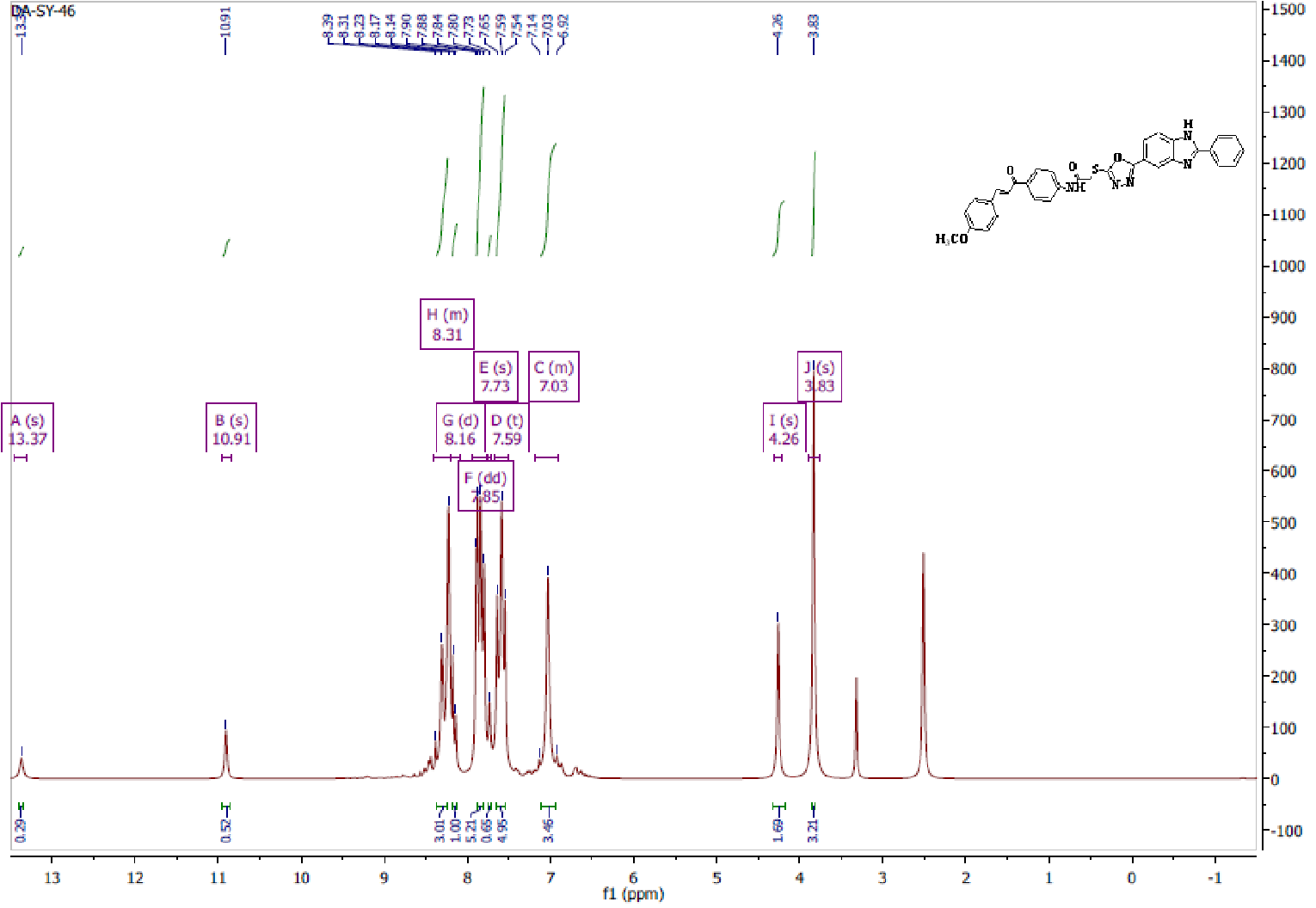
**

**Fig. S 7. ^1^H NMR of compound 7c (500 MHz, DMSO-*d_6_*)**

**
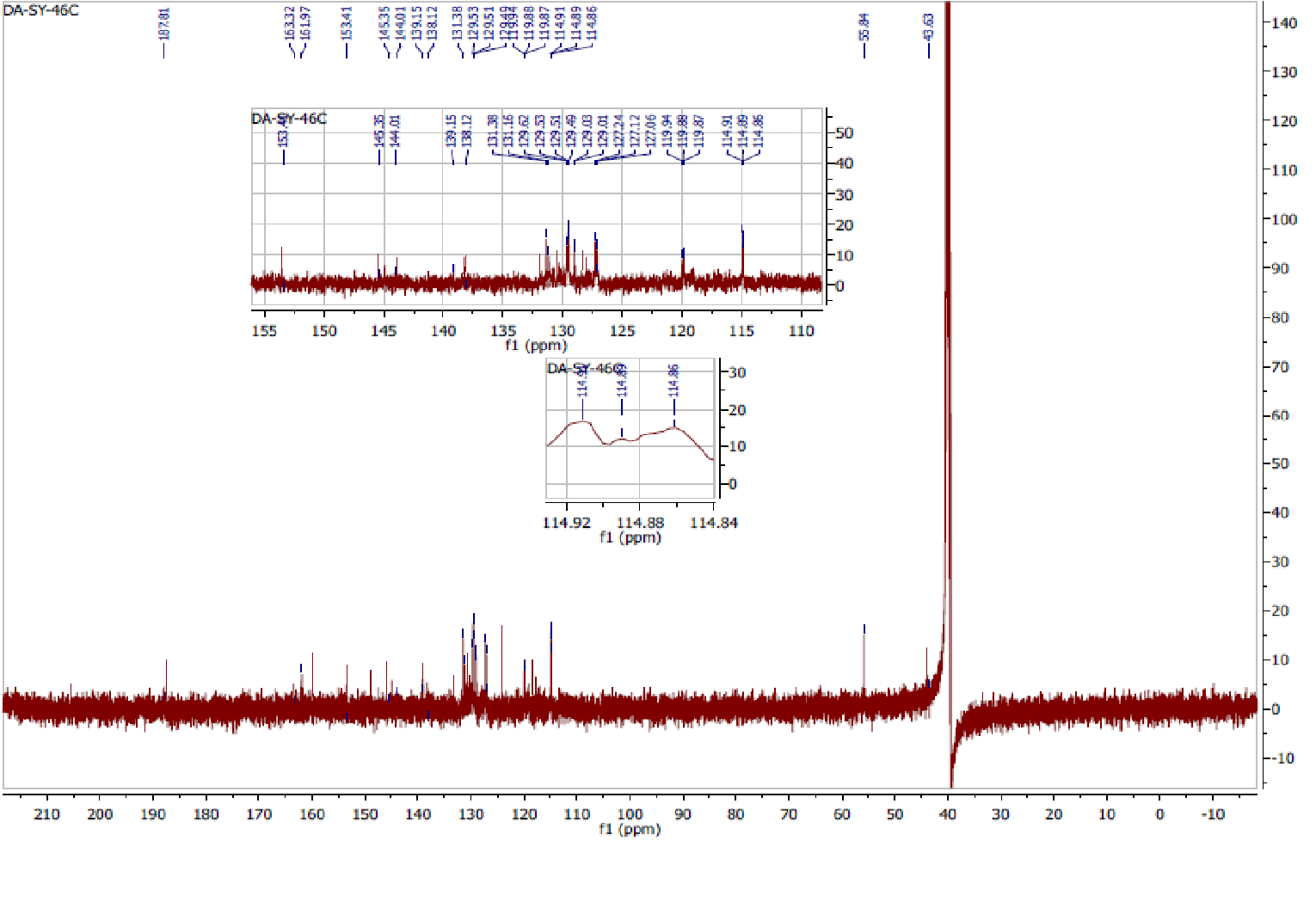
**

**Fig. S 8. ^13^C NMR of compound 7c (125 MHz, DMSO-*d_6_*)**

**
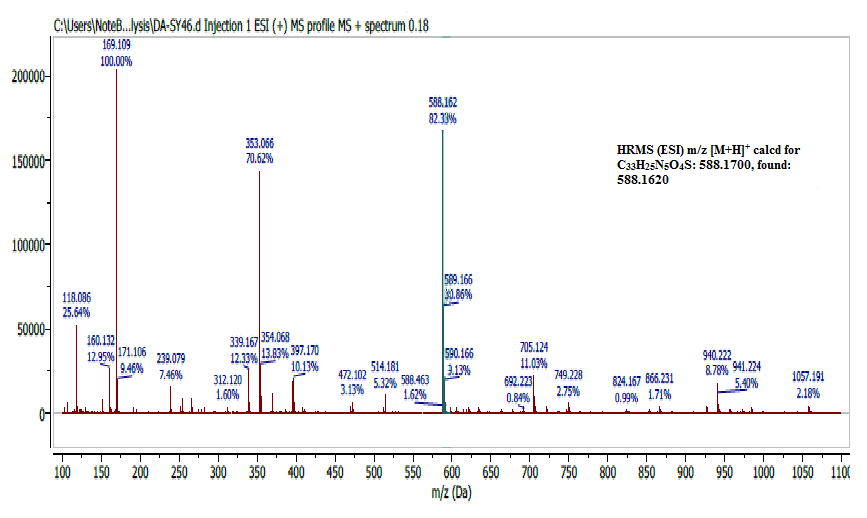
**

**Fig. S 9:** **HRMS (ESI) spectrum of compound** **7c.**

**
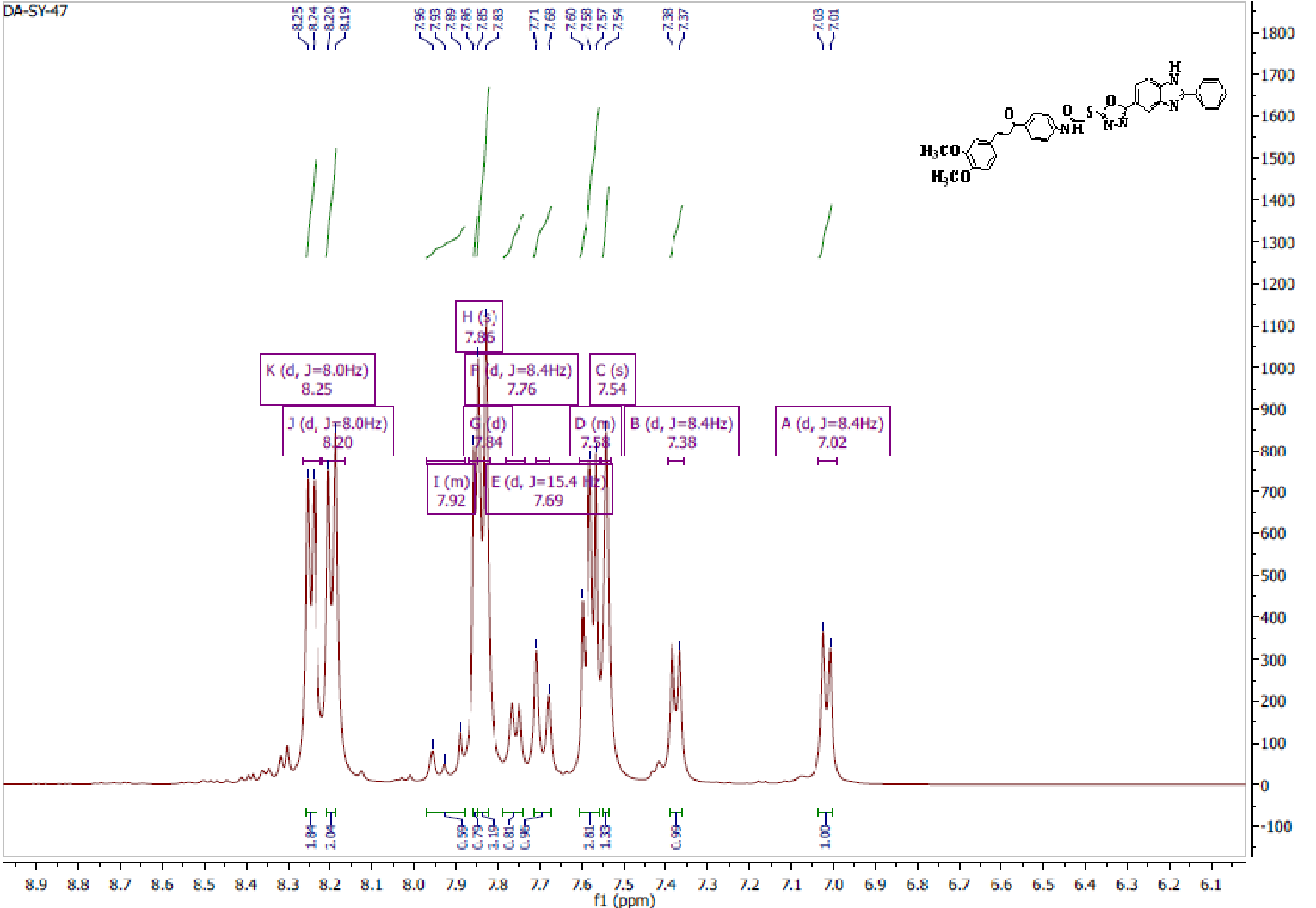
**

**
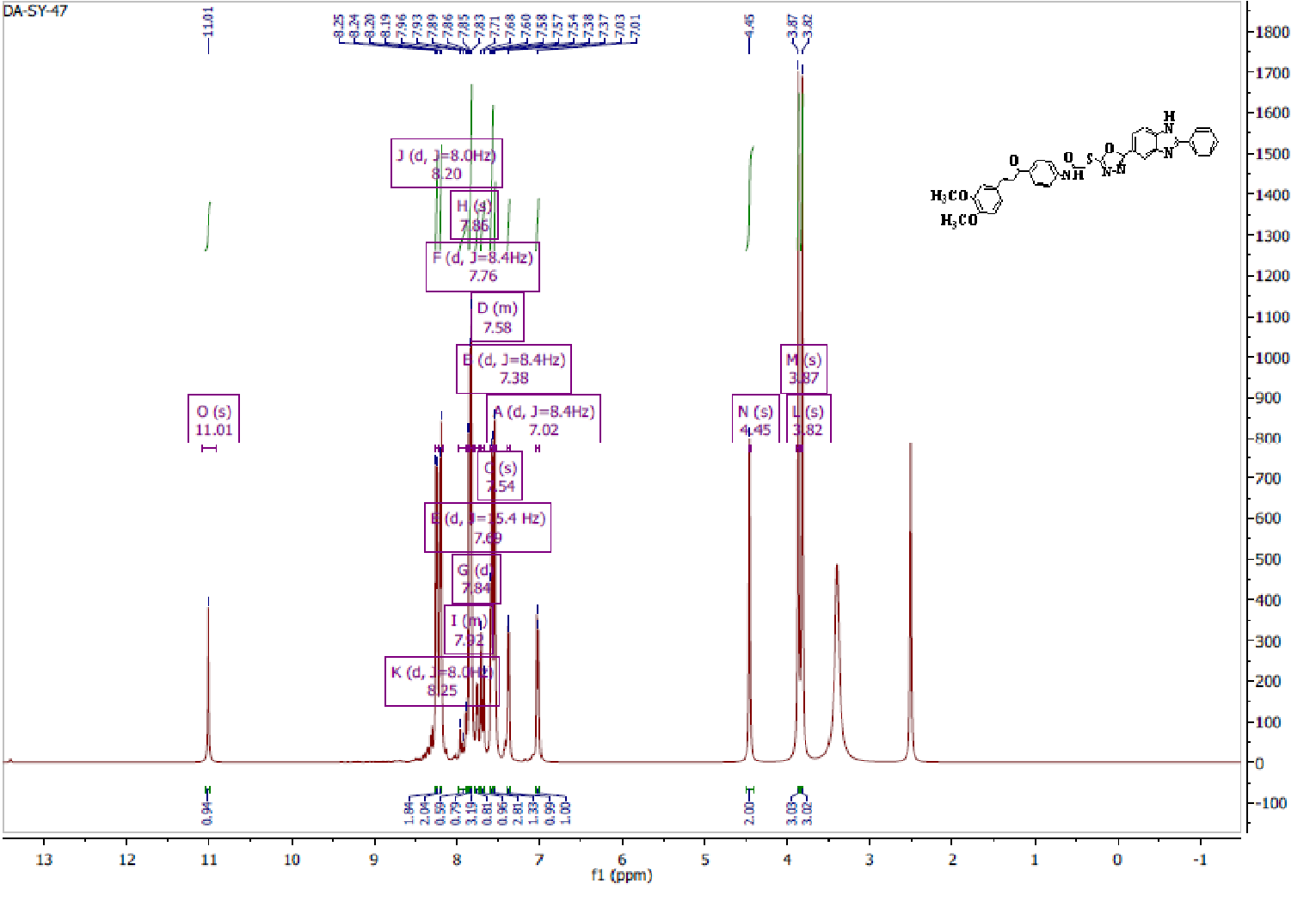
**

**Fig. S 10. ^1^H NMR of compound 7d (500 MHz, DMSO-*d_6_*)**

**
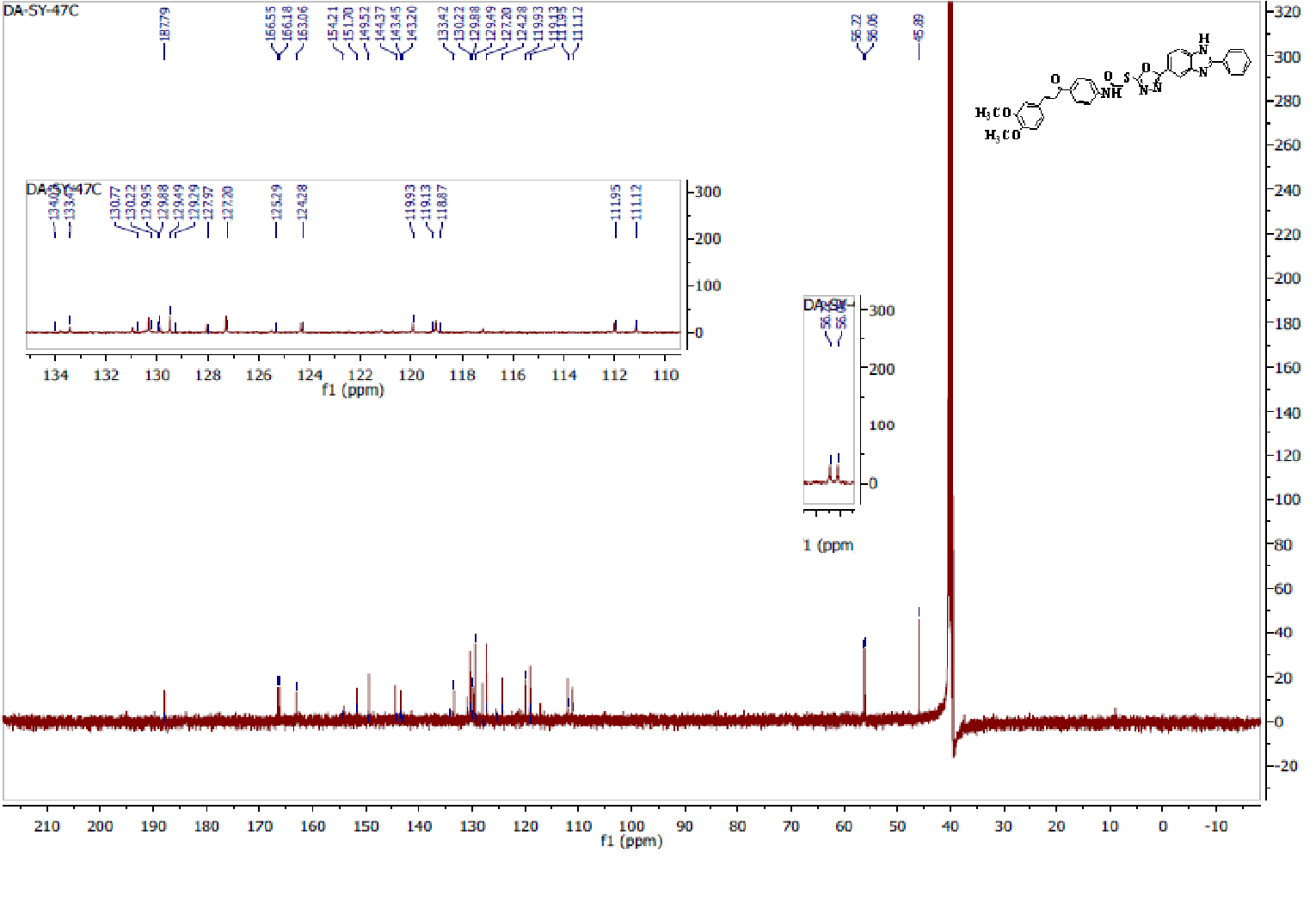
**

**Fig. S 11. ^13^C NMR of compound 7d (125 MHz, DMSO-*d_6_*)**

**
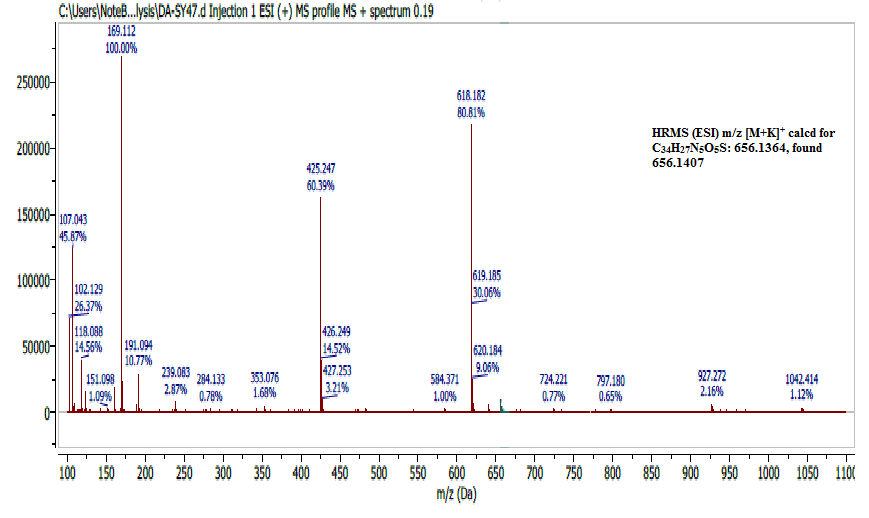
**

**Fig. S 12:** **HRMS (ESI) spectrum of compound** **7d.**

**
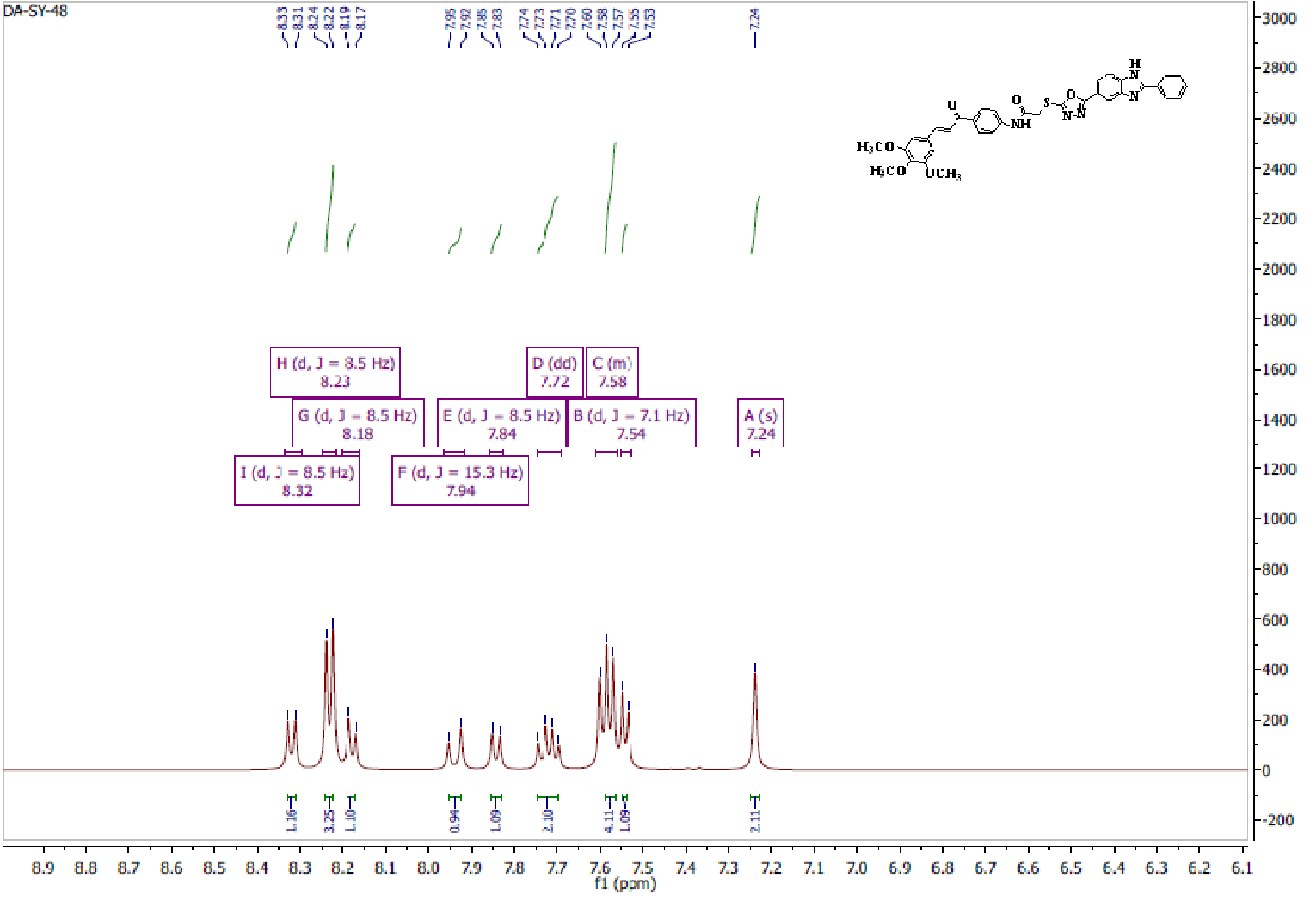
**

**
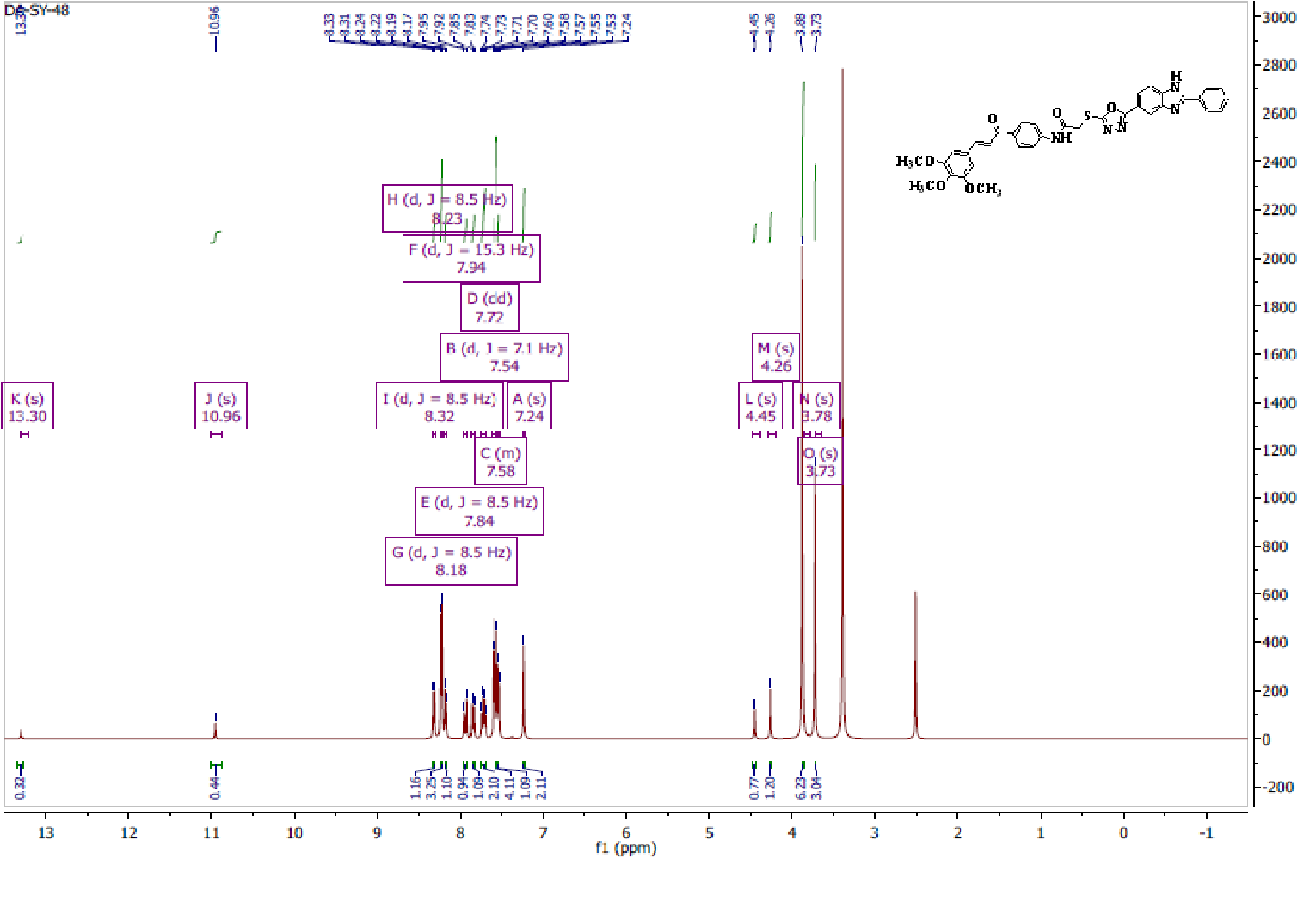
**

**Fig. S 13. ^1^H NMR of compound 7e (500 MHz, DMSO-*d_6_*)**

**
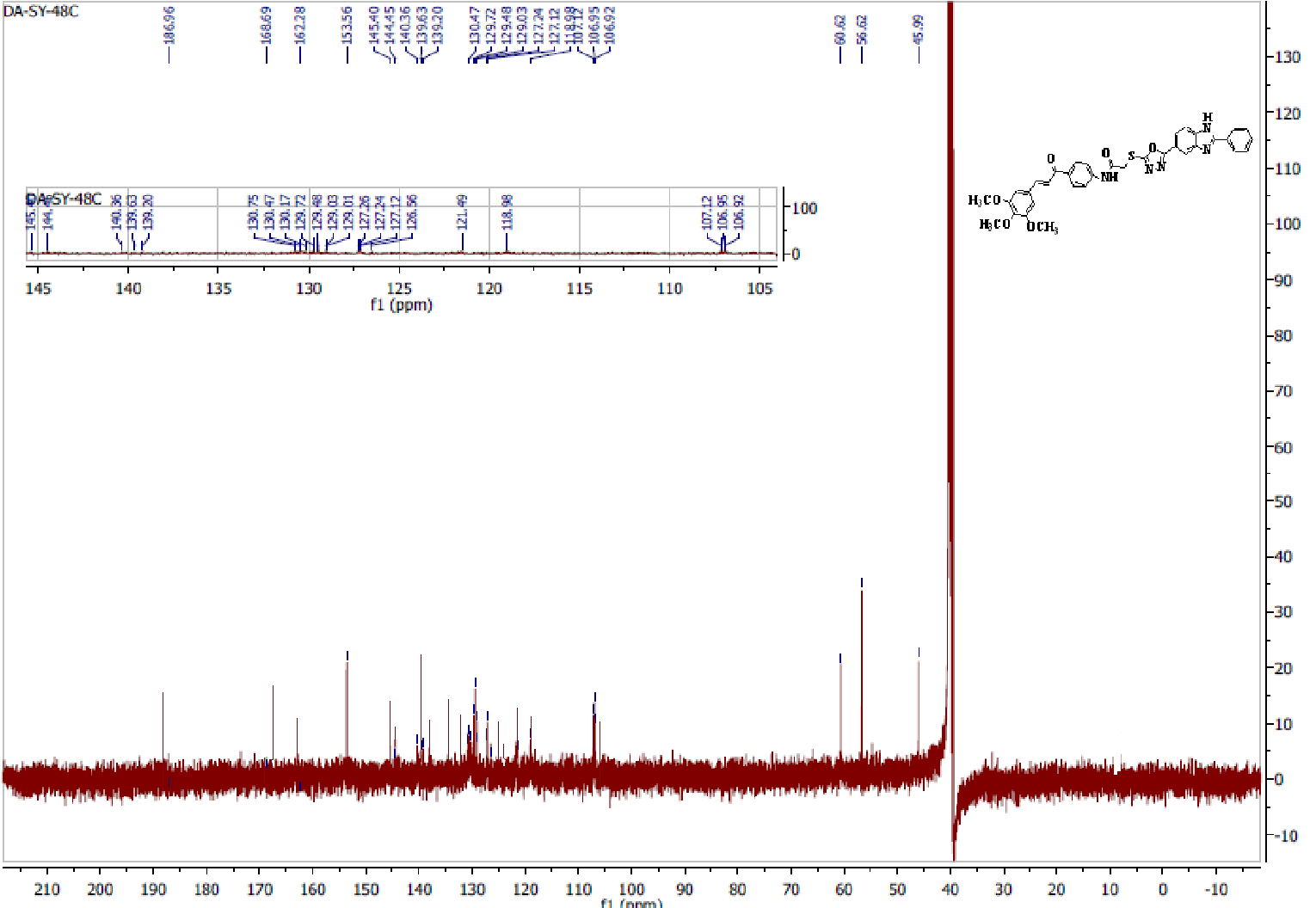
**

**Fig. S 14. ^13^C NMR of compound 7e (125 MHz, DMSO-*d_6_*).**

**
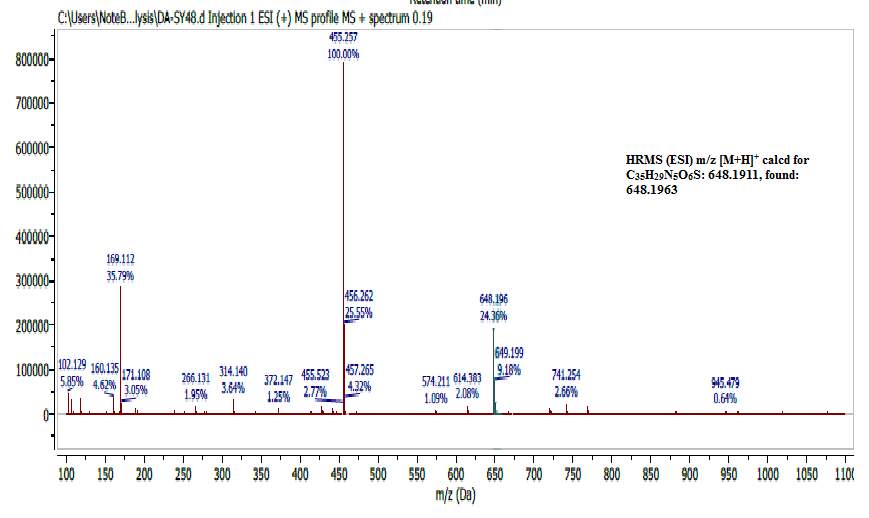
**

**Fig. S 15:** **HRMS (ESI) spectrum of compound** **7e.**

**
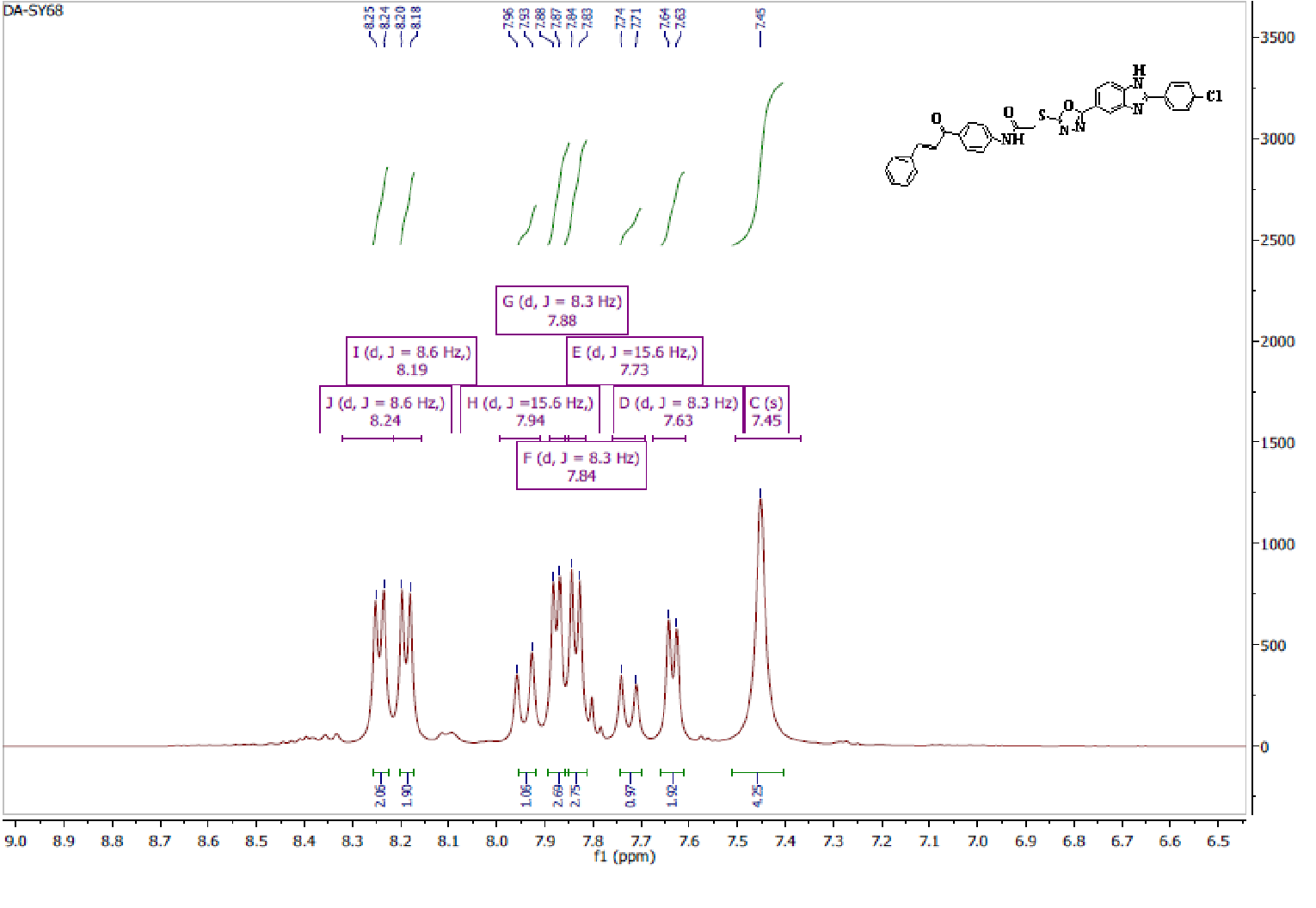
**

**
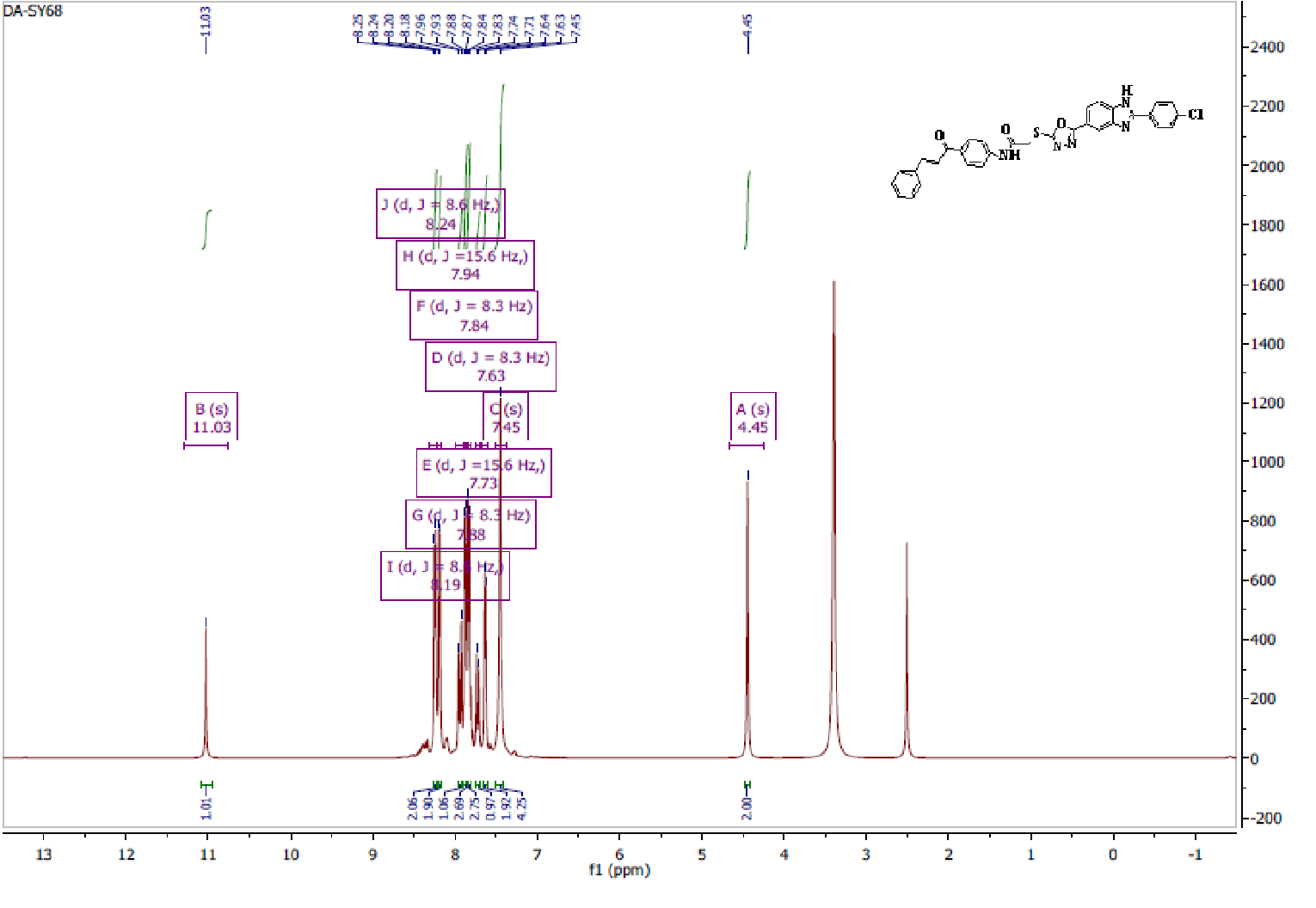
**

**Fig. S 16. ^1^H NMR of compound 7f (500 MHz, DMSO-*d_6_*)**

**
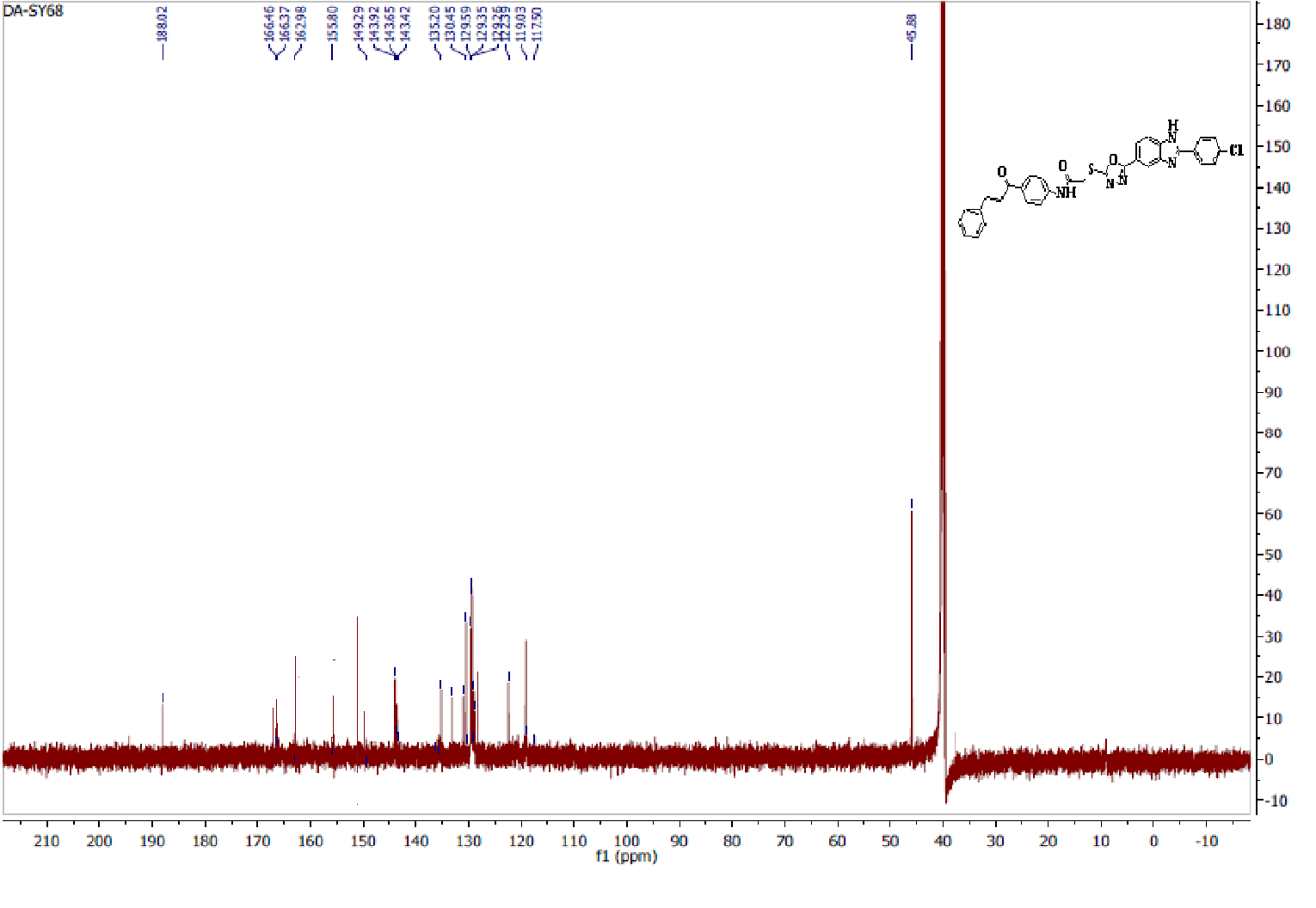
**

**Fig. S 17. ^13^C NMR of compound 7f (125 MHz, DMSO-*d_6_*)**

**
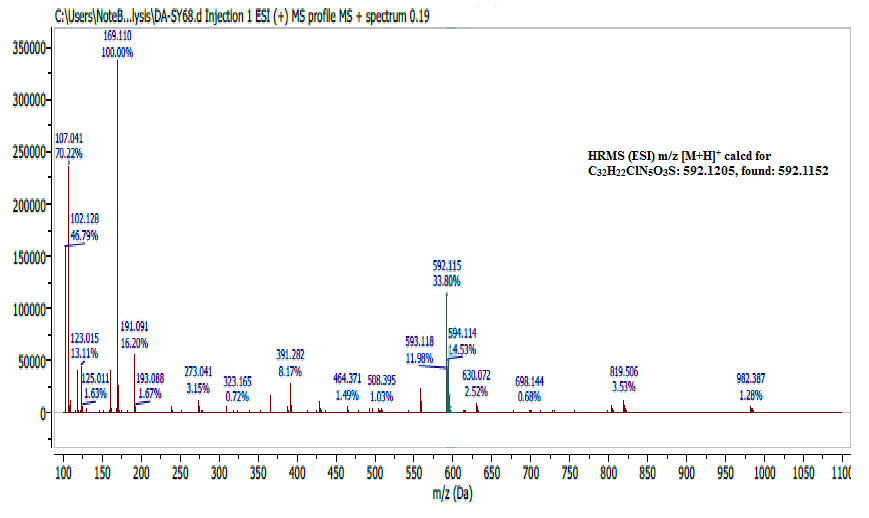
**

**Fig. S 18:** **HRMS (ESI) spectrum of compound** **7f.**

**
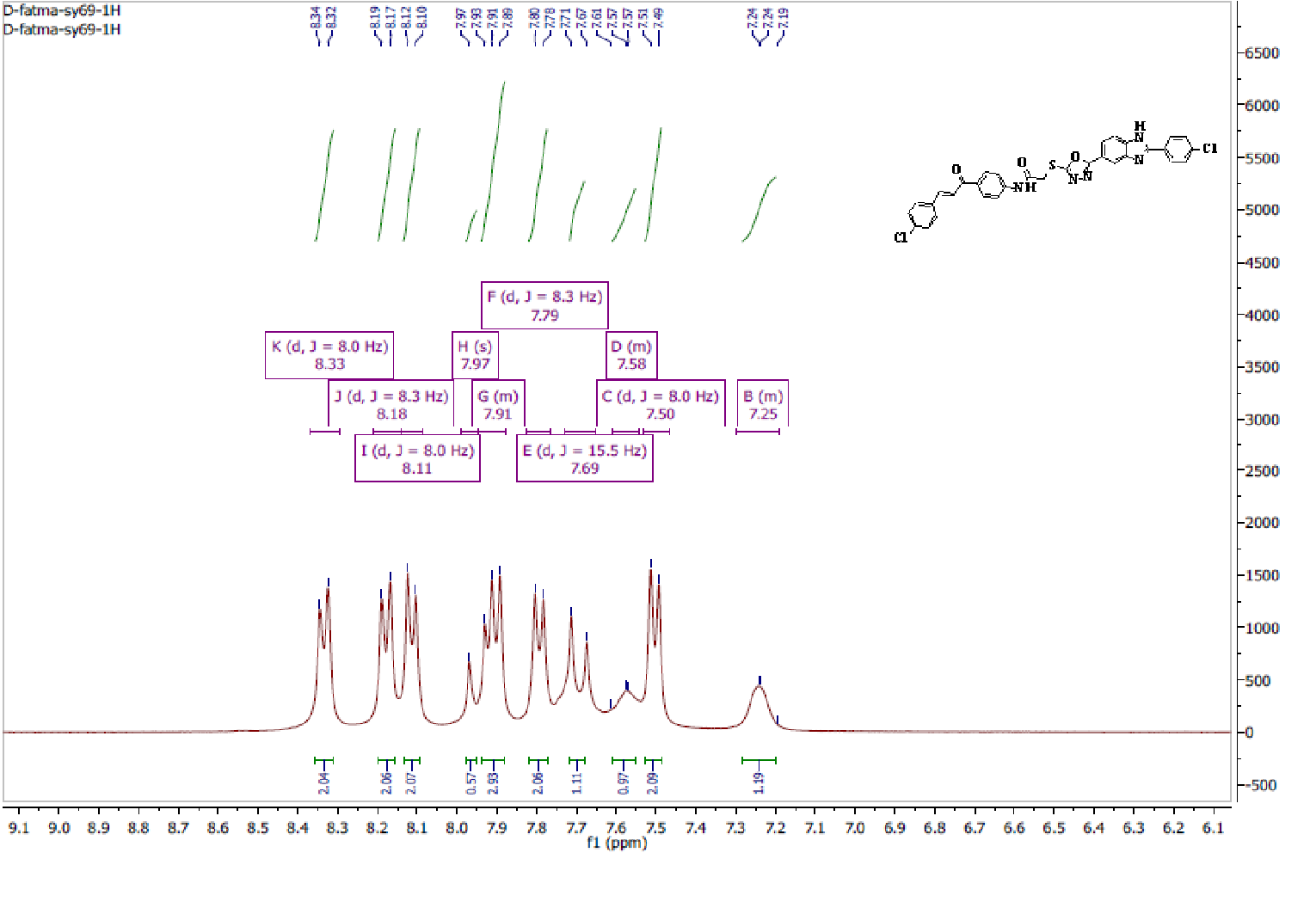
**

**
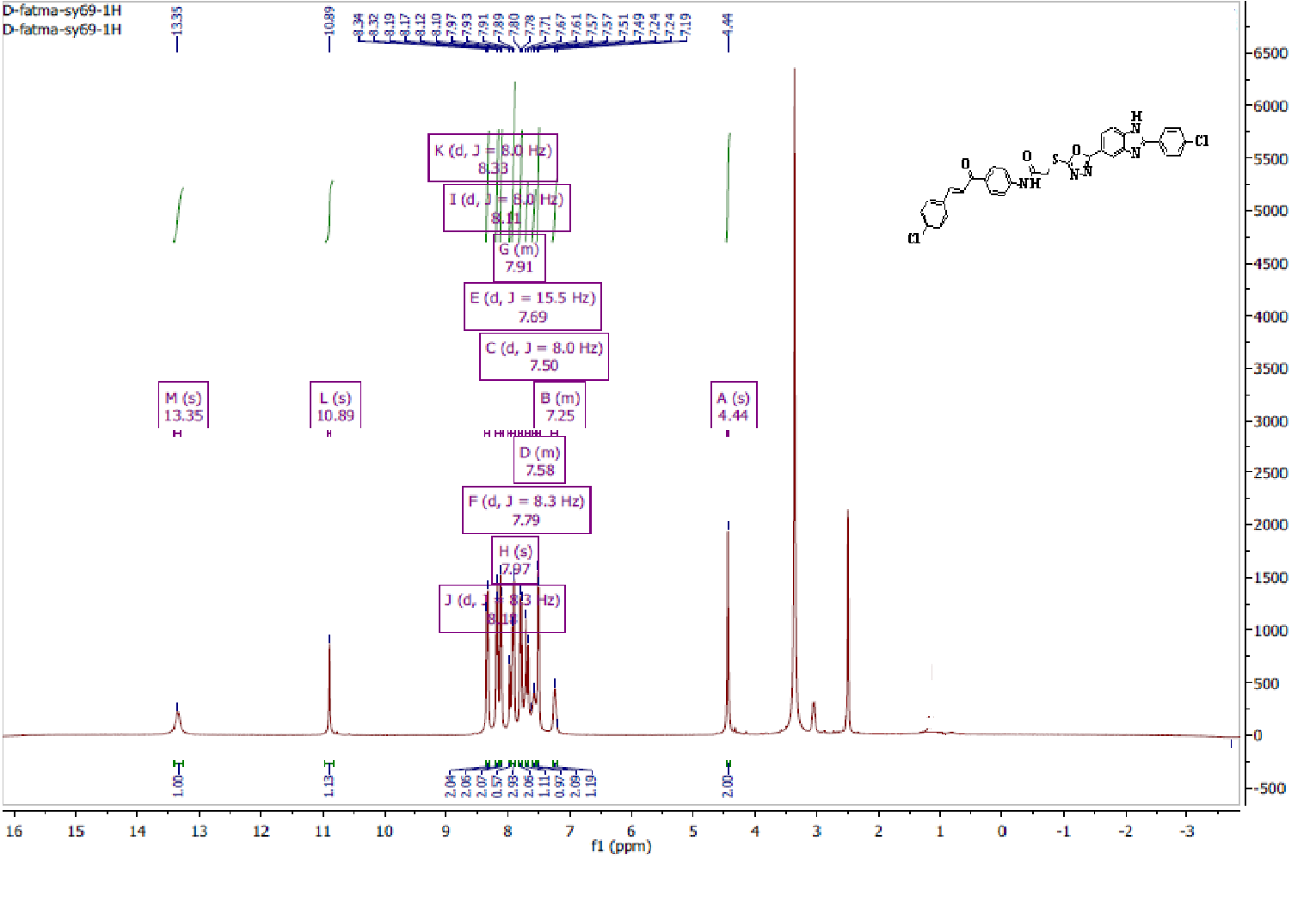
**

**Fig. S 19. ^1^H NMR of compound 7g (500 MHz, DMSO-*d_6_*)**

**
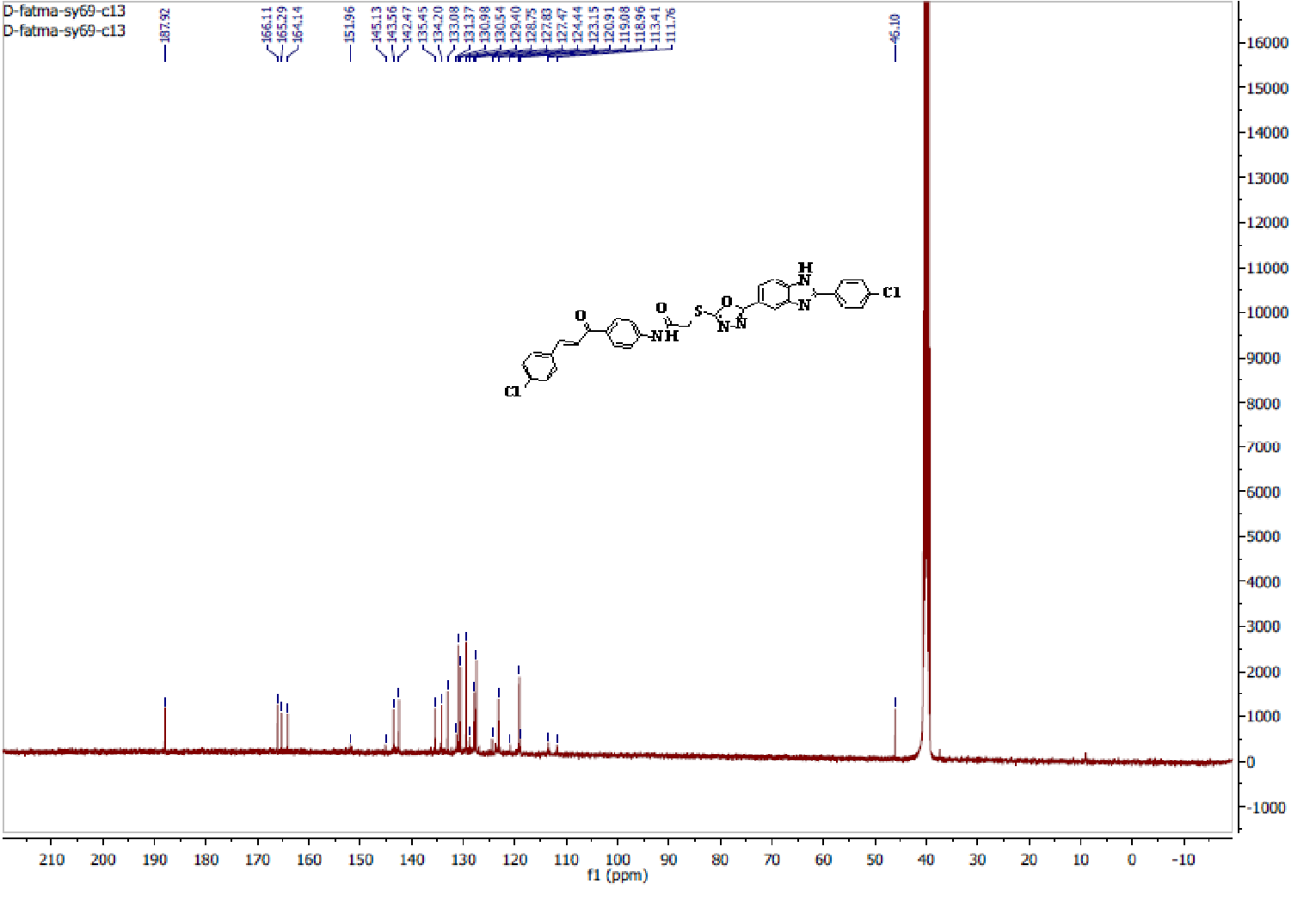
**

**Fig. S 20. ^13^C NMR of compound 7g (125 MHz, DMSO-*d_6_*)**

**
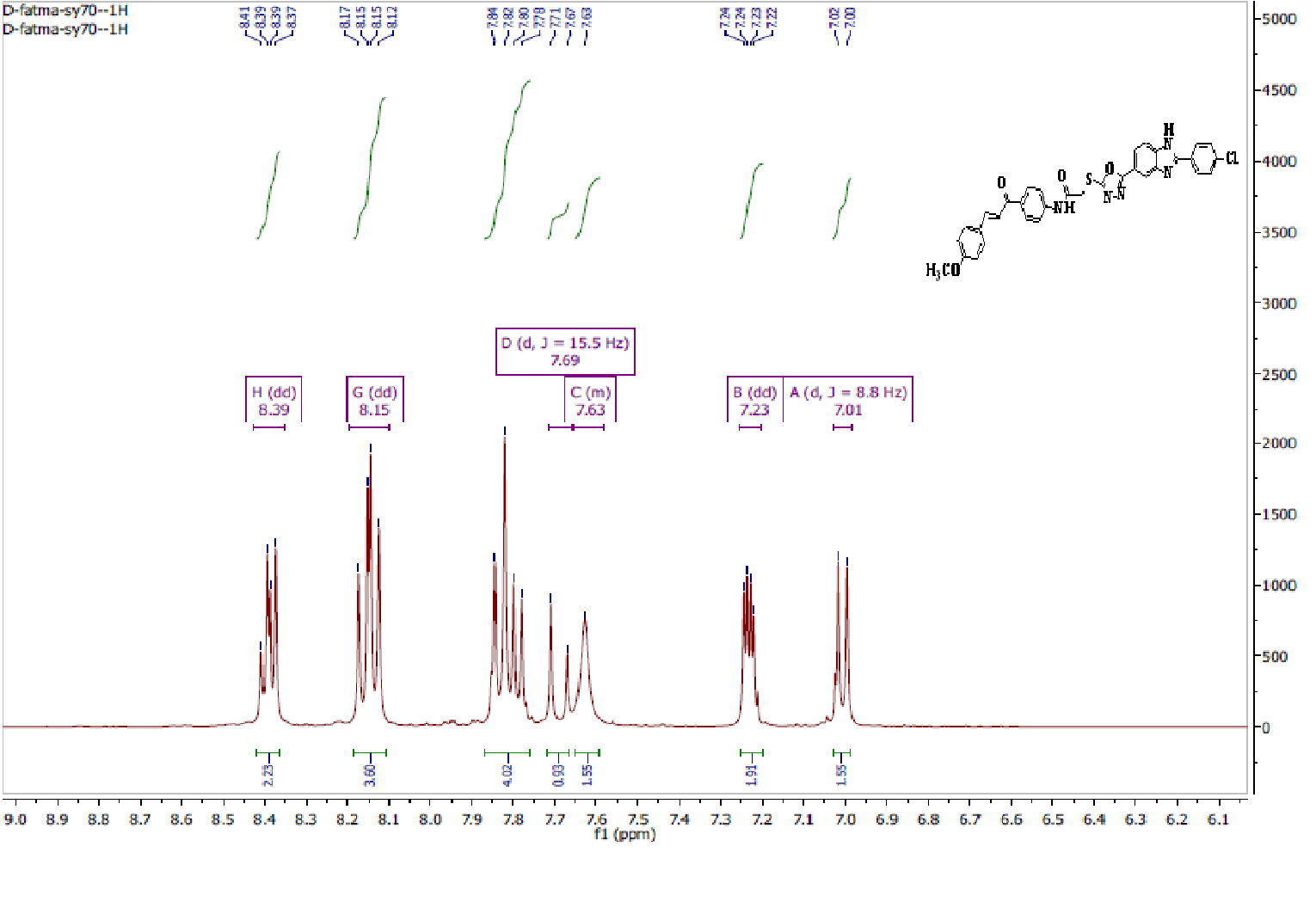
**

**
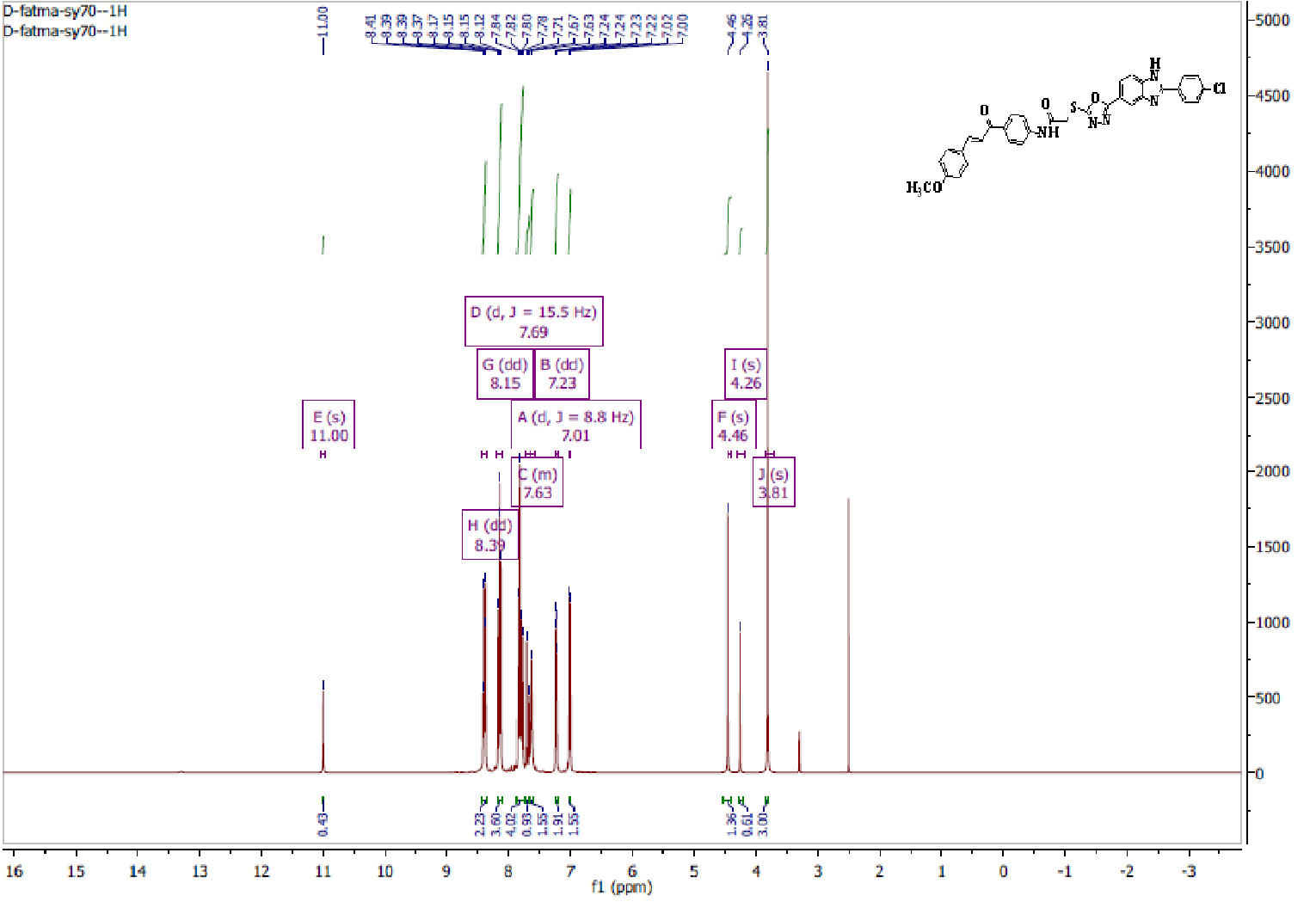
**

**Fig. S 21. ^1^H NMR of compound 7h (500 MHz, DMSO-*d_6_*)**

**
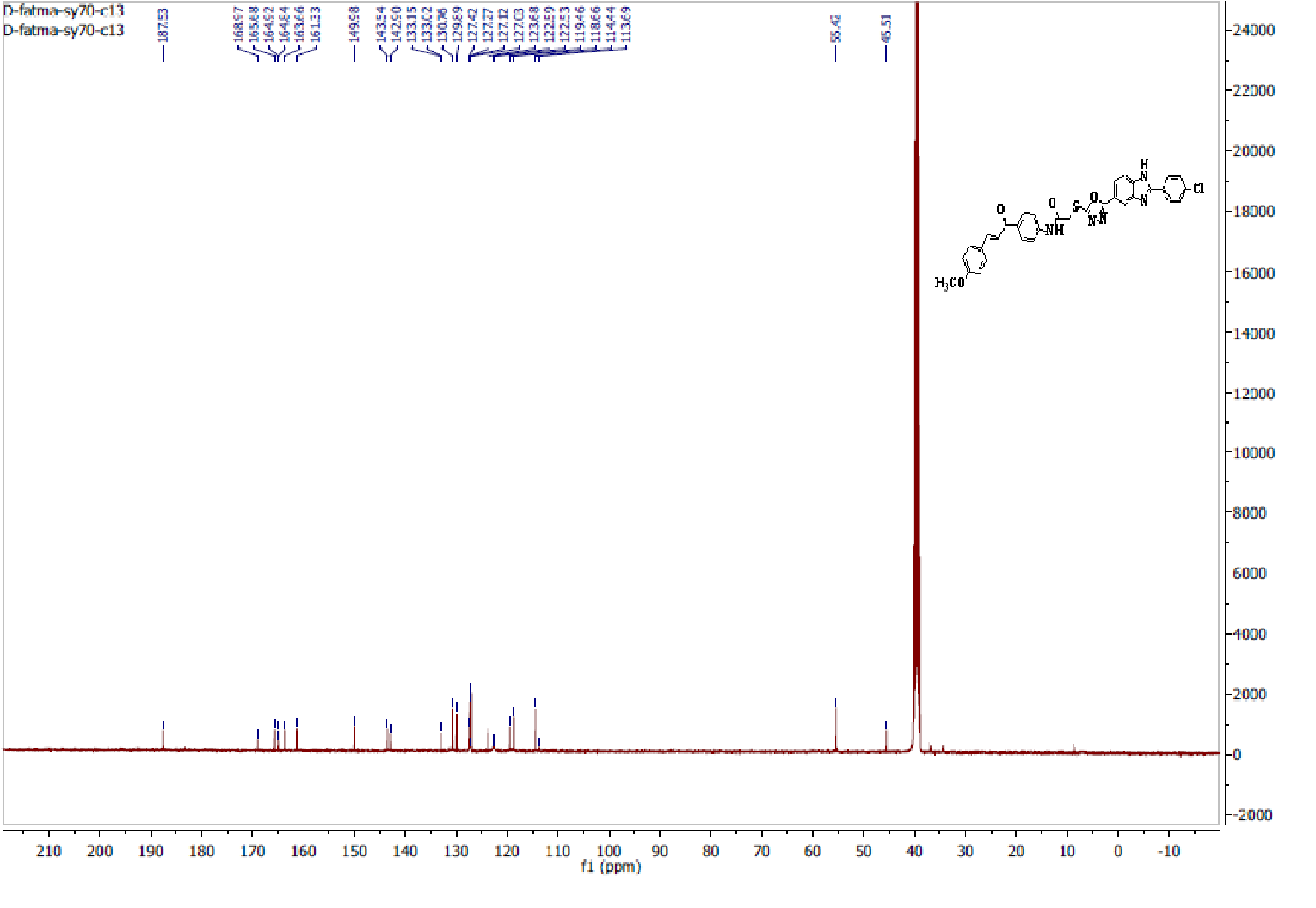
**

**Fig.S22. ^13^C NMR of compound 7h (125 MHz, DMSO-*d_6_*)**

**.**

**
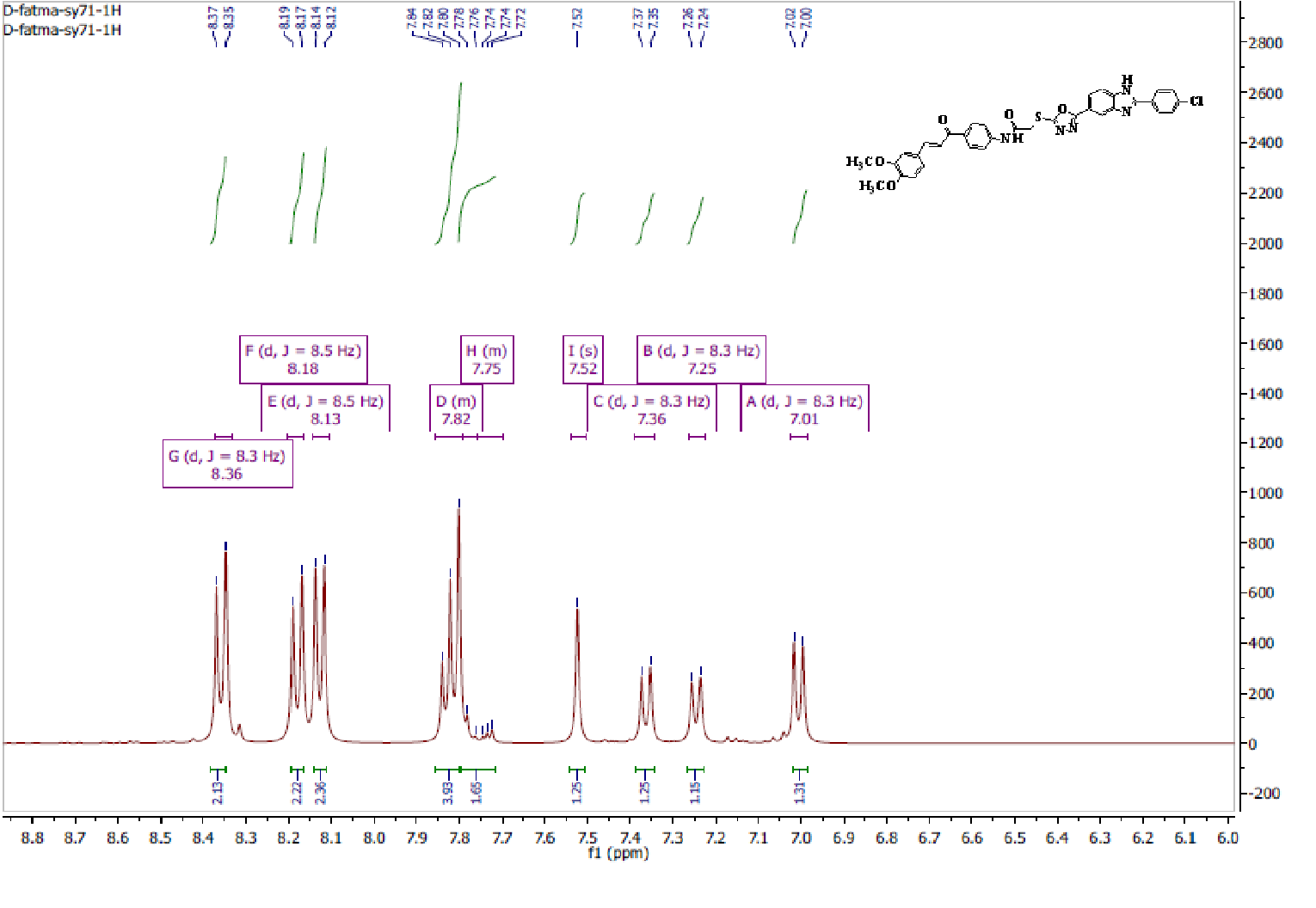
**

**
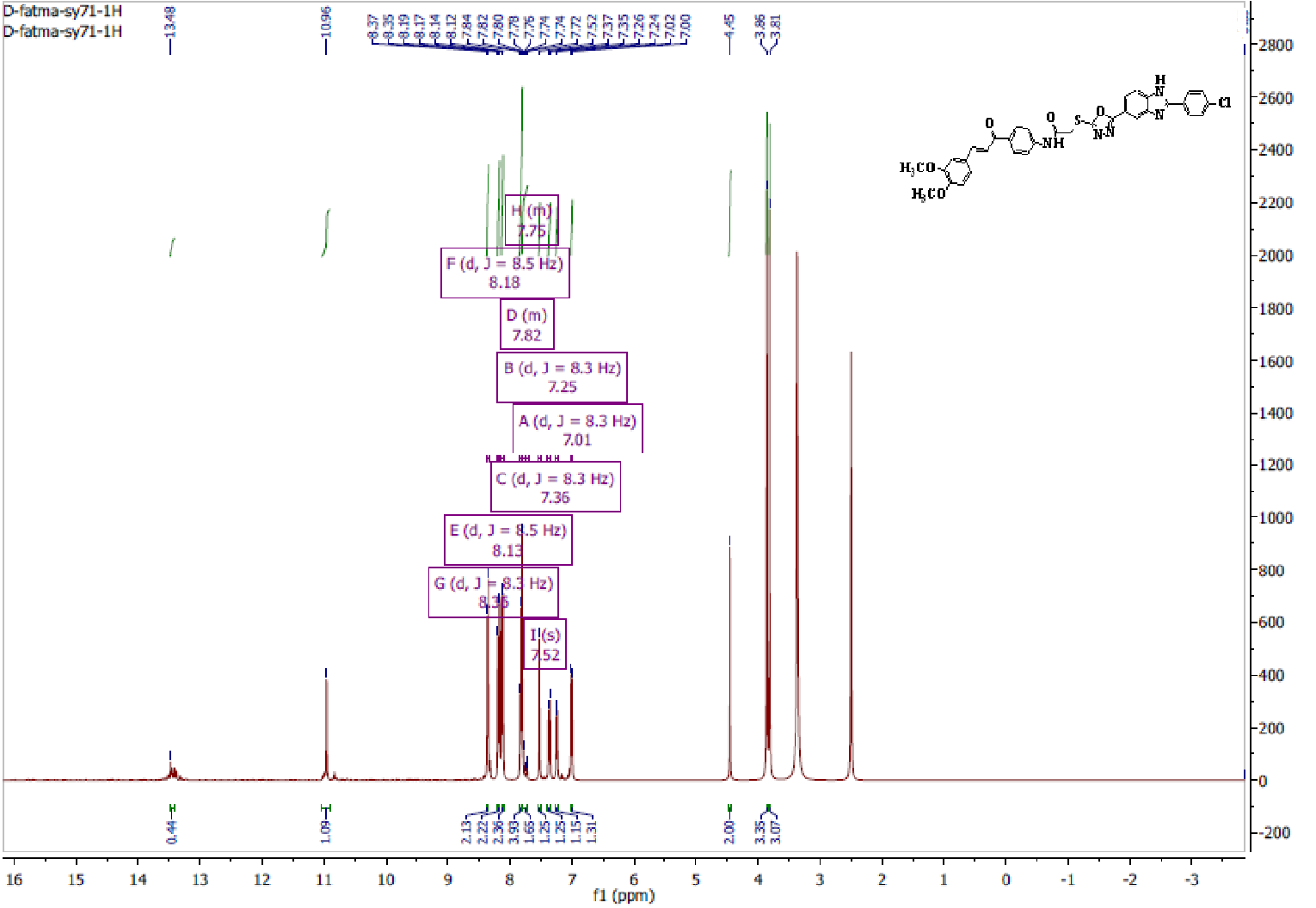
**

**Fig.S23. ^1^H NMR of compound 7i (500 MHz, DMSO-*d_6_*)**

**
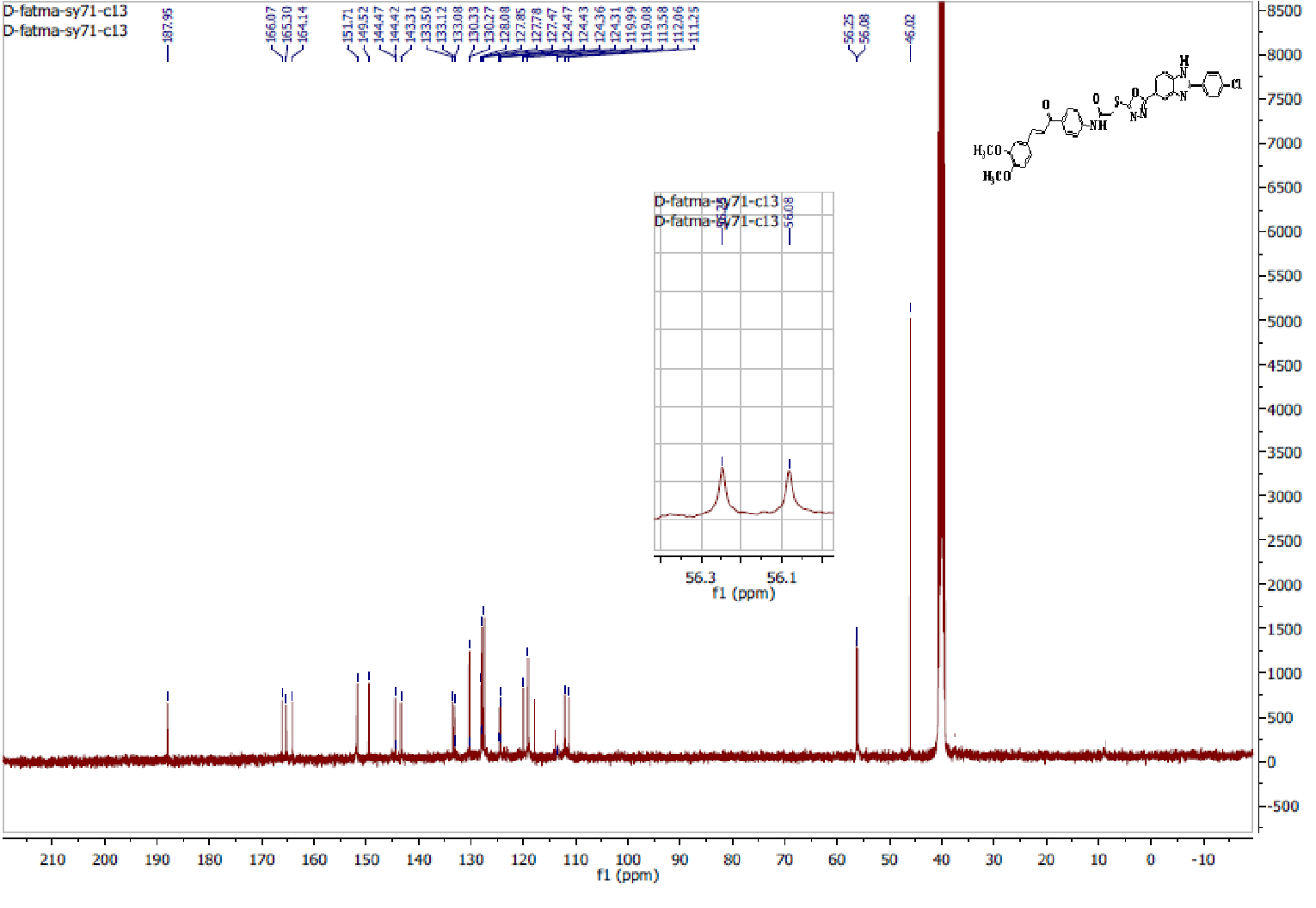
**

**Fig.S24. ^13^C NMR of compound 7i (125 MHz, DMSO-*d_6_*)**

**
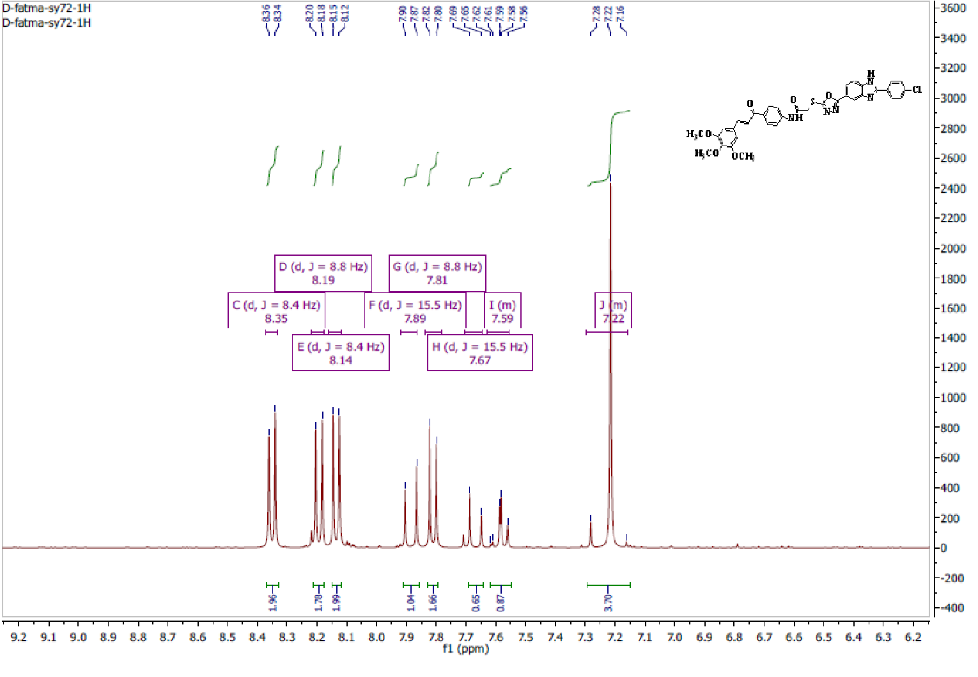
**

**
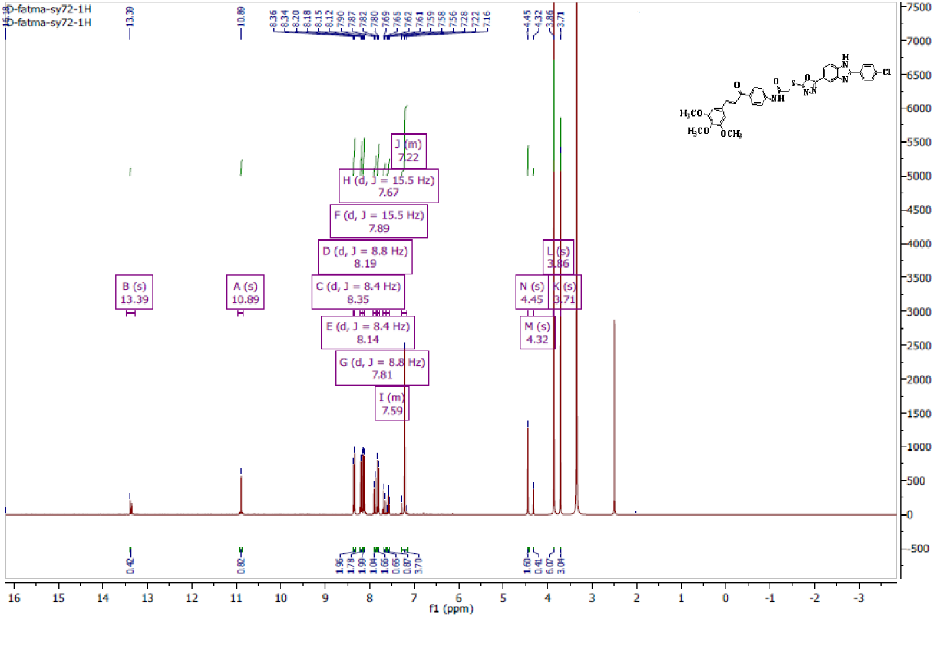
**

**Fig.S25. ^1^HNMR of compound 7j (500 MHz, DMSO-*d_6_*)**

**
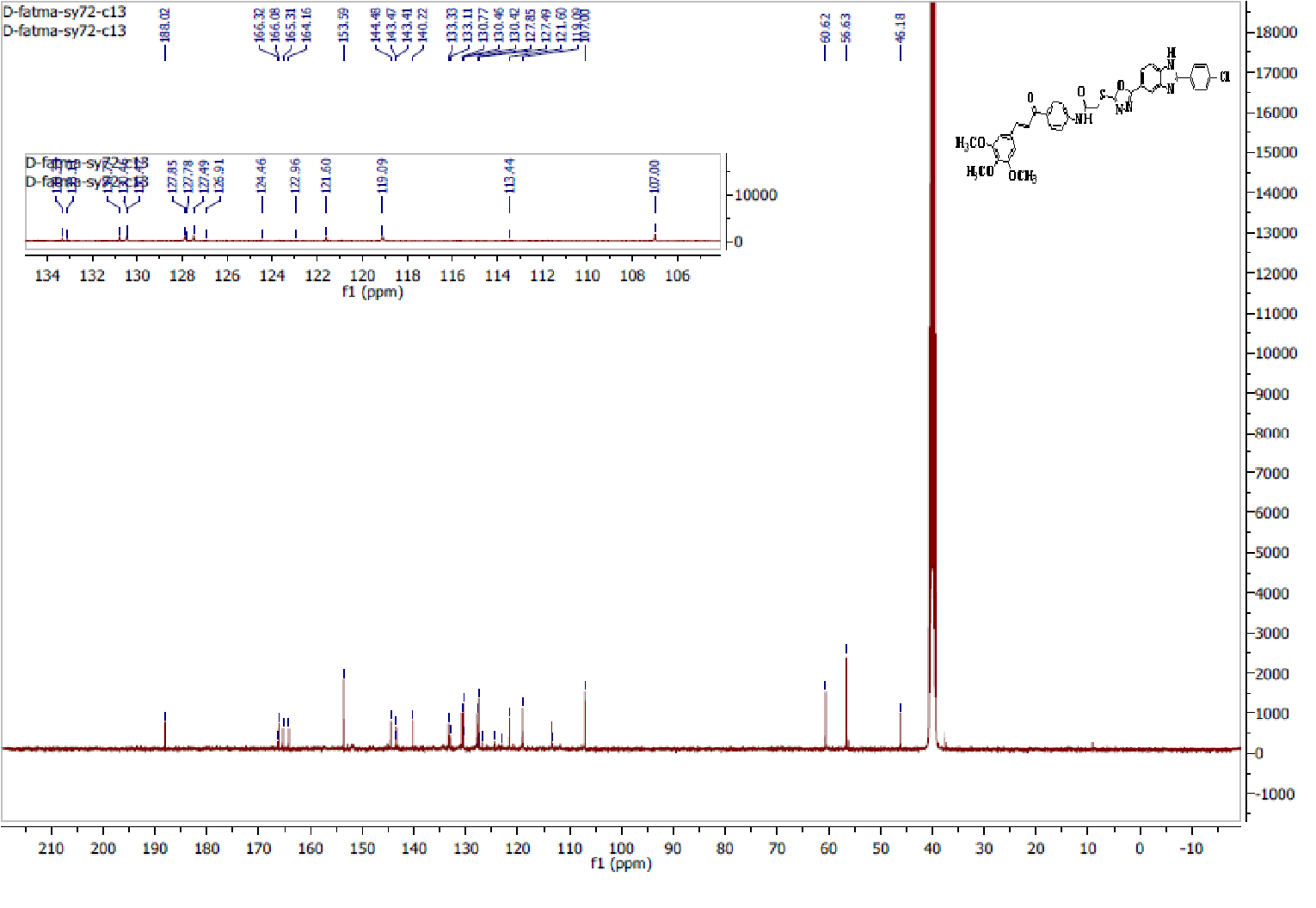
**

**Fig.S26. ^13^C NMR of compound 7j (125 MHz, DMSO-*d_6_*)**

**
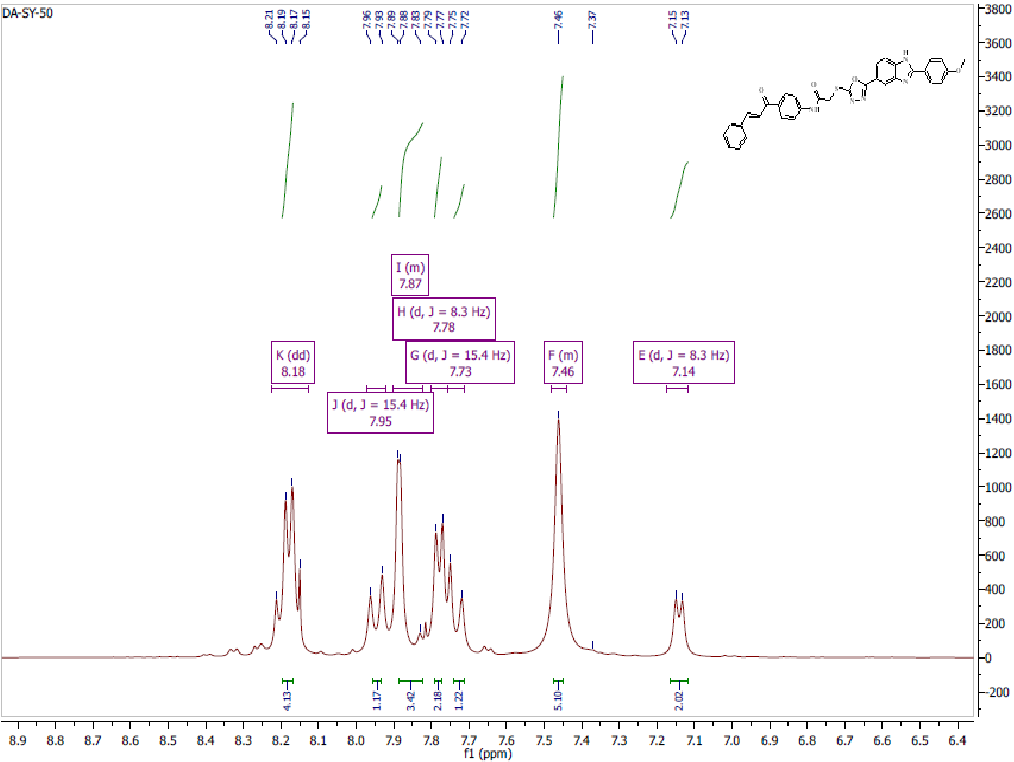
**

**
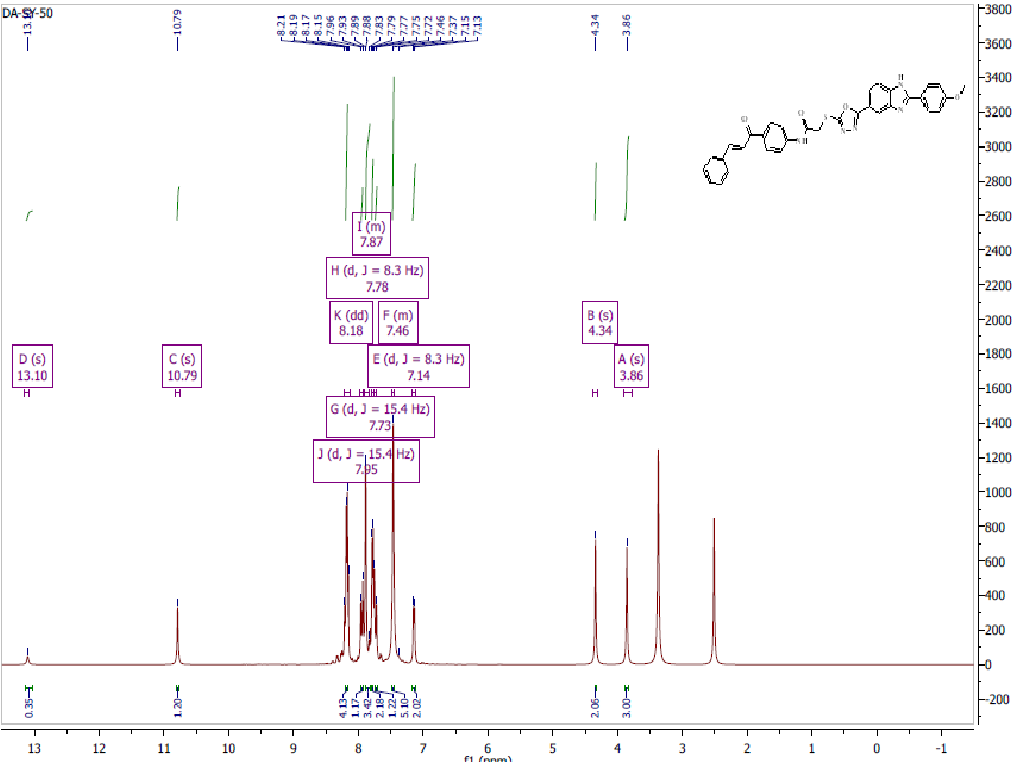
**

**Fig.S27. ^1^H NMR of compound 7k (500 MHz, DMSO-*d_6_*)**

**
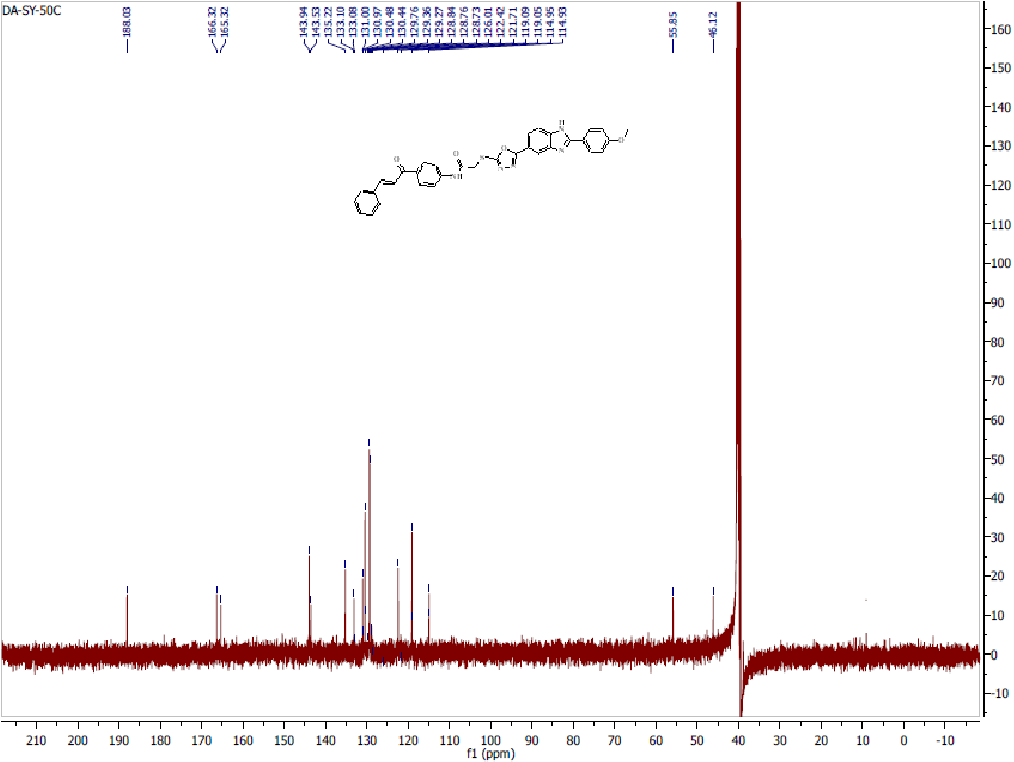
**

**Fig.S28. ^13^C NMR of compound 7k (125 MHz, DMSO-*d_6_*)**

**
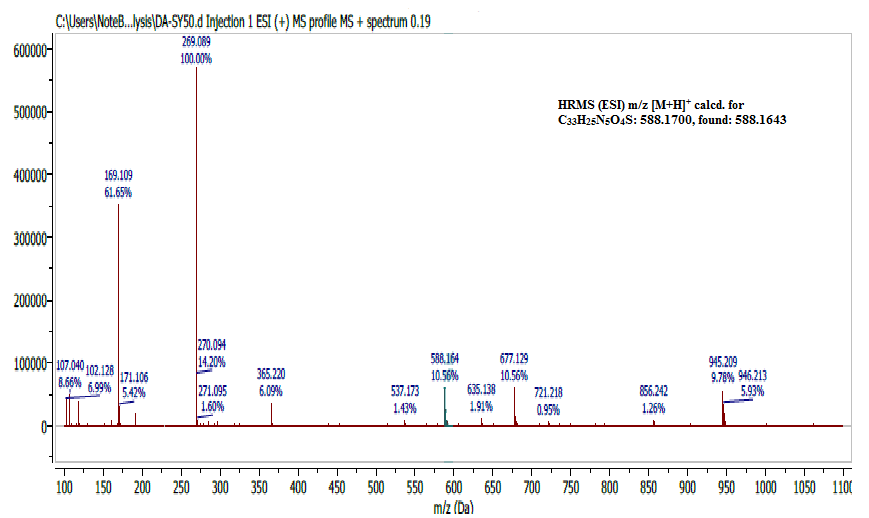
**

**Fig.S29.** HRMS **(ESI) spectrum of compound** **7k.**

**
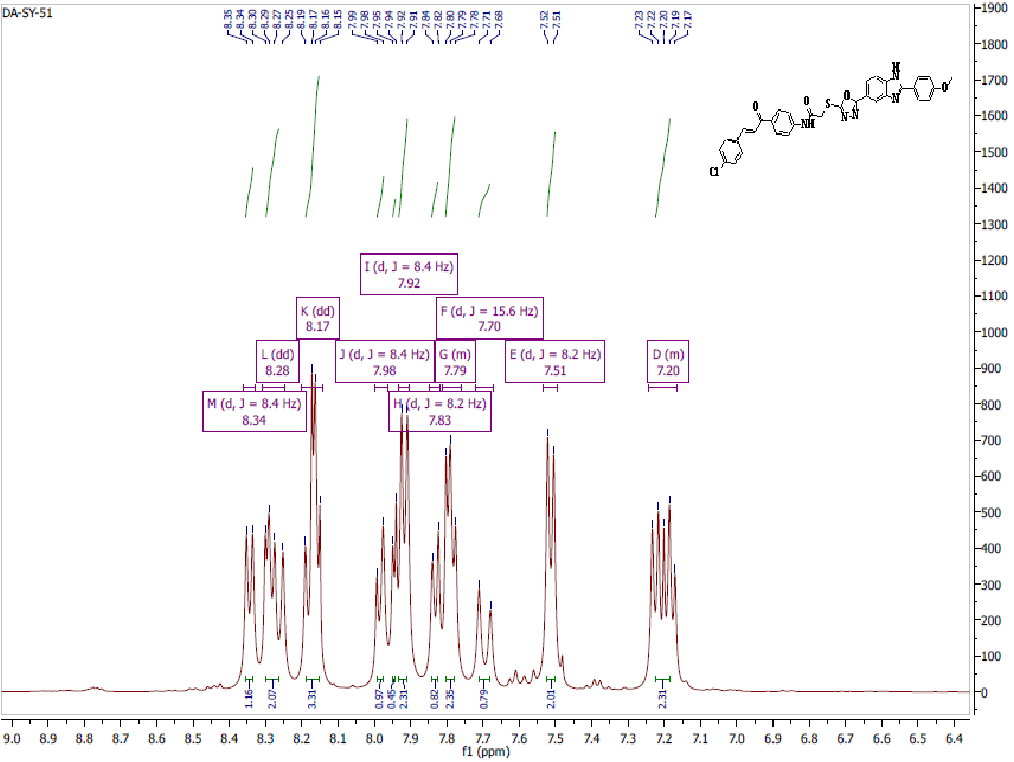
**

**
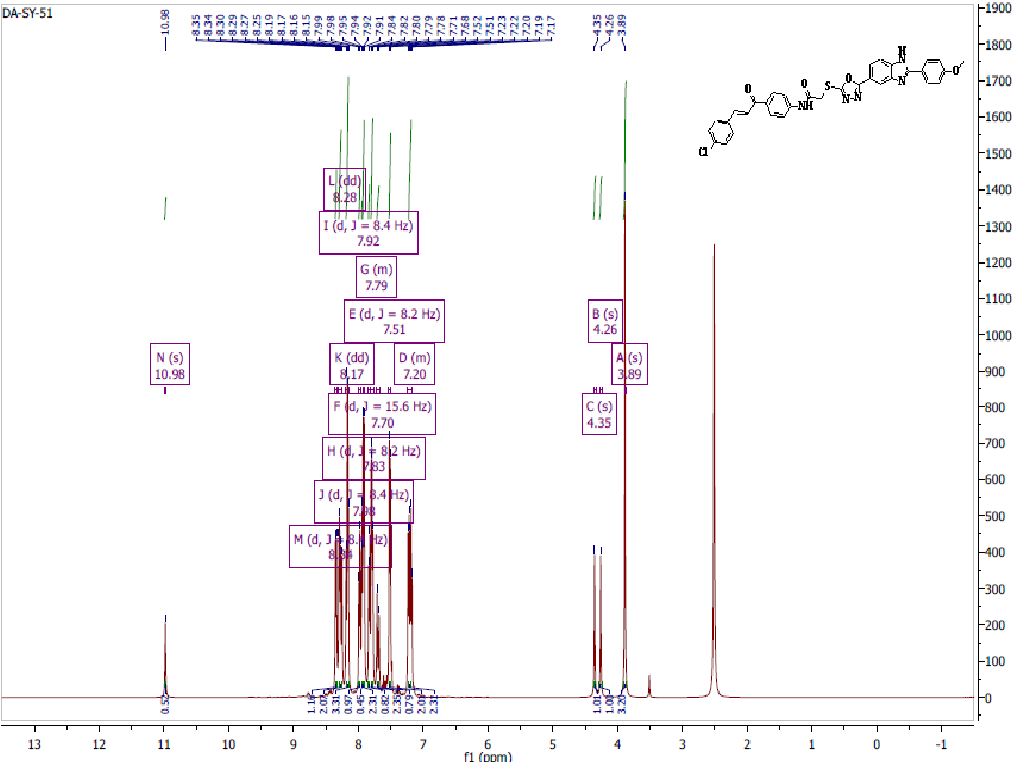
**

**Fig.S30. ^1^H NMR of compound 7l (400 MHz, DMSO-*d_6_*)**

**
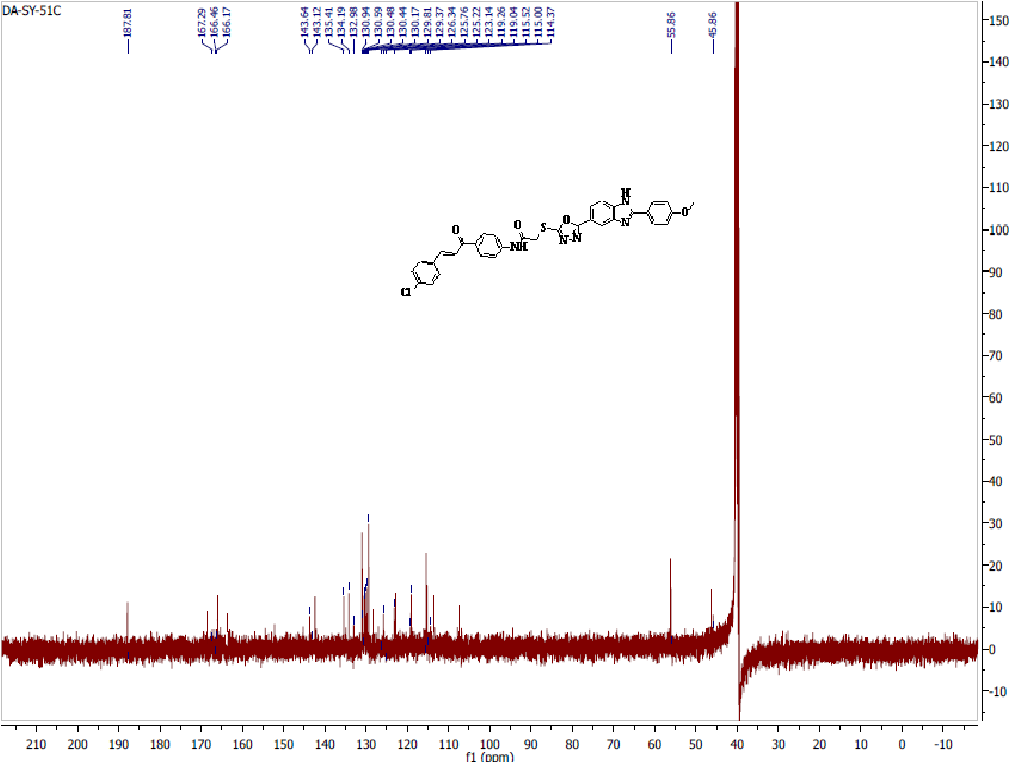
**

**Fig.S31. ^13^C NMR of compound 7l (125 MHz, DMSO-*d_6_*)**

**
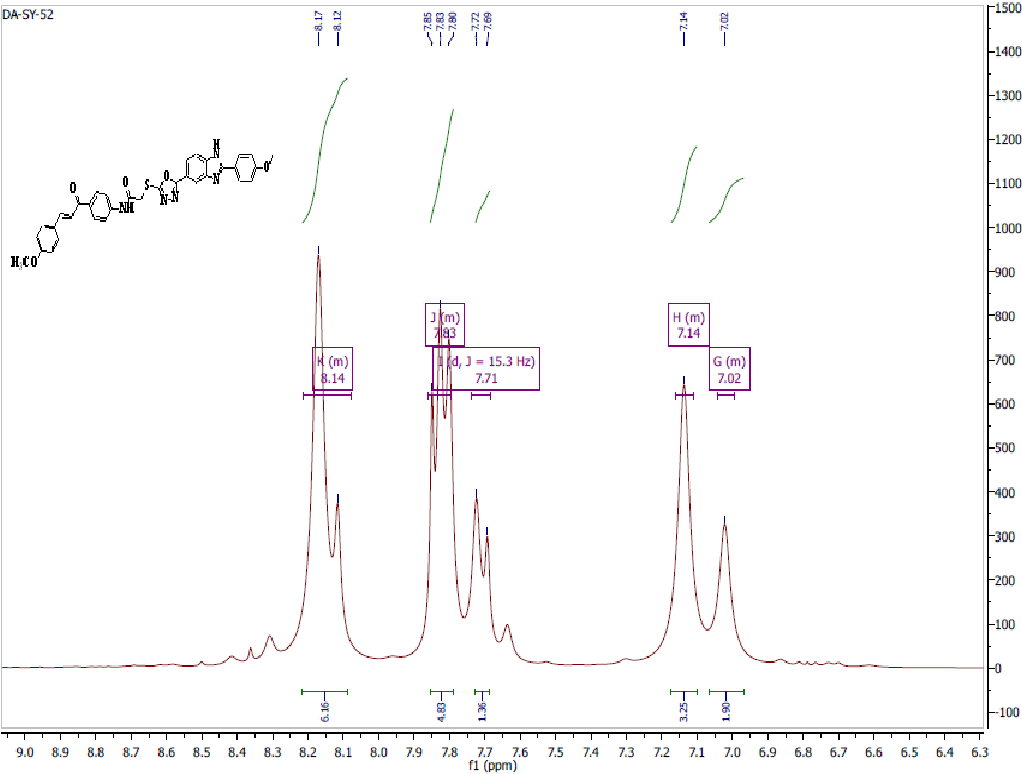
**

**
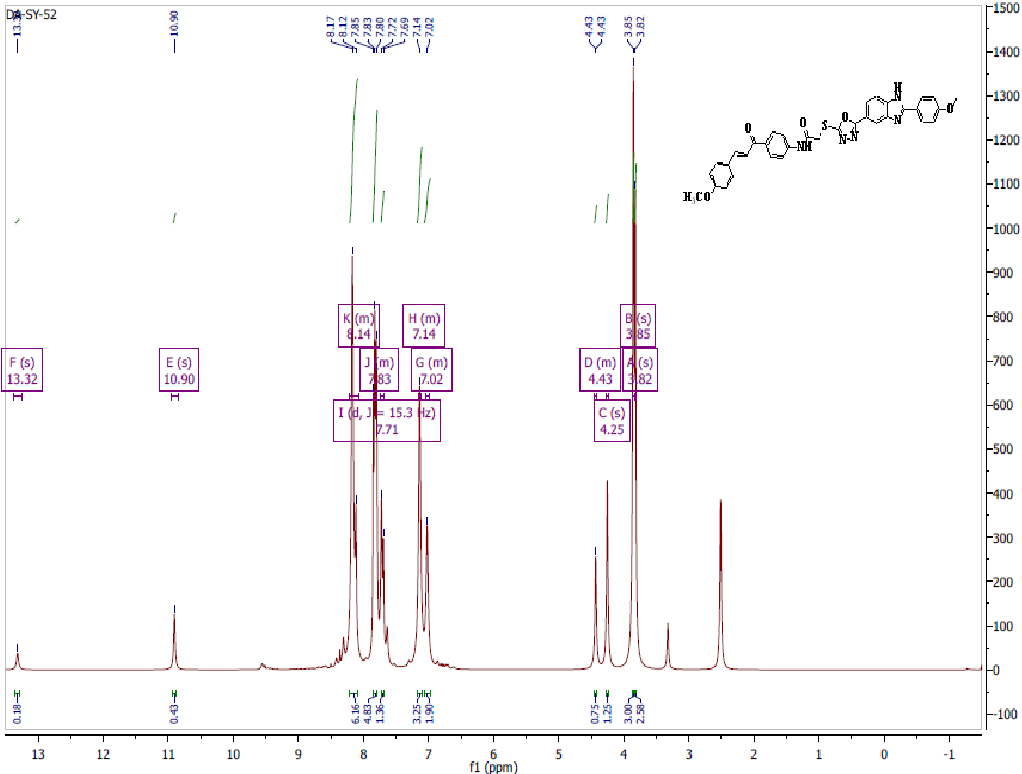
**

**Fig.S32. ^1^H NMR of compound7m (500 MHz, DMSO-*d_6_*)**

**
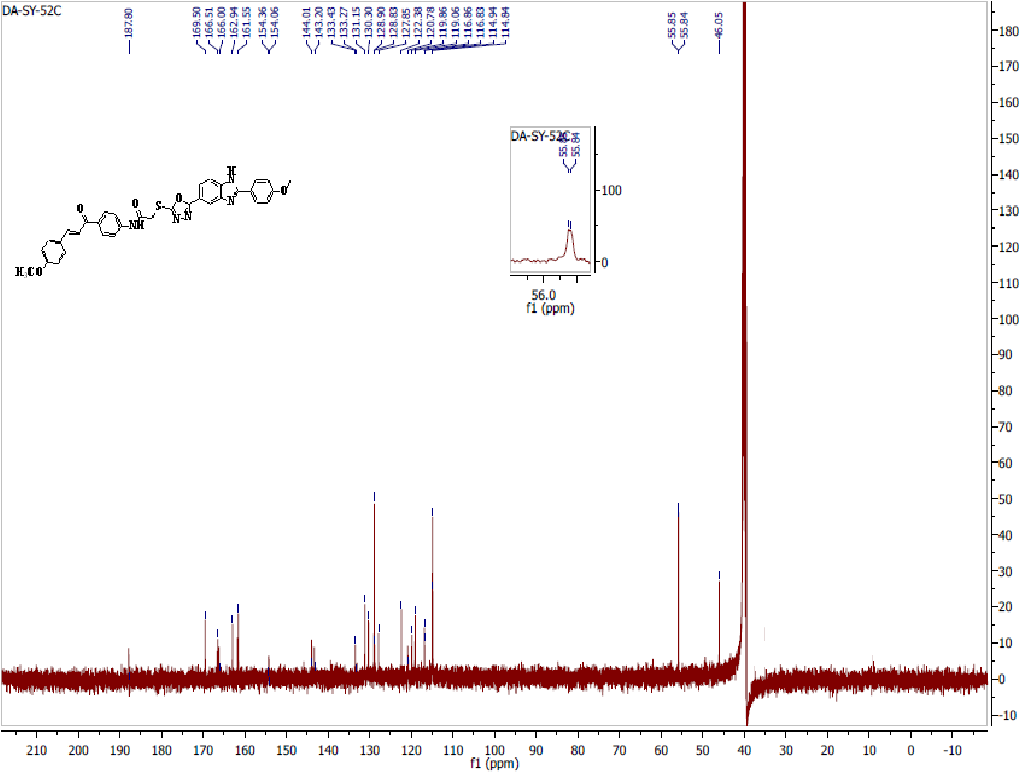
**

**Fig.S33. ^13^C NMR of compound 7m (125 MHz, DMSO-*d_6_*)**

**
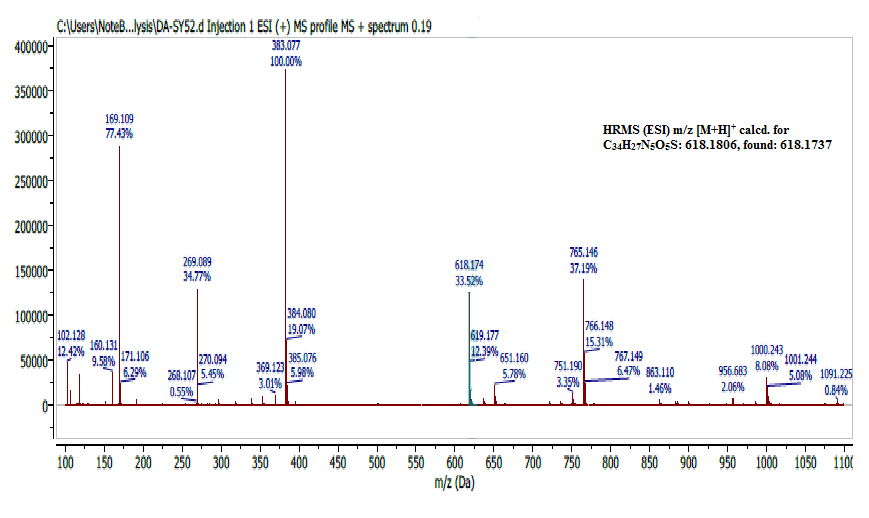
**

**Fig.S34. HRMS (ESI) spectrum of compound** **7m.**

**
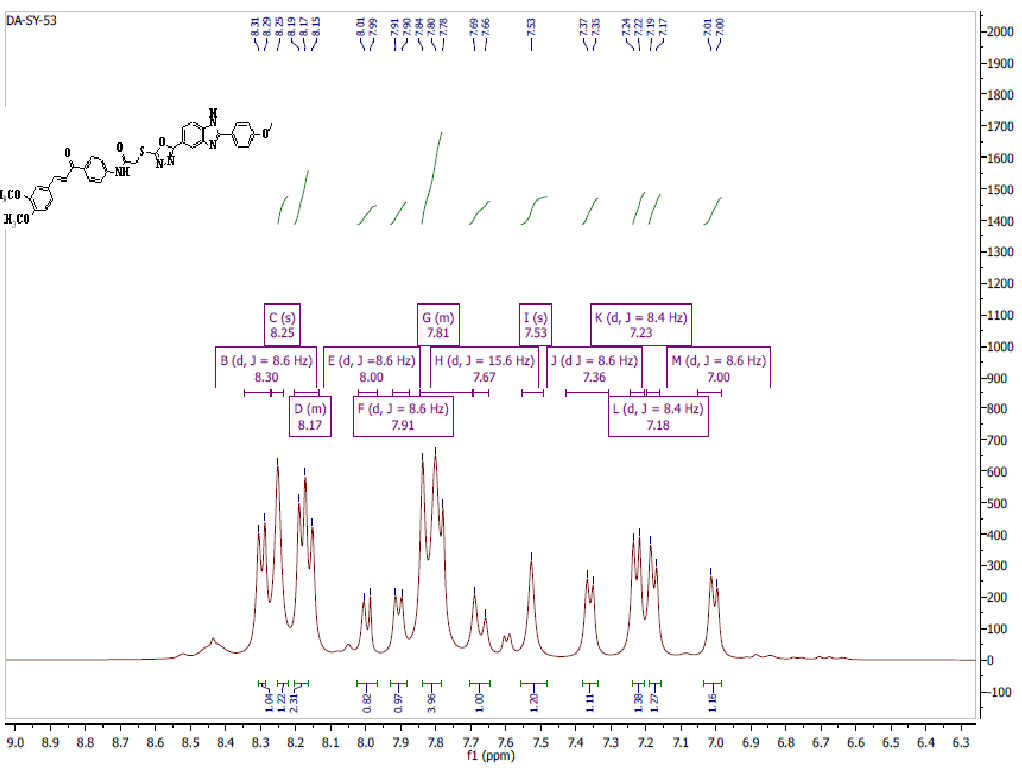
**

**
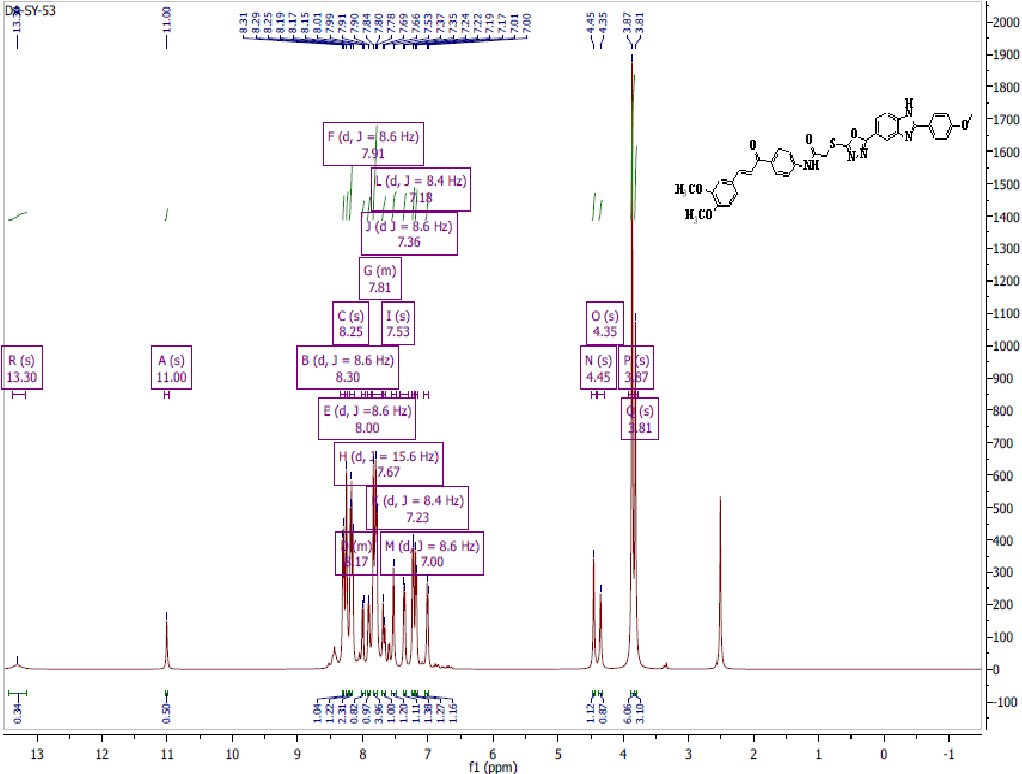
**

**Fig.S35. ^1^H NMR of compound 7n (500 MHz, DMSO-*d_6_*)**

**
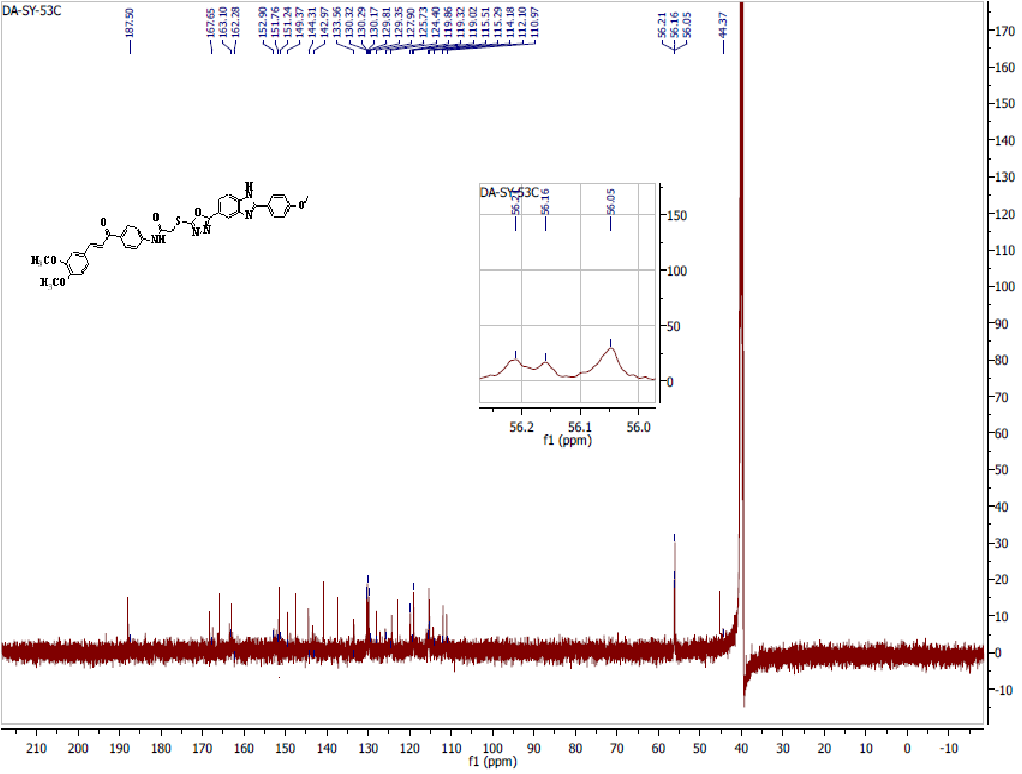
**

**Fig.S36. ^13^C NMR of compound 7n (125 MHz, DMSO-*d_6_*)**

**
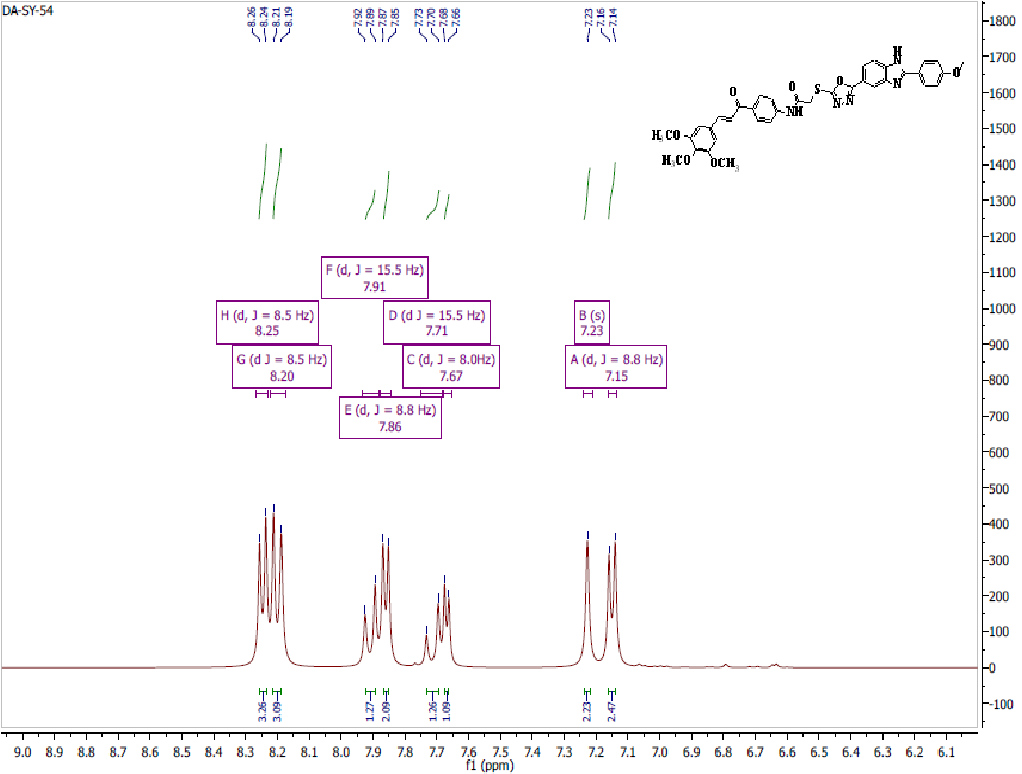
**

**
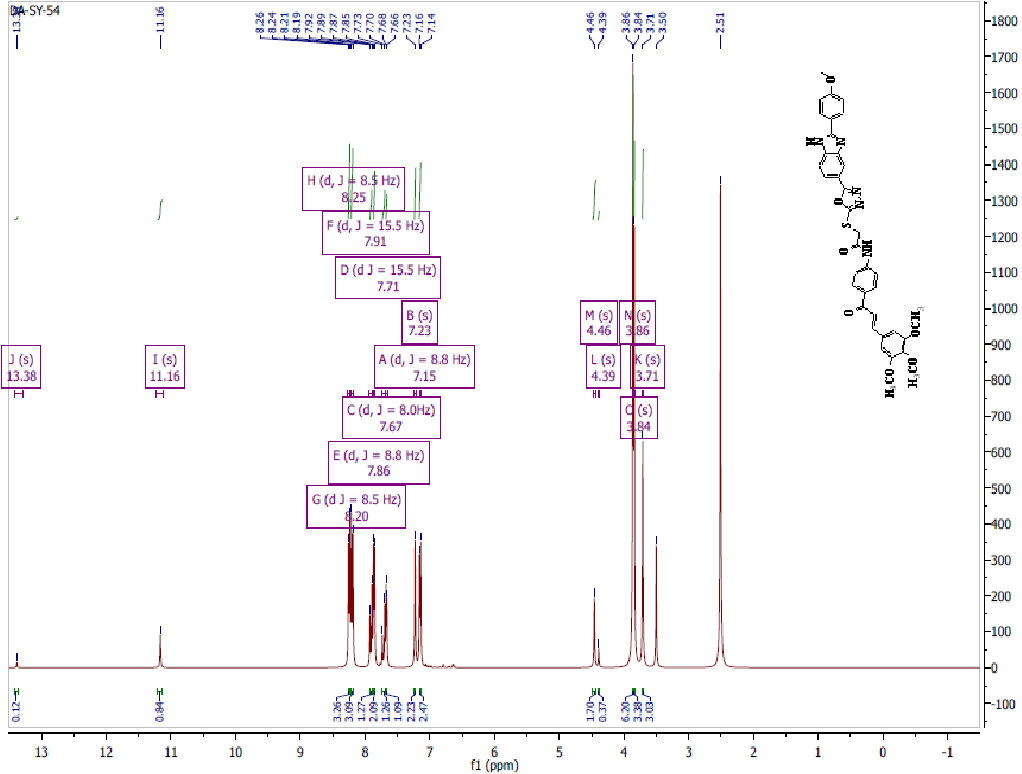
**

**Fig.S37. ^1^H NMR of compound 7o (500 MHz, DMSO-*d_6_*)**

**
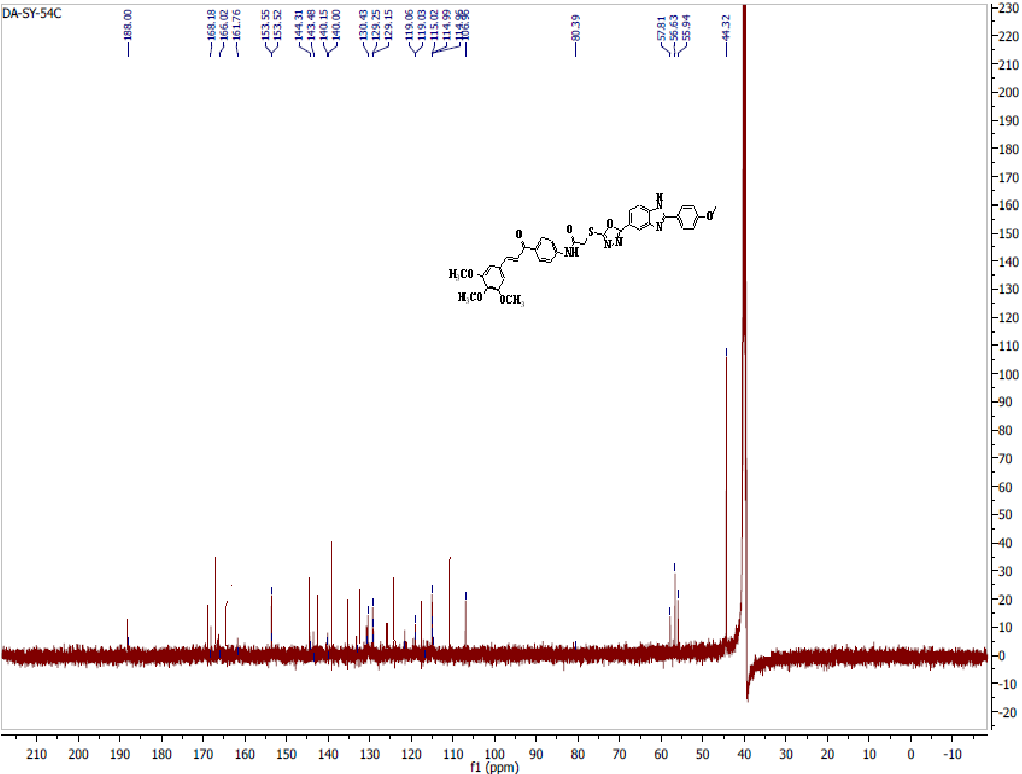
**

**Fig38. ^13^C NMR of compound 7o (125 MHz, DMSO-*d_6_*)**

**
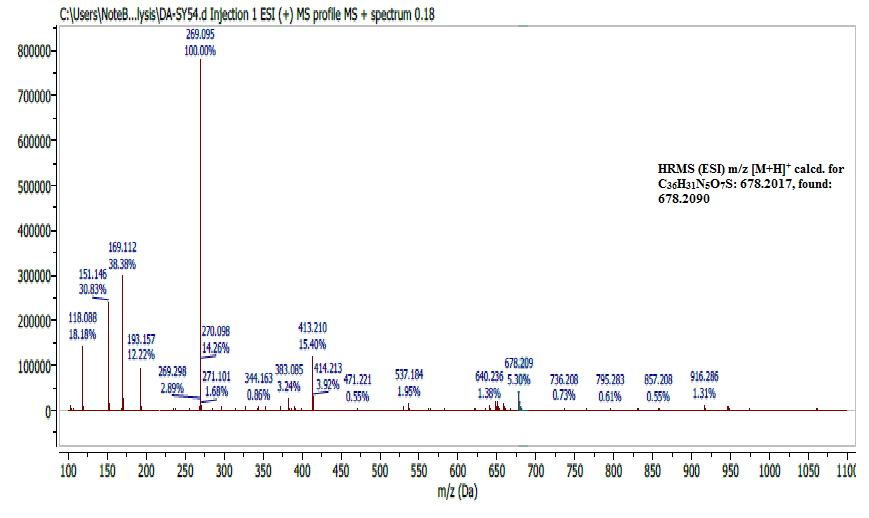
**

**Fig.S39. HRMS (ESI) spectrum of compound** **7o.**

**
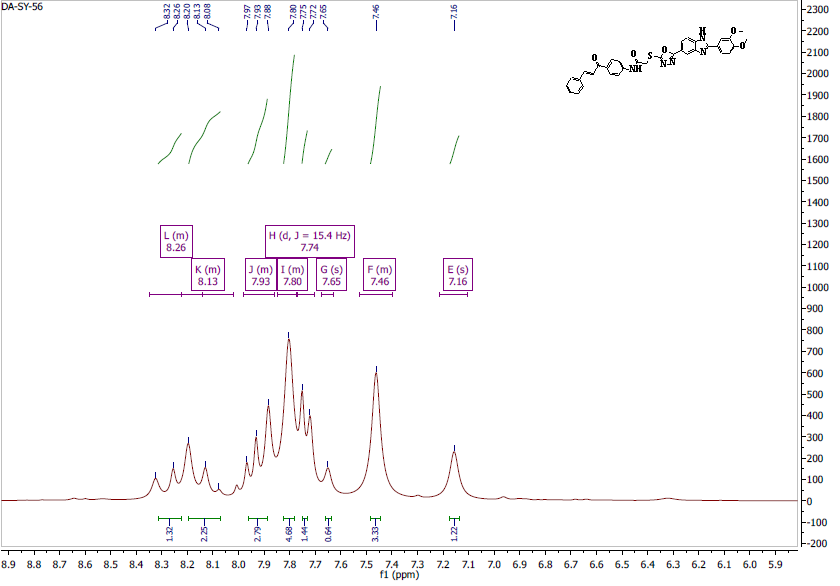
**

**
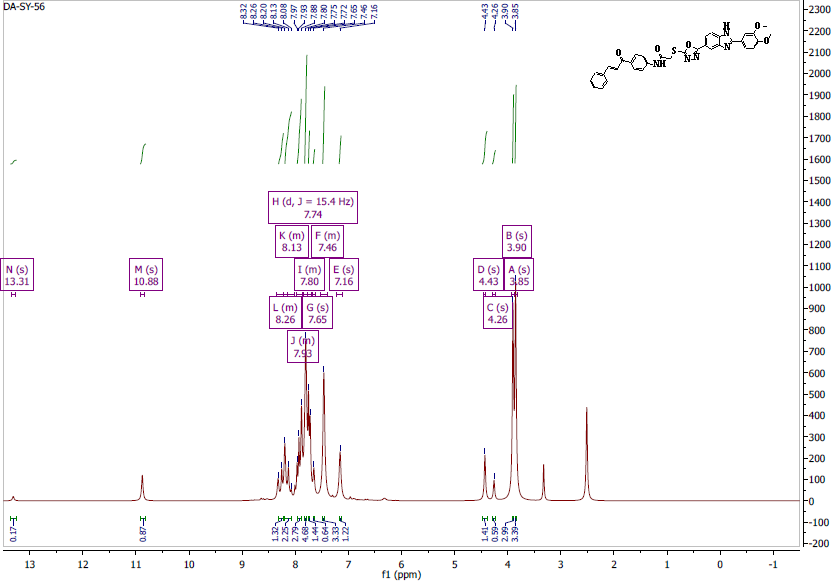
**

**Fig.S40. ^1^H NMR of compound 7p (500 MHz, DMSO-*d_6_*)**

**
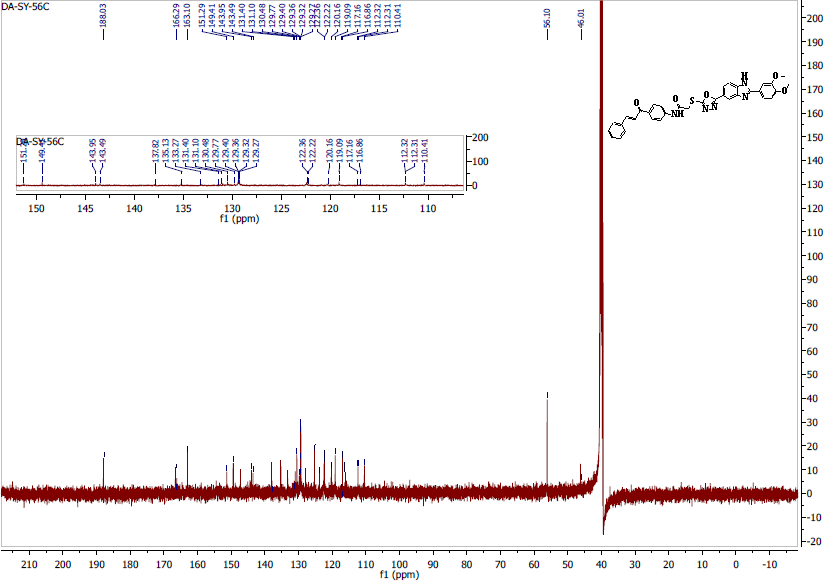
**

**Fig.S41. ^13^C NMR of compound 7p (125 MHz, DMSO-*d_6_*)**

**
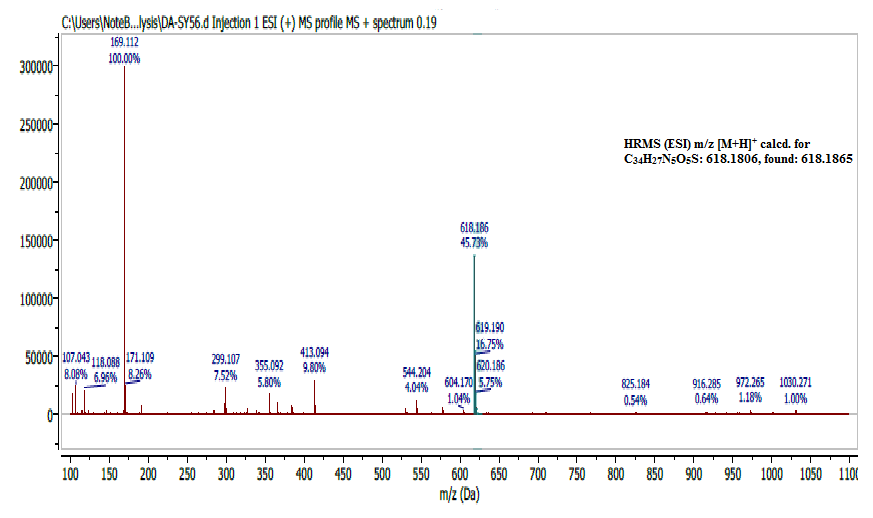
**

**Fig.S42. HRMS (ESI) spectrum of compound** **7p.**

**
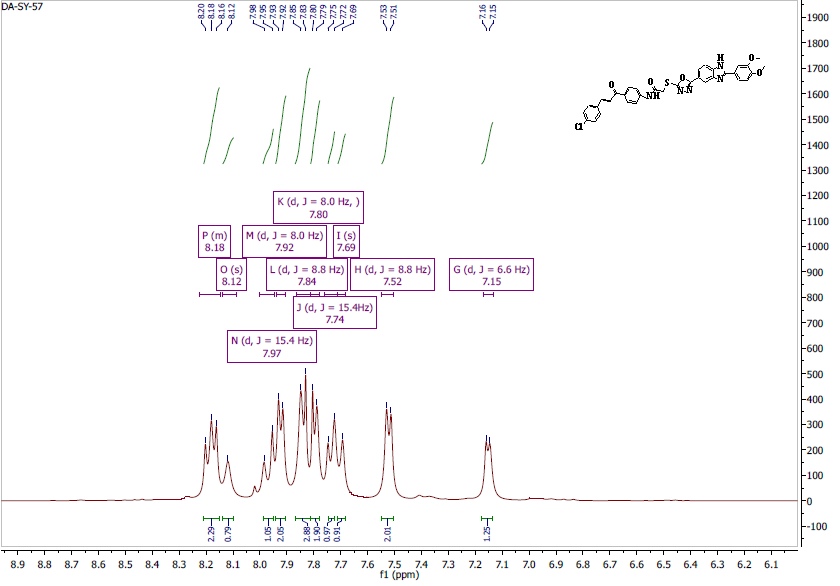
**

**
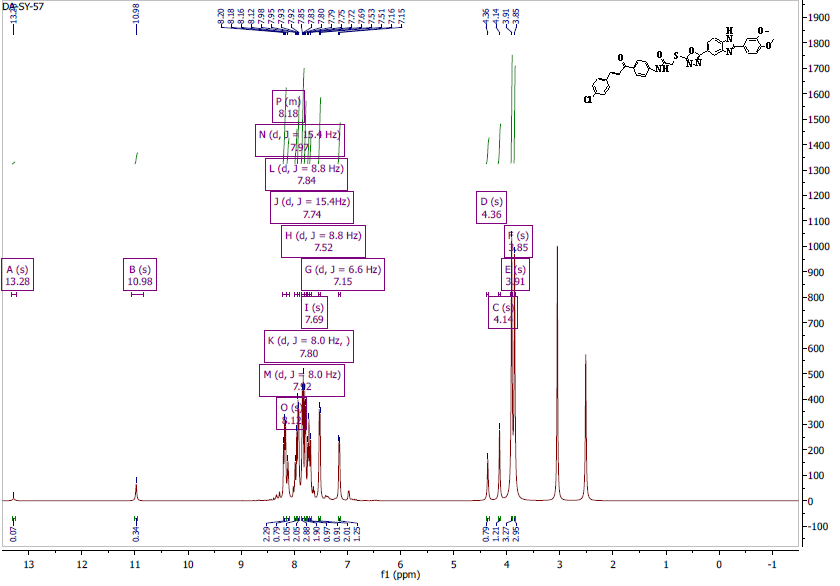
**

**Fig.S43. ^1^H NMR of compound 7q (400 MHz, DMSO-*d_6_*)**

**
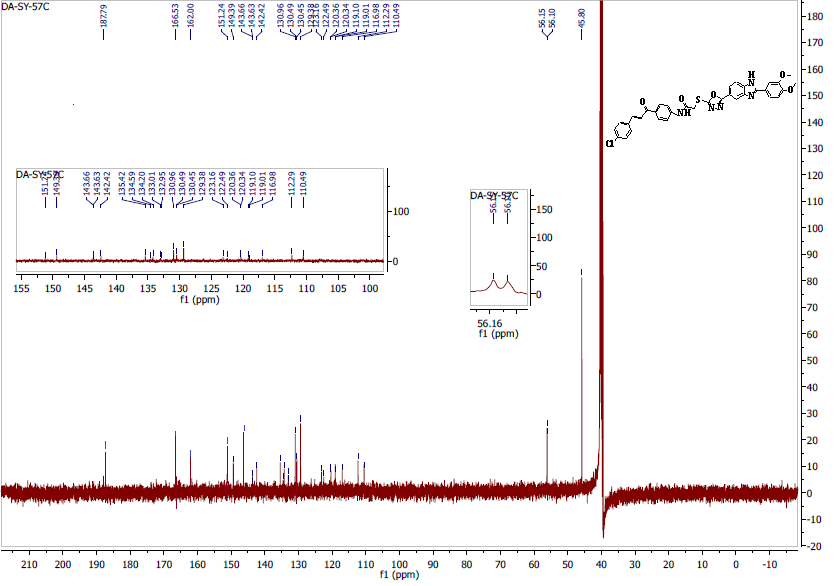
**

**Fig.S44. ^13^C NMR of compound 7q (100 MHz, DMSO-*d_6_*)**

**
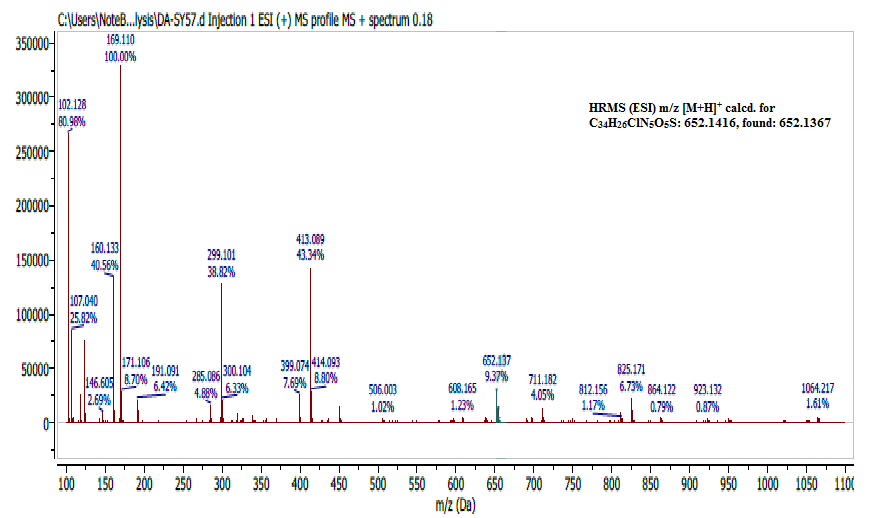
**

**Fig.S 45.** **HRMS (ESI) spectrum of compound** **7q**

**
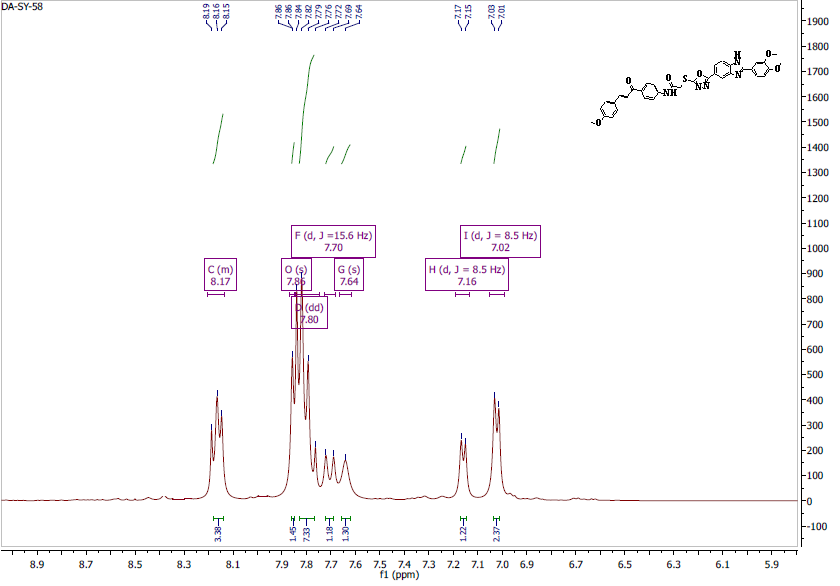
**

**
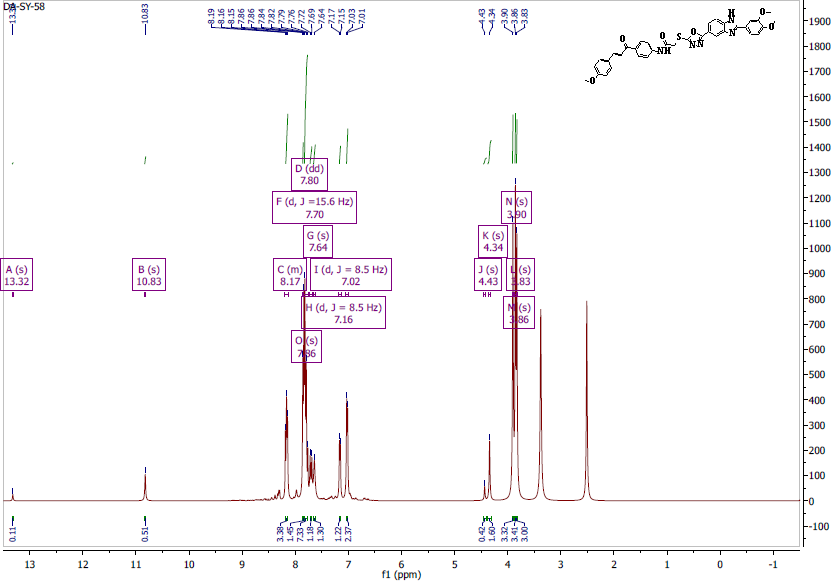
**

**Fig.S46. ^1^H NMR of compound 7r (500 MHz, DMSO-*d_6_*)**

**
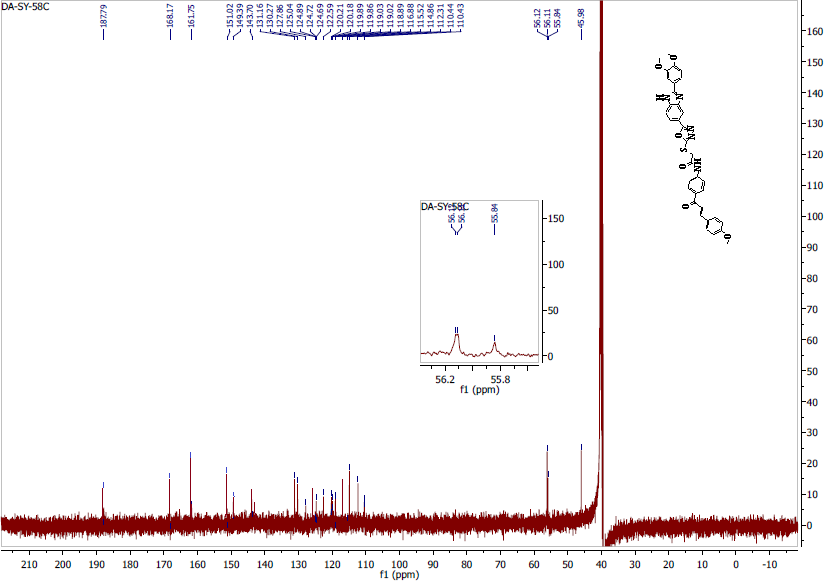
**

**Fig.S47 ^13^C NMR of compound 7r (125 MHz, DMSO-*d_6_*)**

**
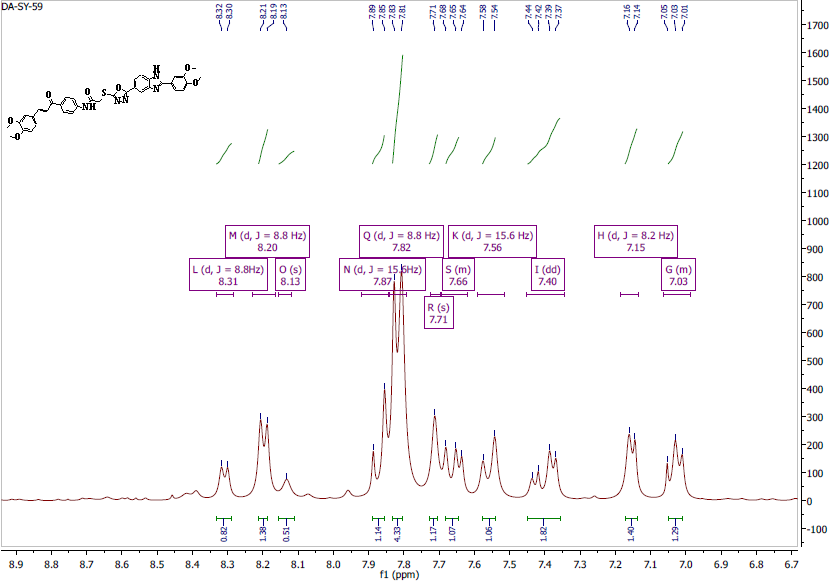
**

**
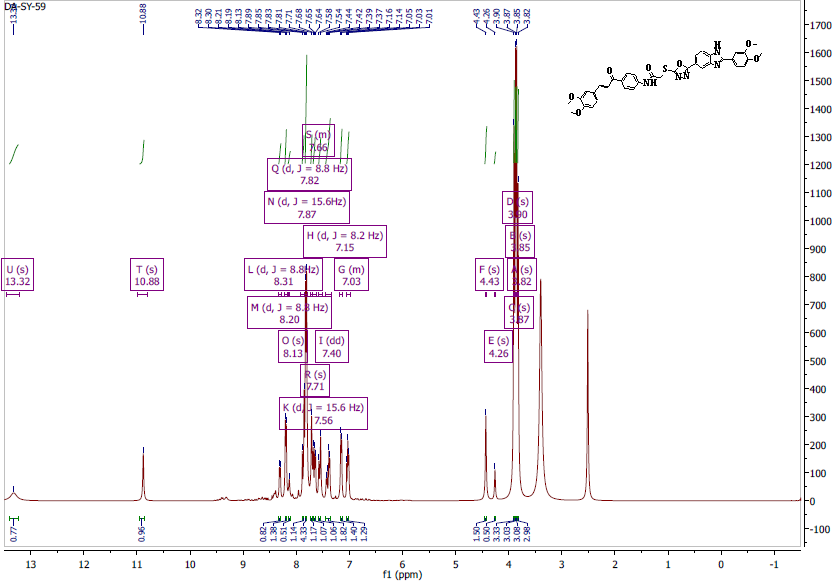
**

**Fig.S48. ^1^H NMR of compound 7s (500 MHz, DMSO-*d_6_*).**

^
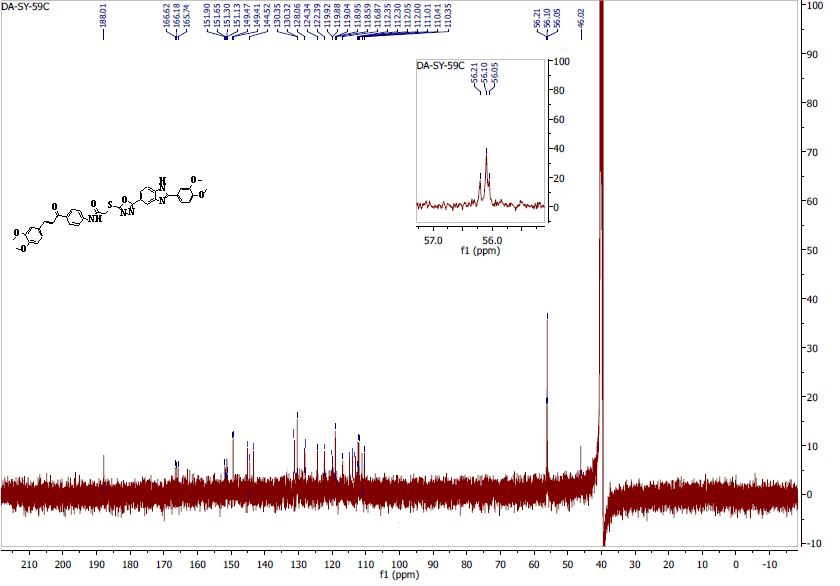
^

**Fig.S49. ^13^C NMR of compound 7s (125 MHz, DMSO-*d_6_*)**

**
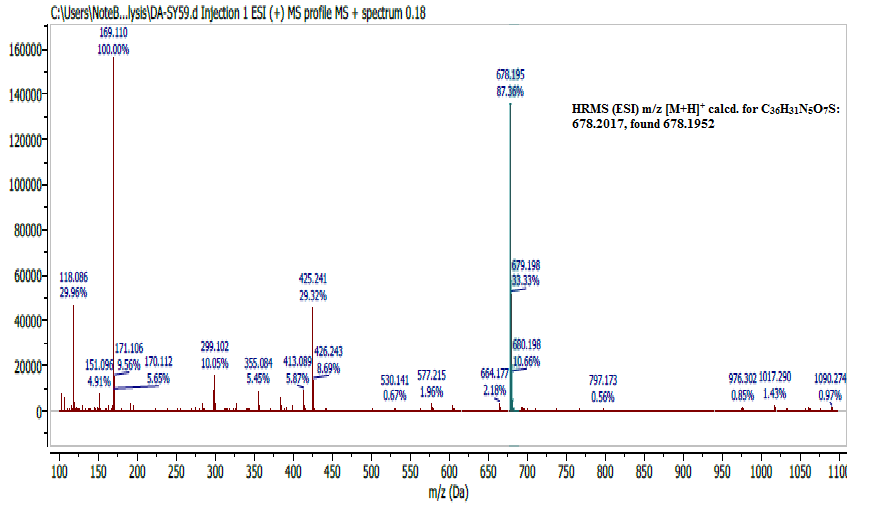
**

**Fig.S 50:** **HRMS (ESI) spectrum of compound** **7s.**

**
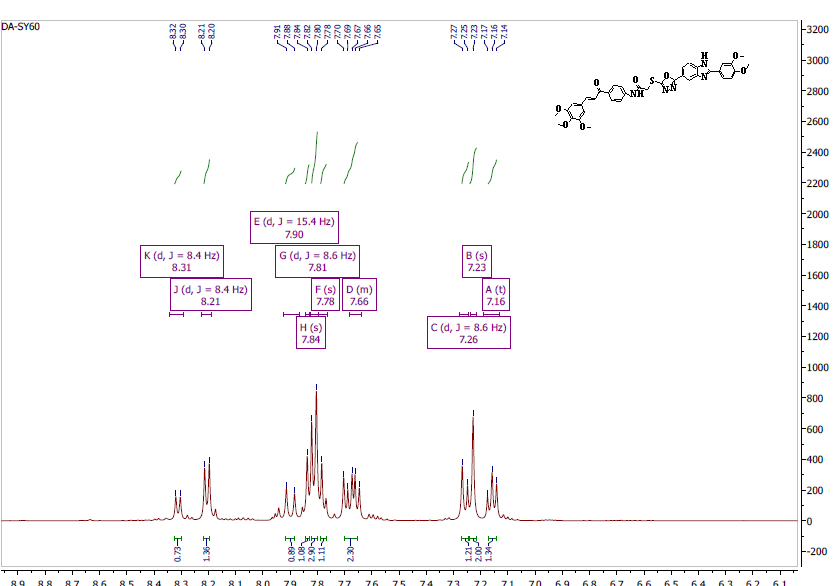
**

**
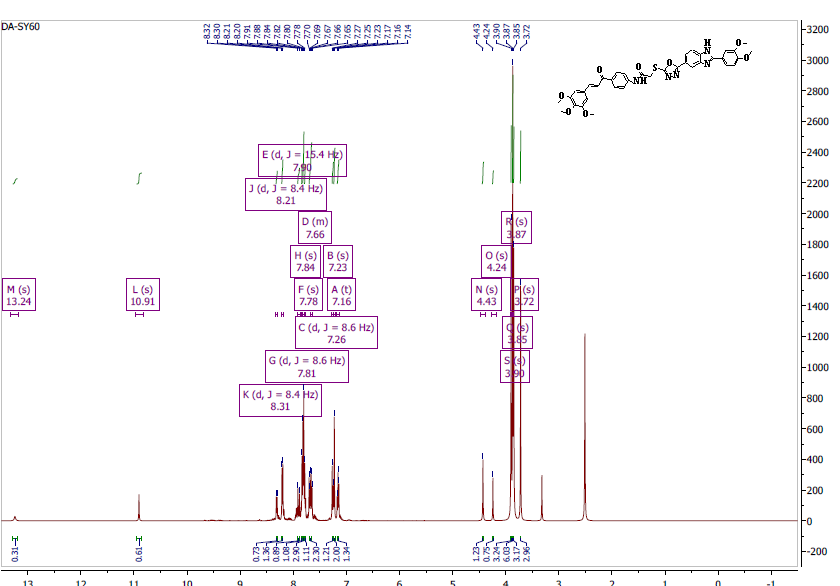
**

**Fig.S51. ^1^H NMR of compound 7t (500 MHz, DMSO-*d_6_*)**

**
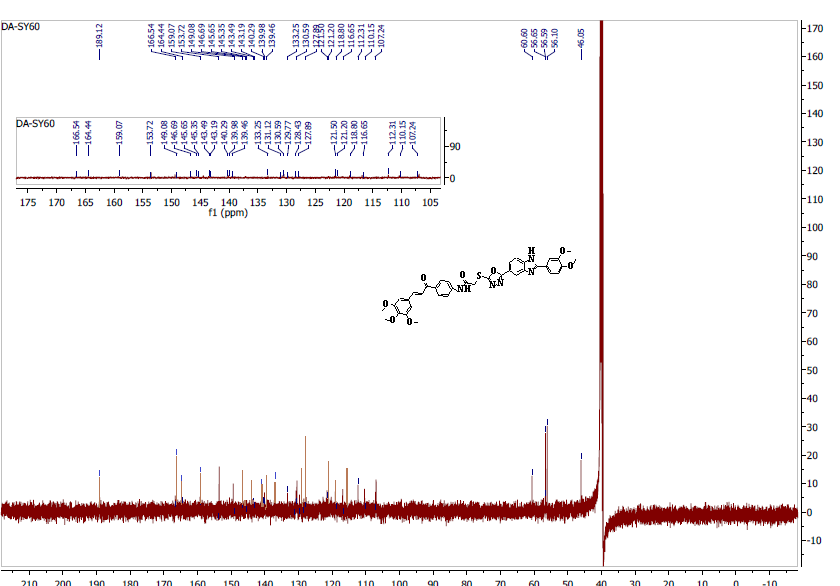
**

**Fig.S52. ^13^C NMR of compound 7t (125 MHz, DMSO-*d_6_*)**

**
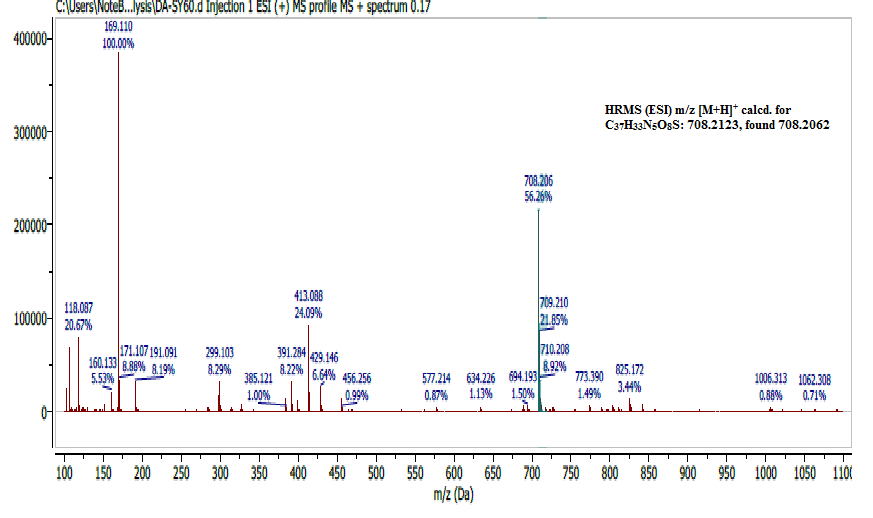
**

**Fig.S 53:** **HRMS (ESI) spectrum of compound** **7t.**

**
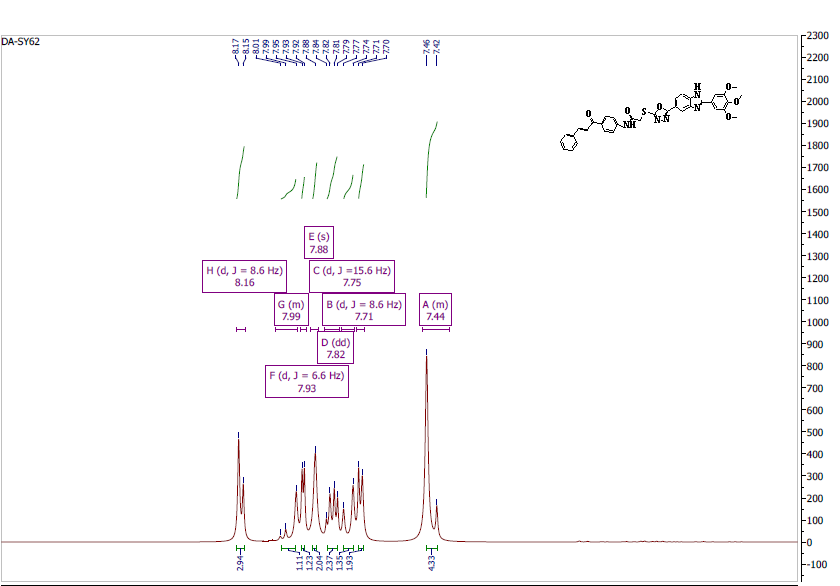
**

**
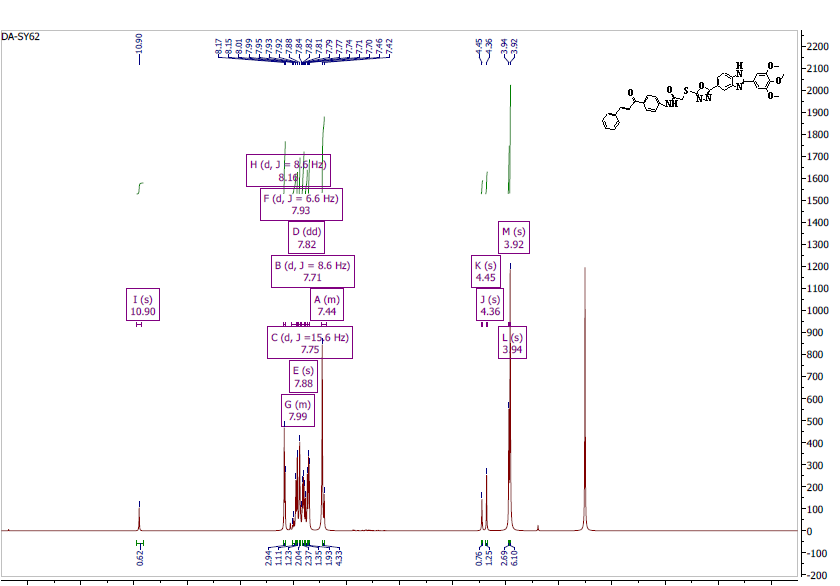
**

**Fig.S54. ^1^H NMR of compound 7u (500 MHz, DMSO-*d_6_*)**

**
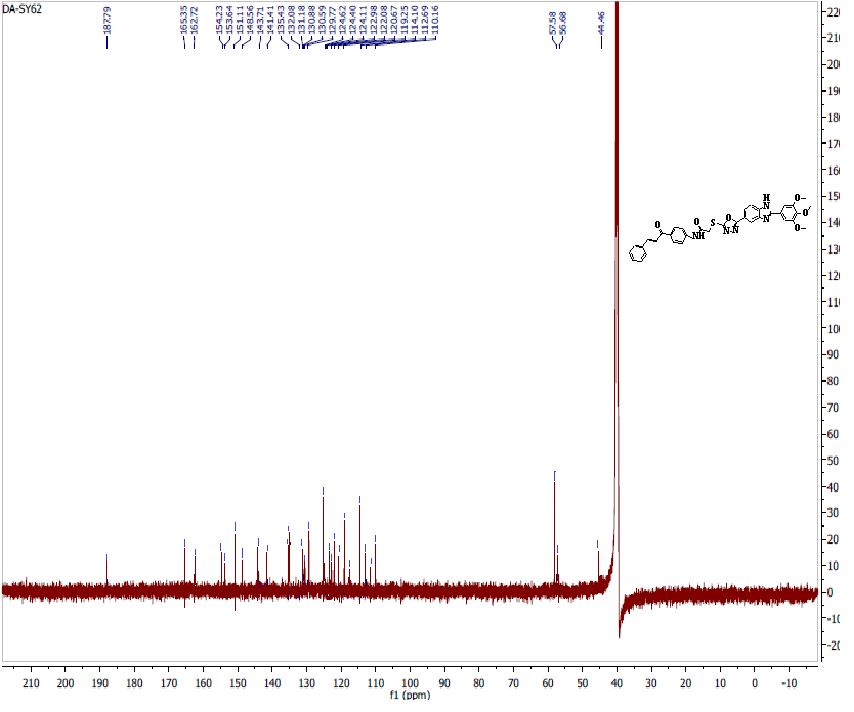
**

**Fig.S55. ^13^C NMR of compound 7u (125 MHz, DMSO-*d_6_*)**

**
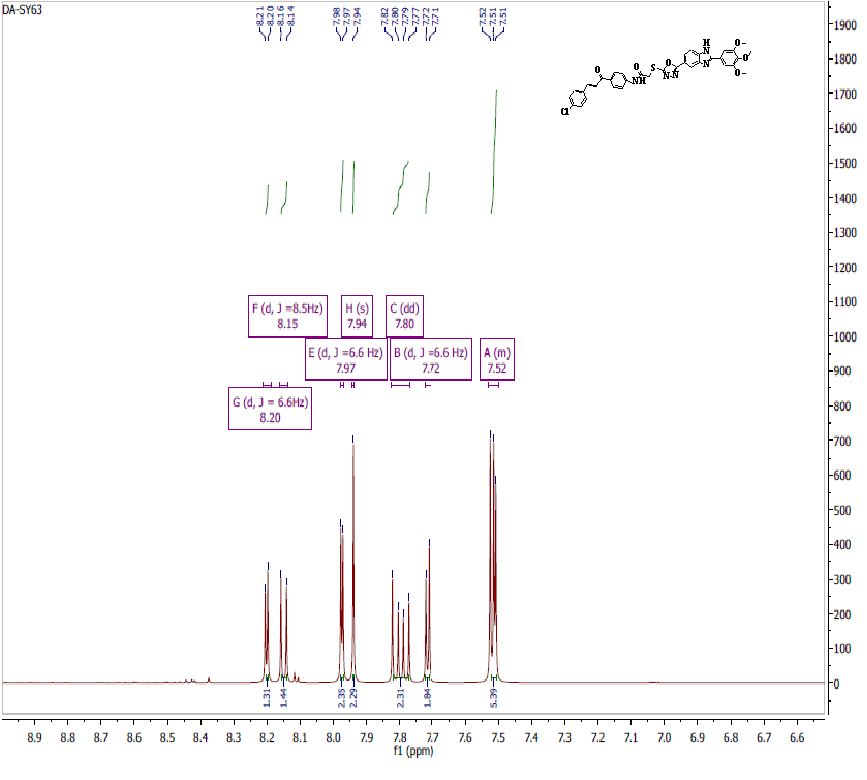
**

**
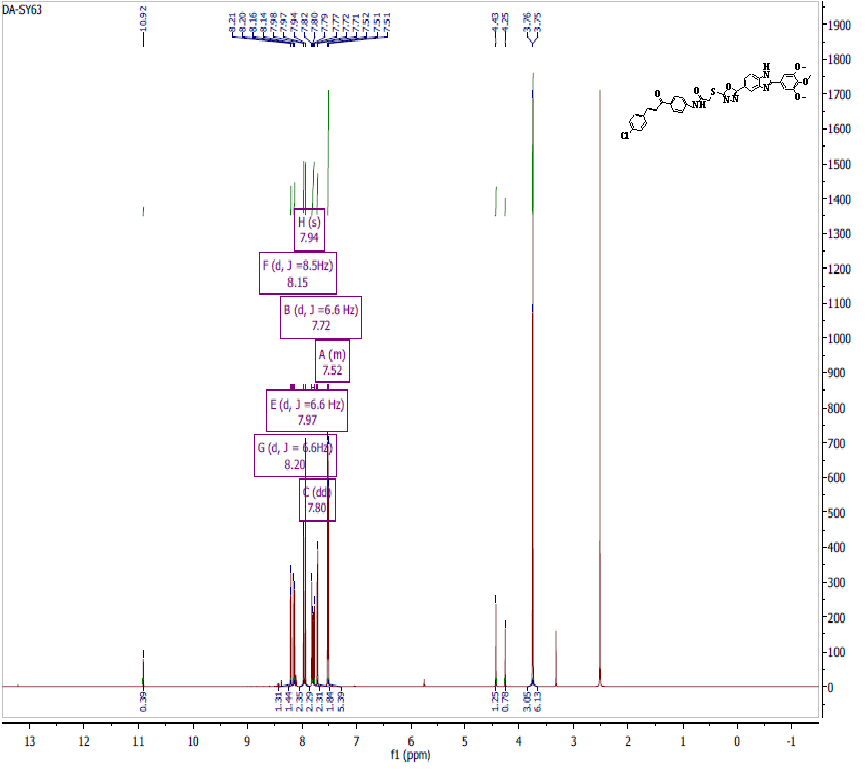
**

**Fig.S56. ^1^H NMR of compound 7v (500 MHz, DMSO-*d_6_*).**

**
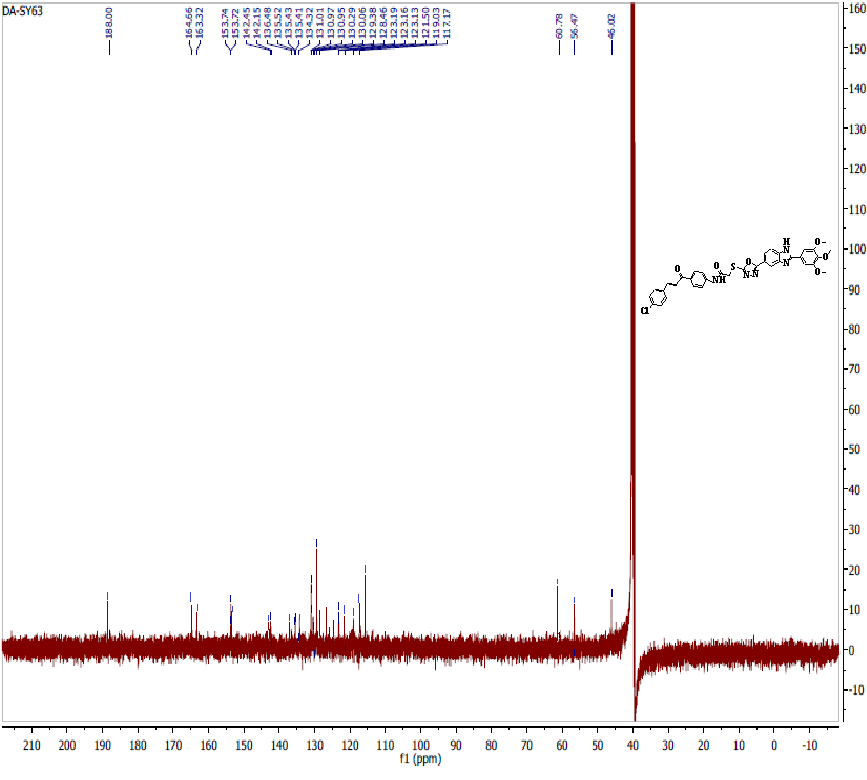
**

**Fig.S57. ^13^C NMR of compound 7v (125 MHz, DMSO-*d_6_*)**

**
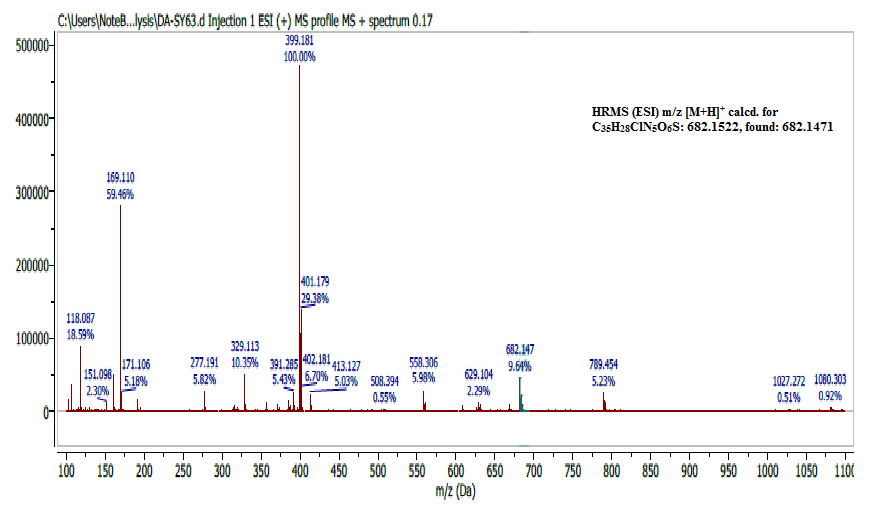
**

**Fig.S58. HRMS (ESI) spectrum of compound** **7v.**

**
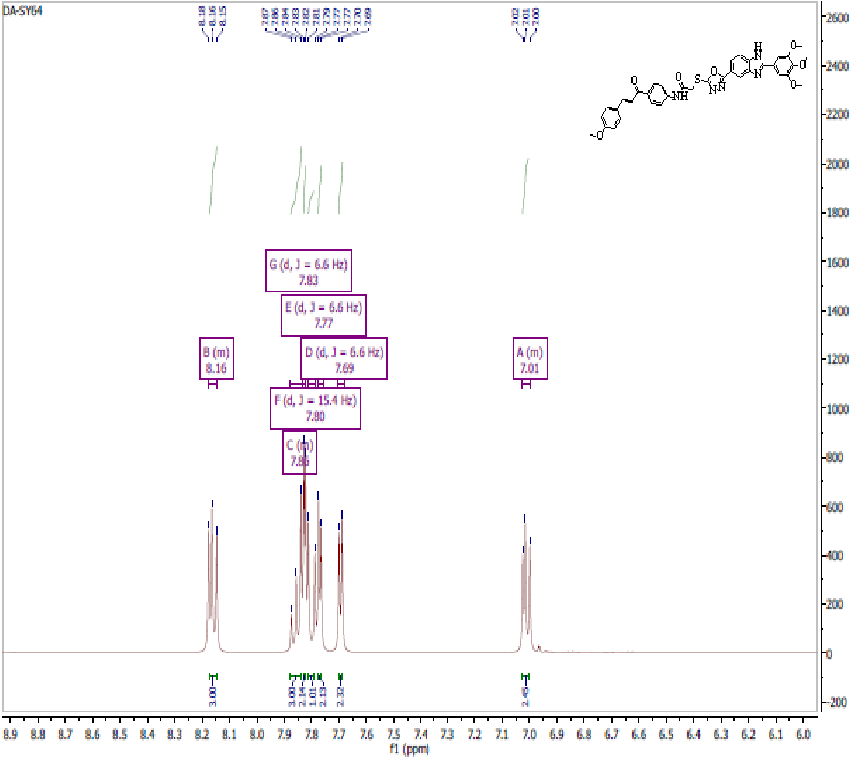
**

**
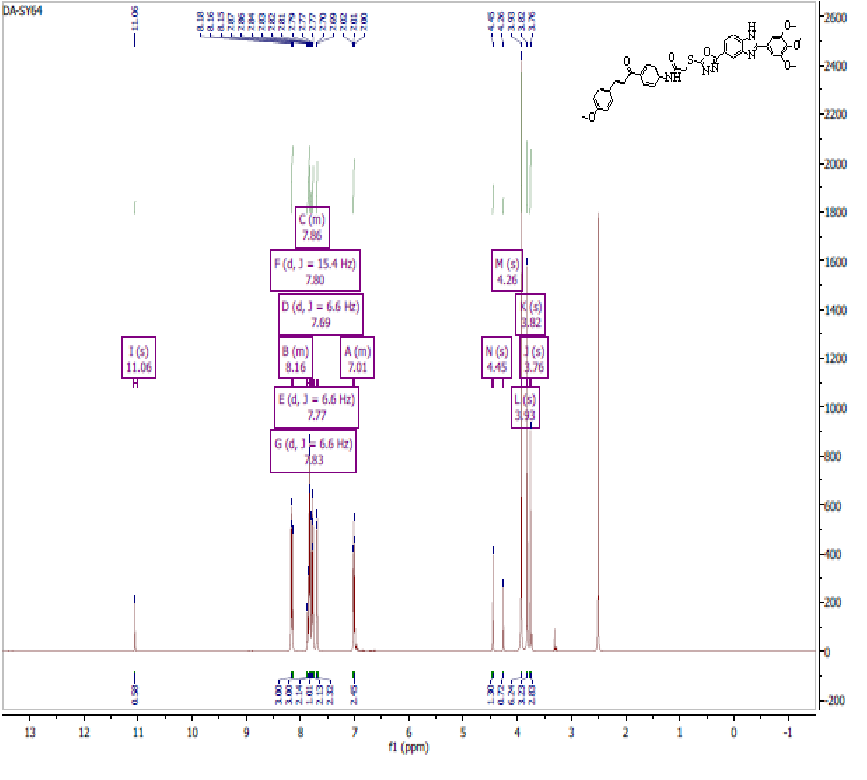
**

**Fig.S59. ^1^H NMR of compound 7w (500 MHz, DMSO-*d_6_*)**

**
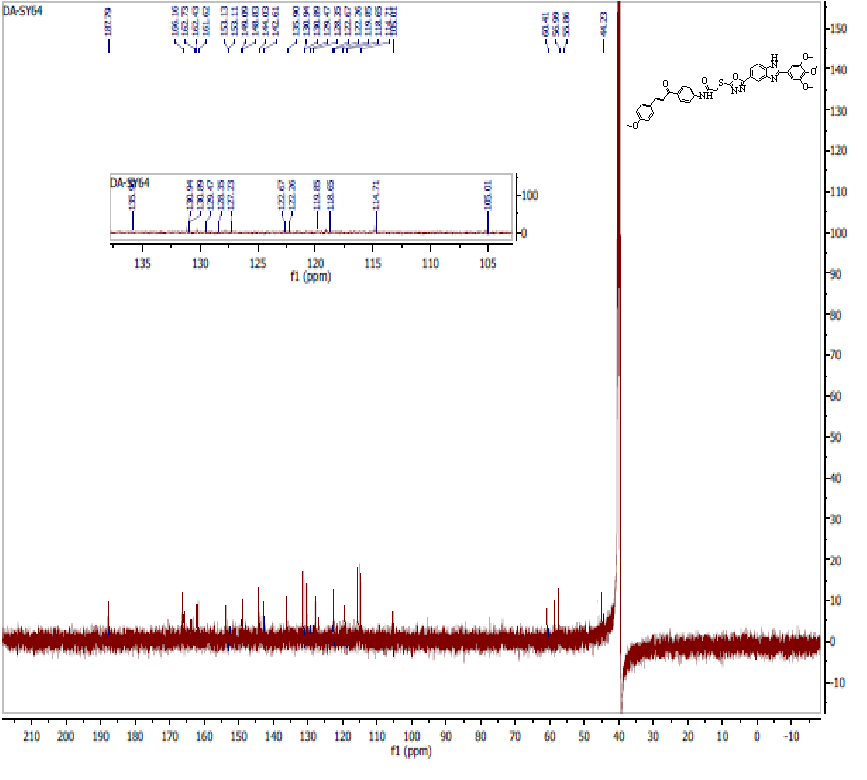
**

**Fig.S60. ^13^C NMR of compound 7w (125 MHz, DMSO-*d_6_*)**

**
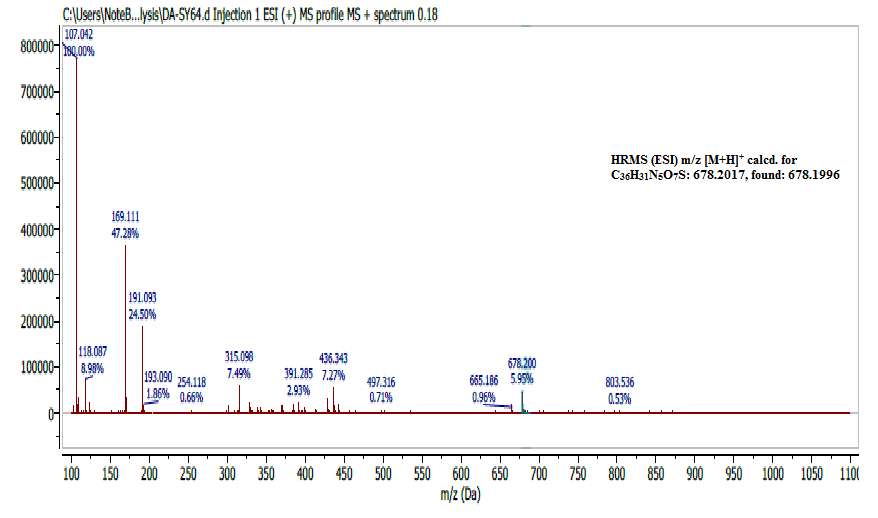
**

**Fig.S61. HRMS (ESI) spectrum of compound** **7w.**

**
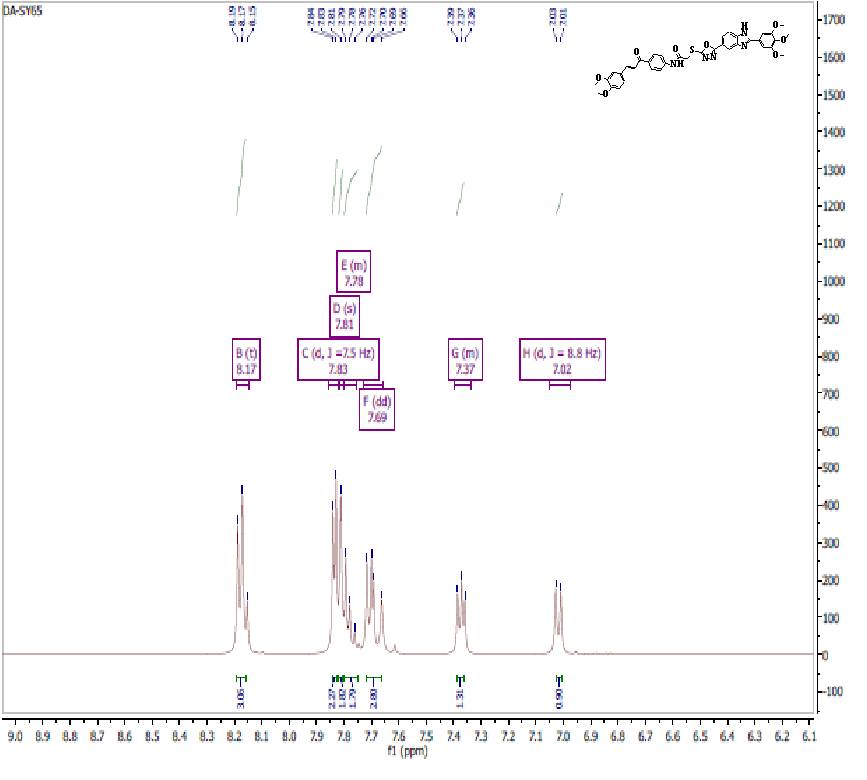
**

**
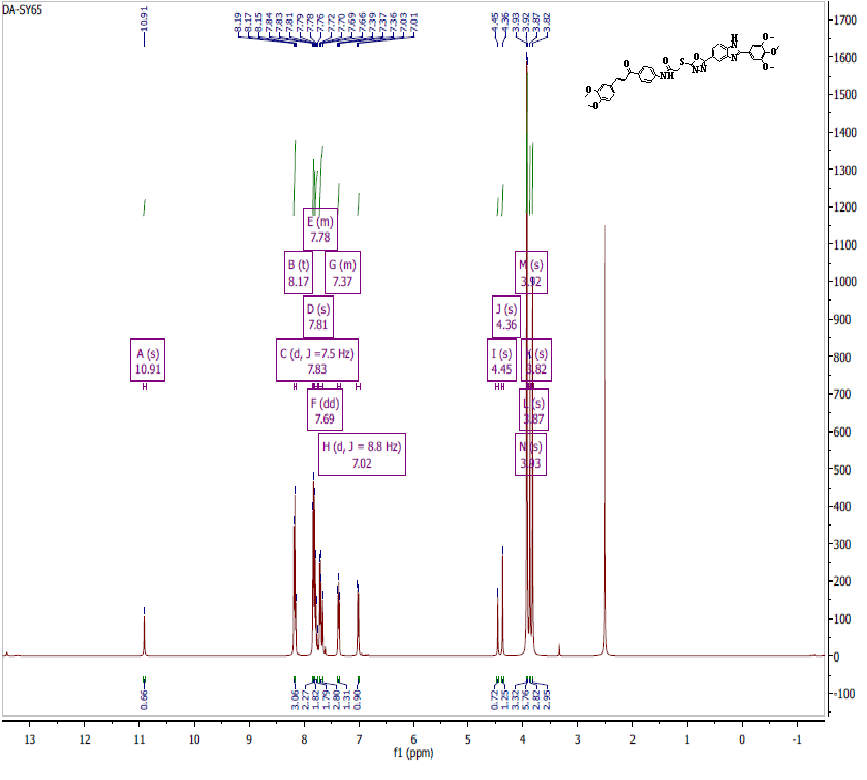
**

**Fig.S62. ^1^H NMR of compound 7x (500 MHz, DMSO-*d_6_*)**

**
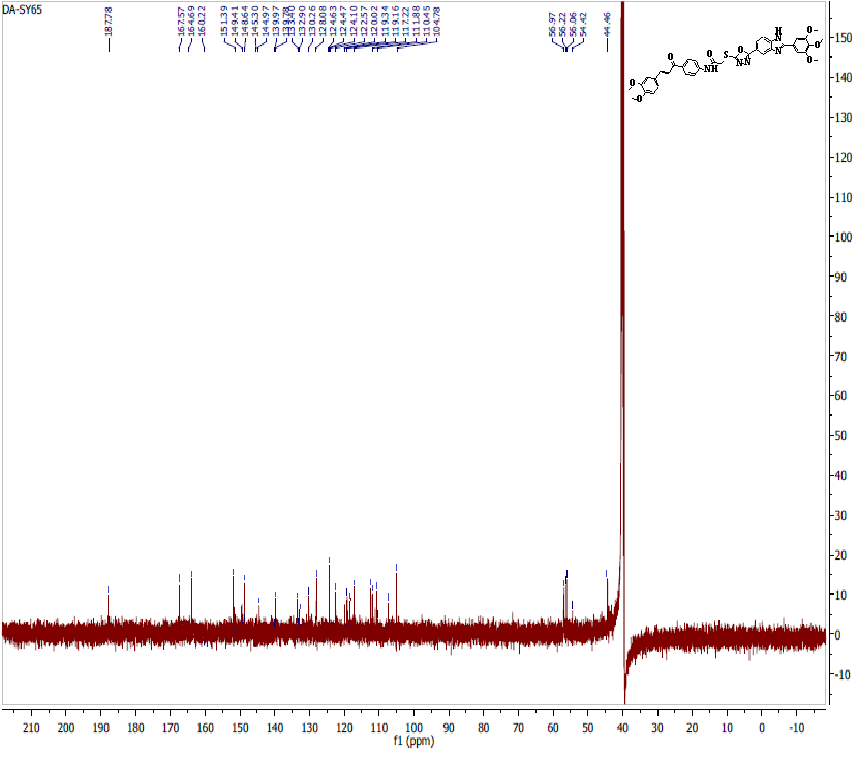
**

**Fig.S63. ^13^C NMR of compound 7x (125 MHz, DMSO-*d_6_*)**

**
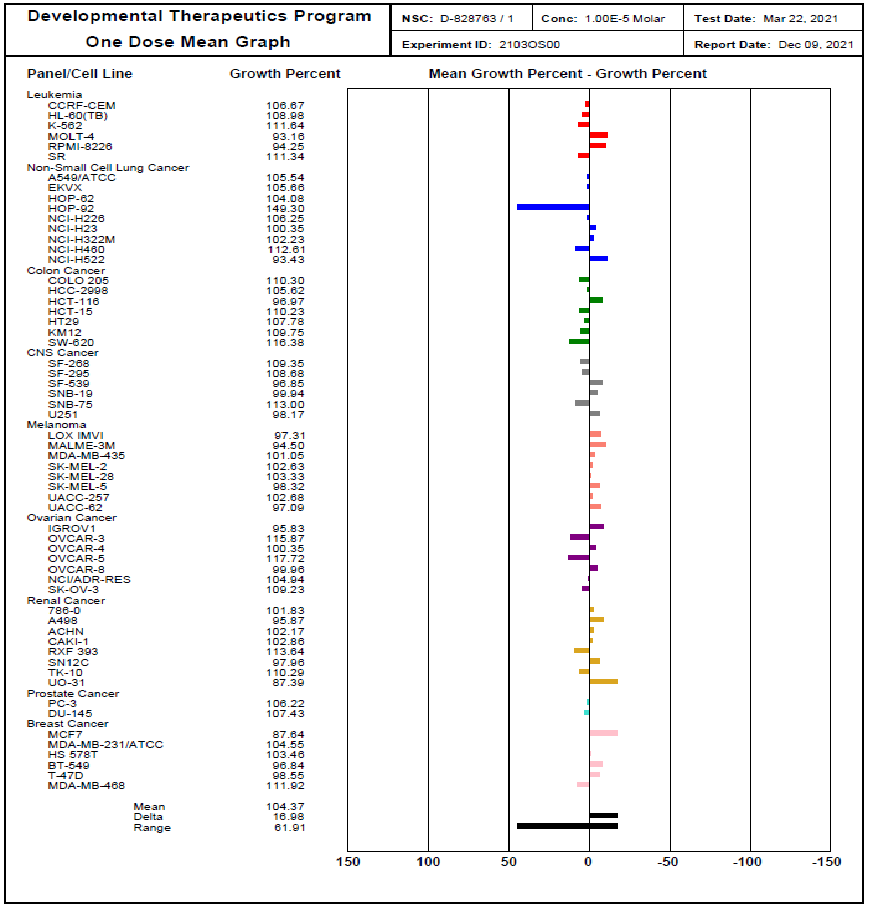
**

**Fig.S64.** one dose mean graph of nine different cancer cell line panels for compound **7a**

**Fig.S65.**One dose mean graph of nine different cancer cell line panels for compound **7b**

**Fig.S66.** one dose mean graph of nine different cancer cell line panels for compound **7c**

**
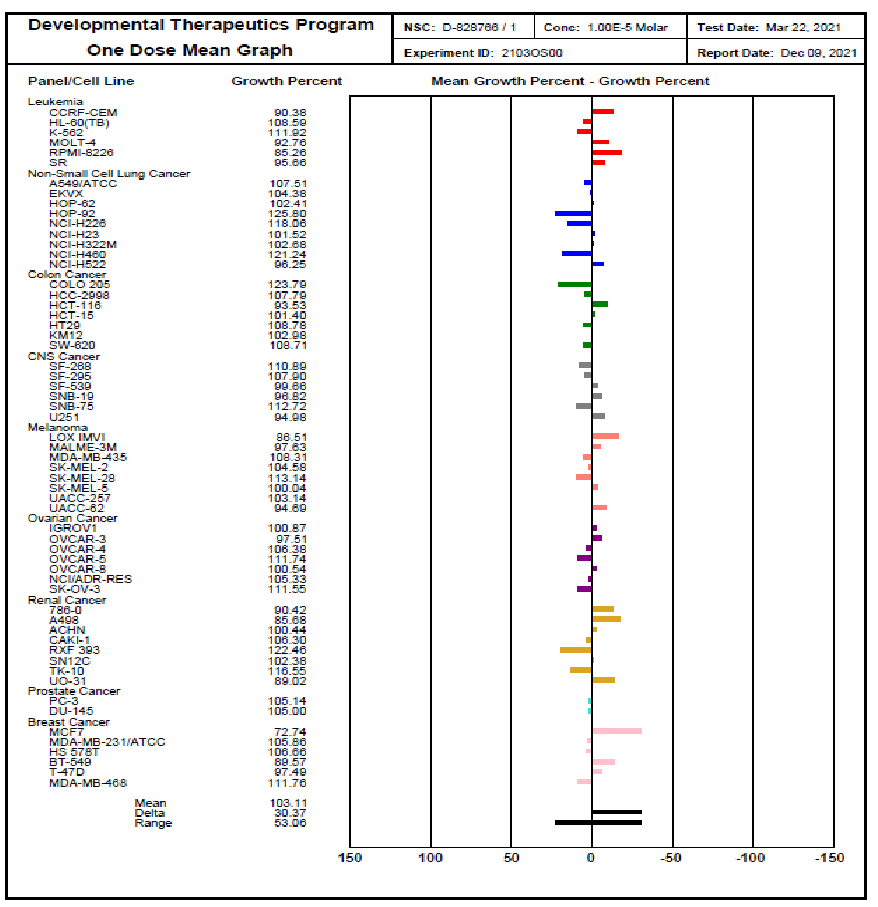
**

**Fig.S67.** one dose mean graph of nine different cancer cell line panels for compound **7d**

**
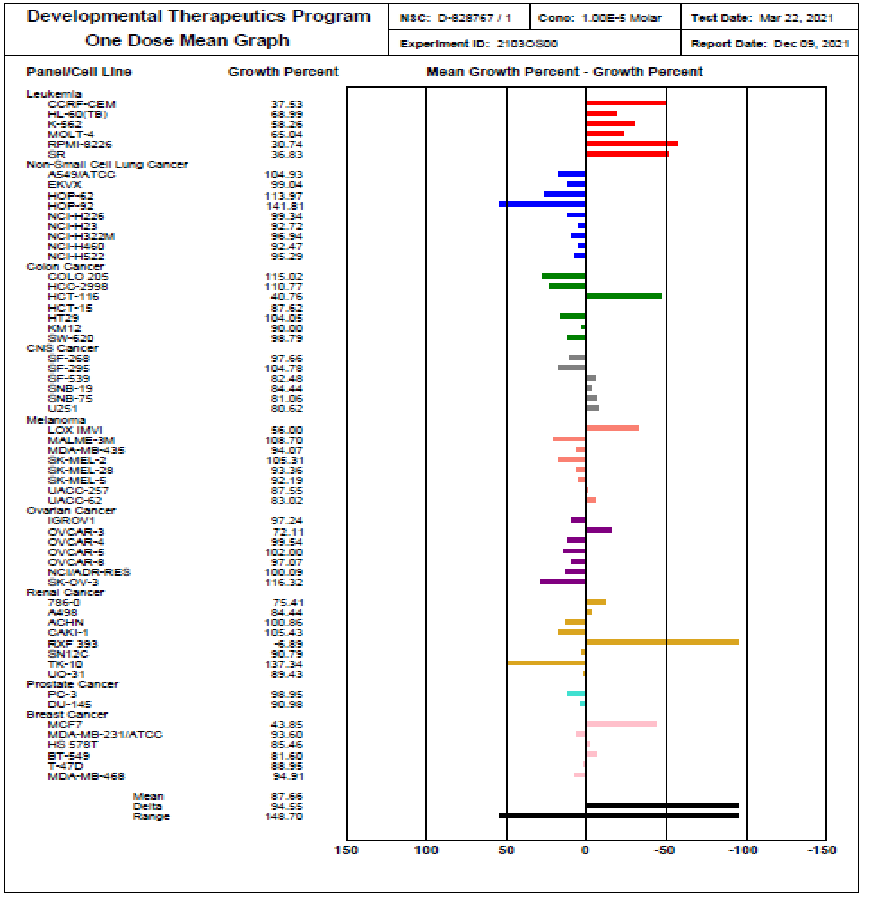
**

**Fig.S68.**One dose mean graph of nine different cancer cell line panels for compound **7e**

**
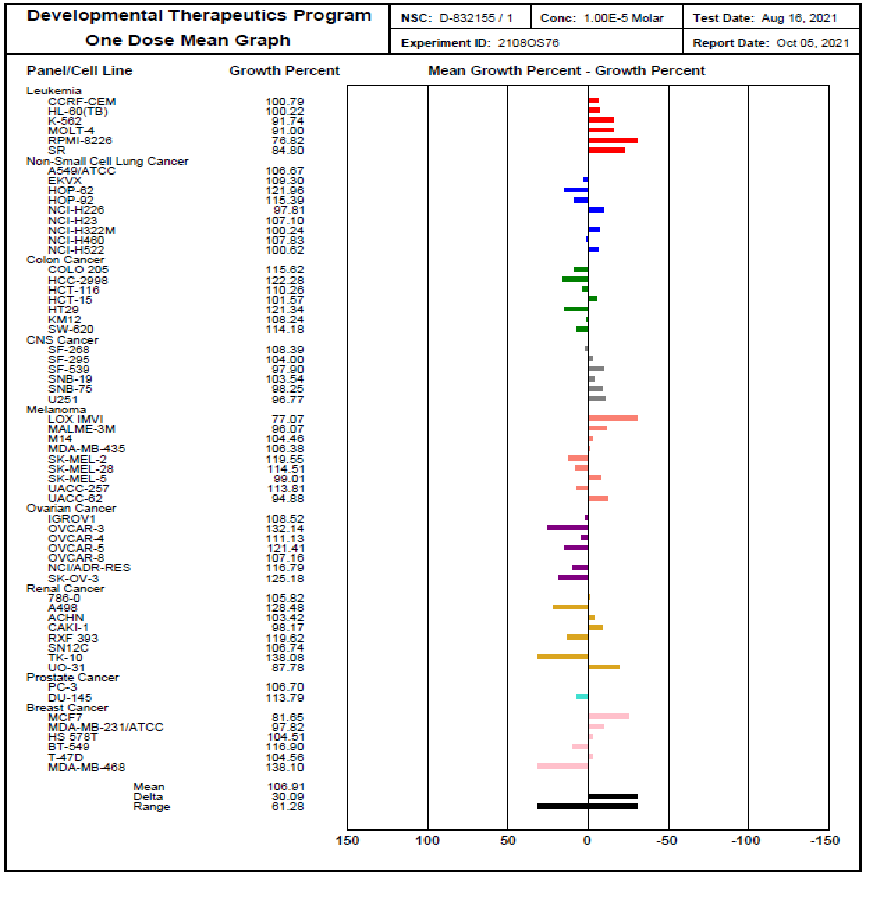
**

**Fig.S69.**One dose mean graph of nine different cancer cell line panels for compound **7f**

**Fig.S70.**One dose mean graph of nine different cancer cell line panels for compound **7g**

**
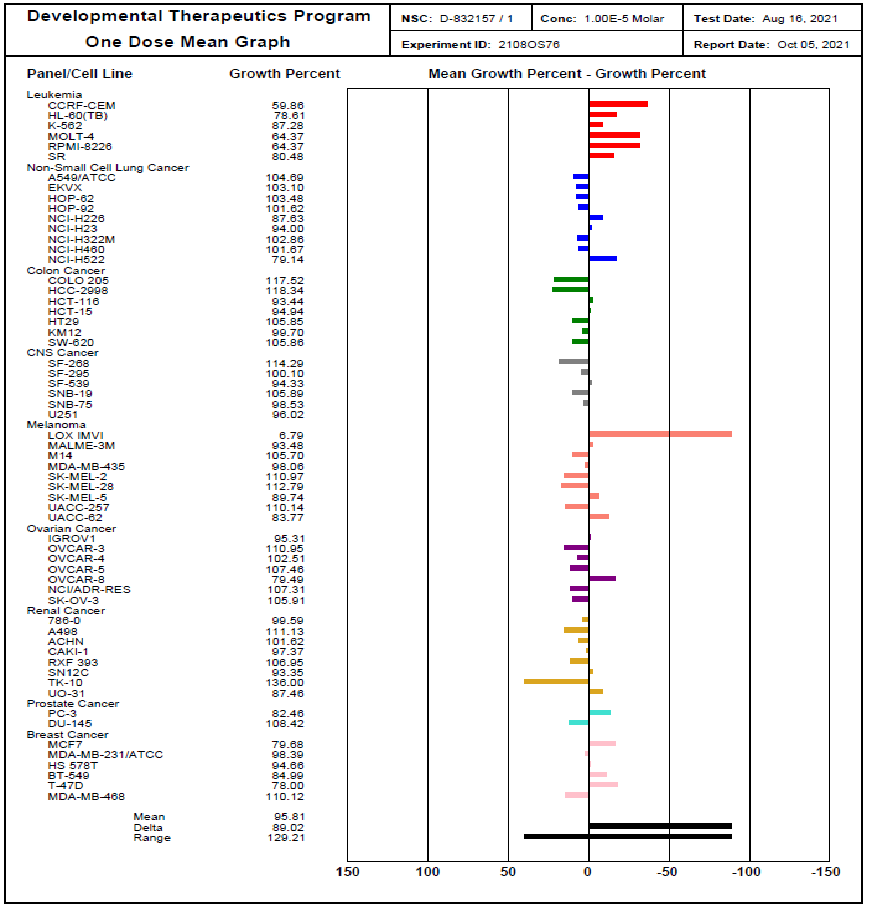
**

**Fig.S71.**One dose mean graph of nine different cancer cell line panels for compound **7h**

**
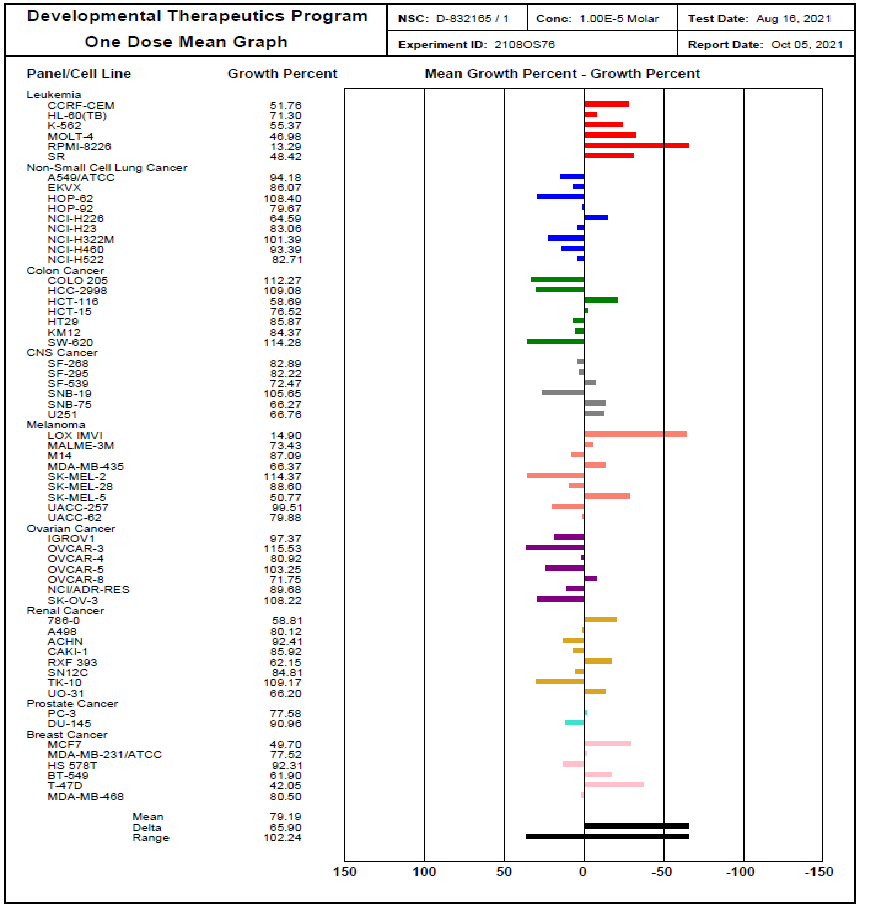
**

**Fig.S72.**One dose mean graph of nine different cancer cell line panels for compound **7k**

**
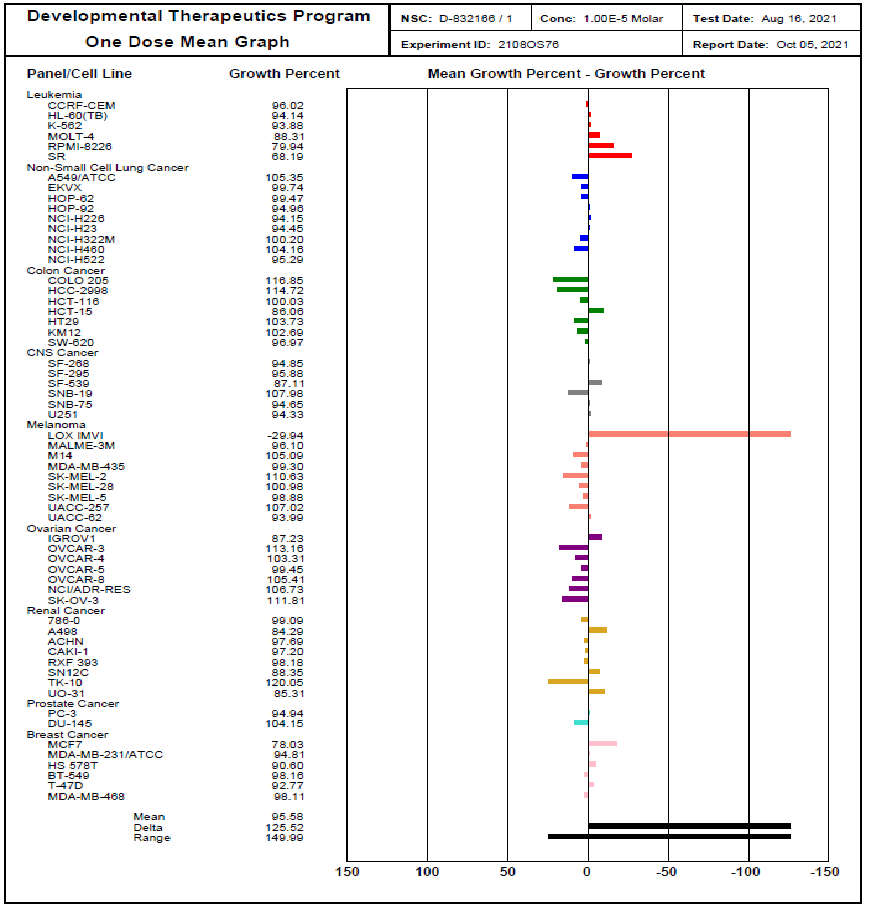
**

**Fig.S73.**One dose mean graph of nine different cancer cell line panels for compound **7l**

**
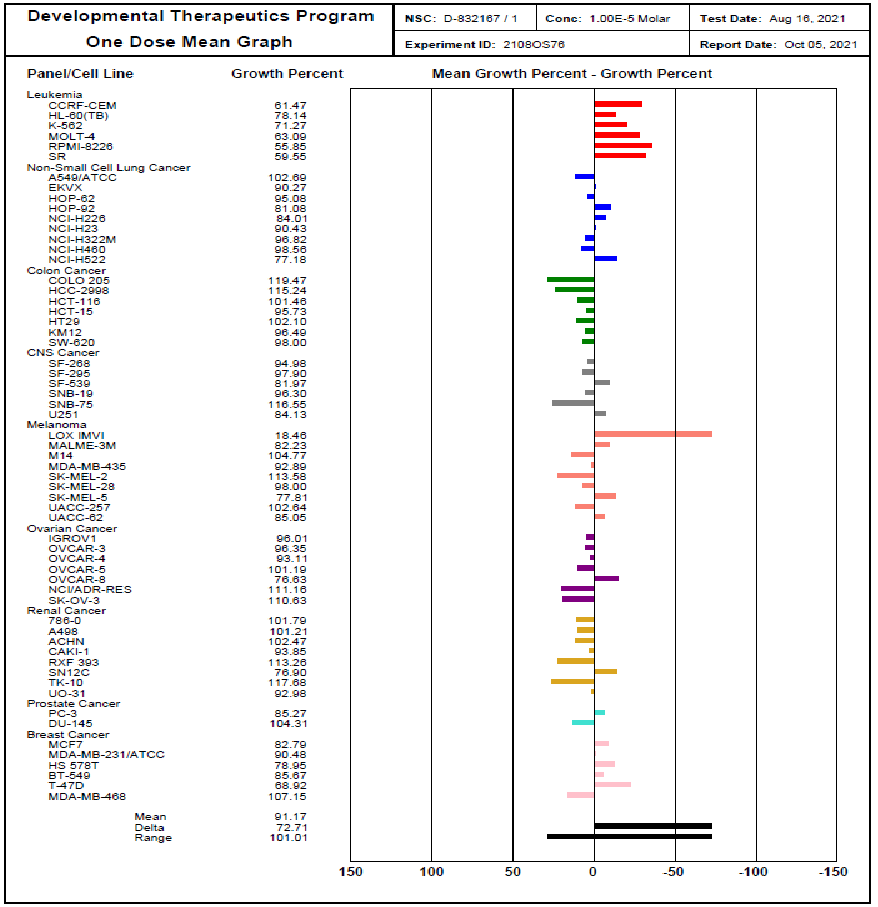
**

**Fig.S74.**One dose mean graph of nine different cancer cell line panels for compound **7m**

**
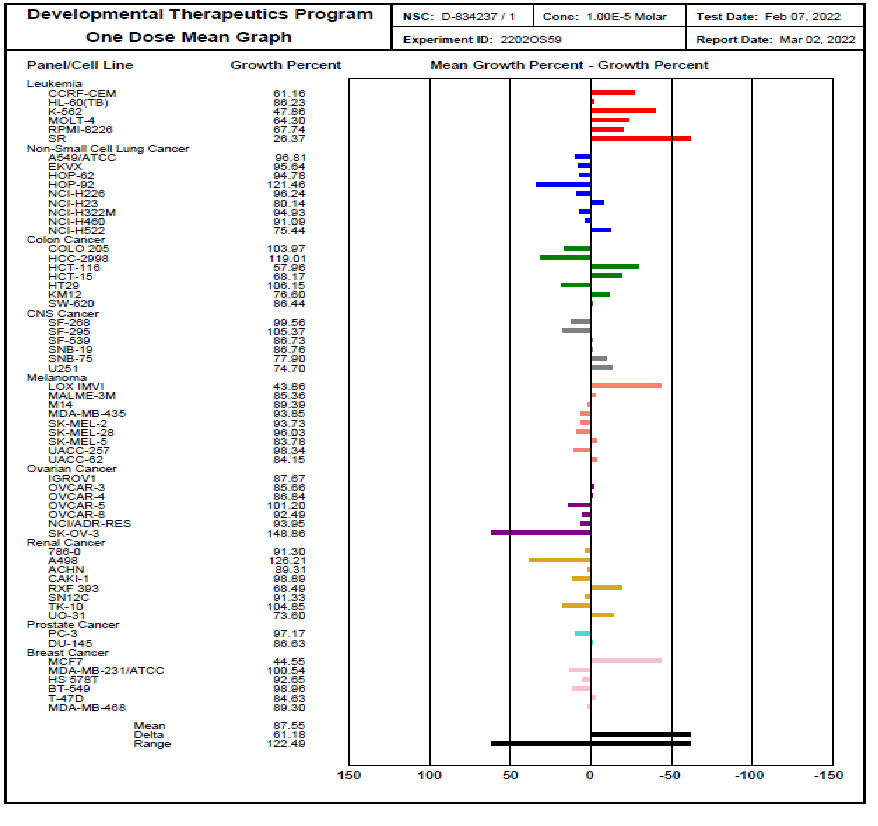
**

**Fig75.**One dose mean graph of nine different cancer cell line panels for compound **7n**

**Fig.S76.**One dose mean graph of nine different cancer cell line panels for compound **7o**

**Fig.S77.**One dose mean graph of nine different cancer cell line panels for compound **7p**

**Fig.S78.**One dose mean graph of nine different cancer cell line panels for compound **7q.**

**Fig.S79.**One dose mean graph of nine different cancer cell line panels for compound **7r.**

**
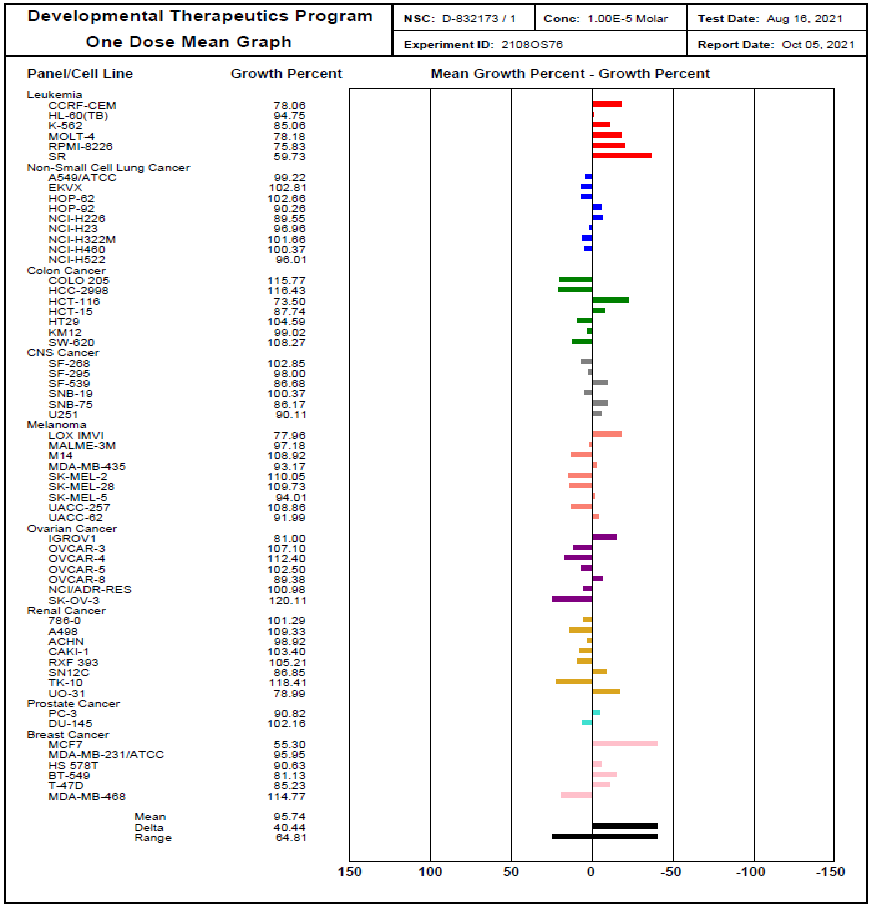
**

**Fig.S80.**One dose mean graph of nine different cancer cell line panels for compound **7u.**

**
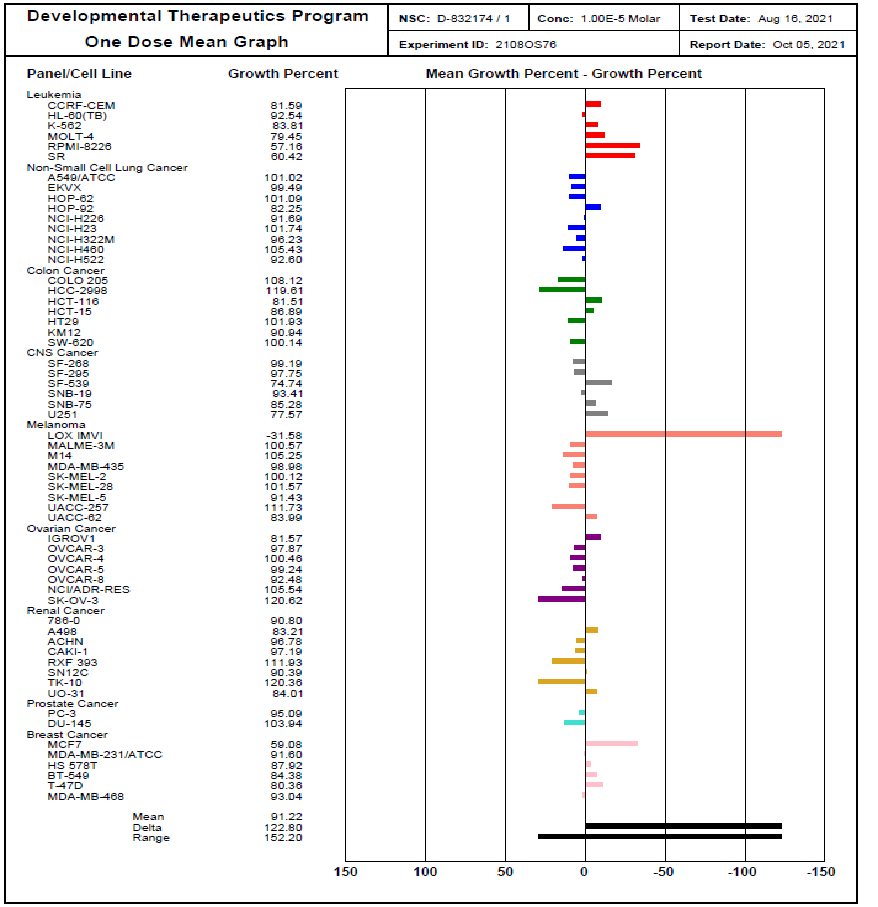
**

**Fig.S81.**One dose mean graph of nine different cancer cell line panels for compound **7v.**

|  | | | | | | | | | | | | | | | |
| --- | --- | --- | --- | --- | --- | --- | --- | --- | --- | --- | --- | --- | --- | --- | --- |
| **Table S1.** Cell growth inhibition % from the NCI's *in vitro* human tumor cell screen for compounds **7a:7e and 7k:7o.** | | | | | | | | | | | | | | | |
| **Leukemia** | | | | | | | | | | | | | | | |
| **Panel/ Cell lines** | **Compounds** | | | | | | | | | | | | | | |
|  | **7a** | | **7b** | | **7c** | | **7d** | | **7e** | **7k** | **7l** | **7m** | | **7n** | **7o** |
| **CCRF-CEM** | 0 | | 0 | | 34.46 | | 9.62 | | 62.47 | 48.24 | 3.98 | | 38.53 | 38.84 | 0 |
| **HL-60(TB)** | 0 | | 0 | | 5.24 | | 0 | | 31.01 | 28.70 | 5.86 | | 21.86 | 13.77 | 0 |
| **K-562** | 0 | | 0 | | 3.83 | | 0 | | 41.74 | 44.63 | 6.12 | | 28.73 | 52.14 | 8.26 |
| **MOLT-4** | 6.84 | | 6.55 | | 23.88 | | 7.24 | | 34.98 | 53.02 | 11.69 | | 36.91 | 35.70 | 9.00 |
| **RPMI-8226** | 5.75 | | 12.23 | | 50.87 | | 14.74 | | 69.26 | 86.71 | 20.06 | | 44.15 | 32.26 | 23.18 |
| **SR** | 0 | | 3.59 | | 12.71 | | 4.34 | | 63.17 | 51.58 | 31.81 | | 40.45 | 73.63 | 15.20 |
| **Non-Small Cell Lung Cancer** | | | | | | | | | | | | | | | |
| **A549/ATCC** | 0 | 0 | | 0 | | 0 | | | 0 | 5.82 | 0 | | 0 | 3.19 | 0 |
| **EKVX** | 0 | 0 | | 0 | | 0 | | | 0.96 | 13.93 | 0.26 | | 9.73 | 4.36 | 0 |
| **HOP-62** | 0 | 0 | | 0 | | 0 | | | 0 | 0 | 0.53 | | 4.92 | 5.22 | 0 |
| **HOP-92** | 0 | 0 | | 0 | | 0 | | | 0 | 20.33 | 5.04 | | 18.92 | 0 | 0 |
| **NCI-H226** | 0 | 3.63 | | 2.2 | | 0 | | | 0.66 | 35.41 | 5.85 | | 15.99 | 3.76 | 2.19 |
| **NCI-H23** | 0 | 0 | | 7.79 | | 0 | | | 7.28 | 16.94 | 5.55 | | 9.57 | 19.86 | 0 |
| **NCI-H322M** | 0 | 0 | | 0 | | 0 | | | 3.06 | 0 | 0 | | 3.18 | 5.07 | 0 |
| **NCI-H460** | 0 | 0 | | 0 | | 0 | | | 7.53 | 6.61 | 0 | | 1.44 | 8.91 | 0 |
| **NCI-H522**  **Table 1.** Cell growth inhibition % from the NCI's *in vitro* human tumor cell screen for compounds **9a-o** | 6.57 | 11.64 | | 8.81 | | 3.75 | | | 4.71 | 17.29 | 4.71 | | 22.82 | 24.56 | 0 |
|  | | | | | | | | | | | | | | | |
| **Panel/ cell line** | **Compounds**  **F2**  **F3**  **F4**  **F19**  **F20**  **F21**  **F22**  **F23**  **F24** | | | | | | | | | | | | | | |
|  | **7a** | **7b** | | **7c** | | **7d** | | | **7e** | **7k** | **7l** | | **7m** | **7n** | **7o** |
| **Colon cancer** | | | | | | | | | | | | | | | |
| COLO 205 | 0 | 0 | | 0 | | 0 | | | 0 | 0 | 0 | | 0 | 0 | 0 |
| HCC-2998 | 0 | 0 | | 0 | | 0 | | | 0 | 0 | 0 | | 0 | 0 | 0 |
| HCT-116 | 3.03 | 8.66 | | 15.08 | | 6.47 | | | 59.24 | 41.31 | 0 | | 0 | 42.04 | 0 |
| HCT-15 | 0 | 0 | | 3.81 | | 0 | | | 12.38 | 23.48 | 13.94 | | 4.27 | 31.83 | 0 |
| HT29 | 0 | 0 | | 1.01 | | 0 | | | 0 | 14.13 | 0 | | 0 | 0 | 0 |
| KM12 | 0 | 0 | | 1.55 | | 0 | | | 10.00 | 15.63 | 0 | | 3.51 | 23.40 | 0 |
| SW-620 | 0 | 0 | | 0 | | 0 | | | 1.21 | 0 | 3.03 | | 2 | 13.56 | 0 |
| **CNS Cancer** | | | | | | | | | | | | | | | |
| SF-268 | 0 | 0 | | 0 | | 0 | | | 2.34 | 17.11 | 5.15 | | 5.02 | 0.44 | 0 |
| SF-295 | 0 | 0 | | 1.65 | | 0 | | | 0 | 17.78 | 4.12 | | 2.10 | 0 | 0 |
| SF-539 | 3.15 | 4.06 | | 8.76 | | 0.34 | | | 17.52 | 27.53 | 12.89 | | 18.03 | 13.27 | 2.10 |
| SNB-19 | 0.06 | 3.92 | | 7.28 | | 3.18 | | | 15.56 | 0 | 0 | | 3.70 | 13.24 | 0 |
| SNB-75 | 0 | 0 | | 0 | | 0 | | | 18.94 | 33.73 | 5.35 | | 0 | 22.10 | 1.75 |
| U251 | 1.83 | 7.14 | | 17.24 | | 5.02 | | | 19.38 | 33.24 | 5.67 | | 15.87 | 25.30 | 3.23 |
| **Prostate Cancer** | | | | | | | | | | | | | | | |
| PC-3 | 0 | 0.53 | | 5.12 | | 0 | | | 1.05 | 22.42 | 5.06 | | 14.73 | 2.83 | 0 |
| DU-145 | 0 | 0 | | 0 | | 0 | | | 9.02 | 9.04 | 0 | | 0 | 13.37 | 0 |
| **Table S1(continuous).** Cell growth inhibition % from the NCI's *in vitro* human tumor cell screen for compounds **7a:7e and 7k:7o.** | | | | | | | | | | | | | | | |
|  |  |  | |  | |  | | |  |  |  | |  |  |  |
| **Panel/Cell Line** | **Compounds**  **F1**  **F2**  **F3**  **F4**  **F19**  **F20**  **F21**  **F22**  **F23**  **F24** | | | | | | | | | | | | | | |
|  | **7a** | **7b** | | **7c** | | **7d** | | | **7e** | **7k** | **7l** | | **7m** | **7n** | **7o** |
| **Melanoma** | | | | | | | | | | | | | | | |
| LOX IMVI | 2.69 | 12.87 | | 6.46 | | 13.49 | | 44.00 | | 85.10 | 129.94 | | 81.54 | 56.14 | 22.93 |
| MALME-3M | 5.5 | 0 | | 8.64 | | 2.37 | | 0 | | 26.57 | 3.90 | | 17.77 | 14.64 | 3.93 |
| M14 | - | - | | - | | - | | - | | 12.91 | 0 | | 0 | 10.61 | 0 |
| MDA-MB-435 | 0 | 0 | | 0 | | 0 | | 5.93 | | 33.63 | 0.7 | | 7.11 | 6.15 | 0 |
| SK-MEL-2 | 0 | 0 | | 2.65 | | 0 | | 0 | | 0 | 0 | | 0 | 6.27 | 0 |
| SK-MEL-28 | 0 | 0 | | 0 | | 0 | | 6.64 | | 11.40 | 0 | | 2.00 | 3.97 | 0 |
| SK-MEL-5 | 1.68 | 3.35 | | 16.82 | | 0 | | 7.81 | | 49.23 | 1.12 | | 22.19 | 16.22 | 0.99 |
| UACC-257 | 0 | 0 | | 0 | | 0 | | 12.45 | | 0.49 | 0 | | 0 | 1.66 | 0 |
| UACC-62 | 2.91 | 11.64 | | 9.5 | | 5.31 | | 16.98 | | 20.12 | 6.01 | | 14.95 | 15.85 | 5.12 |
| **Ovarian Cancer** | | | | | | | | | | | | | | | |
| IGROV1 | 4.17 | 15.31 | | 0 | | 0 | | | 2.76 | 2.63 | 12.77 | | 3.99 | 12.33 | 0 |
| OVCAR-3 | 0 | 0 | | 0 | | 2.49 | | | 27.89 | 0 | 0 | | 3.65 | 14.34 | 0 |
| OVCAR-4 | 0 | 0 | | 0 | | 0 | | | 0.46 | 19.08 | 0 | | 6.89 | 13.16 | 0 |
| OVCAR-5 | 0 | 0 | | 0 | | 0 | | | 0 | 0 | 0.55 | | 0 | 0 | 0 |
| OVCAR-8 | 0.04 | 2.36 | | 9.58 | | 0 | | | 2.93 | 28.25 | 0 | | 23.37 | 7.51 | 0 |
| NCI/ADR-RES | 0 | 0 | | 0 | | 0 | | | 0 | 10.32 | 0 | | 0 | 6.05 | 0 |
| SK-OV-3 | 0 | 0 | | 0 | | 0 | | | 0 | 0 | 0 | | 0 | 0 | 0 |
| **Table S1.** Cell growth inhibition % from the NCI's *in vitro* human tumor cell screen for compounds **7a:7e and 7k:7o.** | | | | | | | | | | | | | | | |
| **Panel/Cell Line** | **Compounds** | | | | | | | | | | | | | | |
|  | **7a** | **7b** | | **7c** | | **7d** | | **7e** | | **7k** | **7l** | | **7m** | **7n** | **7o** |
| **Renal cancer** | | | | | | | | | | | | | | | |
| 786-0 | 0 | 2.96 | | 22.55 | | 9.58 | | 24.59 | | 41.19 | 0.91 | | 0 | 8.70 | 0 |
| A498 | 4.13 | 6.84 | | 22.56 | | 14.32 | | 15.56 | | 19.88 | 15.71 | | 0 | 0 | 0 |
| ACHN | 0 | 0 | | 2.58 | | 0 | | 0 | | 7.59 | 2.31 | | 0 | 10.69 | 0 |
| CAKI-1 | 0 | 1.52 | | 0 | | 0 | | 0 | | 14.08 | 2.80 | | 6.15 | 1.11 | 1.83 |
| RXF 393 | 0 | 0 | | 8.38 | | 0 | | 106.89 | | 37.85 | 1.82 | | 0 | 31.51 | 0 |
| SN 12C | 2.04 | 9.80 | | 3.32 | | 0 | | 9.21 | | 15.19 | 11.65 | | 23.10 | 8.67 | 0 |
| TK-10 | 0 | 0 | | 0 | | 0 | | 0 | | 0 | 0 | | 0 | 0 | 0 |
| UO-31 | 12.61 | 25.50 | | 8.67 | | 10.98 | | 10.57 | | 33.80 | 14.69 | | 7.02 | 26.40 | 12.22 |
| **Breast cancer** | | | | | | | | | | | | | | | |
| MCF7 | 12.36 | 16.81 | | 27.48 | | 27.26 | | 56.15 | | 50.3 | 21.97 | | 17.21 | 55.45 | 18.35 |
| MDA-MB-231/ATCC | 0 | 4.03 | | 0 | | 0 | | 6.4 | | 22.48 | 5.19 | | 9.52 | 0 | 2.18 |
| HS 578T | 0 | 0 | | 0 | | 0 | | 14.54 | | 7.69 | 9.40 | | 21.05 | 7.35 | 0 |
| BT-549 | 3.16 | 1.92 | | 18.67 | | 10.43 | | 18.40 | | 38.10 | 1.84 | | 14.33 | 1.04 | 0 |
| T-47D | 1.45 | 12.32 | | 47.65 | | 2.51 | | 11.05 | | 57.95 | 7.23 | | 31.08 | 15.37 | 0 |
| MDA-MB-468 | 0 | 0 | | 0 | | 0 | | 5.09 | | 19.50 | 1.89 | | 0 | 10.70 | 0 |

| **Table S2.** Cell growth inhibition % from the NCI's *in vitro* human tumor cell screen for compounds **7p, 7q, 7r, 7u, 7v and 7f to 7h.** | | | | | | | | |
| --- | --- | --- | --- | --- | --- | --- | --- | --- |
| **Panel /Cell Lines** | **Compounds** | | | | | | | |
|  | **7p** | **7q** | **7r** | **7u** | **7v** | **7f** | **7g** | **7h** |
| **Leukemia** | | | | | | | | |
| **CCRF-CEM** | 11.61 | 0 | 38.56 | 21.94 | 18.41 | 0 | 13.58 | 40.14 |
| **HL-60(TB)** | 0 | 11.28 | 20.87 | 5.25 | 7.46 | 0 | 13.97 | 21.39 |
| **K-562** | 8.36 | 7.27 | 21.95 | 14.94 | 16.19 | 8.26 | 18.64 | 12.72 |
| **MOLT-4** | 19.51 | 24.22 | 34.45 | 21.82 | 20.55 | 9.00 | 22.16 | 35.36 |
| **RPMI-8226** | 35.97 | 24.53 | 14.65 | 24.17 | 42.84 | 23.18 | 95.05 | 35.63 |
| **SR** | 16.41 | 27.01 | 30.61 | 40.27 | 39.58 | 15.20 | 50.25 | 19.54 |
| **Non-Small Cell Lung Cancer** | | | | | | | | |
| **A549/ATCC** | 0 | 0 | 0 | 0.78 | 0 | 0 | 4.52 | 0 |
| **EKVX** | 0 | 8.14 | 3.51 | 0 | 0.51 | 0 | 6.19 | 0 |
| **HOP-62** | 0 | 0 | 0 | 0 | 0 | 0 | 2.12 | 0 |
| **HOP-92** | 1.64 | 0 | 5.88 | 9.74 | 17.75 | 0 | 5.81 | 0 |
| **NCI-H226** | 1.84 | 1.46 | 2.08 | 10.45 | 8.31 | 2.19 | 14.04 | 12.37 |
| **NCI-H23** | 0.22 | 0 | 5.15 | 3.04 | 0 | 0 | 0 | 6.00 |
| **NCI-H322M** | 0 | 4.21 | 1.05 | 0 | 3.77 | 0 | 4.64 | 0 |
| **NCI-H460** | 0 | 0 | 0 | 0 | 0 | 0 | 0 | 0 |
| **NCI-H522** | 1.56 | 1.57 | 14.26 | 3.99 | 7.40 | 0 | 11.61 | 20.86 |
| **Prostate Cancer** | | | | | | | | |
| **PC-3** | 0 | 2.29 | 7.58 | 9.18 | 4.91 | 0 | 12.31 | 17.54 |
| **DU-145** | 0 | 0 | 0 | 0 | 0 | 0 | 0 | 0 |

| **Continued Table S2.** Cell growth inhibition % from the NCI's *in vitro* human tumor cell screen for compounds **7p, 7q, 7r, 7u, 7v and 7f to 7h.** | | | | | | | | |
| --- | --- | --- | --- | --- | --- | --- | --- | --- |
| **Panel/ Cell Lines** | **Compounds** | | | | | | | |
|  | **7p** | **7q** | **7r** | **7u** | **7v** | **7f** | **7g** | **7h** |
| **Colon Cancer** | | | | | | | | |
| **COLO 205** | 0 | 0 | 0 | 0 | 0 | 0 | 0 | 0 |
| **HCC-2998** | 0 | 0 | 0 | 0 | 0 | 0 | 0 | 0 |
| **HCT-116** | 10.31 | 0 | 3.79 | 26.50 | 18.49 | 0 | 22.16 | 6.56 |
| **HCT-15** | 8.17 | 2.92 | 2.32 | 12.26 | 13.11 | 0 | 10.23 | 5.06 |
| **HT29** | 0 | 0 | 0 | 0 | 0 | 0 | 0 | 0 |
| **KM12** | 0.98 | 0 | 0 | 0.98 | 9.06 | 0 | 0.67 | 0.30 |
| **SW-620** | 0 | 0 | 0 | 0 | 0 | 0 | 0 | 0 |
| **CNS Cancer** | | | | | | | | |
| **SF-268** | 0 | 0 | 0 | 0 | 0.81 | 0 | 0.08 | 0 |
| **SF-295** | 5.09 | 1.96 | 3.20 | 2.00 | 2.25 | 0 | 7.04 | 0 |
| **SF-539** | 16.36 | 16.20 | 7.36 | 13.32 | 25.26 | 2.10 | 15.71 | 5.67 |
| **SNB-19** | 0 | 0 | 0 | 0 | 6.59 | 0 | 0.54 | 0 |
| **SNB-75** | 9.72 | 0 | 1.88 | 13.83 | 14.72 | 1.75 | 9.09 | 1.47 |
| **U251** | 6.84 | 0 | 0 | 9.89 | 22.43 | 3.23 | 17.80 | 3.98 |
| **Melanoma** | | | | | | | | |
| **LOX IMIV** | 111.79 | 96.83 | 20.17 | 22.04 | 131.58 | 22.93 | 124.15 | 93.21 |
| **MALME-3M** | 2.95 | 4.86 | 8.71 | 2.82 | 0 | 3.93 | 0 | 6.52 |
| **M14** | 0 | 0 | 0 | 0 | 0 | 0 | 0 | 0 |
| **MDA-MB-435** | 0 | 0 | 0 | 6.83 | 1.02 | 0 | 0.11 | 1.94 |
| **SK-MEL-2** | 0 | 0 | 0 | 0 | 0 | 0 | 0 | 0 |
| **SK-MEL-28** | 0 | 0 | 0 | 0 | 0 | 0 | 0 | 0 |
| **SK-MEL-5** | 4.49 | 2.38 | 10.83 | 5.99 | 8.57 | 0.99 | 6.32 | 10.26 |
| **UACC-257** | 0 | 0 | 0 | 0 | 0 | 0 | 0 | 0 |
| **UACC-62** | 4.99 | 1.95 | 11.02 | 8.01 | 16.01 | 5.12 | 16.80 | 16.23 |
| **Table 2(continuous).** Cell growth inhibition % from the NCI's *in vitro* human tumor cell screen for compounds **7p, 7q, 7r, 7u, 7v and 7f to 7h.** | | | | | | | | |

| **Panel/Cell Line** | **Compounds** | | | | | | | |
| --- | --- | --- | --- | --- | --- | --- | --- | --- |
|  | **7p** | **7q** | **7r** | **7u** | **7v** | **7f** | **7g** | **7h** |
| **Ovarian Cancer** | | | | | | | | |
| **IGROV1** | 3.63 | 8.10 | 2.07 | 29.00 | 18.43 | 0 | 14.45 | 4.69 |
| **OVCAR-3** | 0 | 0 | 0 | 0 | 2.13 | 0 | 0 | 0 |
| **OVCAR-4** | 0 | 0 | 0 | 0 | 0 | 0 | 0 | 0 |
| **OVCAR-5** | 0.55 | 4.80 | 0 | 0 | 0.76 | 0 | 0 | 0 |
| **OVCAR-8** | 0 | 0 | 11.10 | 10.62 | 7.52 | 0 | 2.88 | 20.51 |
| **NCI/ADR-RES** | 0 | 0 | 0 | 0 | 0 | 0 | 0 | 0 |
| **SK-OV-3** | 0 | 0 | 0 | 0 | 0 | 0 | 0 | 0 |
| **Renal Cancer** | | | | | | | | |
| **786-0** | 0.75 | 0 | 0 | 0 | 9.2 | 0 | 7.38 | 0.41 |
| **A498** | 0 | 0 | 0 | 0 | 16.79 | 0 | 9.85 | 0 |
| **ACHN** | 3.66 | 5.74 | 2.15 | 1.08 | 3.22 | 0 | 1.98 | 0 |
| **CAKI-1** | 2.96 | 0 | 0.08 | 0 | 2.81 | 1.83 | 16.36 | 2.63 |
| **RXF 393** | 0 | 0 | 0 | 0 | 0 | 0 | 0 | 0 |
| **SN 12C** | 5.25 | 5.10 | 6.98 | 13.15 | 9.61 | 0 | 15.11 | 6.65 |
| **TK-10** | 0 | 0 | 0 | 0 | 0 | 0 | 0 | 0 |
| **UO-31** | 11.48 | 12.99 | 12.39 | 21.01 | 15.99 | 12.22 | 24.68 | 12.54 |
| **Breast Cancer** | | | | | | | | |
| **MCF7** | 21.71 | 24.14 | 24.69 | 44.70 | 40.92 | 18.35 | 44.78 | 20.32 |
| **MDA-MB-231/ATCC** | 8.78 | 4.84 | 4.13 | 4.05 | 8.40 | 2.18 | 9.38 | 1.61 |
| **HS 578T** | 3.94 | 3.98 | 13.23 | 9.37 | 12.08 | 0 | 11.03 | 5.34 |
| **BT-549** | 5.86 | 0 | 5.47 | 28.87 | 15.62 | 0 | 0.37 | 15.01 |
| **T-47D** | 13.05 | 10.55 | 28.88 | 14.77 | 19.64 | 0 | 12.92 | 22.00 |
| **MDA-MB-468** | 0 | 0 | 0 | 0 | 6.96 | 0 | 0 | 0 |
|  | | | | | | | | |

| **Docking poses EGFR** | **No** | **Docking poses EGFR** | **No** |
| --- | --- | --- | --- |
| 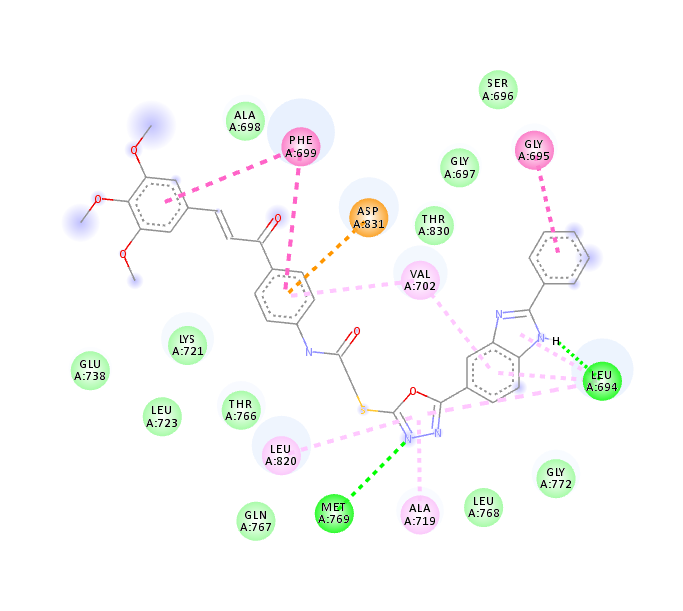 | **7e** | 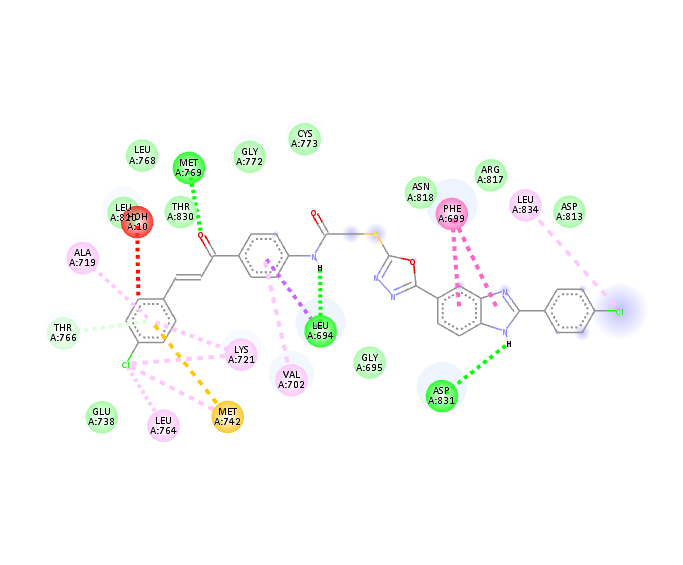 | **7g** |
|  | **7h** |  | **7k** |
|  | **7l** |  | **7m** |
|  | **7n** |  | **7p** |
|  | **7q** |  |  |

| **Docking poses BRAF** | **NO** | **Docking poses BRAF** | **NO** |
| --- | --- | --- | --- |
|  | **7e** |  | **7g** |
|  | **7h** |  | **7k** |
|  | **7l** |  | **7m** |
|  | **7n** |  | **7p** |
|  | **7q** |  |  |
