# Supplementary material for: Chalcone/1,3,4-Oxadiazole/Benzimidazole hybrids as novel anti-proliferative agents inducing apoptosis and inhibiting EGFR & BRAFV600E
Source: BMC Chem. 2023 Sep 16;17(1):116. doi: 10.1186/s13065-023-01003-3 (PMC10504751; doi:10.1186/s13065-023-01003-3)
Supplement: Supplementary file 2 — Supplementary Material 2 [file 13065_2023_1003_MOESM2_ESM.docx]

**Chalcone/1,3,4-Oxadiazole/Benzimidazole Hybrids as Novel Anti-proliferative Agents Inducing Apoptosis and Inhibiting EGFR & BRAFV^600E^**

Fatma Hagar^1^ | Samar Abbas^1^ | Hesham Gomaa^2^ | Bahaa Youssif^3^ | Ahmed Sayed^4^ | Dalia Abdelhamid*****^1^ | Mohamed Abdel-Aziz^1^

^1^ Medicinal Chemistry Department, Faculty of Pharmacy, Minia University, Minia, Egypt

^2^ Pharmacology Department, College of Pharmacy, Jouf University, Sakaka, Saudi Arabia

^3^ Pharmaceutical Organic Chemistry Department, Faculty of Pharmacy, Assiut University, Assiut, Egypt

^4^ Pharmacognosy Department, Faculty of Pharmacy, Nahda University, Benisuef, Egypt

*** Corresponding Author:**

Dalia Abdelhamid; e-mail: dalia_abdelhameed@mu.edu.eg

**4. EXPERIMENTAL**

**4.1. Chemistry**

**4.1.1. General procedure for the synthesis of (*E*) (1-(4-aminophenyl)-3-arylprop-2-en-1-one 5a-e.**^1^

An equimolar amount of p-aminoacetophenone (13.51 gm, 0.1 mol) and the appropriate aldehyde (0.1mol), were dissolved in a minimum amount of ethanol, aqueous KOH (0.25 mol, 60%) was added dropwise. The reaction mixture was stirred in an ice bath for 30 min then at rt until a precipitate was formed within 3 h. The precipitate was filtered off and washed thoroughly with cold distilled water and cold methanol (2x20 mL). The product was recrystallized from absolute ethanol. The structure of the product was confirmed by comparing their melting point with the reported mp.

| **Compound** | **Yield (%)** | **m.p (^o^C)** | **Lit.mp (^o^C)** |
| --- | --- | --- | --- |
| (*E*)1-(4-Aminophenyl)-3-phenylprop-2-en-1-one 8a | 79 | 154-155 | 156-157^1,2^ |
| (*E*)1-(4-Aminophenyl)-3-(4-chlorophenyl)prop-2-en-1-one 8b | 75 | 159-160 | 158-159^3^ |
| (*E*)1-(4-Aminophenyl)-3-(4-methoxyphenyl)prop-2-en-1-one 8c | 73 | 115-116 | 114-116^4,5^ |
| (*E*)1-(4-Aminophenyl)-3-(3,4dimethoxyphenyl)prop-2-en-1-one 8d | 78 | 163-165 | 158-160^3,4^ |
| (*E*)1-(4-Aminophenyl)-3-(3,4,5-trimethoxyphenyl)prop-2-en-1-one 8e | 80 | 164-166 | 166-168^2^ |

**4.1.3. General procedure for synthesis of** **2-Bromo-*N*-(4-((*E*)-3-arylacryloyl)phenyl)acetamides 3a-e and 4-((*E*)-3-arylacryloyl)phenyl 2-bromoacetates 6a-e.**^2^

To a stirred mixture of the appropriate chalcones **5a-e** (6.30mmol) in dichloromethane (20mL) and potassium carbonate (0.18gm, 1.302mmol) in 100 mL water in an ice bath. Bromo acetyl bromide (1.856g, 9.20mmol) or chloroacetyl chloride (9.20mmol) in 30 mL dichloromethane was added in a dropwise manner with stirring over 30 min. Stirring was continued for 2h at 0ᴼC and at rt overnight. The reaction mixture was extracted with dichloromethane (2x60 mL) and the organic layer was washed with distilled water (2x40 mL), dried over anhydrous sodium sulphate, filtered, evaporated under vacum and the residue was recrystallized from ethanol.

| **Compound** | **Yield (%)** | **m.p (^o^C)** | **Lit.mp (^o^C)** |
| --- | --- | --- | --- |
| 2-Bromo-*N*-(4-((*E*)-3-phenylacryloyl)phenyl)acetamide 5a | 82 | 157-159 | 157-159^2^ |
| (*E*)-2-Bromo-*N*-(4-(3-(4-chlorophenyl)acryloyl)phenyl)acetamide 5b | 80 | 194-196 | 191-192^2^ |
| (*E*)-2-Bromo-*N*-(4-(3-(4-methoxyphenyl)acryloyl)phenyl)acetamide 5c | 78 | 153-155 | 155-156^2^ |
| (*E*)-2-Bromo-*N*-(4-(3-(3,4-dimethoxyphenyl)acryloyl)phenyl)acetamide 5d | 80 | 150-153 | 149-150^1^ |
| (*E*)-2-Bromo-*N*-(4-(3-(3,4,5-trimethoxyphenyl)acryloyl)phenyl)acetamide 5e | 77 | 169-171 | 166-167^2^ |

**4.1.4.** **General procedure for the synthesis of the adduct** ^6^

Benzaldehyde derivative was dissolved in EtOH (50 mL) in ice bath. Sodium meta bisulfite (1.6gm) was dissolved in cooled water (10 mL). Solution of sodium meta bisulfite was added drop wisely to the benzaldehyde solution with vigorous stirring. The precipitate that formed was filtered off and dried in vacuo.

**4.1.3.** **General procedure for the synthesis of substituted phenyl-1*H*-benzo[*d*]imidazole-5-carboxylic acid 1a-e.**^6^

**1a-e**

A mixture of the appropriate adduct (2 mmol) and 3,4-di-amino benzoic acid (2 mmol) in DMF (5 mL) was heated under reflux for (3-6) h after which the reaction mixture was cooled, poured into ice water. The formed solid was collected and recrystallized from the appropriate solvent.

| **Compound** | **Yield (%)** | **m.p (^o^C)** | **Lit.mp (^o^C)** |
| --- | --- | --- | --- |
| 2-Phenyl-1*H*-benzo[*d*]imidazole-5-carboxylic acid | 83 | >300 | >300^6^ |
| 2-(4-Chlorophenyl)-1*H*-benzo[*d*]imidazole-5-carboxylic acid | 80 | 180-181 | 180-181^6^ |
| 2-(4-Methoxyphenyl)-1*H*-benzo[*d*]imidazole-5-carboxylic acid | 78 | 244-246 | 244-246^6^ |
| 2-(3,4-Dimethoxyphenyl)-1*H*-benzo[*d*]imidazole-5-carboxylic acid | 69 | 215-217 | 215-217^6^ |
| 2-(3,4,5-Trimethoxyphenyl)-1*H*-benzo[*d*]imidazole-5-carboxylic acid | 66 | 275-277 | 275-277^6^ |

**4.1.4.** **General procedure for the synthesis of substituted** **ethyl 2-phenyl-1*H*-benzo[*d*]imidazole-5-carboxylate (2a-e).**^6^

**2a-e**

A mixture of the appropriate substituted phenyl-1H-benzo[d]imidazole-5-carboxylic acid (0.1 mol) and 2 mL of concentrated H_2_SO_4_ in 100 mL of absolute methanol was heated under reflux for 20 h. The reaction mixture was concentrated under vacuum, then washed with saturated NaHCO_3_ (2 X 20 mL). The resulting solid was filtered off, dried to give the methyl ester derivatives **2a-e**, and used for following step.

| **Compound** | **Yield(%)** | **m.p (^O^C)** | **Lit.mp (^O^C)** |
| --- | --- | --- | --- |
| Ethyl-2-phenyl-1H-benzo[d]imidazole-5-carboxylate | 71 | 183-184 | 183-184^6^ |
| Ethyl-2-(4-chlorophenyl)-1H-benzo[d]imidazole-5-carboxylate | 78 | >300 | >300^6^ |
| Ethyl-2-(4-methoxyphenyl)-1H-benzo[d]imidazole-5-carboxylate | 68 | 202-205 | 202-205^6^ |
| Ethyl-2-(3,4-dimethoxyphenyl)-1H-benzo[d]imidazole-5-carboxylate | 69 | 230-232 | 230-232^6^ |
| Ethyl-2-(3,4,5-trimethoxyphenyl)-1H-benzo[d]imidazole-5-carboxylate | 74 | 260 | 260^6^ |

**4.1.5.** **General procedure for the synthesis of** **substituted 5-hydrazineyl-2-phenyl-1H-benzo[d]imidazole(3a-c).**^6^

A mixture of the appropriate substituted methyl 4-(1H-benzo[d]imidazol-2-ylbenzoate **3a-c** (0.1 mol) hydrazine monohydrate 99% (0.25 mol), was heated under reflux for 6 h. Hydrazine monohydrate was removed under vacuum and the residue was poured into 200 mL of cold water. The formed solid was collected, washed with ice-cold water and recrystallized from 95% ethanol.

| **Compound** | **Yield(%)** | **m.p (^o^C)** | **Lit.mp(^o^C)** |
| --- | --- | --- | --- |
| 2-Phenyl-1H-benzo[d]imidazole-5-carbohydrazide | 73 | 247-249 | 247-249^6^ |
| 2-(4-Chlorophenyl)-1H-benzo[d]imidazole-5-carbohydrazide | 70 | 250-252 | 250-252^6^ |
| 2-(4-Methoxyphenyl)-1H-benzo[d]imidazole-5-carbohydrazide | 80 | 257-259 | 257-259^6^ |
| 2-(3,4-Dimethoxyphenyl)-1H-benzo[d]imidazole-5-carbohydrazide | 72 | 230-231 | 230-231^6^ |
| 2-(3,4,5-Trimethoxyphenyl)-1H-benzo[d]imidazole-5-carbohydrazide | 68 | 264-266 | 264-266^6^ |

**4.1.6.** **General procedure for the synthesis of** **substitute** **5-(2-phenyl-1*H*-benzo[*d*]imidazol-5-yl)-1,3,4-oxadiazole-2-thiol(4a-e)^.^**^5^**^,^**^6^

Equimolar quantities of 5-substituted ethyl 4-(1H-benzo[d]imidazol-2-yl)benzohydrazides **3a-e** (0.05mol) and KOH (0.05mol) refluxed with carbon di sulfide (0.17 mol, 10mL) in ethanol 70ml for (12h) until evaluation of H_2_S gas stopped. The reaction mixture was concentrated under vacuum. The formed solid was collected and dissolved in H_2_O then acidified with 10% HCl until pH 2. the solid was collected and and washed with water and recrystallized with an appropriate solvent**.**

| **Compound** | **Yield (%)** | **m.p (^O^C)** |
| --- | --- | --- |
| 5-(2-Phenyl-1H-benzo[d]imidazol-5-yl)-1,3,4-oxadiazole-2-thiol | 81 | 270-272 as reported^6^ |
| 5-(2-(4-Chlorophenyl)-1H-benzo[d]imidazol-5-yl)-1,3,4-oxadiazole-2-thiol | 76 | 253-255 as reported^6^ |
| 5-(2-(4-Methoxyphenyl)-1H-benzo[d]imidazol-5-yl)-1,3,4-oxadiazole-2-thiol | 80 | 284-286 as reported^6^ |
| 5-(2-(3,4-Dimethoxyphenyl)-1H-benzo[d]imidazol-5-yl)-1,3,4-oxadiazole-2-thiol | 74 | 283-285 as reported^6^ |
| 5-(2-(3,4,5-Trimethoxyphenyl)-1H-benzo[d]imidazol-5-yl)-1,3,4-oxadiazole-2-thiol | 70 | 232-234 as reported^6^ |

**4.2. Biological evaluation**

**4.2.2. Cytotoxic activity using MTT Assay and Determination of IC_50_**

**4.2.2.1. MTT assay**

MTT assay was carried out to study the effect of compounds on mammary epithelial cells (MCF-10A).^7, 8^ The medium in which cells were propagated contained Dulbecco's modified Eagle's medium (DMEM)/ Ham's F-12 medium (1:1) supplemented with epidermal growth factor (20 ng/mL), hydrocortisone (500 ng/mL), insulin (10 μg/mL), 2 mM glutamine and 10% foetal calf serum. After every 2-3 days, the cells were passaged using trypsin ethylenediamine tetra acetic acid (EDTA). The cells were seeded at a density of 10^4^ cells mL^-1^ in flat-bottomed culture plates containing 96 wells each. After 24 h, medium was removed from the plates and the compounds in (in 0.1% DMSO) were added (in 200 μL medium to yield a final concentration of 0.1% v/v) to the wells of plates. A single compound was designated with four wells followed by incubation of plates for 96h at 37°C. After incubation, medium was removed completely from the plates followed by addition of MTT (0.4 mg/mL in medium) to each well and subsequent incubation of plates for 3h. MTT (along with the medium) was removed and DMSO (150μL) was added to each well of the culture plates, followed by vortexing and subsequent measurement of absorbance (at 540 nm) using microplate reader. The data are shown as percentage inhibition of proliferation in comparison with controls containing 0.1% DMSO.

**4.2.2.2. Assay for antiproliferative effect**

To explore the antiproliferative potential of compounds propidium iodide fluorescence assay was performed using different cell lines (melanoma cancer cell line LOX-IMVI, pancreatic cancer cell line (Panc-1), epithelial line cancer cell (A-549), breast cancer cell line (MCF-7), and colon cancer cell line (HT-29). To calculate the total nuclear DNA, a fluorescent dye (propidium iodide, PI) is used which can attach to the DNA,^9,10^ thus offering a quick and precise technique. PI cannot pass through the cell membrane and its signal intensity can be considered as directly proportional to the quantity of cellular DNA. Cells whose cell membranes are damaged or have changed permeability are counted as dead ones. The assay was performed by seeding the cells of different cancer cell lines at a density of 3000-7500 cells/well (in 200µl medium) in culture plates followed by incubation for 24h at 37 °C in humidified 5% CO_2_/95% air atmospheric conditions. The medium was removed; the compounds were added to the plates at 10 µM concentrations (in 0.1% DMSO) in triplicates, followed by incubation for 48 h. DMSO (0.1%) was used as control. After incubation, medium was removed followed by the addition of PI (25 µl, 50µg/mL in water/medium) to each well of the plates. At -80 °C, the plates were allowed to freeze for 24 h, followed by thawing at 25^o^C. A fluorometer (Polar-Star BMG Tech) was used to record the readings at excitation and emission wavelengths of 530 and 620 nm for each well. The percentage cytotoxicity of compounds was calculated using the following formula:


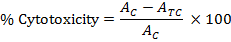


Where A*_TC_*= Absorbance of treated cells and A_C_= Absorbance of control. Staurosporine (with LOX-IMVI) and doxorubicin (in Panc-1, A-549, MCF-7, and HT-29) were used as positive control in the assays.

**4.2.2.3. EGFR inhibitory assay**

EGFR-TK assay was performed to evaluate the inhibitory potency of novel compounds**; 7e**, **7g**, **7h**, **7k-n**, **7p**, **7q**, and **7v** against EGFR^.11,12^ Baculoviral expression vectors including pBlueBacHis2B and pFASTBacHTc were used separately to clone 1.6 kb cDNA coding for EGFR cytoplasmic domain (EGFR-CD, amino acids 645–1186). 5ʹ upstream to the EGFR sequence comprised a sequence that encoded (His)_6_. Sf-9 cells were infected for 72h for protein expression. The pellets of Sf-9 cells were solubilized in a buffer containing sodium vanadate (100 µM), aprotinin (10 µg/mL), triton (1%), HEPES buffer (50mM), ammonium molybdate (10 µM), benzamidine HCl (16 µg/mL), NaCl (10 mM), leupeptin (10 µg/mL) and pepstatin (10 µg/mL) at 0°C for 20 min at pH 7.4, followed by centrifugation for 20 min. To eliminate the non-specifically bound material, a Ni-NTA super flow packed column was used to pass through and wash the crude extract supernatant first with 10 mM and then with 100 mM imidazole. Histidine-linked proteins were first eluted with 250 and then with 500 mM imidazole subsequent to dialysis against NaCl (50 mM), HEPES (20 mM), glycerol (10%) and 1 µg/mL each of aprotinin, leupeptin and pepstatin for 120 min. The purification was performed either at 4 °C or on ice. To record autophosphorylation level, EGFR kinase assay was carried out on the basis of DELFIA/Time-Resolved Fluorometry. The compounds were first dissolved in DMSO absolute, subsequent to dilution to appropriate concentration using HEPES (25 mM) at pH 7.4. Each compound (10 µL) was incubated with recombinant enzyme (10 µL, 5 ng for EGFR, 1:80 dilution in 100 mM HEPES) for 10 min at 25^o^C, subsequent to the addition of 5X buffer (10 µL, containing 2 mM MnCl_2_, 100 µM Na_3_VO_4_, 20 mM HEPES and 1 mM DTT) and ATP-MgCl_2_ (20 µL, containing 0.1 mM ATP and 50 mM MgCl_2_) and incubation for 1h. The negative and positive controls were included in each plate by the incubation of enzyme either with or without ATP-MgCl_2_. The liquid was removed after incubation and the plates were washed thrice using wash buffer. Europium-tagged antiphosphotyrosine antibody (75 µL, 400 ng) was added to each well followed by incubation of 1h and then plates were washed using buffer. Enhancement solution was added to each well and the signal was recorded at excitation and emission wavelengths of 340 at 615 nm respectively. The autophosphorylation percentage inhibition by compounds was calculated using the following equation:


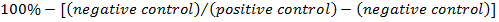


Using the curves of percentage inhibition of eight concentrations of each compound, IC_50_ was calculated. Majority of signals detected by antiphosphotyrosine antibody were from EGFR because the enzyme preparation contained low impurities.

**4.2.2.4. BRAF kinase assay**

V^600E^ mutant BRAF kinase assay was performed to investigate the activity of compounds **7e**, **7g**, **7h**, **7k-n**, **7p**, **7q**, and **7v** against BRAF.^13,14^ Mouse full-length GST-tagged BRAF^V600E^ (7.5 ng, Invitrogen, PV3849) was pre-incubated with drug (1 µL) and assay dilution buffer (4 µL) for 60 min at 25^o^C. In assay dilution buffer, a solution (5 µL) containing MgCl_2_ (30 mM), ATP (200 µM), recombinant human full length (200 ng) and *N*-terminal His-tagged MEK1 (Invitrogen) was added to start the assay, subsequent to incubation for 25 min at 25^o^C. The assay was stopped using 5X protein denaturing buffer (LDS) solution (5 µL). To further denature the protein, heat (70° C) was applied for 5 min. 4-12% precast NuPage gel plates (Invitrogen) were used to carry out electrophoresis (at 200 V). 10 µL of each reaction was loaded into the precast plates and electrophoresis was allowed to proceed. After completion of electrophoresis, the front part of the precast gel plate (holding hot ATP) was cut and afterwards cast-off. The dried gel was developed using a phosphor screen. A reaction without active enzyme was used as negative control while that containing no inhibitor served as positive control. To study the effect of compounds on cell-based pERK1/2 activity in cancer cells, commercially available ELISA kits (Invitrogen) were used according to manufacturer’s instructions.

**4.2.2.5 Caspases assays**

**a) Caspase-3 activation assay**

All reagents were allowed to reach room temperature before use. All liquid reagents were gently mixed before use. The number of 8-well strips needed for the assay was determined. These were inserted into the frame(s) for current use. A total of 100 µL of the Standard Diluent Buffer was added to the zero standard wells. Well(s) reserved for chromogen blank were left empty. A total of 100 µL of standards and controls or diluted samples was added to the appropriate microtiter wells. The sample dilution chosen was optimized for each experimental system. The side of the plate was gently tapped on to

mix. Wells were covered with a plate cover and incubated for 2 h at room temperature. The solution was thoroughly aspirated or decanted from the wells and the liquid was discarded; wells were washed four times. A total of 100 µL of Caspase 3 (Active) Detection Antibody solution was pipetted into each well except the chromogen blank(s). The side of the plate was gently tapped on to mix. The plate was covered with a plate cover and incubated for 1h at room temperature. The solution was thoroughly aspirated or decanted from wells and the liquid was discarded; wells were washed four times. A total of 100 µL Anti-Rabbit IgG HRP Working Solution was added to each well except the chromogen blank(s). The working dilution was prepared as described in Preparing IgG HRP. Wells were covered with the plate cover and incubated for 30 min at room temperature. The solution was thoroughly aspirated or decanted from the wells and the liquid was discarded. Wells were washed four times. A total of 100 µL of Stabilized Chromogen was added to each well. The liquid in the wells began to turn blue. It was incubated for 30 min at room temperature and in the dark. The incubation time for chromogen substrate was determined by the microtiter plate reader used. Many plate readers have the capacity to record a maximum optical density (O.D.) of 2.0. The O.D. values were monitored, and the substrate reaction was stopped before the O.D. of the positive wells exceeds the limits of the instrument. The O.D. values at 450 nm could only be read after the Stop Solution had been added to each well. A total of 100 µL of Stop Solution was added to each well. The side of the plate was gently tapped to mix. The solution in the wells changed from blue to yellow. The absorbance of each well was read at 450 nm, having blanked the plate reader against a chromogen blank composed of 100 µL each of Stabilized Chromogen and Stop Solution. The plate was read within 2 h after adding the Stop Solution. A curve fitting software was used to generate the standard curve.

A four-parameter algorithm provided the best standard curve fit. The concentrations for unknown samples and controls from the standard curve were read. Value(s) obtained for sample(s) by the appropriate dilution factor were multiplied to correct for the dilution in step 3. Samples producing signals greater than that of the highest standard were diluted in Standard Diluent Buffer and reanalyzed.
**b) Caspase-8 activation assay**

Cells were obtained from American Type Culture Collection, cells were grown in RPMI 1640 containing 10% fetal bovine serum at 37°C, stimulated with the compounds to be tested for caspase8, and lysed with Cell Extraction Buffer. This lysate was diluted in Standard Diluent Buffer over the range of the assay and measured for human active caspase-8 content. (*Cells are Plated in a density of 1.2 – 1.8 × 10,000 cells/well in a volume of 100µl complete growth medium + 100 ul of the tested compound per well in a 96-well plate for 24 hours before the enzyme assay for Tubulin*.). The absorbance of each microwell was read on a spectro-photometer at 450 nm. A standard curve is prepared from 7human Caspase-8 standard dilutions and human Caspase-8 concentration determined.

**4.2.2.6 Cytochrome C Assay**

Cells were obtained from American Type Culture Collection, cells were grown in RPMI 1640 containing 10% fetal bovine serum at 37 °C, stimulated with the compounds to be tested for cytochrome c, and lysed with Cell Extraction Buffer. This lysate was diluted in Standard Diluent Buffer over the range of the assay and measured for cytochrome c content (cells are plated in cells/well in a volume of 100 μL complete growth medium + 100 μL of the tested compound + 50 μL of 1× biotin-conjugated antibody + 100 μL of 1× streptavidin-HRP + 100 μL TMB substrate solution of per well in a 96-well plate for 24 hr before assay.

**4.2.2.7 Expression levels of BAX and Bcl-2 proteins**

**a) BAX activation assay**

All reagents were brought to room temperature for at least 30 min before opening (except the human BAX-α Standard). Human BAX-α Standard solution was not be left at room temperature for more than 10 min. Duplicates of all standards, controls, and samples were run. The Assay Layout Sheet was used to determine the number of wells to be used, and any remaining wells were put with the desiccant back into the pouch, and the ziplock was sealed. Unused wells were stored at 4 ◦C. A total of 100 µL of Assay Buffer was pipetted into the S0 (0 pg/mL standard) wells. A total of 100 µL of Standards #1 through #6 was pipetted into the appropriate wells. A total of 100 µL of the samples was pipetted into the appropriate wells. The plate was gently tapped to mix the contents. The plate was sealed and incubated at room temperature on a plate shaker for 1 h at ~500 rpm. The contents of the wells were emptied and washed by adding 400 µL of wash solution to every well. The wash was repeated four more times for a total of five washes. After the final wash, the wells were emptied or aspirated and firmly the plate on a lint-free paper towel was tapped to remove any remaining wash buffer. A total of 100 µL of yellow antibody was pipetted into each well, except the Blank. The plate was sealed and incubated at room temperature on a plate shaker for 1 h at ~500 rpm. The contents of the wells were emptied and washed by adding 400 µL of wash solution to every well. The wash was repeated four more times for a total of five washes. After the final wash, the wells were emptied or aspirated, and the plate was firmly tapped on a lint-free paper towel to remove any remaining wash buffer. A total of 100 µL of blue Conjugate was added to each well, except the Blank. The plate was sealed and incubated at room temperature on a plate shaker for 30 min at ~500 rpm. The contents of the wells were emptied and washed by adding 400 µL of wash solution to every well. The wash was repeated four more times for a total of five washes. After the final wash, the wells were emptied or aspirated and the plate was firmly tapped on a lint-free paper towel to remove any remaining wash buffer. A total of 100 µL of Substrate Solution was pipetted into each well. It was incubated for 30 min at room temperature on a plate shaker at ~500 rpm. A total of 100 µL Stop Solution was pipetted into to each well. The plate reader was blanked against the Blank wells, and the optical density was read at 450 nm. The average net optical density (OD) bound for each standard and sample was calculated by subtracting the average Blank OD from the average OD for each standard and sample. Using linear graph paper, the Average Net OD for each standard versus BAX concentration in each standard were plotted. A straight line was approximated through the points. The concentration of BAX in the unknowns could be determined by interpolation.

**b) Bcl-2 inhibition assay**

All reagents were thoroughly mixed without foaming before use. The microwells were washed twice with approximately 300 µL Wash Buffer per well with the thorough aspiration of microwell contents between washes. Care was taken not to scratch the surface of the microwells. After the last wash, the wells were emptied and microwell strips were tapped on an absorbent pad or paper towel to remove excess Wash Buffer. The microwell strips were used immediately after washing or placed upside down on a wet absorbent paper for not longer than 15 min. Wells were not allowed to dry. A total of 100 µL of Sample Diluent was added in duplicate to all standard wells and the blank wells. Standard (1:2 dilution) in duplicate was prepared ranging from 32 to 0.5 ng/mL. A total of 100 µL of Sample Diluent, in duplicate, was added to the blank wells. A total of 80 µL of Sample Diluent, in duplicate, was added to the sample wells. A total of 20 µL of each sample, in duplicate, was added to the designated wells. A total of 50 µL of diluted biotin was added, conjugated to all wells, including the blank wells. It was covered with a plate cover and incubated at room temperature on a microplate shaker at 100 rpm for 2 h. The plate cover was removed and the wells were emptied. Microwell strips were washed times as described in Step 2. A total of 100 µL of diluted Streptavidin-HRP was added to all wells, including the blank wells. It was covered with a plate cover and incubated at room temperature on a microplate shaker at 100 rpm for 1 h. The plate cover was removed and the wells were emptied. Microwell strips were washed three times, as described in Step 2. A total of 100 µL of mixed TMB Substrate Solution was pipetted into all wells, including the blanks. The microwell strips were incubated at room temperature (18 ◦C to 25 ◦C) for about 15 min, on a rotator set at 100 rpm. Direct exposure to intense light was avoided. The point at which the substrate reaction is stopped was determined by the ELISA reader. Many ELISA readers record absorbance only up to 2.0 O.D. Therefore, the color development within individual microwells was watched by the person running the assay, and the substrate reaction was stopped before positive wells were no longer properly detectable. The enzyme reaction was stopped by quickly pipetting 100 µL of Stop Solution into each well, including the blank wells. It was important that the Stop Solution was spread quickly and uniformly throughout the microwells to inactivate the enzyme completely. Results were read immediately after the Stop Solution was added or within one hour if the microwell strips were stored at 2–8 ◦C in the dark. The absorbance of each microwell was read on a spectrophotometer using 450 nm as the primary wavelength.

**4.2.2.8.**  **Cell apoptosis assay**

Apoptosis was determined by flow cytometry based on the Annexin-V-fluoresce in isothiocyanate (FITC) and propidium iodide (PI) staining kit (BD Pharmingen, San Diego, USA.^15,16^ Apoptosis was determined by flow cytometry based on the Annexin-V-fluoresce in isothiocyanate (FITC) and propidium iodide (PI) staining kit (BD Pharmingen, San Diego, USA). Apoptotic cells were defined as Annexin-V-positive. Cells were grown to approximately ∼70% confluence and exposed to different concentrations of compounds (0, 2, 4, 6 and8 μmol/L) for 24 h. Treated cells were trypsinized, washed twice with PBS and transferred into micro centrifuge tubes for centrifugation at1000 rpm for 5 min at room temperature, then resuspended in binding buffer, 5 μL of FITC and PI were added to per eppendorf tube, cells were vortexed, incubated for 15 min at room temperature in dark. Subsequently, cells were analyzed by flow cytometry (Becton Dickinson, Franklin Lakes, and USA).

**4.2.3. Statistical analysis**

Computerized Prism 5 program was used to statistically analyzed data using one-way ANOVA test followed by Tukey’s as post ANOVA for multiple comparison at P ≤.05. Data were presented as mean ± SEM.^17^

**4.3. Docking based virtual screening**

**4.3.1. Ligand Structure Generation**

OpenBabel v.3.sed to convert the structures’ SMILE codes to three-dimensional configurations that were subsequently subjected to a minimization of energy using the steepest descent technique with the same software.^28^ The minimization was performed by the force field MMFF94. Using AutoDockTools v.4.2, all torsions of the selected structures were assigned and their Gasteiger charges were provided for all studied atoms in structures.^17^

**4.3.2. Protein Structure Preparation**

For docking screening, the human EGFR and BRAF crystal structures (PDB codes: 1M17 and 3OG7, respectively) were used.^18,19^ PDBfixer was used to edit the downloaded structure, adding missing residues and atoms, and removing co-crystalized H_2_O and heteroatoms.^18^ Through AutoDock Tools v.4.2, polar hydrogen and Gasteiger charges were subsequently made available for both proteins.

**4.3.3. Structural Docking**

The docking process was carried out using the PyRx platform's built-in AutoDock Vina software. According to the co-crystalized ligands of both enzymes, the docking search grid boxes were determined to perfectly enclose them with a 20 Å^3^ total size. ^20, 21^

The grid box's coordinates were set to be x = -10.857; y = 22.836; z = -16.347 and x = 33.992; y = 65.846; z = 24.351, respectively. Pymol software was used to evaluate and display docking poses. Exhaustiveness was set to 24. Ten poses were generated for each docking experiment. Docking poses were analyzed and visualized using Pymol software.^22^ The docking protocol was validated by re-docking the co-crystalized ligands (i.e. Erlotinib and Vemurafenib, respectively) into the active sites of both enzymes (i.e. EGFR and BRAF, respectively). The resulting top-scoring poses of both ligands were in good alignment with the co-crystalized ones with slight deviations (RMSDs = 1.143 and 1.224 Å, respectively).

**References**

(1) Abdel-Aziz, M.; Park, S.-E.; Abuo-Rahma, G. E.-D. A. A.; Sayed, M. A.; Kwon, Y. Novel N-4-Piperazinyl-Ciprofloxacin-Chalcone Hybrids: Synthesis, Physicochemical Properties, Anticancer and Topoisomerase I and II Inhibitory Activity. *Eur. J. Med. Chem.* **2013**, *69*, 427–438. https://doi.org/10.1016/j.ejmech.2013.08.040.

(2) Ahmed, F. F.; Abd El-Hafeez, A. A.; Abbas, S. H.; Abdelhamid, D.; Abdel-Aziz, M. New 1, 2, 4-Triazole-Chalcone Hybrids Induce Caspase-3 Dependent Apoptosis in A549 Human Lung Adenocarcinoma Cells. *Eur. J. Med. Chem.* **2018**, *151*, 705–722.

(3) Mourad, M. A. E.; Abdel-Aziz, M.; Abuo-Rahma, G. E.-D. A. A.; Farag, H. H. Design, Synthesis and Anticancer Activity of Nitric Oxide Donating/Chalcone Hybrids. *Eur. J. Med. Chem.* **2012**, *54*, 907–913. https://doi.org/10.1016/j.ejmech.2012.05.030.

(4) Ansari, F. L.; Umbreen, S.; Hussain, L.; Makhmoor, T.; Nawaz, S. A.; Lodhi, M. A.; Khan, S. N.; Shaheen, F.; Choudhary, M. I.; Atta-ur-Rahman. Syntheses and Biological Activities of Chalcone and 1,5-Benzothiazepine Derivatives: Promising New Free-Radical Scavengers, and Esterase, Urease, and α-Glucosidase Inhibitors. *Chem. Biodivers.* **2005**, *2* (4), 487–496. https://doi.org/10.1002/cbdv.200590029.

(5) Fathi, M. A. A.; Abd El-Hafeez, A. A.; Abdelhamid, D.; Abbas, S. H.; Montano, M. M.; Abdel-Aziz, M. 1,3,4-Oxadiazole/Chalcone Hybrids: Design, Synthesis, and Inhibition of Leukemia Cell Growth and EGFR, Src, IL-6 and STAT3 Activities. *Bioorganic Chem.* **2019**, *84*, 150–163. https://doi.org/10.1016/j.bioorg.2018.11.032.

(6) Hagar, F. F.; Abbas, S. H.; Sayed, A.; Abdelhamid, D.; Abdel-Aziz, M. New Oxadiazole/ Benzimidazole Hybrids: Design, Synthesis, and Molecular Docking Studies. *J. Adv. Biomed. & Pharm. Sci.* **2023**, *6*, 97-106. DOI: 10.21608/jabps.2023.190430.1179.

(7) Youssif, B. G. M.; Mohamed, A. M.; Osman, E. E. A.; Abou-Ghadir, O. F.; Elnaggar, D. H.; Abdelrahman, M. H.; Treamblu, L.; Gomaa, H. A. M. 5-Chlorobenzofuran-2-Carboxamides: From Allosteric CB1 Modulators to Potential Apoptotic Antitumor Agents. *Eur. J. Med. Chem.* **2019**, *177*, 1–11. https://doi.org/10.1016/j.ejmech.2019.05.040.

(8) B. G. M. Youssif, M. H. Abdelrahman, A. H. Abdelazeem, M. A. Abdelgawad, H. M. Ibrahim, O. I. A. Salem, M. F. A. Mohamed, L. Treambleau, S. N. A. Bukhari, Eur. J. Med. Chem. 2018, 146, 260.

(9) Al-Wahaibi, L. H.; Gouda, A. M.; Abou-Ghadir, O. F.; Salem, O. I. A.; Ali, A. T.; Farghaly, H. S.; Abdelrahman, M. H.; Trembleau, L.; Abdu-Allah, H. H. M.; Youssif, B. G. M. Design and Synthesis of Novel 2,3-Dihydropyrazino[1,2-a]Indole-1,4-Dione Derivatives as Antiproliferative EGFR and BRAFV600E Dual Inhibitors. *Bioorganic Chem.* **2020**, *104*, 104260. https://doi.org/10.1016/j.bioorg.2020.104260.

(10) H. A. M. Gomaa, M. E. Shaker, S. I. Alzarea, O. M. Hendawy, F. A. M. Mohamed, A. M. Gouda, A. T. Ali, M. M. Morcoss, HAGAR ET AL. | 15 of 16 15214184, 0, Downloaded from https://onlinelibrary.wiley.com/doi/10.1002/ardp.202200357 by Egyptian National Sti. Network (Enstinet), Wiley Online Library on [10/11/2022]. See the Terms and Conditions (https://onlinelibrary.wiley.com/terms-and-conditions) on Wiley Online Library for rules of use; OA articles are governed by the applicable Creative Commons License M. H. Abdelrahman, L. Trembleau, B. G. M. Youssif, Bioorg. Chem. 2022, 120, 105616.

(11) Mohamed, F. A. M.; Gomaa, H. A. M.; Hendawy, O. M.; Ali, A. T.; Farghaly, H. S.; Gouda, A. M.; Abdelazeem, A. H.; Abdelrahman, M. H.; Trembleau, L.; Youssif, B. G. M. Design, Synthesis, and Biological Evaluation of Novel EGFR Inhibitors Containing 5-Chloro-3-Hydroxymethyl-Indole-2-Carboxamide Scaffold with Apoptotic Antiproliferative Activity. *Bioorganic Chem.* **2021**, *112*, 104960. https://doi.org/10.1016/j.bioorg.2021.104960.

(12) Mohassab, A. M.; Hassan, H. A.; Abdelhamid, D.; Gouda, A. M.; Youssif, B. G. M.; Tateishi, H.; Fujita, M.; Otsuka, M.; Abdel-Aziz, M. Design and Synthesis of Novel Quinoline/Chalcone/1,2,4-Triazole Hybrids as Potent Antiproliferative Agent Targeting EGFR and BRAFV600E Kinases. *Bioorganic Chem.* **2021**, *106*, 104510. https://doi.org/10.1016/j.bioorg.2020.104510.

(13) Youssif, B. G. M.; Gouda, A. M.; Moustafa, A. H.; Abdelhamid, A. A.; Gomaa, H. A. M.; Kamal, I.; Marzouk, A. A. Design and Synthesis of New Triarylimidazole Derivatives as Dual Inhibitors of BRAFV600E/P38α with Potential Antiproliferative Activity. *J. Mol. Struct.* **2022**, *1253*, 132218. https://doi.org/10.1016/j.molstruc.2021.132218.

(14) Abdelbaset, M. S.; Abdel-Aziz, M.; Abuo-Rahma, G. E.-D. A.; Abdelrahman, M. H.; Ramadan, M.; Youssif, B. G. M. Novel Quinoline Derivatives Carrying Nitrones/Oximes Nitric Oxide Donors: Design, Synthesis, Antiproliferative and Caspase-3 Activation Activities. *Arch. Pharm. (Weinheim)* **2019**, *352* (1), 1800270. https://doi.org/10.1002/ardp.201800270.

(15) Mahmoud, M. A.; Mohammed, A. F.; Salem, O. I. A.; Gomaa, H. A. M.; Youssif, B. G. M. New 1,3,4-Oxadiazoles Linked with the 1,2,3-Triazole Moiety as Antiproliferative Agents Targeting the EGFR Tyrosine Kinase. *Arch. Pharm. (Weinheim)* **2022**, *355* (6), 2200009. https://doi.org/10.1002/ardp.202200009.

(16) Dutta, R.; Lunzer, M. M.; Auger, J. L.; Akgün, E.; Portoghese, P. S.; Binstadt, B. A. A Bivalent Compound Targeting CCR5 and the Mu Opioid Receptor Treats Inflammatory Arthritis Pain in Mice without Inducing Pharmacologic Tolerance. *Arthritis Res. Ther.* **2018**, *20* (1), 154. https://doi.org/10.1186/s13075-018-1661-5.

(17) *Computational Methods in Protein Evolution 2019 | PDF | Proteins | Protein Structure*. Scribd. https://www.scribd.com/document/424702163/Computational-Methods-in-Protein-Evolution-2019 (accessed 2022-11-10).

(18) Morris, G. M.; Huey, R.; Lindstrom, W.; Sanner, M. F.; Belew, R. K.; Goodsell, D. S.; Olson, A. J. AutoDock4 and AutoDockTools4: Automated Docking with Selective Receptor Flexibility. *J. Comput. Chem.* **2009**, *30* (16), 2785–2791. https://doi.org/10.1002/jcc.21256.

(19) Stamos, J.; Sliwkowski, M. X.; Eigenbrot, C. Structure of the Epidermal Growth Factor Receptor Kinase Domain Alone and in Complex with a 4-Anilinoquinazoline Inhibitor. *J. Biol. Chem.* **2002**, *277* (48), 46265–46272. https://doi.org/10.1074/jbc.M207135200.

(20) Bollag, G.; Hirth, P.; Tsai, J.; Zhang, J.; Ibrahim, P. N.; Cho, H.; Spevak, W.; Zhang, C.; Zhang, Y.; Habets, G.; Burton, E. A.; Wong, B.; Tsang, G.; West, B. L.; Powell, B.; Shellooe, R.; Marimuthu, A.; Nguyen, H.; Zhang, K. Y. J.; Artis, D. R.; Schlessinger, J.; Su, F.; Higgins, B.; Iyer, R.; D’Andrea, K.; Koehler, A.; Stumm, M.; Lin, P. S.; Lee, R. J.; Grippo, J.; Puzanov, I.; Kim, K. B.; Ribas, A.; McArthur, G. A.; Sosman, J. A.; Chapman, P. B.; Flaherty, K. T.; Xu, X.; Nathanson, K. L.; Nolop, K. Clinical Efficacy of a RAF Inhibitor Needs Broad Target Blockade in BRAF-Mutant Melanoma. *Nature* **2010**, *467* (7315), 596–599. https://doi.org/10.1038/nature09454.

(21) Eastman, P.; Friedrichs, M. S.; Chodera, J. D.; Radmer, R. J.; Bruns, C. M.; Ku, J. P.; Beauchamp, K. A.; Lane, T. J.; Wang, L.-P.; Shukla, D.; Tye, T.; Houston, M.; Stich, T.; Klein, C.; Shirts, M. R.; Pande, V. S. OpenMM 4: A Reusable, Extensible, Hardware Independent Library for High Performance Molecular Simulation. *J. Chem. Theory Comput.* **2013**, *9* (1), 461–469. https://doi.org/10.1021/ct300857j.

(22) Dallakyan, S.; Olson, A. J. Small-Molecule Library Screening by Docking with PyRx. In *Chemical Biology: Methods and Protocols*; Hempel, J. E., Williams, C. H., Hong, C. C., Eds.; Methods in Molecular Biology; Springer: New York, NY, 2015; pp 243–250. https://doi.org/10.1007/978-1-4939-2269-7_19.
